# The Nitrile Bis-Thiol Bioconjugation Reaction

**DOI:** 10.1021/jacs.3c08762

**Published:** 2023-12-21

**Authors:** Mikesh Patel, Nafsika Forte, Charlie R. Bishop, Michael J. Porter, Matthew Dagwell, Kersti Karu, Vijay Chudasama, James R. Baker

**Affiliations:** † Department of Chemistry, University College London, 20 Gordon Street, London, WC1H 0AJ, U.K.

## Abstract

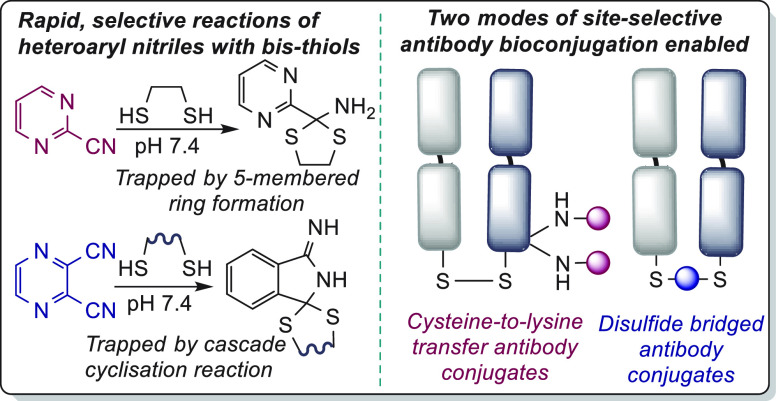

Electron-poor aryl nitriles are promising reagents for bioconjugation due to their high electrophilicity and selectivity for reaction with thiols, albeit generally in a reversible manner. A transient species has previously been observed in such reactions, involving the addition of two thiols to the nitrile functional group, forming a tetrahedral amino dithioacetal (ADTA). In this work, the reaction of heteroaryl nitriles with bis-thiols is explored in an attempt to generate stable ADTAs, which could facilitate new bioconjugation protocols. By use of a 1,2-dithiol, or the incorporation of an electrophilic trap into the aryl nitrile design, the formation of stable products is achieved. The resultant “nitrile bis-thiol” (NBT) reaction is then explored in the context of protein modification, specifically to carry out antibody conjugation. By addition of these nitriles to the reduced disulfide bond of an antibody fragment, it is shown that, depending on the reagent design, cysteine-to-lysine transfer or disulfide bridged NBT products can be generated. Both represent site-selective conjugates and are shown to be stable when challenged with glutathione under physiological conditions and upon incubation in serum. Furthermore, the NBT reaction is tested in the more challenging context of a full antibody, and all four disulfide bonds are effectively modified by these new one-carbon bridging reagents. Overall, this reaction of heteroaryl-nitriles with bis-thiols is shown to be highly efficient and versatile, of tunable reversibility, and offers enticing prospects as a new addition to the toolbox of biocompatible “click”-type reactions.

## Introduction

Nitriles contain a highly polarized triple bond and, as such, are soft electrophiles exhibiting preferential reactivity with thiols over other nucleophiles. This reaction results in the reversible formation of thioimidates, and has been employed extensively in covalent inhibitors targeting active-site cysteine residues.^1^ When electron-poor nitriles are considered, these thioimidates are also susceptible to nucleophilic substitution reactions. For example, Powner and co-workers have implicated the reaction of α-amidonitriles with thiols in prebiotic catalytic peptide ligation, via the formation of thioimidates and their subsequent reaction with amines.^2,3^ An intramolecular version of this S,N-transfer reactivity has also been exploited by Bertozzi and co-workers, who developed an 11-amino acid peptide tag that served to optimize the efficiency of transfer of a nitrile reagent from a cysteine to a lysine.^4^ Notably, when 1,2-amino thiols are employed, the result is cyclization to form stable thiazolines.^5−7^ Known as the nitrile-aminothiol (NAT), or 2-cyanobenzothiazole (CBT), click reaction, it has been widely utilized in applications ranging from selective labeling of N-terminal cysteine residues in peptides and proteins^5^ to *in situ* nanoparticle formation^8^ and nanostructure formation in living cells.^9^ This reaction can also be found in nature, in the synthesis of firefly luciferin.^10^

Recently Bayley and co-workers utilized a protein nanoreactor to study the NAT click, and the reaction of nitriles with a selection of simple thiols, enabling detailed kinetic analyses.^11^ Intriguingly, they identified an additional reaction pathway involving the successive addition of two thiols to nitriles to form a transient tetrahedral product. This species, referred to here as an amino dithioacetal (ADTA), was found to be approximately 80-fold shorter lived than the thioimidate intermediate. In this project, we were motivated to explore whether such amino dithioacetals could be isolated as stable species and thus if a nitrile bis-thiol (NBT) bioconjugation reaction could be developed. A particular motivation was whether this reaction could be an effective strategy for the site-selective construction of antibody conjugates.

Antibody conjugates combine the exquisite targeting ability of antibodies with the diverse functionality of small molecules to generate a variety of constructs. Examples include antibody drug conjugates (ADCs),^12,13^ radio-immunoconjugates,^14^ antibody nanoparticle conjugates^15^ and targeted imaging agents.^16^ With such systems, it has been identified that site-selective modification strategies result in superior conjugates, including exhibiting improved *in vivo* properties.^17^ While a number of strategies are being applied for the construction of site-selective ADCs,^18^ such as engineering antibodies suitable for single cysteine or enzymatic conjugation, the targeting of disulfide bonds with bridging reagents represents a particularly appealing option with the advantage of being able to conjugate antibodies taken directly “off-the-shelf”.^19^ These bridging reagents overcome the limitations associated with modifying each cysteine residue of a reduced cystine separately, which results in a loss of this structurally stabilizing motif. It also allows a controlled conjugation stoichiometry of one attachment per disulfide. A range of reagents have been developed to effect this bridging of disulfides in antibodies, including next generation maleimides (NGMs),^20−22^ pyridazinediones (PDs),^23−25^ bis-sulfones,^26,27^ divinylpyrimidines,^28,29^ divinyltriazines,^30^ arylenedipropiolonitriles,^31^ and diethynyl phosphinates.^32^ Recently an example of a one-carbon bridging reagent was also described, referred to as an oxSTEF motif, via two conjugate addition−elimination mechanisms.^33^ Such linkers are desirable as they minimize the imposed distance extension between the two sulfur atoms. While this is unlikely to be of significance in large, structurally stable motifs such as antibodies, this is still a favorable design feature and may have greater significance when applied in the context of more structurally sensitive peptides or proteins. We envisaged that an NBT reaction could be an intriguing alternative approach to accessing one-carbon disulfide bridged bioconjugates. We were also interested in the prospects of this reaction more generally, as it would represent a unique mechanism for linking two thiols, and controlling the dynamic nature of the nitrile-thiol chemistry would be a key challenge.

## Results and Discussion

**Figure 1:**
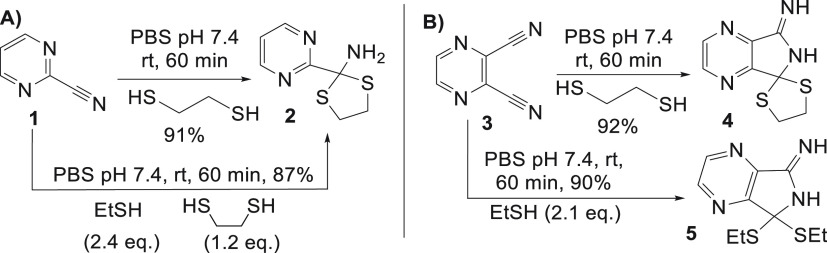
Initial examples of the nitrile bisthiol (NBT) reaction to afford isolable amino dithioacetals (ADTAs). (A) Use of 1,2-ethanedithiol to form a stable, cyclic ADTA. (B) A cascade reaction is observed with pyrazine bis-nitrile, in which the ADTA amine is trapped by the second nitrile.

To test the viability of the NBT reaction, we selected 2-cyanopyrimidine **1** as the model electron deficient aryl nitrile, as it had been reported to react rapidly with cysteine^8,34^ and would be a readily modifiable structure. 1,2-Ethanedithiol was used as the bis-thiol, as it was hypothesized that 5-membered ring formation would be favorable and may afford an isolable product. Indeed, carrying out this reaction under buffered aqueous conditions (pH 7.4) rapidly generated the desired ADTA **2** (Figure 1A), in 91% isolated yield. A similar outcome was observed when a water-soluble, nonpungent 1,2-dithiol (2,3-dimercapto-1-propanesulfonic acid, DMPS) was employed, which allowed a convenient *in situ* NMR rate analysis (see Supporting Information (SI) S49). A second-order rate constant of 0.08 ± 0.01 M^–1^ s^–1^ was determined, which places the NBT reaction as comparable with other biorthogonal reactions^35^ (such as strain-promoted alkyne–azide click, SPAAC) and will be readily tunable by reagent design (e.g., incorporating an electron-withdrawing *p*-amide group already affords a ∼4-fold acceleration, see SI S49). ADTA **2** was found to be stable in buffer (PBS 7.4, 22 °C) overnight, as well as upon addition of stoichiometric equivalents of ethanethiol (see SI Figures S1 and S2). The reaction was found to be highly selective for bis-thiols, with only unstable thioimidate species observed upon addition of monothiols; and a competition reaction yielded ADTA **2** as the sole product (Figure 1A). This confirmed the dynamic nature of the initial thioimidate formation and the “trapping” of the ADTA species via the dithiol cyclization step.

Trialling the NBT reaction of 2-cyanopyrimidine **1** with 1,3-propanedithiol revealed that the 6-membered ADTA was formed in an analogous manner by *in situ* NMR analysis. Intriguingly, upon isolation, while this ADTA is observed as the only product in D_2_O it forms a 1:2.6 mixture with thioimidates in CD_3_CN (S7). This is consistent with it being a more dynamic example of the NBT reaction, with water playing a role in stabilizing the ADTA. Indeed, addition of the 1,2- and 1,3-dithiols together led to complete selectivity for the 5-membered ADTA (even with 5 equiv of the 1,3-dithiol, see SI Figure S3). DFT calculations supported this outcome, indicating that the five-membered ring product **2** had a free energy 23 kJ mol^–1^ lower than that of the thioimidate arising from addition of one of the two SH groups to nitrile **1**. By contrast, the corresponding free energy difference for the six-membered ADTA was only 16 kJ mol^–1^ lower. Notably, due to the dynamic nature of the NBT reaction, formation of ADTA **2** could be reversed by the addition of the rapid thiol capping reagent *N*-methyl maleimide. This resulted in complete regeneration of the pyrimidine nitrile in just 2 h (see SI Figure S4). This indicates the potential for EDT-related nitrile protecting groups, and indeed broadly in other applications where reversible click-chemistries are employed.^36−38^

This initial study revealed 1,2-dithiols as a special-case to enable an NBT reaction, due to the formation of a 5-membered ring. To facilitate NBT reactions more widely, we envisaged another class of nitrile reagents, which would incorporate an electrophilic trap for the amino group formed in the ADTA intermediate. Pyrazine bis-nitrile **3** was identified as a convenient example due to its symmetry, and as the second nitrile could serve to facilitate an intramolecular cyclization (Figure 1B). Indeed, reaction of pyrazine **3** with a bis- or monothiol afforded ADTAs **4** and **5** in very high isolated yields, confirming that the reversible nature of the reaction can be overcome by use of an electrophilic trap within the molecular design.

Attention then shifted to exploring the application of such aryl nitriles in antibody conjugation. We hypothesized that upon reaction with bis-thiols generated from disulfide reduction, ADTA intermediates would be formed. In the absence of the electrophilic trap, these would likely be transient intermediates and would interconvert with thioimidates, offering the prospect of their subsequent reaction with nearby lysine residues to form site-selective cysteine-to-lysine transfer (CLT) conjugates (similar to that observed with thioesters^39^). Alternatively in the presence of the second electrophilic group stable NBT conjugates could be formed, which would represent a new one-carbon disulfide bridging strategy.

**Figure 2:**
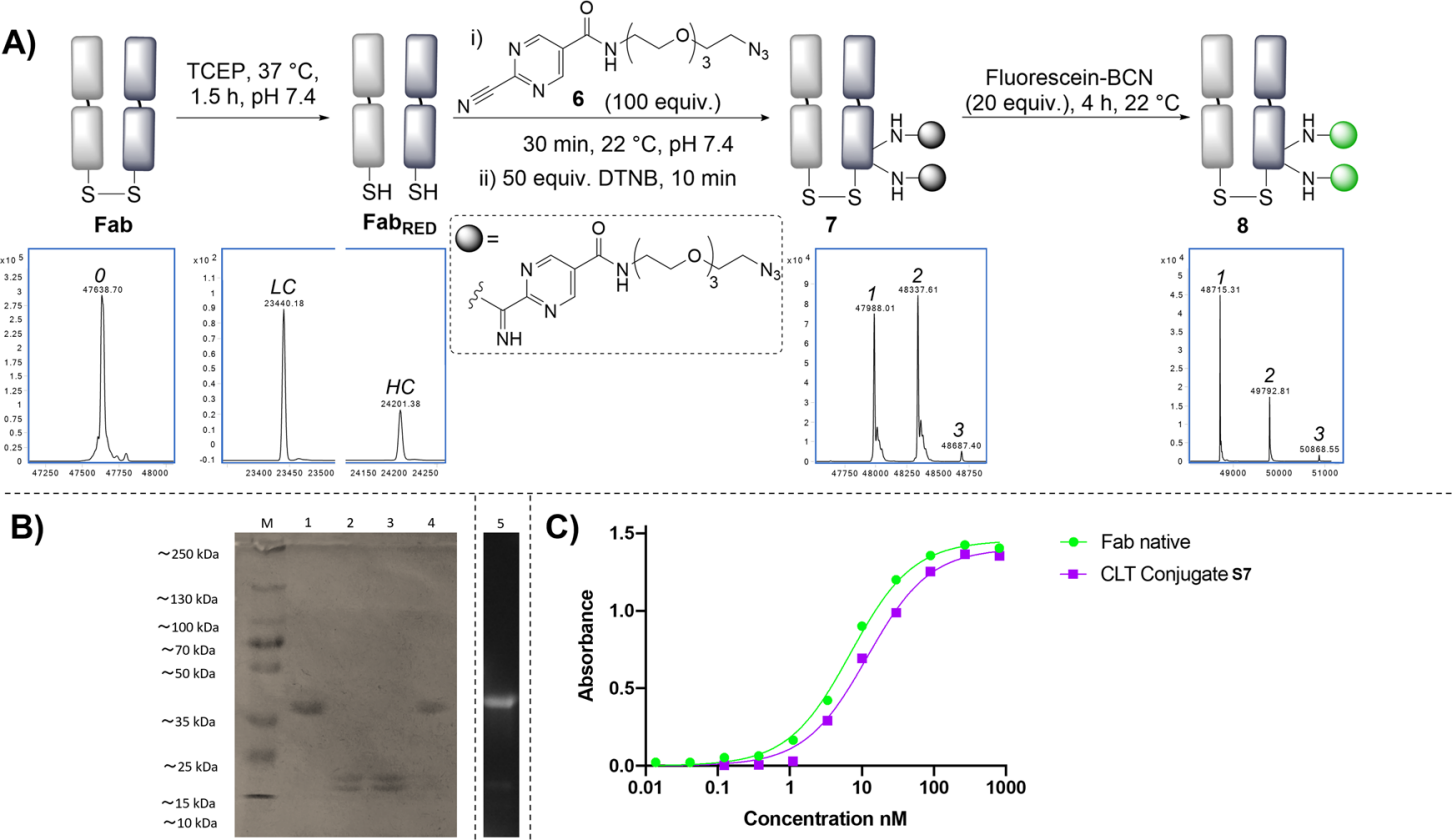
Cysteine-to-lysine transfer (CLT) with aryl nitrile (**6**) (A) general optimized scheme for CLT followed by SPAAC to generate functional conjugates, LC = Light chain, HC = Heavy chain (B) SDS-PAGE analysis: (M) molecular marker, (1) native Fab, (2) reduced Fab, (3) nitrile CLT (1 h), (4) after DTNB disulfide reoxidation, (5) fluorescent imaging of conjugate **8**. (C) ELISA data for Fab CLT conjugate **S7**.

To test these hypotheses, we utilized the Fab fragment of Her-2 targeting breast cancer drug trastuzumab. This Fab contains a single disulfide, allowing clear analysis of the outcomes of the bioconjugation, while also containing 26 lysine residues which would challenge the selectivity of the methods. Treatment of the Fab fragment with 2-cyanopyrimidine **1** (100 equiv, pH 7.4 for 1 h, RT) led to no reaction, confirming the absence of background lysine reactivity. Reduction of the Fab disulfide with TCEP generated two free cysteines (Fab_RED_) which upon addition of the 2-cyanopyrimidine **1**, or its azido analogue **6** primed for functionalization, led to an observed bioconjugation reaction to give a distribution with 1, 2, and 3 attachments as the major products (Figure 2). Addition of Ellman’s reagent led to reformation of the disulfide bond in these conjugates, revealing that they were not modifications on the cysteines but rather amidines (e.g., **7**) formed on proximal lysine residues by rapid CLT. This is consistent with previous work showing thioimidates undergo S,N-transfer,^2−4^ and suggests that any amino dithioacetal formation is reversible as anticipated. Trypsin digest, followed by MS/MS, confirmed that attachment had indeed taken place on proximal lysines (heavy chain K136, K221 and K225, and light chain K190, see SI S46). This is consistent with that observed previously for CLT using thioesters, with the exception of K190.^39^

This lysine transfer is notably remarkably efficient (pH 7.4 for 1 h, RT), given that on an engineered peptide the CLT required up to 40 h to achieve 35–41% conversion,^4^ indicating that the structured Fab and amino-group proximity afforded a greater acceleration to the transfer reaction. We continued to investigate the impact of the reaction conditions and reagent design on the CLT outcome. For example, the reaction with pyrimidine **1** (100 equiv., pH 7.4, RT) was analyzed at 15 min, 30 min, and 1 h, with the average loading increasing in each case, and a final loading of ∼1.8 (calculation by LCMS, which assumes that the conjugates ionize to a similar degree). This demonstrated that the distribution of conjugates could be controlled to achieve a higher loading as desired. However, despite trialling various conditions (e.g., pH, temp, and equiv) it was not possible to obtain a completely homogeneous conjugate, as a statistical distribution was always obtained. This is presumably due to the reversible nature of the thioimidate formation coupled with efficient transfer to the different proximal lysines. Intriguingly, unlike the thioesters, the aryl nitrile reagents do not appear to undergo any competing hydrolysis. For example, stoichiometric addition of **1** (2 equiv., pH 7.4 for 24 h, RT) resulted in quantitative conversion to afford a distribution of products with an average loading of ∼1.9, indicating that the reagent is not hydrolytically labile (see SI Figure S10). The effect of reagent reactivity was also explored, with more electron-poor heteroaromatics, such as a *p-*trifluoromethyl pyrimidine nitrile or a dimethyl-1,2,4-triazine nitrile, also undergoing effective CLT, showing a similar loading under the same reaction conditions (SI Figures S41 and S42).

Attachment of fluorescein-PEG-BCN by a strain-promoted azide–alkyne click (SPAAC) generated functional Fab conjugate **8**. While the amount of 2-loaded species appears to decrease by LCMS, this is likely due to the significant mass differences impacting on the accuracy of LCMS quantification, as UV/vis confirmed the average loading remained at ∼1.5. The 2-cyanopyrimidine conjugate was shown, by LCMS analyses (see SI Figures S36 and S40), to be stable for at least 24 h in blood concentrations of glutathione (5 μM, pH 7.4, 37 °C) and endosomal concentrations of glutathione (5 mM glutathione, pH 6.5, 37 °C).^40^ These conjugates also demonstrated full retention of binding activity to HER2, confirmed via ELISA analysis. Fab conjugate **8** showed no evidence of fluorophore transfer by SDS-PAGE analysis when incubated in serum for 37 °C, 5 days (see SI Figure S45).

**Figure 3:**
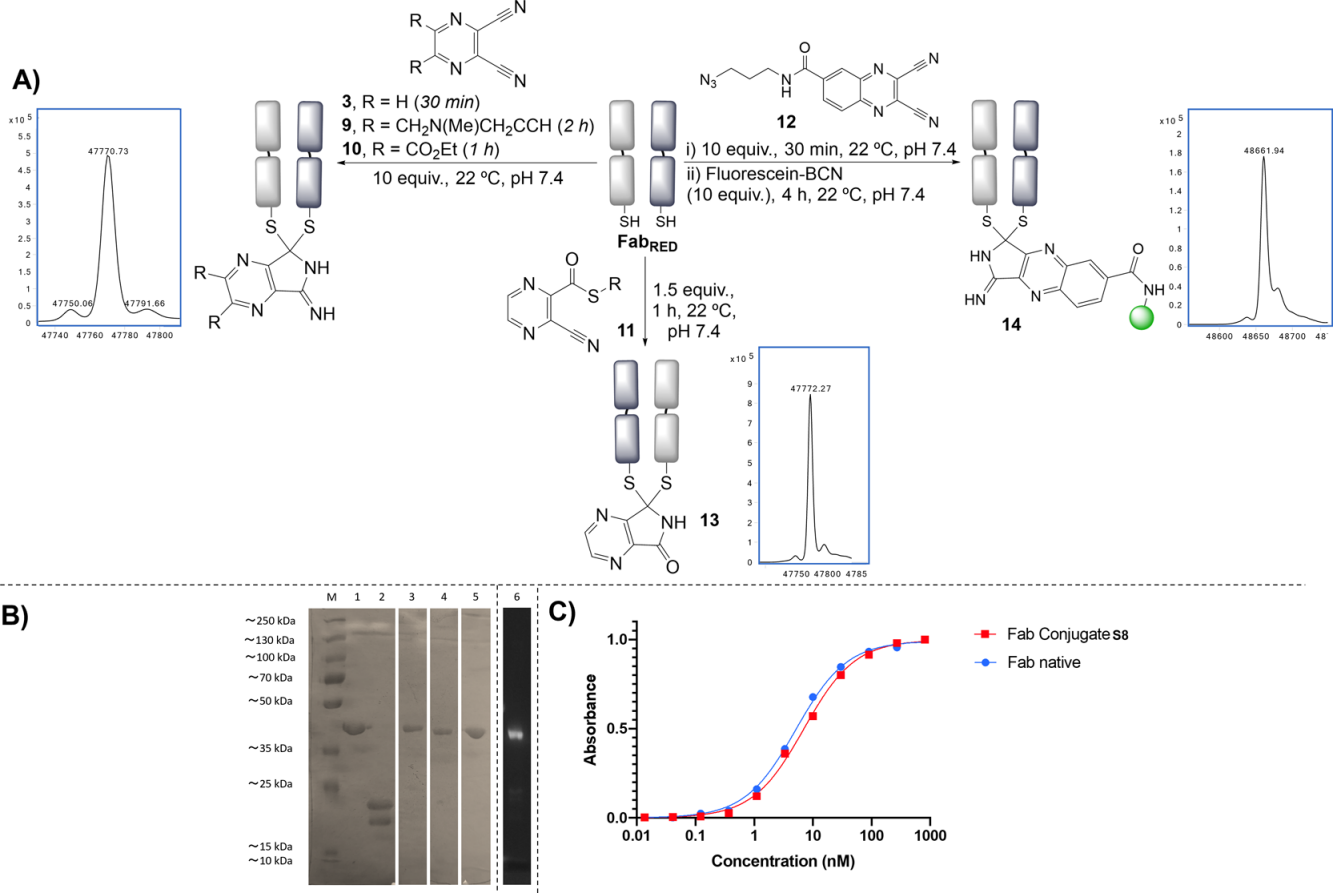
(A) Site-selective disulfide rebridging with a library of bis-nitrile reagents. (B) SDS-PAGE analysis: (M) molecular marker, (1) native Fab, (2) reduced Fab, (3) Fab bridge with **3**, (4) Fab bridge with **12**, (5) Fab bridge with **11**, (6) fluorescent imaging of conjugate **14**. (C) ELISA data for the Fab conjugate **S8**.

In order to generate NBT antibody conjugates, we next trialled trapping aryl-nitrile designs. Treatment of the reduced Fab with pyrazine bis-nitrile **3** resulted in the rapid formation of a one-carbon disulfide bridged species (Figure 3). The resultant bridge was found to be stable to addition of both 2-mercaptoethanol and cysteine (100 equiv, pH 7.4 for 2 h at 37 °C). Initial efforts to functionalize this pyrazine core led to bis-alkyne **9**, albeit this reagent was synthesized in low yield presumed to be due to instability caused by the presence of acidic benzylic hydrogens present on the benzylic bromide precursor. Despite this, pyrazine **9** was carried forward and demonstrated effective antibody conjugation (SI Figure S18). However, subsequent CuAAC functionalization led to cleavage of the conjugate and the restoration of the native disulfide bond to afford unmodified Fab (see SI Figure S19). Small molecule studies utilizing **5** disclosed a sensitivity to high concentrations of CuSO_4_, with the hypothesized mechanism of release akin to that of copper mediated deprotection of thiazolidine derivatives.^41^

To overcome the issue of poor synthesis yield of the reagent and instability, a quinoxaline-nitrile analogue **S4** was readily accessed, in 2 steps from 3,4-diaminobenzoic acid (see SI), which contained a pendant alkyne. The quinoxaline core proved to be more stable, demonstrating effective antibody conjugation and successful subsequent CuAAC functionalization with an optimized quantity of CuSO_4_ (5 equiv). The reasoning behind the greater copper stability of the quinoxaline analogue is not yet fully understood and will be further explored along with the enticing prospect of high concentration metal ion triggered release for the controlled triggered cleavage of pyrazine bis-nitrile NBT conjugates. Alternatively, azide functionalized quinoxaline analogue **12** enabled antibody conjugation followed by SPAAC attachment of fluorescein, conveniently affording the final fluorescent conjugate **14**. As with CLT conjugates, the bridged species was also found to be stable under physiological conditions (serum and early endosomal conditions tested, as described above, see SI Figures S34 and S38) while demonstrating full retention of binding activity to HER2. A range of other pyrazine analogues were trialled to explore the scope of this cascade reaction and indicated significant diversification of design and properties proved viable. For example, bis-ester **10** was found to be a more reactive version (see SI Figure S21), with the conjugation in this case being reversible by addition of cysteine (100 equiv, pH 7.4 for 3 h at 37 °C). Incorporation of a thioester in reagent **11** successfully led to an analogous amide NBT conjugate **13**, demonstrating that this cascade reaction is not limited to bis-nitriles.

Due to the dynamic mechanism of the NBT reaction, it was hypothesized that these disulfide bridging reagents may be tolerant to the presence of competing monothiols. This may provide evidence that the NBT reaction could be employed in the selective labeling of bis-thiol containing proteins in more complex biological mixtures. Indeed, a competition reaction between the peptidic monothiol glutathione and the reduced Fab at a 3:1 ratio, with 1.2 equiv of the bis-nitrile quinoxaline **S3**, yielded the fully rebridged species (see SI Figure S32). This can be compared with an equivalent reaction using *N*-methyl maleimide which led to minimal protein labeling, as the maleimide preferentially reacts with the glutathione (see SI Figure S31).

**Figure 4:**
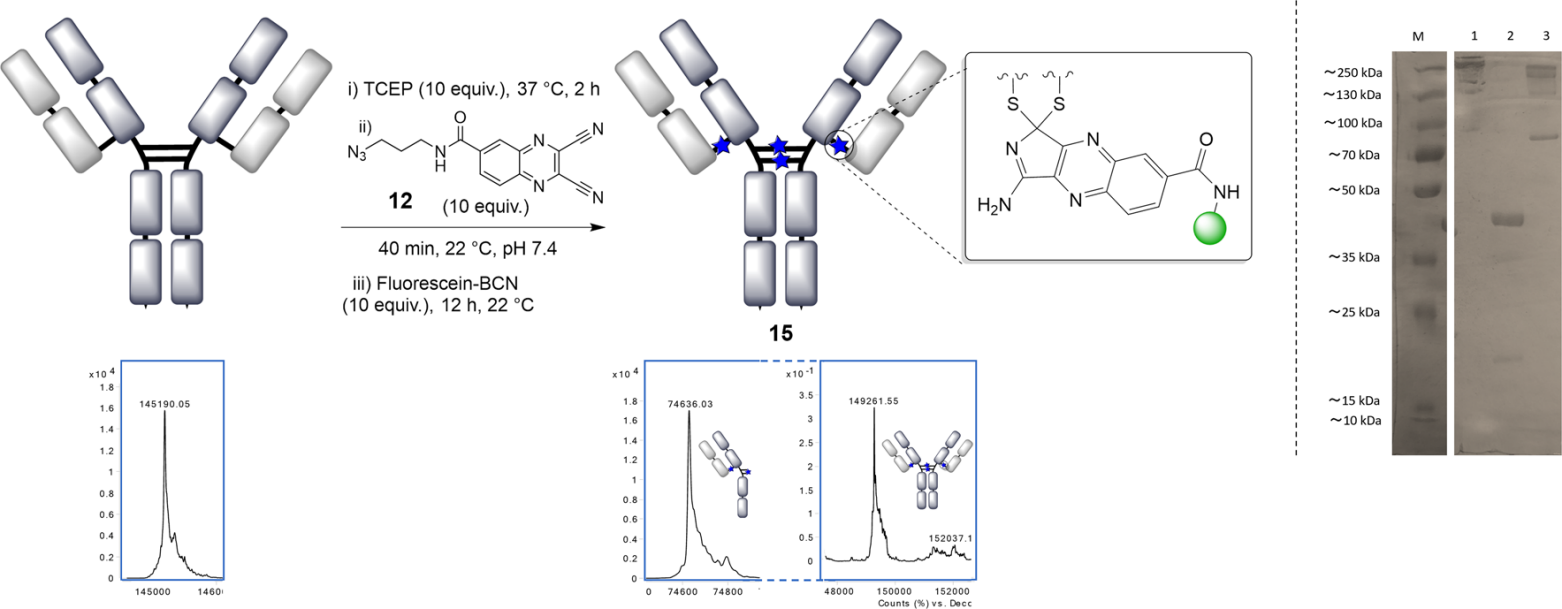
(a) General scheme for the rebridging of trastuzumab with **12**, followed by SPAAC functionalization (dashed line indicating two different regions of the same LCMS trace), (b) SDS-PAGE analysis: (M) molecular marker, (1) native mAb, (2) reduced mAb, (3) mAb bridge with **12**.

The NBT reaction was trialled on the more complex system of trastuzumab, a full IgG1 antibody which contains 4 interchain disulfide bonds. Quinoxaline bis-nitrile **12** was able to efficiently rebridge all 4 disulfide bonds, then undergo SPAAC to yield the final conjugate **15** (Figure 4). As for other disulfide bridging reagents, two regioisomers are formed due to disulfide scrambling in the hinge region,^22,28^ as observed by the half an tibody along with the full antibody conjugate in denaturing LCMS and SDS-PAGE analyses. Finally, to demonstrate applicability to other protein classes, thioredoxin, a bis-thiol containing enzyme, was modified effectively (see SI Figure S44) with the pyrazine bis-nitrile **3** (10 equiv, pH 8.0, 2.5 h), indicating prospective applications of such reagents in selective covalent inhibition of such enzymes.

## Conclusions

In conclusion, we have demonstrated that electron-deficient aryl nitriles undergo efficient reactions with bis-thiols to form amino dithioacetals, which can be trapped as isolable products. By use of a 1,2-dithiol, pyrimidine nitriles can be captured in near-quantitative yields due to the favored formation of a 5-membered ADTA, even in the presence of other competing thiols. Alternatively, by incorporating an adjacent electrophilic trap within the aryl nitrile reagent, a cascade reaction results, capturing the ADTA product as a stable species. Challenging these reactions to the site-selective modification of antibodies, we report that bis-thiols generated from the reduction of interchain disulfide bonds are able to react with aryl nitriles to generate valuable site-selective conjugates. In the case of the pyrimidine nitriles, the transient ADTA intermediate is not observed; instead, an efficient S,N-transfer pathway is followed, to generate proximity labeled lysine conjugates. Alternatively, by use of the heteroaromatic bis-nitrile species, one-carbon disulfide bridged NBT conjugates are accessed. Both these lysine and disulfide bridged antibody conjugates are shown to be robustly stable to physiological thiols (e.g., glutathione) and represent new reagent classes for site-selective antibody conjugation. More generally, the NBT reaction represents a new biorthogonal click reaction which could offer diverse new opportunities. The dynamic nature of the mechanism differentiates it from the related nitrile amino thiol (NAT), and this is likely to prove extremely tunable by the choice of the bisthiol and electron-poor nitrile. Applications can be envisaged stretching from highly selective biorthogonal reactions in complex biological media (e.g., for selective protein labeling and pull-down assays) to reversible surface functionalization, and new dynamic materials.

## Supporting Information Available

Synthetic chemistry experimental details, including procedures, compound characterisations, kinetic analysis and DFT calculations. Chemical biology experimental details, including bioconjugation procedures and LC-MS analysis of bioconjugates (PDF)

## References

[ref1] Bonatto V.; Lameiro R. F.; Rocho F. R.; Lameira J.; Leitão A.; Montanari C. A. Nitriles: an attractive approach to the development of covalent inhibitors. RSC Med. Chem. 2023, 14 (2), 201–217. 10.1039/D2MD00204C. 36846367 PMC9945868

[ref2] Foden C. S.; Islam S.; Fernández-García C.; Maugeri L.; Sheppard T. D.; Powner M. W. Prebiotic synthesis of cysteine peptides that catalyze peptide ligation in neutral water. Science 2020, 370 (6518), 865–869. 10.1126/science.abd5680. 33184216

[ref3] Singh J.; Whitaker D.; Thoma B.; Islam S.; Foden C. S.; Aliev A. E.; Sheppard T. D.; Powner M. W. Prebiotic Catalytic Peptide Ligation Yields Proteinogenic Peptides by Intramolecular Amide Catalyzed Hydrolysis Facilitating Regioselective Lysine Ligation in Neutral Water. J. Am. Chem. Soc. 2022, 144 (23), 10151–10155. 10.1021/jacs.2c03486. 35640067 PMC9204760

[ref4] Keyser S. G. L.; Utz A.; Bertozzi C. R. Computation-Guided Rational Design of a Peptide Motif That Reacts with Cyanobenzothiazoles via Internal Cysteine–Lysine Relay. J. Org. Chem. 2018, 83 (14), 7467–7479. 10.1021/acs.joc.8b00625. 29771122 PMC6258261

[ref5] Ren H.; Xiao F.; Zhan K.; Kim Y.-P.; Xie H.; Xia Z.; Rao J. A Biocompatible Condensation Reaction for the Labeling of Terminal Cysteine Residues on Proteins. Angew. Chem., Int. Ed. 2009, 48 (51), 9658–9662. 10.1002/anie.200903627. PMC4878437 19924746

[ref6] Zhang M.; Liang G. Applications of CBT-Cys click reaction: past, present, and future. Sci. China Chem. 2018, 61 (9), 1088–1098. 10.1007/s11426-018-9277-6.

[ref7] Zhu Y.; Zhang X.; You Q.; Jiang Z. Recent applications of CBT-Cys click reaction in biological systems. Bioorg. Med. Chem. 2022, 68, 116881 10.1016/j.bmc.2022.116881. 35716587

[ref8] Chen Z.; Chen M.; Cheng Y.; Kowada T.; Xie J.; Zheng X.; Rao J. Exploring the Condensation Reaction between Aromatic Nitriles and Amino Thiols To Optimize In Situ Nanoparticle Formation for the Imaging of Proteases and Glycosidases in Cells. Angew. Chem., Int. Ed. 2020, 59 (8), 3272–3279. 10.1002/anie.201913314. PMC7012687 31828913

[ref9] Liang G.; Ren H.; Rao J. A biocompatible condensation reaction for controlled assembly of nanostructures in living cells. Nat. Chem. 2010, 2 (1), 54–60. 10.1038/nchem.480. 21124381 PMC3196337

[ref10] White E. H.; Wörther H.; Field G. F.; McElroy W. D. Analogs of Firefly Luciferin. J. Org. Chem. 1965, 30 (7), 2344–2348. 10.1021/jo01018a054. 5931937

[ref11] Qing Y.; Liu M. D.; Hartmann D.; Zhou L.; Ramsay W. J.; Bayley H. Single-Molecule Observation of Intermediates in Bioorthogonal 2-Cyanobenzothiazole Chemistry. Angew. Chem., Int. Ed. 2020, 59 (36), 15711–15716. 10.1002/anie.202005729. PMC7496719 32589803

[ref12] Drago J. Z.; Modi S.; Chandarlapaty S. Unlocking the potential of antibody–drug conjugates for cancer therapy. Nat. Rev. Clin. Oncol. 2021, 18 (6), 327–344. 10.1038/s41571-021-00470-8. 33558752 PMC8287784

[ref13] Dumontet C.; Reichert J. M.; Senter P. D.; Lambert J. M.; Beck A. Antibody–drug conjugates come of age in oncology. Nat. Rev. Drug Discovery 2023, 22 (8), 641–661. 10.1038/s41573-023-00709-2. 37308581

[ref14] Nasr D.; Kumar P. A.; Zerdan M. B.; Ghelani G.; Dutta D.; Graziano S.; Lim S. H. Radioimmunoconjugates in the age of modern immuno-oncology. Life Sci. 2022, 310, 121126 10.1016/j.lfs.2022.121126. 36309222

[ref15] Johnston M. C.; Scott C. J. Antibody conjugated nanoparticles as a novel form of antibody drug conjugate chemotherapy. Drug Discovery Today Technol. 2018, 30, 63–69. 10.1016/j.ddtec.2018.10.003. 30553522

[ref16] Parakh S.; Lee S. T.; Gan H. K.; Scott A. M. Radiolabeled Antibodies for Cancer Imaging and Therapy. Cancers 2022, 14 (6), 1454 10.3390/cancers14061454. 35326605 PMC8946248

[ref17] Zhou Q. Site-Specific Antibody Conjugation for ADC and Beyond. Biomedicines 2017, 5 (4), 64 10.3390/biomedicines5040064. 29120405 PMC5744088

[ref18] Walsh S. J.; Bargh J. D.; Dannheim F. M.; Hanby A. R.; Seki H.; Counsell A. J.; Ou X.; Fowler E.; Ashman N.; Takada Y.; Isidro-Llobet A.; Parker J. S.; Carroll J. S.; Spring D. R. Site-selective modification strategies in antibody–drug conjugates. Chem. Soc. Rev. 2021, 50 (2), 1305–1353. 10.1039/D0CS00310G. 33290462

[ref19] Forte N.; Chudasama V.; Baker J. R. Homogeneous antibody-drug conjugates via site-selective disulfide bridging. Drug Discovery Today Technol. 2018, 30, 11–20. 10.1016/j.ddtec.2018.09.004. 30553515

[ref20] Schumacher F. F.; Nunes J. P. M.; Maruani A.; Chudasama V.; Smith M. E. B.; Chester K. A.; Baker J. R.; Caddick S. Next generation maleimides enable the controlled assembly of antibody-drug conjugates via native disulfide bond bridging. Org. Biomol. Chem. 2014, 12 (37), 7261–9. 10.1039/C4OB01550A. 25103319 PMC4159697

[ref21] Nunes J. P.; Morais M.; Vassileva V.; Robinson E.; Rajkumar V. S.; Smith M. E.; Pedley R. B.; Caddick S.; Baker J. R.; Chudasama V. Functional native disulfide bridging enables delivery of a potent, stable and targeted antibody-drug conjugate (ADC). Chem. Commun. 2015, 51 (53), 10624–7. 10.1039/C5CC03557K. 26051118

[ref22] Morais M.; Nunes J. P. M.; Karu K.; Forte N.; Benni I.; Smith M. E. B.; Caddick S.; Chudasama V.; Baker J. R. Optimisation of the dibromomaleimide (DBM) platform for native antibody conjugation by accelerated post-conjugation hydrolysis. Org. Biomol. Chem. 2017, 15 (14), 2947–2952. 10.1039/C7OB00220C. 28290574

[ref23] Maruani A.; Smith M. E. B.; Miranda E.; Chester K. A.; Chudasama V.; Caddick S. A plug-and-play approach to antibody-based therapeutics via a chemoselective dual click strategy. Nat. Commun. 2015, 6, 6645 10.1038/ncomms7645. 25824906 PMC4389247

[ref24] Robinson E.; Nunes J. P. M.; Vassileva V.; Maruani A.; Nogueira J. C. F.; Smith M. E. B.; Pedley R. B.; Caddick S.; Baker J. R.; Chudasama V. Pyridazinediones deliver potent, stable, targeted and efficacious antibody-drug conjugates (ADCs) with a controlled loading of 4 drugs per antibody. RSC Adv. 2017, 7 (15), 9073–9077. 10.1039/C7RA00788D.

[ref25] Bahou C.; Richards D. A.; Maruani A.; Love E. A.; Javaid F.; Caddick S.; Baker J. R.; Chudasama V. Highly homogeneous antibody modification through optimization of the synthesis and conjugation of functionalised dibromopyridazinediones. Org. Biomol. Chem. 2018, 16 (8), 1359–1366. 10.1039/C7OB03138F. 29405223 PMC6058253

[ref26] Balan S.; Choi J. W.; Godwin A.; Teo I.; Laborde C. M.; Heidelberger S.; Zloh M.; Shaunak S.; Brocchini S. Site-specific PEGylation of protein disulfide bonds using a three-carbon bridge. Bioconjugate Chem. 2007, 18 (1), 61–76. 10.1021/bc0601471. 17226958

[ref27] Badescu G.; Bryant P.; Bird M.; Henseleit K.; Swierkosz J.; Parekh V.; Tommasi R.; Pawlisz E.; Jurlewicz K.; Farys M.; Camper N.; Sheng X. B.; Fisher M.; Grygorash R.; Kyle A.; Abhilash A.; Frigerio M.; Edwards J.; Godwin A. Bridging Disulfides for Stable and Defined Antibody Drug Conjugates. Bioconjugate Chem. 2014, 25 (6), 1124–1136. 10.1021/bc500148x. 24791606

[ref28] Walsh S. J.; Omarjee S.; Galloway W. R. J. D.; Kwan T. T. L.; Sore H. F.; Parker J. S.; Hyvönen M.; Carroll J. S.; Spring D. R. A general approach for the site-selective modification of native proteins, enabling the generation of stable and functional antibody–drug conjugates. Chem. Sci. 2019, 10 (3), 694–700. 10.1039/C8SC04645J. 30774870 PMC6349026

[ref29] Dannheim F. M.; Walsh S. J.; Orozco C. T.; Hansen A. H.; Bargh J. D.; Jackson S. E.; Bond N. J.; Parker J. S.; Carroll J. S.; Spring D. R. All-in-one disulfide bridging enables the generation of antibody conjugates with modular cargo loading. Chem. Sci. 2022, 13 (30), 8781–8790. 10.1039/D2SC02198F. 35975158 PMC9350601

[ref30] Counsell A. J.; Walsh S. J.; Robertson N. S.; Sore H. F.; Spring D. R. Efficient and selective antibody modification with functionalised divinyltriazines. Org. Biomol. Chem. 2020, 18 (25), 4739–4743. 10.1039/D0OB01002B. 32608446

[ref31] Koniev O.; Dovgan I.; Renoux B.; Ehkirch A.; Eberova J.; Cianférani S.; Kolodych S.; Papot S.; Wagner A. Reduction–rebridging strategy for the preparation of ADPN-based antibody–drug conjugates. MedChemComm 2018, 9 (5), 827–830. 10.1039/C8MD00141C. 30108971 PMC6071946

[ref32] Stieger C. E.; Franz L.; Körlin F.; Hackenberger C. P. R. Diethynyl Phosphinates for Cysteine-Selective Protein Labeling and Disulfide Rebridging. Angew. Chem., Int. Ed. 2021, 60 (28), 15359–15364. 10.1002/anie.202100683. PMC8362001 34080747

[ref33] Nisavic M.; Wørmer G. J.; Nielsen C. S.; Jeppesen S. M.; Palmfeldt J.; Poulsen T. B. oxSTEF Reagents Are Tunable and Versatile Electrophiles for Selective Disulfide-Rebridging of Native Proteins. Bioconjugate Chem. 2023, 34 (6), 994–1003. 10.1021/acs.bioconjchem.3c00005. 37201197

[ref34] Berteotti A.; Vacondio F.; Lodola A.; Bassi M.; Silva C.; Mor M.; Cavalli A. Predicting the Reactivity of Nitrile-Carrying Compounds with Cysteine: A Combined Computational and Experimental Study. ACS Med. Chem. Lett. 2014, 5 (5), 501–505. 10.1021/ml400489b. 24900869 PMC4027605

[ref35] Lang K.; Chin J. W. Bioorthogonal Reactions for Labeling Proteins. ACS Chem. Biol. 2014, 9 (1), 16–20. 10.1021/cb4009292. 24432752

[ref36] Chatterjee S.; Anslyn E. V.; Bandyopadhyay A. Boronic acid based dynamic click chemistry: recent advances and emergent applications. Chem. Sci. 2021, 12 (5), 1585–1599. 10.1039/D0SC05009A. PMC8179052 34163920

[ref37] Wu C.-S.; Cheng L. Recent Advances towards the Reversible Chemical Modification of Proteins. ChemBioChem. 2023, 24 (2), e202200468 10.1002/cbic.202200468. 36201252

[ref38] Chakma P.; Konkolewicz D. Dynamic Covalent Bonds in Polymeric Materials. Angew. Chem., Int. Ed. 2019, 58 (29), 9682–9695. 10.1002/anie.201813525. 30624845

[ref39] Forte N.; Benni I.; Karu K.; Chudasama V.; Baker J. R. Cysteine-to-lysine transfer antibody fragment conjugation. Chem. Sci. 2019, 10 (47), 10919–10924. 10.1039/C9SC03825F. 32190247 PMC7066670

[ref40] Bahou C.; Szijj P. A.; Spears R. J.; Wall A.; Javaid F.; Sattikar A.; Love E. A.; Baker J. R.; Chudasama V. A Plug-and-Play Platform for the Formation of Trifunctional Cysteine Bioconjugates that also Offers Control over Thiol Cleavability. Bioconjugate Chem. 2021, 32 (4), 672–679. 10.1021/acs.bioconjchem.1c00057. PMC8154211 33710874

[ref41] Naruse N.; Kobayashi D.; Ohkawachi K.; Shigenaga A.; Otaka A. Copper-Mediated Deprotection of Thiazolidine and Selenazolidine Derivatives Applied to Native Chemical Ligation. J. Org. Chem. 2020, 85 (3), 1425–1433. 10.1021/acs.joc.9b02388. 31592642

