# Supplementary material for: The Nitrile Bis-Thiol Bioconjugation Reaction
Source: J Am Chem Soc. 2023 Dec 21;146(1):274–80. doi: 10.1021/jacs.3c08762 (PMC10786040; doi:10.1021/jacs.3c08762)
Supplement: Supplementary file 1 — ja3c08762_si_001.pdf [file ja3c08762_si_001.pdf]

The Nitrile Bis-Thiol Bioconjugation Reaction - ESI

Mikesh Patel,[†] Nafsika Forte,[†] Charlie R. Bishop,[†] Michael J. Porter,[†] Matthew Dagwell,[†] Kersti Karu,[†] Vijay Chudasama,[†,*] and James R. Baker[†,*]

† Department of Chemistry, University College London, 20 Gordon Street, London, WC1H 0AJ, UK. Email j.r.baker@ucl.ac.uk, v.chudasama@ucl.ac.uk.

Table of Contents

1. Chemical synthesis general remarks 3
2. Synthesis and characterisation of compounds 3
   N-(2-(2-(2-(2-azidoethoxy)ethoxy)ethoxy)ethyl)-2-cyanopyrimidine-5-carboxamide (**6**) 3
   5,6-Bis(bromomethyl)pyrazine-2,3-dicarbonitrile (**S1**)[1] 5
   5,6-Bis((methyl(prop-2-yn-1-yl)amino)methyl)pyrazine-2,3-dicarbonitrile (**9**) 7
   Methyl 3-cyanopyrazine-2-carboxylate (**S2**)[2] 8
   Methyl 2-((3-cyanopyrazine-2-carbonyl)thio)acetate (**11**) 10
   Diethyl 5,6-dicyanopyrazine-2,3-dicarboxylate (**10**)[3] 12
   2,3-Dicyanoquinoxaline-6-carboxylic acid (**S3**)[4] 14
   N-(3-azidopropyl)-2,3-dicyanoquinoxaline-6-carboxamide (**12**)[5] 16
   2,3-Dicyano-*N*-(prop-2-yn-1-yl)quinoxaline-6-carboxamide (**S4**) 18
   2-(Pyrimidin-2-yl)-1,3-dithiolan-2-amine - 5 membered ADTA (**2**) 20
   Spiro[pyrrolo[3,4-b]pyrazine-5,2'-[1,3]dithiolan]-7(6H)-imine (**4**) 22
   7,7-Bis(ethylthio)-6,7-dihydro-5H-pyrrolo[3,4-b]pyrazin-5-imine (**5**) 23
   5-(Trifluoromethyl)pyrimidine-2-carbonitrile (**S5**)[6] 25
   5,6-Dimethyl-3-(methylsulfonyl)-1,2,4-triazine[7] 26
   5,6-Dimethyl-1,2,4-triazine-3-carbonitrile (**S6**) 28
   2-(Pyrimidin-2-yl)-1,3-dithian-2-amine - 6 membered ADTA (**S7**) 30
   ADTA (**2**) thiol stability studies 33
      ADTA (**2**) incubation with ethanethiol 33
      ADTA (**2**) aqueous stability 35
   Ethanedithiol Vs. Propanedithiol NBT competition experiment 36
   ADTA (**2**) reversibility- *N*-methyl maleimide 37
   DFT general remarks and calculated co-ordinates (Figure S5) 40
   M062X/6-31+G(d,p) – 5-membered Acyclic optimised geometry 41
   M062X/6-31+G(d,p) – 5-membered cyclic optimised geometry 42
   M062X/6-31+G(d,p) – 6-membered Acyclic optimised geometry 43
   M062X/6-31+G(d,p) – 6-membered Cyclic optimised geometry 44
3. Bioconjugation general remarks 45
   LCMS general remarks 46
   Native Fab 47
   Reduced Fab 48
   Trastuzumab 49
   Reduced Trastuzumab 50
   CLT reaction of Fab with 2-cyanopyrimidine (Low equivalents) 51
   CLT reaction of Fab with 2-cyanopyrimidine (High equivalents) 52

CLT reaction of Fab with 2-cyanopyrimidine (Reduce and cap) 56
CLT reaction of Fab with *N*-(2-(2-(2-(2-azidoethoxy)ethoxy)ethoxy)ethyl)-2-cyanopyrimidine-5-carboxamide 57
SPAAC functionalisation of Fab conjugate **7** 59
Reaction of Fab with pyrazine-2,3-dicarbonitrile 61
Reaction of Fab with 5,6-bis((methyl(prop-2-yn-1-yl)amino)methyl)pyrazine-2,3-dicarbonitrile 62
Reaction of Fab with 5,6-bis((methyl(prop-2-yn-1-yl)amino)methyl)pyrazine-2,3-dicarbonitrile with CuAAC attempt 63
Reaction of Fab with methyl 2-((3-cyanopyrazine-2-carbonyl)thio)acetate 65
Reaction of Fab with diethyl 5,6-dicyanopyrazine-2,3-dicarboxylate 66
Removal of diethyl 5,6-dicyanopyrazine-2,3-dicarboxylate bridge 67
Reaction of Fab with *N*-(3-azidopropyl)-2,3-dicyanoquinoxaline-6-carboxamide 69
Reaction of Fab with *N*-(3-azidopropyl)-2,3-dicyanoquinoxaline-6-carboxamide and SPAAC 70
..... 71
Reaction of Fab with 2,3-dicyano-N-(prop-2-yn-1-yl)quinoxaline-6-carboxamide. 72
Reaction of Fab with 2,3-dicyano-N-(prop-2-yn-1-yl)quinoxaline-6-carboxamide with CuAAC. 73
Re-bridging of full antibody with *N*-(3-azidopropyl)-2,3-dicyanoquinoxaline-6-carboxamide 75
Re-bridging of full antibody with *N*-(3-azidopropyl)-2,3-dicyanoquinoxaline-6-carboxamide and SPAAC 76
Disulfide competition experiment (GSH Vs Fab)- NMM 78
Disulfide competition experiment (GSH Vs Fab)- 2,3-dicyanoquinoxaline-6-carboxylic acid 79
GSH stability studies 81
Blood mimicking Glutathione (GSH) concentration 81
Early endosomal mimicking Glutathione (GSH) concentration 85
CLT reaction of Fab with 5-(trifluoromethyl)pyrimidine-2-carbonitrile 89
CLT reaction of Fab with 5,6-Dimethyl-1,2,4-triazine-3-carbonitrile 90
Thioredoxin (Trx) 91
Reaction of Trx with pyrazine-2,3-dicarbonitrile 92
Serum Stability Study[10] 93
Enzyme-linked immunosorbent assay (ELISA) – Trastuzumab against HER2 94
MS/MS analysis (S46) 95
Sequence coverage of CLT conjugate S7 by trypsin digestion 98
Modification summary table 101
Fab conjugate **8** SEC trace[10] 106

NBT rate studies (S49)........ 107
References 0

1. Chemical synthesis general remarks

All chemical reagents and solvents were purchased from commercial sources; Sigma UK, Fisher UK or VWR UK and used as per manufacturer instructions. All chemical reactions were carried out at atmospheric pressure, under argon. Room temperature (RT) is defined as between 15-25 °C. The term *in vacuo* refers to organic solvent removal using Buchi rotary evaporator between 15-60 °C under reduced pressure. Chemical reactions were monitored using thin layer chromatography (TLC) on pre-coated silica gel plates (254 µm) purchased from VWR, UK. Detection of synthesised compounds was done by UV (254 nm and 365 nm) or chemical stain ($KMnO_4$, ninhydrin). Flash column chromatography was carried out using pre-loaded FlashPure Eco Flex column on Biotage Isolera Spektra One flash chromatography system. ^{1}H NMR and ^{13}C NMR were obtained at ambient temperature on Bruker Avance instruments operating at 500 MHz, 600 MHz, or 700 MHz for ^{1}H and 126 MHz, 151 MHz or 176 MHz for ^{13}C in the stated solvent. Chemical shifts (δ) are reported in parts per million (ppm) and coupling constants (*J*) in Hertz (Hz). The multiplicity of each signal is indicated as s-singlet, d-doublet, t-triplet, q-quartet, quin-quintet, m-multiplet (i.e., complex peak obtained due to overlap) or a combination of these. All assignments were made with the aid of DEPT, COSY, HSQC, HMBC, or NOESY correlation experiments. Infra-red spectra were recorded on a Bruker ALPHA FT-IR spectrometer operating in ATR mode, with frequencies given in reciprocal centimetres (cm^{-1}). Melting points were taken on a Gallenkamp apparatus and are uncorrected. High- and low-resolution mass spectra were recorded on a VG70 SE mass spectrometer, operating in modes ESI, EI, or CI (+ or -) depending on the sample, at the Department of Chemistry, University College London. Buffers used in organic reactions were prepared with double-deionised water and filter sterilised (0.20 µm).

2. Synthesis and characterisation of compounds

N-(2-(2-(2-(2-azidoethoxy)ethoxy)ethoxy)ethyl)-2-cyanopyrimidine-5-carboxamide (**6**)

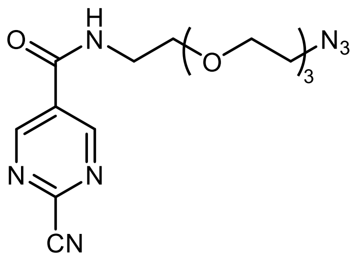

2-Cyanopyrimidine-5-carboxylic acid (80 mg, 0.54 mmol) and EEDQ (160 mg, 0.65 mmol) were dissolved in MeCN (10 mL). The resultant solution was then stirred at RT for 0.5 h. 2-(2-(2-(2-Azidoethoxy)ethoxy)ethoxy)ethan-1-amine (95.8 µL, 0.48 mmol) was added and the reaction was stirred at RT for 14 h after which the solvent was removed *in vacuo*. The crude mixture was purified by flash column chromatography (Flashpure, 4 g, eluent 0-100% EtOAc:Cyclohexane, followed by 0-10% MeOH:EtOAc) to yield the title product as a colourless oil (85 mg, 0.24 mmol, 51%).^{1}H NMR (500 MHz, $CDCl_3$) δ 9.28 (s, 2H), 3.70-3.66 (m, 12H), 3.62 (t, 2H, *J* = 5 Hz), 3.39 (t, 2H, *J* = 5 Hz). ^{13}C NMR (126 MHz, $CDCl_3$) δ 162.1, 157.3, 146.2,129.6, 115.5, 70.7 , 70.6, 70.5, 70.3, 70.0, 69.5, 50.6, 40.4. IR vmax / cm^{-1} 3345, 3067, 2954, 2159, 2117. HRMS (ES+) theoretical $[C_{14}H_{19}N_7O_4+H]^+$: 350.1566, measured: 350.1571.

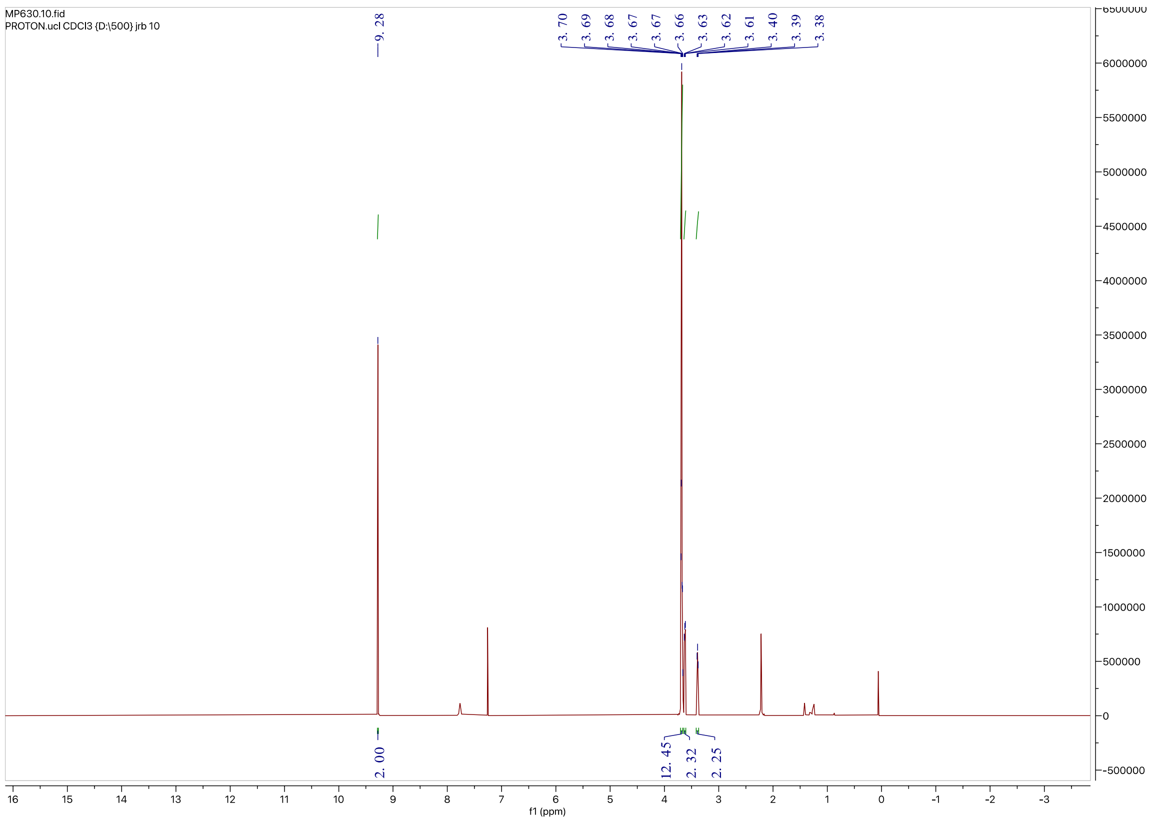

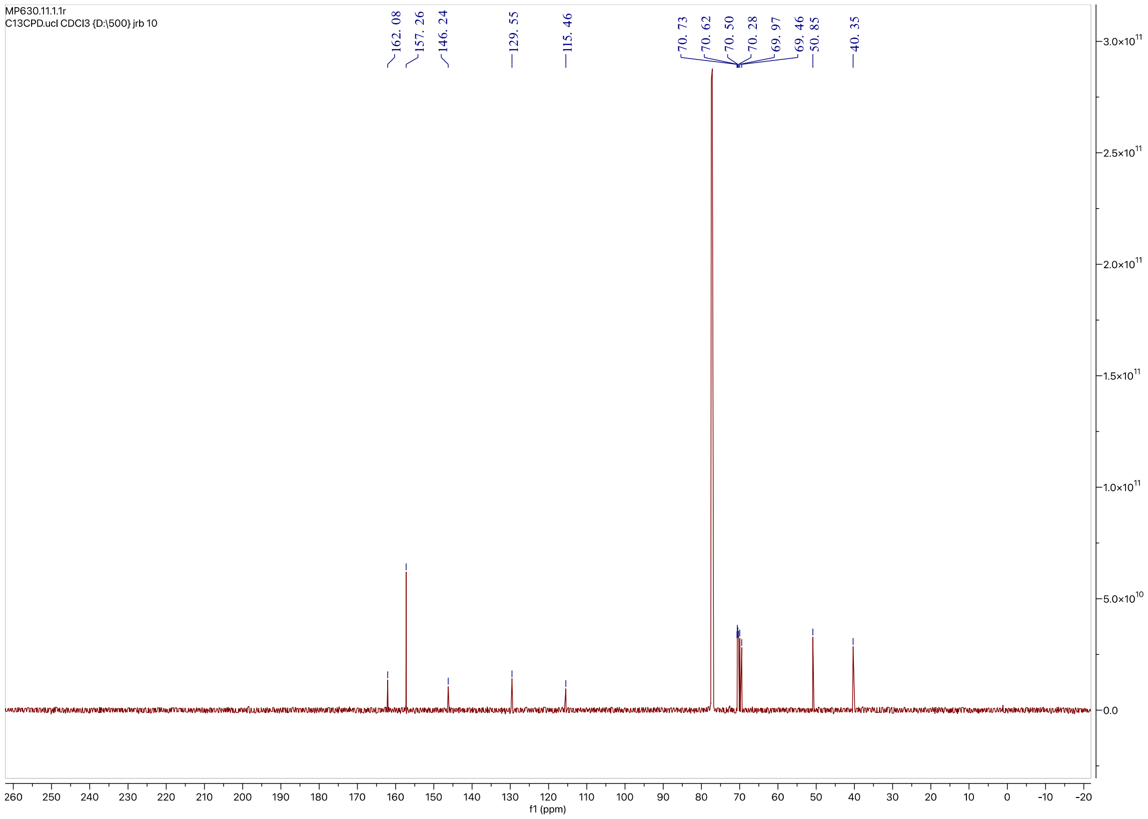

5,6-Bis(bromomethyl)pyrazine-2,3-dicarbonitrile (**S1**)[1]

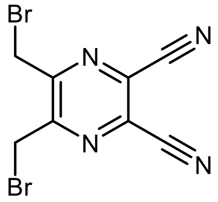

A solution of 1,4-dibromo-butane-2,3-dione (2.0 g, 8.2 mmol) in MeCN (8 mL) was added to a solution of diaminomaleonitrile (887 mg, 8.2 mmol) in MeCN (8 mL). The solution was heated at 70 °C for 1 h, the solvent was removed *in vacuo* and the resulting solid residue was purified with flash column chromatography (Flashpure, 12 g, eluent 100% CH_2Cl_2) to obtain the title product as a pale yellow solid (1.6 g, 5.0 mmol, 61 %); m.p. 136-139 °C. ^{1}H NMR (600 MHz, CD_3CN) δ 4.78 (s, 4H); ^{13}C NMR (151 MHz, CD_3CN) δ 156.1 (Ar-C), 133.2 (Ar-C), 114.2 (CN), 29.0 (CH_2). IR vmax / cm^{-1} 3037, 2978, 2244. HRMS (ES+) theoretical $[C_8H_4Br_2{}^{79}N_4+H]^+$: 314.8875, measured: 314.8872. HRMS (ES+) theoretical $[C_8H_4Br_2{}^{81}N_4+H]+$: 316.8875, measured: 316.8851.

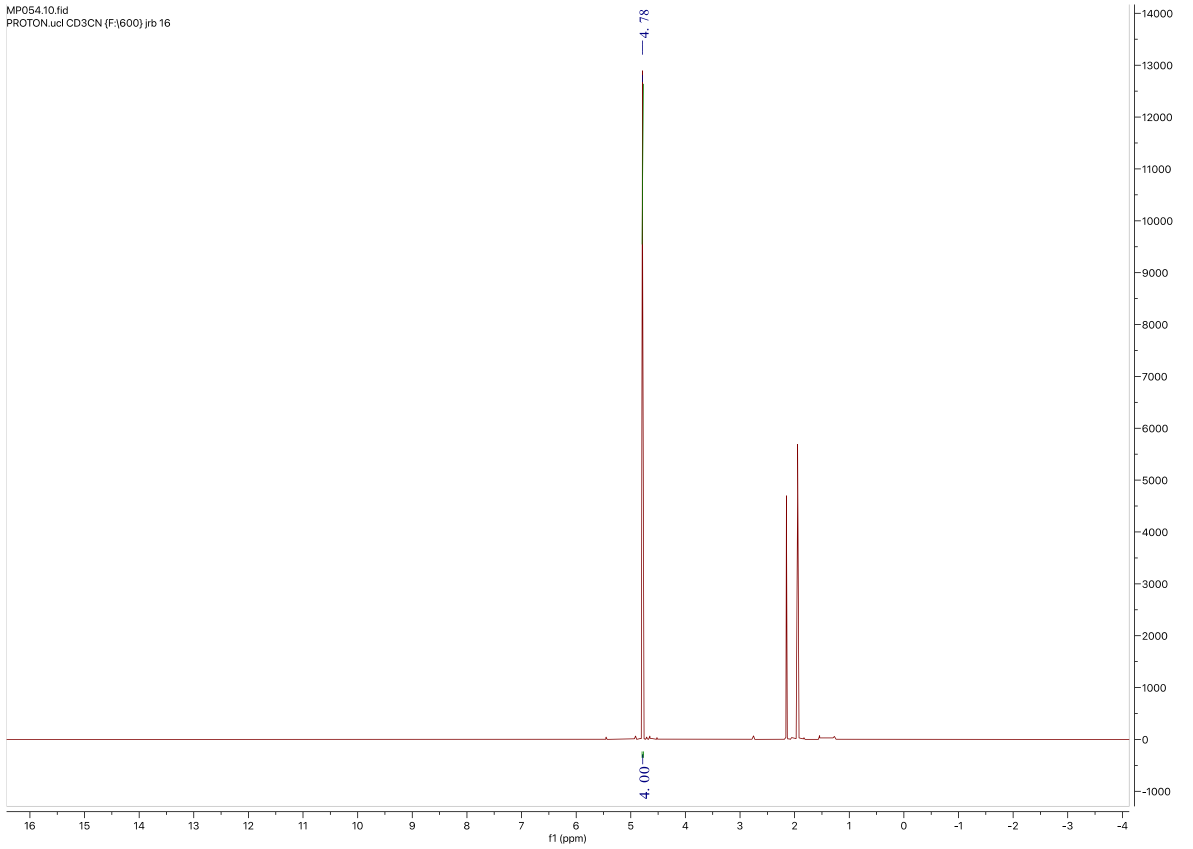
MP054.10.fid
PROTON.ucl CD3CN {F:\600} jrb 16
4.78
4.00
f1 (ppm)

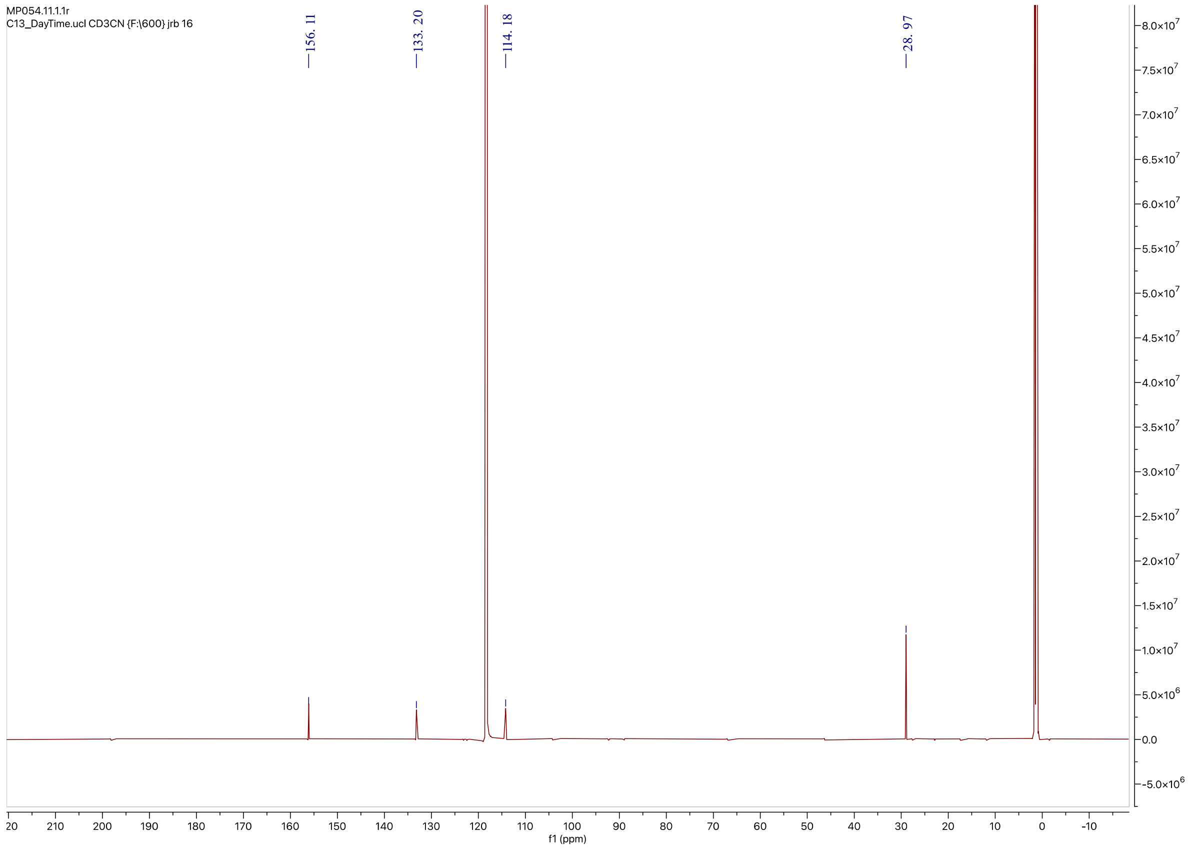
MP054.11.1.1r
C13_DayTime.ucl CD3CN {F:\600} jrb 16
156.11
133.20
114.18
28.97
f1 (ppm)

5,6-Bis((methyl(prop-2-yn-1-yl)amino)methyl)pyrazine-2,3-dicarbonitrile (**9**)

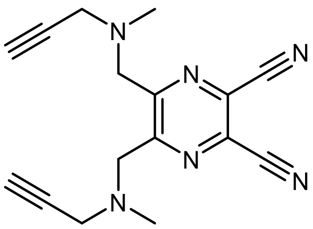

A solution of 5,6-bis(bromomethyl)pyrazine-2,3-dicarbonitrile (150 mg, 0.47 mmol) in acetone (5 mL) was added to a solution of *N*-methyl propargylamine (87.0 µL, 1.04 mmol) and pyridine (83.8 µL, 1.04 mmol) in acetone (5 ml) at RT. The reaction was stirred for 45 min after which the reaction was concentrated *in vacuo* and diluted with CH_2Cl_2 (8 mL). The organic layer was then washed with aq. $CuSO_4$ (3 x 5 mL), water (5 mL) and dried over $MgSO_4$. The solution was then concentrated *in vacuo* and purified using flash column chromatography (Flashpure, 4 g, eluent 0-35% EtOAc:Cyclohexane) to obtain the title product as a yellow oil (21 mg, 0.072 mmol, 16%). ^{1}H NMR (600 MHz, $CDCl_3$) δ 3.40 (d, 4H, J = 2.2 Hz), 2.34 (s, 6H), 2.30 (t, 2H, J = 2.4 Hz); ^{13}C NMR (151 MHz, $CDCl_3$) δ 158.4 (Ar-C), 131.1 (Ar-C), 113.1 (CN), 77.8 (C), 74.4 (C), 58.4 (CH_2), 46.4 (CH_2), 42.4 (CH_3). IR vmax / cm^{-1} 3288, 3012, 2950, 2231, 2107; HRMS (ES+) theoretical $[C_{16}H_{16}N_6+H]^+$: 293.1509, measured: 293.1509.

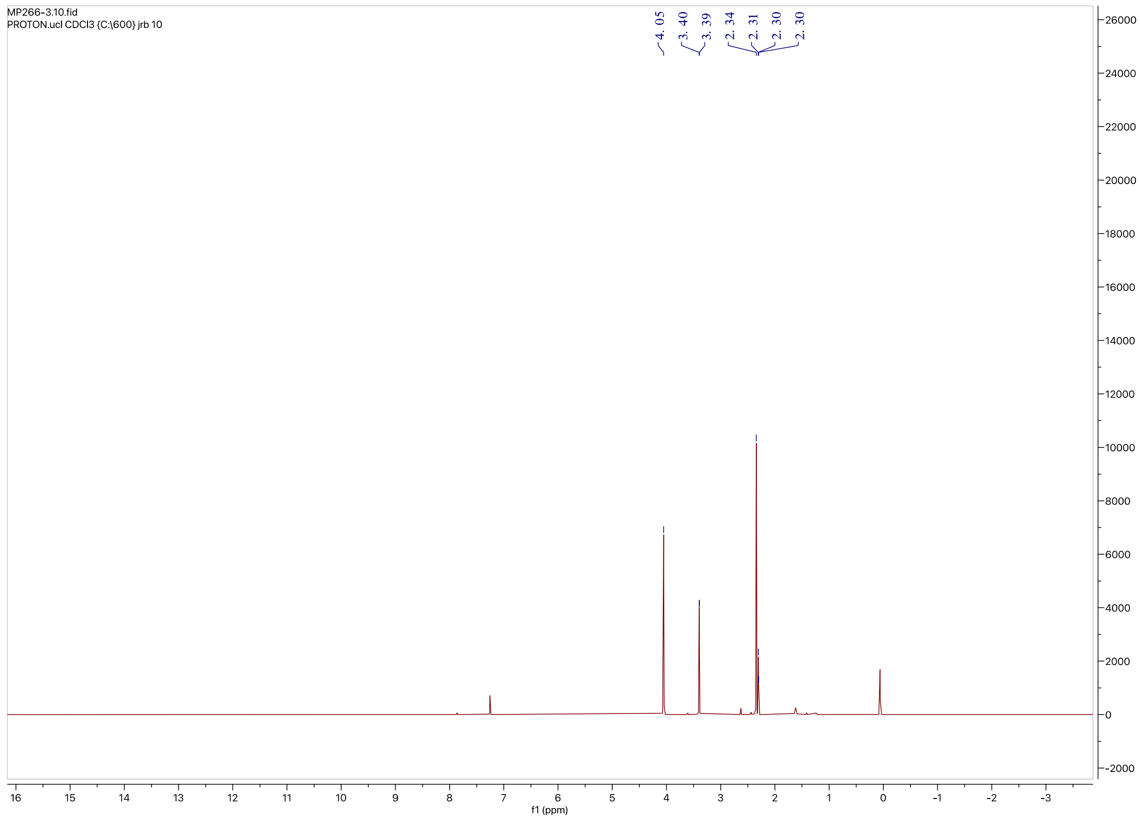

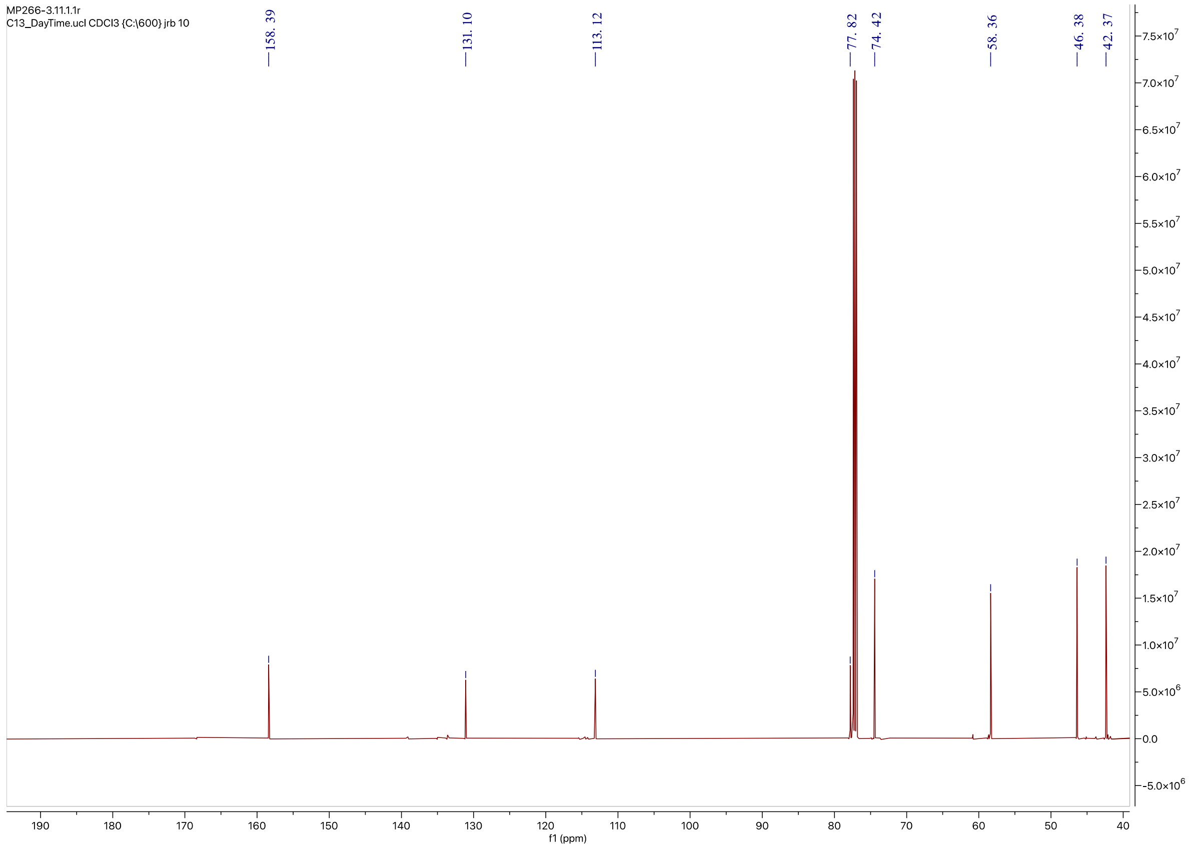

Methyl 3-cyanopyrazine-2-carboxylate (**S2**)[2]

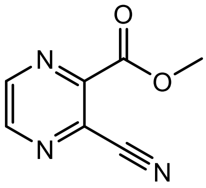

To a stirred suspension of pyrazine-2,3-dicarboxylic acid monoamide (200 mg, 1.20 mmol) in CH_2Cl_2 (2 mL) at 0 °C were added NEt_3 (0.35 mL, 2.51 mmol) and methyl chloroformate (2.03 mL, 2.63 mmol), and the mixture was allowed to reach room temperature and stirred for 2 h. Water and $NaHCO_3$ (sat.) (volume ratio = 1:1) were added (2 mL), and the mixture was extracted with CH_2Cl_2 (3 x 2 mL). The organic layer was washed with H_2O, dried over $MgSO_4$, and concentrated *in vacuo*. The crude product was purified using flash column chromatography (Flashpure, 12 g, eluent 0-50% EtOAc:Cyclohexane) to obtain the title product as a white solid (98 mg, 0.60 mmol, 50%); m.p. 157-158 °C. ^{1}H NMR (600 MHz, CD_3CN) δ 8.92 (d, 1H, *J* = 2.3 Hz), 8.90 (d, 1H, *J* = 2.3 Hz), 4.02 (s, 3H); ^{13}C NMR (151 MHz, CD_3CN) δ 163.7 (Ar-C), 148.5 (Ar-C), 147.8 (Ar-C), 147.0 (Ar-C), 131.4 (Ar-C), 116.2 (CN), 54.1 (CH_3). IR vmax / cm^{-1} 3033, 2956, 2060, 1731; HRMS (ES+) theoretical $[C_7H_5N_3O_2+H]^+$: 164.0455, measured : 164.0455.

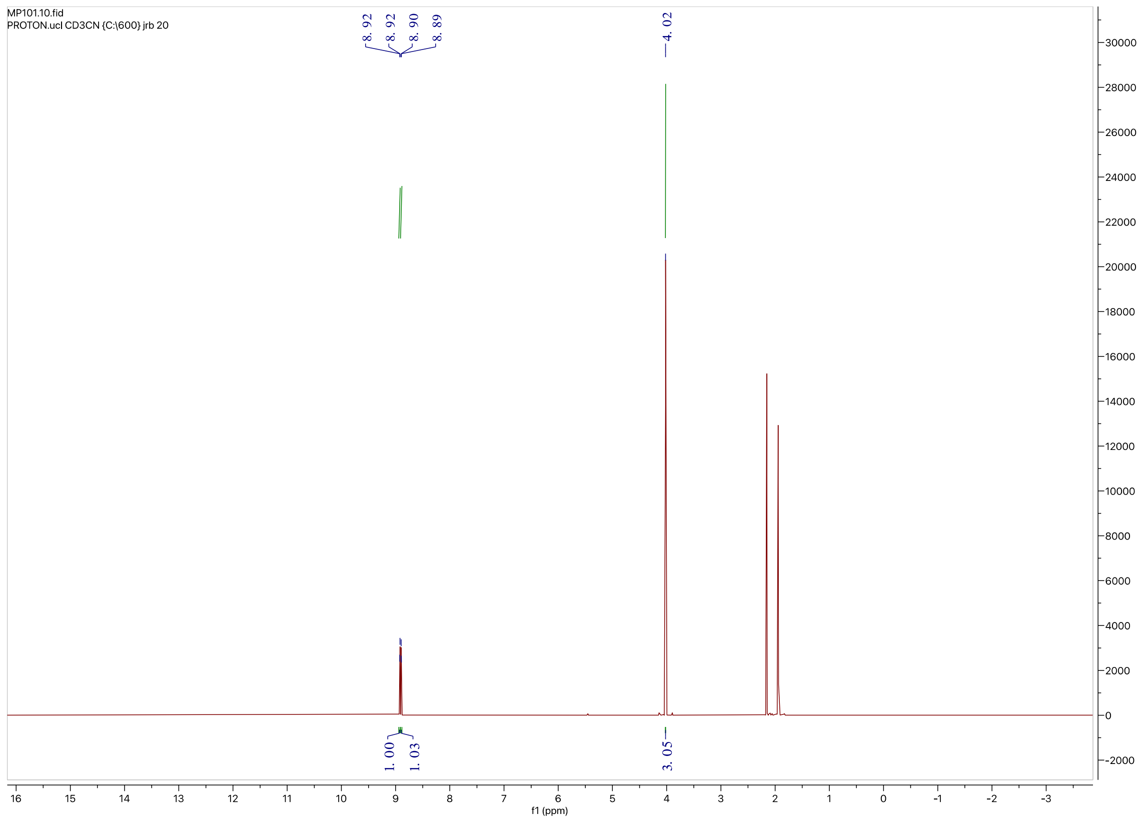
MP101.10.fid
PROTON.ucl CD3CN {C:\600} jrb 20
8.92
8.92
8.90
8.89
4.02
1.00
1.03
3.05
f1 (ppm)

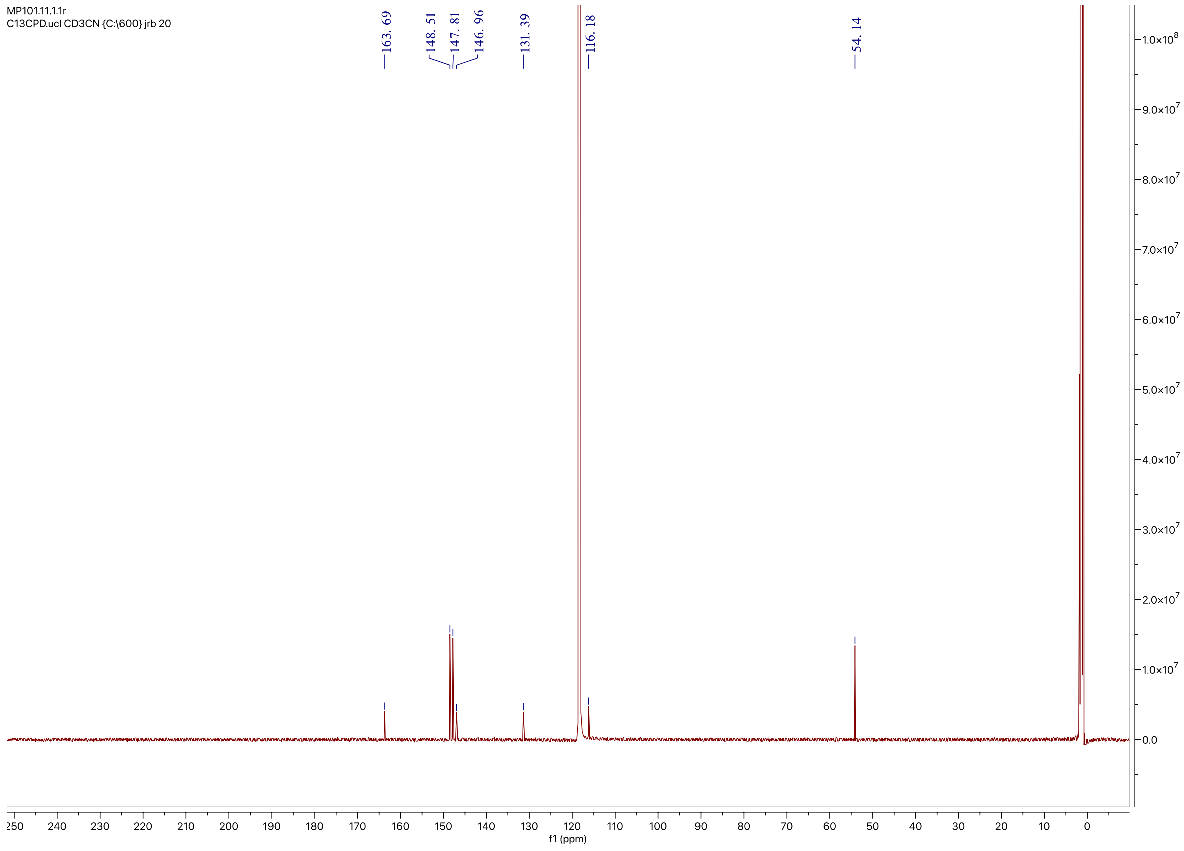
MP101.11.1.1r
C13CPD.ucl CD3CN {C:\600} jrb 20
163.69
148.51
147.81
146.96
131.39
116.18
54.14
f1 (ppm)

Methyl 2-((3-cyanopyrazine-2-carbonyl)thio)acetate (**11**)

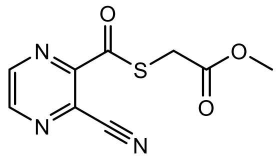

To a solution of pyrazine-2,3-dicarboxylic acid monoamide (96.1 mg, 0.58 mmol) in THF (10 mL) was added DMAP (7.1 mg, 0.058 mmol) followed by EDC.HCl (220 mg, 1.15 mmol) and methyl thioglycolate (0.155 mL, 1.73 mmol). The reaction was stirred for 16 h at RT after which the mixture was concentrated *in vacuo*. To a solution of crude methyl 2-((3-carbamoylpyrazine-2-carbonyl)thio)acetate (31 mg, 0.12 mmol) in CH_2Cl_2 (5 mL) at 0 °C were added NEt_3 (35 µL, 0.25 mmol) and methyl chloroformate (20 µL, 0.263 mmol), and the mixture was allowed to reach room temperature and stirred for 2 hrs. Water and $NaHCO_3$ (sat.) (volume ratio = 1:1) were added (3 mL), and the mixture was extracted with CH_2Cl_2 (3 x 3 mL). The organic layer was washed with H_2O and dried over $MgSO_4$. The crude product was purified using flash column chromatography (Flashpure, 4 g, eluent 0-65% EtOAc:Cyclohexane) to obtain the title product as a yellow oil (13.6 mg, 0.057 mmol, 48%). ^{1}H NMR (600 MHz, $CDCl_3$) δ 8.95 (d, 1H, *J* = 2.3 Hz), 8.88 (d, 1H, *J* = 2.3 Hz), 3.92 (s, 2H), 3.78 (s, 3H); ^{13}C NMR (151 MHz, $CDCl_3$) δ 189.0 (C=O), 168.6 (C=O), 148.5 (Ar-C), 147.4 (Ar-C), 145.8 (Ar-C), 126.7 (Ar-C), 114.4 (CN), 53.2 (CH_3), 31.6 (CH_2). IR vmax / cm^{-1} 3015, 2955, 2233, 1737,1675; HRMS (ES+) theoretical $[C_9H_7N_3O_3S+H]^+$: 238.0281, measured : 238.0279.

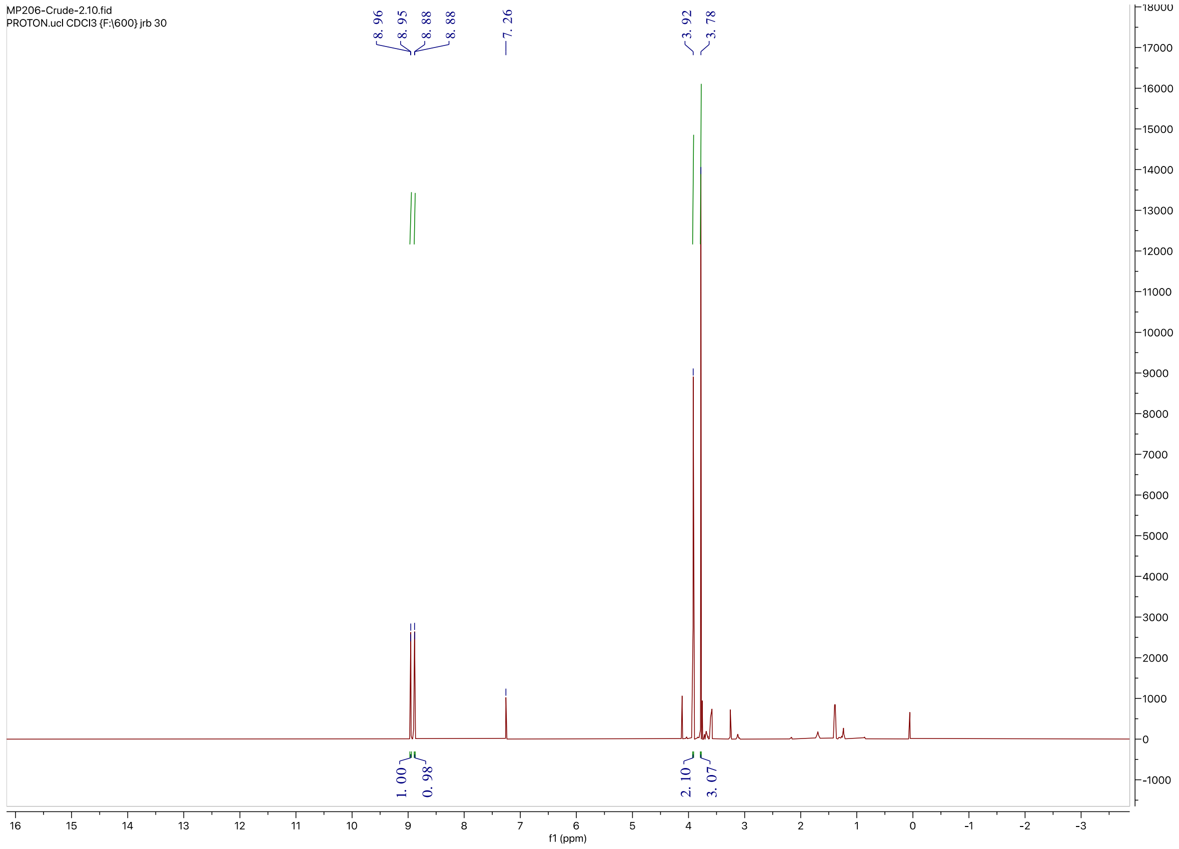
MP206-Crude-2.10.fid
PROTON.ucl CDCl3 {F:\600} jrb 30
8.96
8.95
8.88
8.88
7.26
3.92
3.78
1.00
0.98
2.10
3.07
f1 (ppm)

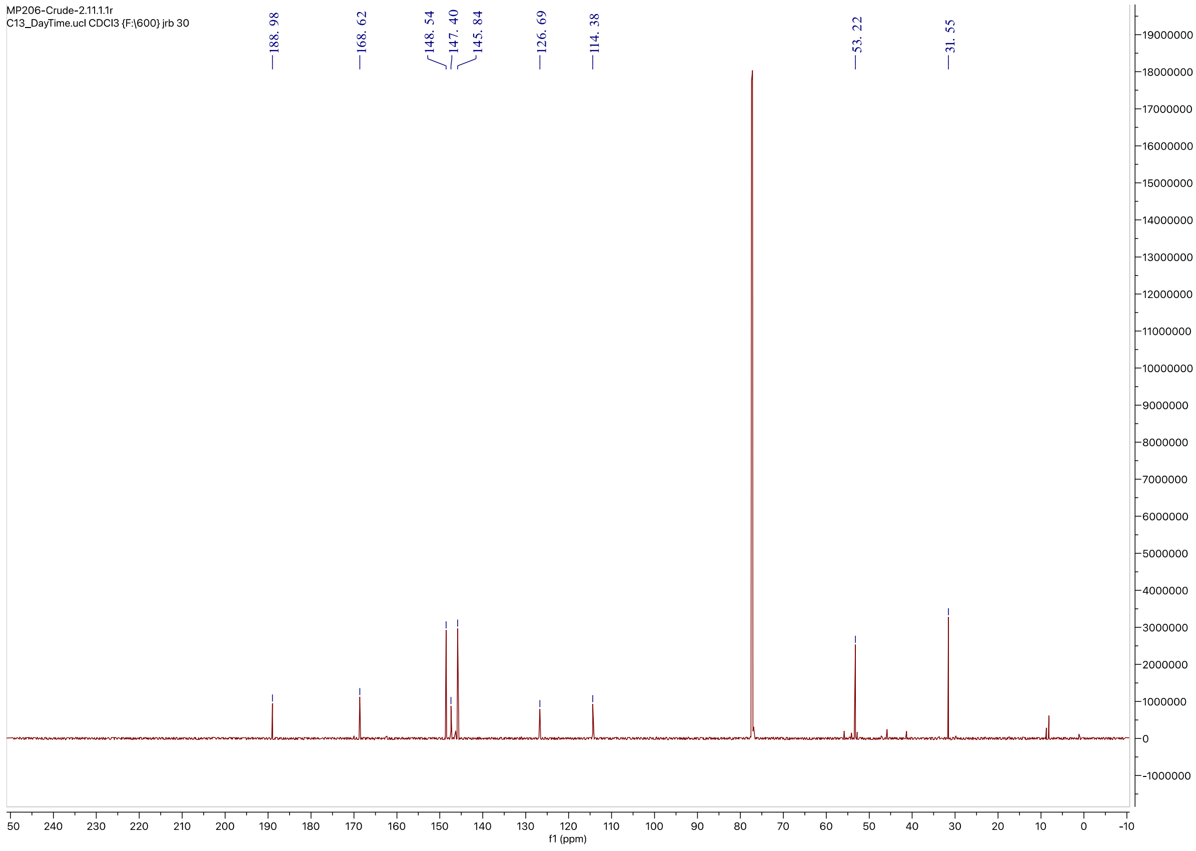
MP206-Crude-2.11.1.1r
C13_DayTime.ucl CDCl3 {F:\600} jrb 30
188.98
168.62
148.54
147.40
145.84
126.69
114.38
53.22
31.55
f1 (ppm)

Diethyl 5,6-dicyanopyrazine-2,3-dicarboxylate (**10**)[3]

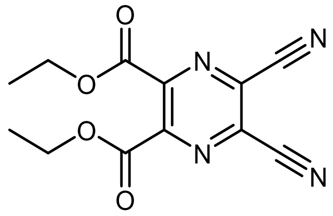

A mixture of diethyl tartrate (1.03 g, 5.0 mmol) and NBS (3.56 g, 20 mmol) in dry $C_2H_4Cl_2$ (7 mL) was heated under reflux for 12 h under N_2 atmosphere. After completion, Na_2SO_3 was added to quench the residual bromine. The reaction mixture was evaporated under reduced pressure to remove $C_2H_4Cl_2$. Then the residue was diluted with diethyl ether (20 mL), filtered, and the solid washed with ether (10 mL). The combined filtrate was evaporated under reduced pressure to yield a yellow oil - crude diethyl 2,3-dioxosuccinate (677 mg, approximately 3.35 mmol). To a solution of crude diethyl 2,3-dioxosuccinate in AcOH (3.35 mL) and EtOH (80 mL) was added DAMN (362 mg, 3.35 mmol). The reaction was stirred at 80 °C for 2 h after which the reaction was concentrated under reduced pressure. The crude product was purified with flash column chromatography (Flashpure, 12 g, eluent 100% CH_2Cl_2) to yield the title product as a white solid (414 mg, 1.51 mmol, 45%); m.p. 81-85 °C. ^{1}H NMR (600 MHz, $CDCl_3$) δ 4.48 (q, 4H, *J* = 7.2 Hz), 1.41 (t, 6H, *J* = 7.2 Hz). ^{13}C NMR (151 MHz, $CDCl_3$) δ 161.6 (C=O), 146.5 (Ar-C), 133.5 (Ar-C), 112.0 (CN), 64.3 (CH_2), 27.0 (CH_3). IR vmax / cm^{-1} 3003, 2940, 2247, 1724. HRMS (ES+) Theoretical $[C_{12}H_{10}N_4O_4+H]^+$: 275.0775 Measured : 275.0735.
EtOAc present in ^{1}H and ^{13}C NMRs.

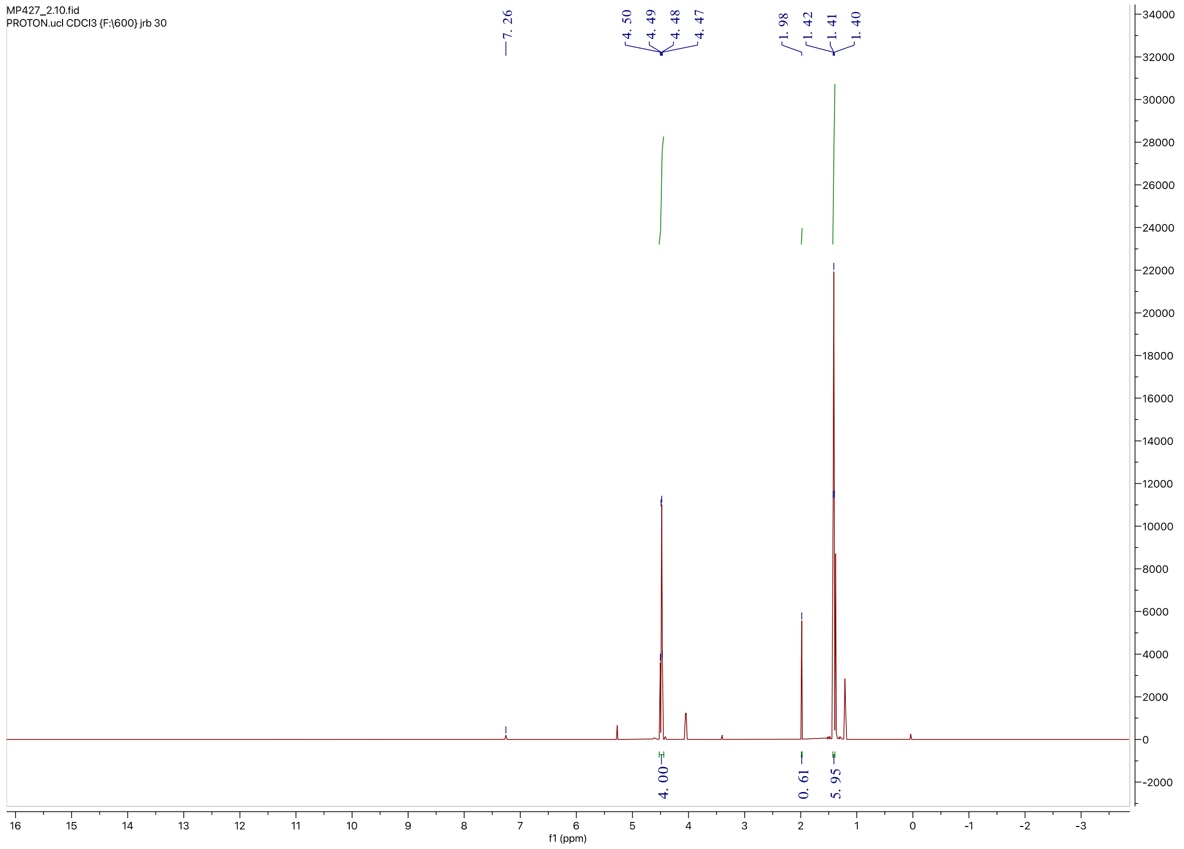
MP427_2.10.fid
PROTON.ucl CDCl3 {F:\600} jrb 30
7.26
4.50
4.49
4.48
4.47
1.98
1.42
1.41
1.40
4.00
0.61
5.95
f1 (ppm)

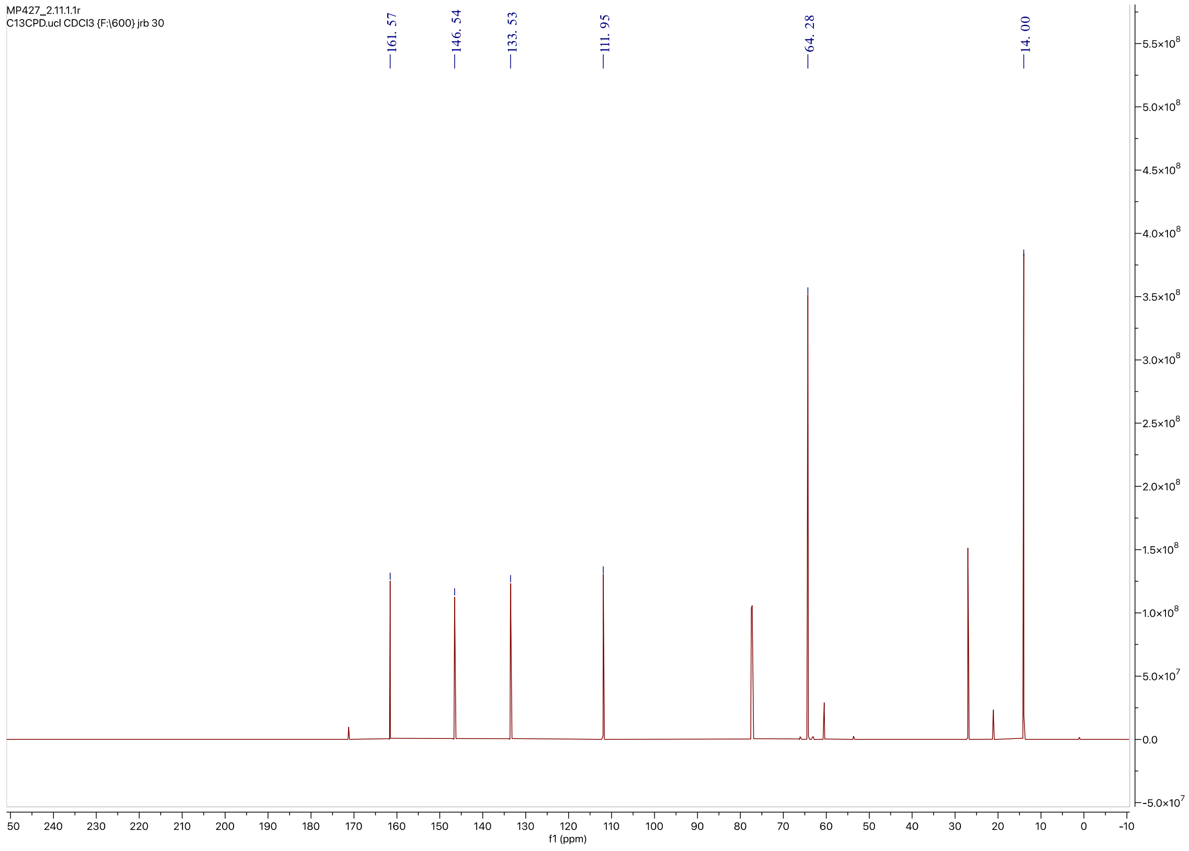
MP427_2.11.1.1r
C13CPD.ucl CDCl3 {F:\600} jrb 30
161.57
146.54
133.53
111.95
64.28
14.00
f1 (ppm)

2,3-Dicyanoquinoxaline-6-carboxylic acid (**S3**)[4]

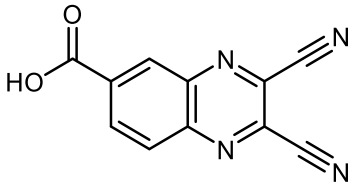

DAMN (540 mg, 5 mmol) was dissolved in MeCN (25 mL), to this solution was added DDQ (1.14 g, 5 mmol). The reaction was stirred for 20 min at RT after which the mixture was filtered and concentrated *in vacuo* to yield DISN (the crude intermediate was carried forward). DISN (531 mg, 5 mmol) was dissolved in TFA (8 mL), to this mixture was added 1,2-diaminobenzoic acid (767mg, approximately 5 mmol) portion wise. The reaction was then stirred overnight at RT under N_2 atmosphere. The solvent was evaporated *in vacuo*, the residue was then purified *via* flash column chromatography (Flashpure, 12 g, eluent 0-30% CH_2Cl_2:EtOAc and 1% AcOH) to yield the title product as a yellow solid (538 mg, 2.4 mmol, 48%); m.p. 217-220 °C; ^{1}H NMR (700 MHz, MeOD) δ 8.85 (s, 1H), 8.64 (d, 1H, *J* = 8.8 Hz), 8.35 (d, 1H, *J* = 8.8 Hz). ^{13}C NMR (176 MHz, MeOD) δ 167.0 (C=O), 144.3 (Ar-C), 142.3 (Ar-C), 137.6 (Ar-C), 135.1 (Ar-C), 133.8 (Ar-C), 133.2 (Ar-C), 132.7 (Ar-C), 131.21 (Ar-C), 114.78 (CN), 114.75 (CN). IR vmax / cm^{-1} 3067, 2940, 2253,1692. HRMS theoretical $[C_{11}H_4N_4O_2\text{-}H]^-$: 223.0262, measured: 223.0258.

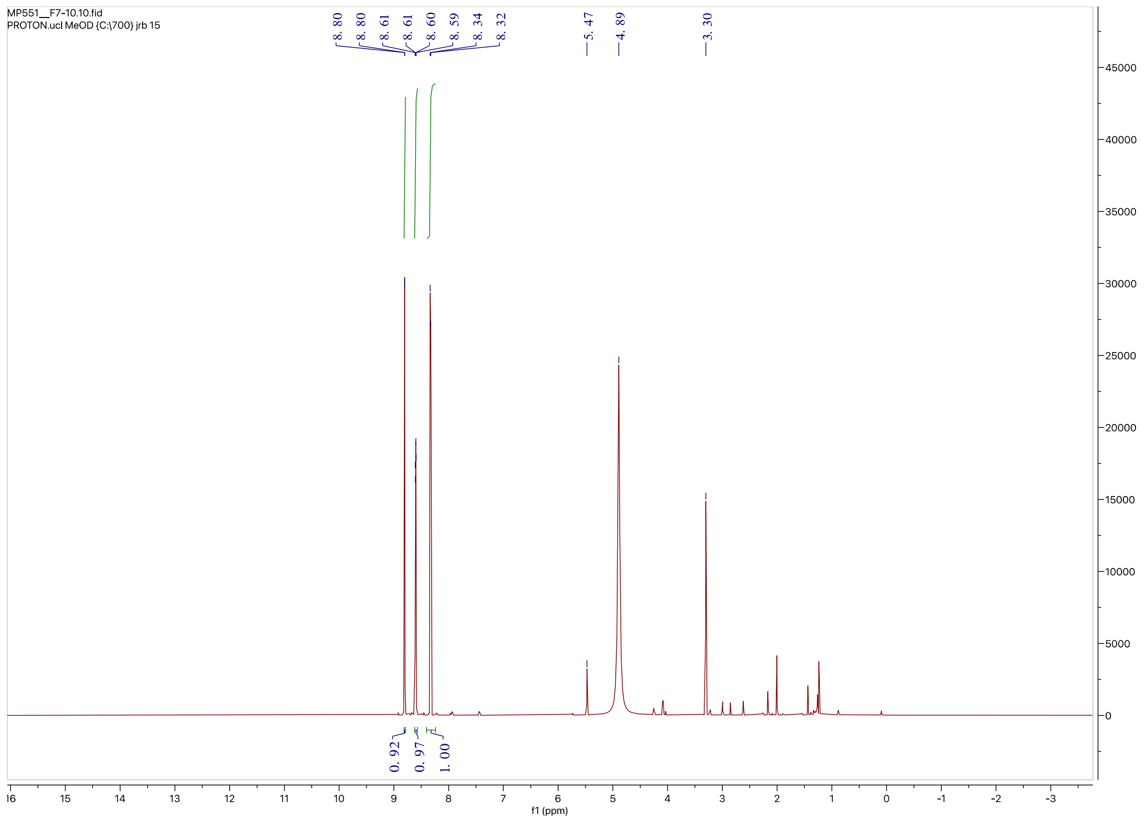
MP551__F7-10.10.fid
PROTON.ucl MeOD {C:\700} jrb 15
8.80
8.80
8.61
8.61
8.60
8.59
8.34
8.32
5.47
4.89
3.30
0.92
0.97
1.00
f1 (ppm)

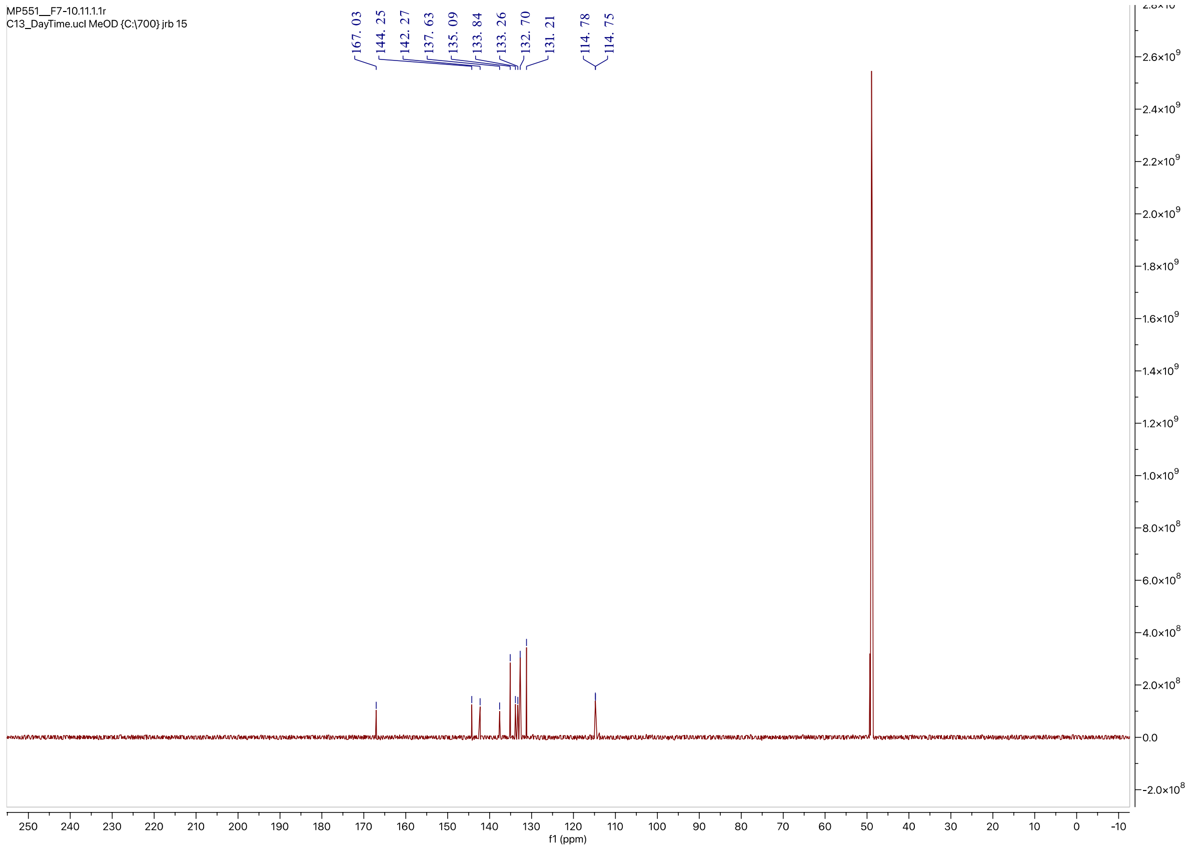
MP551__F7-10.11.1.1r
C13_DayTime.ucl MeOD {C:\700} jrb 15
167.03
144.25
142.27
137.63
135.09
133.84
133.26
132.70
131.21
114.78
114.75
f1 (ppm)

N-(3-azidopropyl)-2,3-dicyanoquinoxaline-6-carboxamide (**12**)[5]

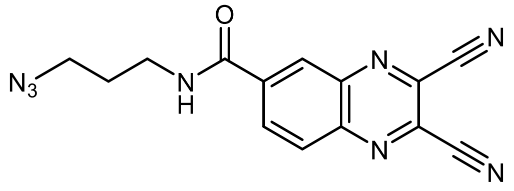

NaN_3 (450 mg, 6.9 mmol) in water (2 mL) was added to water solution (3 mL) of 3-bromopropylamine hydrobromide (500 mg, 2.3 mmol) and heated at 90 °C for 24 h. The solution was then alkalized with potassium hydroxide (approximately pH 10) and extracted with diethyl ether (3 X 5 mL), dried over anhydrous $MgSO_4$ and concentrated *in vacuo* to yield a yellow oil (174 mg, 55 %). The product was used directly in the next step due to its short shelf life.

2,3-Dicyanoquinoxaline-6-carboxylic acid (100 mg, 0.44 mmol) was dissolved in anhydrous THF (10 mL), thionyl chloride (325 µL, 0.45 mmol) in anhydrous toluene (7 mL) was added to this solution and mixture was refluxed for 3 h at 100 °C. After this time, the excess thionyl chloride and solvents were removed *in vacuo* and the resulting residue was dissolved in anhydrous THF (2 mL). 3-Azidopropan-1-amine (120 mg, 1.2 mmol) in anhydrous THF (0.5 mL) was then slowly dropped into the mixture which was then refluxed for a further 3 h at 60 °C. The crude mixture was concentrated *in vacuo* and extracted from water (5 mL) with EtOAc (3 X 5 mL). The organic layer was then washed with brine, dried over $MgSO_4$ and purified by flash column chromatography (Flashpure, 4 g, eluent 0-20% CH_2Cl_2:EtOAc) to yield the title product as a white solid (78 mg, 0.25 mmol, 58%). m.p.139-142 °C . ^{1}H NMR (700 MHz, $CDCl_3$) δ 8.55 (s, 1H), 8.52 (d, 1H, *J* = 8.8 Hz), 8.35 (d, 2H, *J* = 8.8 Hz), 3.67 (q, 3H, *J* = 6.3 Hz), 3.54 (t, 3H, *J* = 6.3 Hz), 1.99 (p, 3H, *J* = 6.5 Hz). ^{13}C NMR (176 MHz, $CDCl_3$) δ 164.86 (C=O), 142.70 (Ar-C), 141.40 (Ar-C), 140.54 (Ar-C), 133.83 (Ar-C), 131.82 (Ar-C), 131.57 (Ar-C), 130.80 (Ar-C), 128.01 (Ar-C), 113.23 (CN), 50.03 (CH_2), 38.87 (CH_2), 28.62 (CH_2). EtOAc, CH_2Cl_2 present in ^{13}C NMR. IR vmax / cm^{-1} 3083, 2955, 2110, 1737. HRMS Theoretical $[C_{14}H_{10}N_8O\text{-}H]^-$: 305.0905 Measured : 305.0905.

EtOAc, CH_2Cl_2 present in ^{13}C NMR, additional ^{1}H NMR provided in MeOD for clarity on purity.

^1H NMR in MeOD

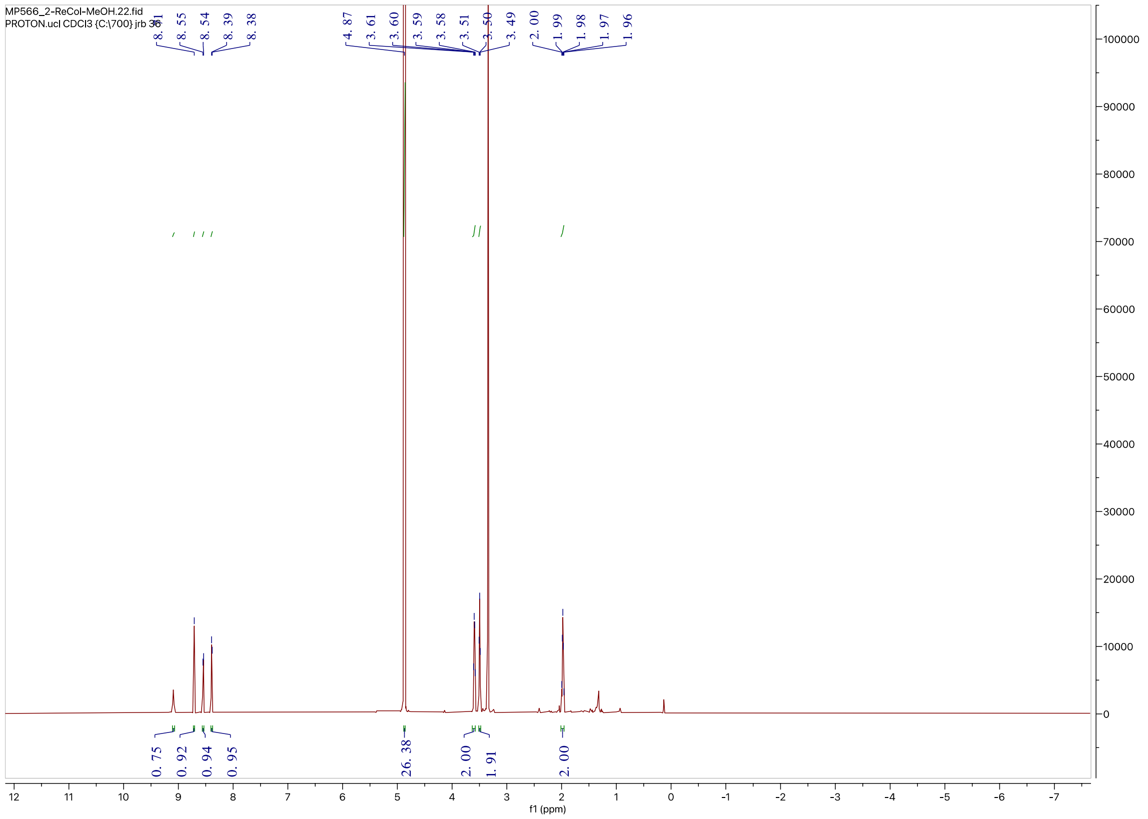

^1H NMR in $CDCl_3$

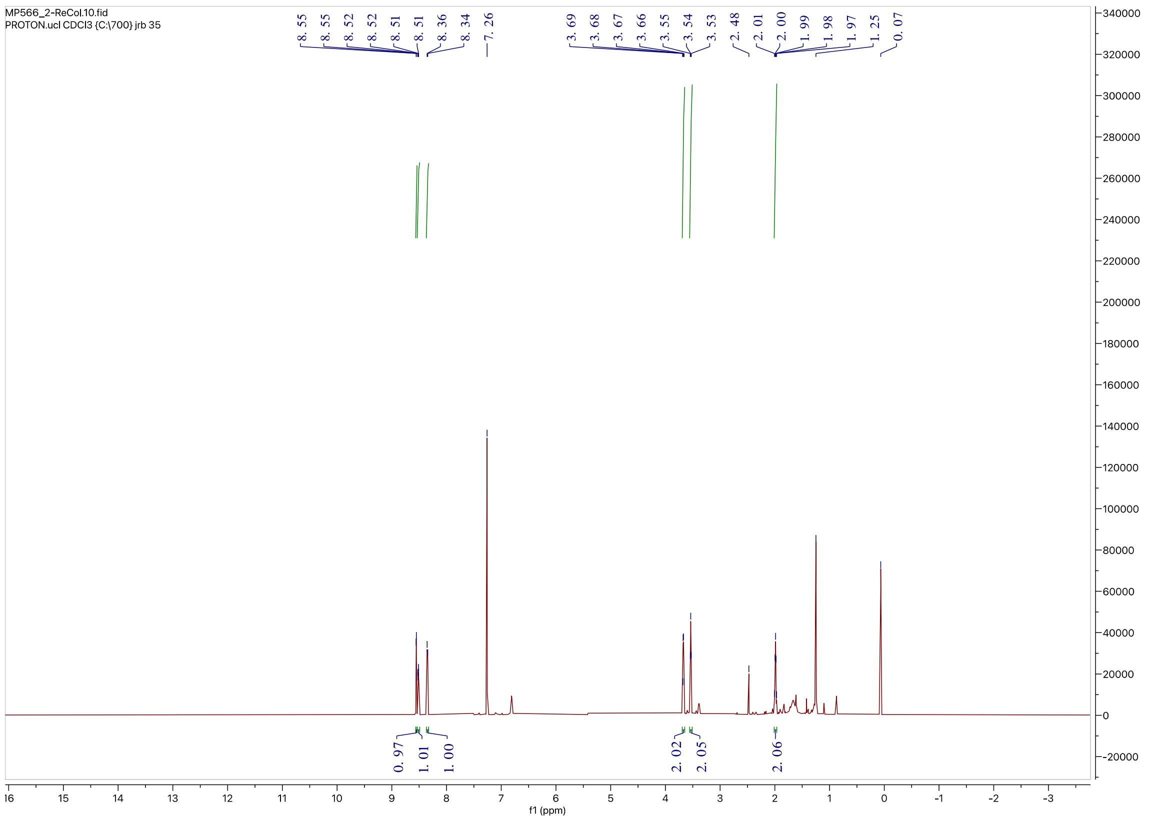

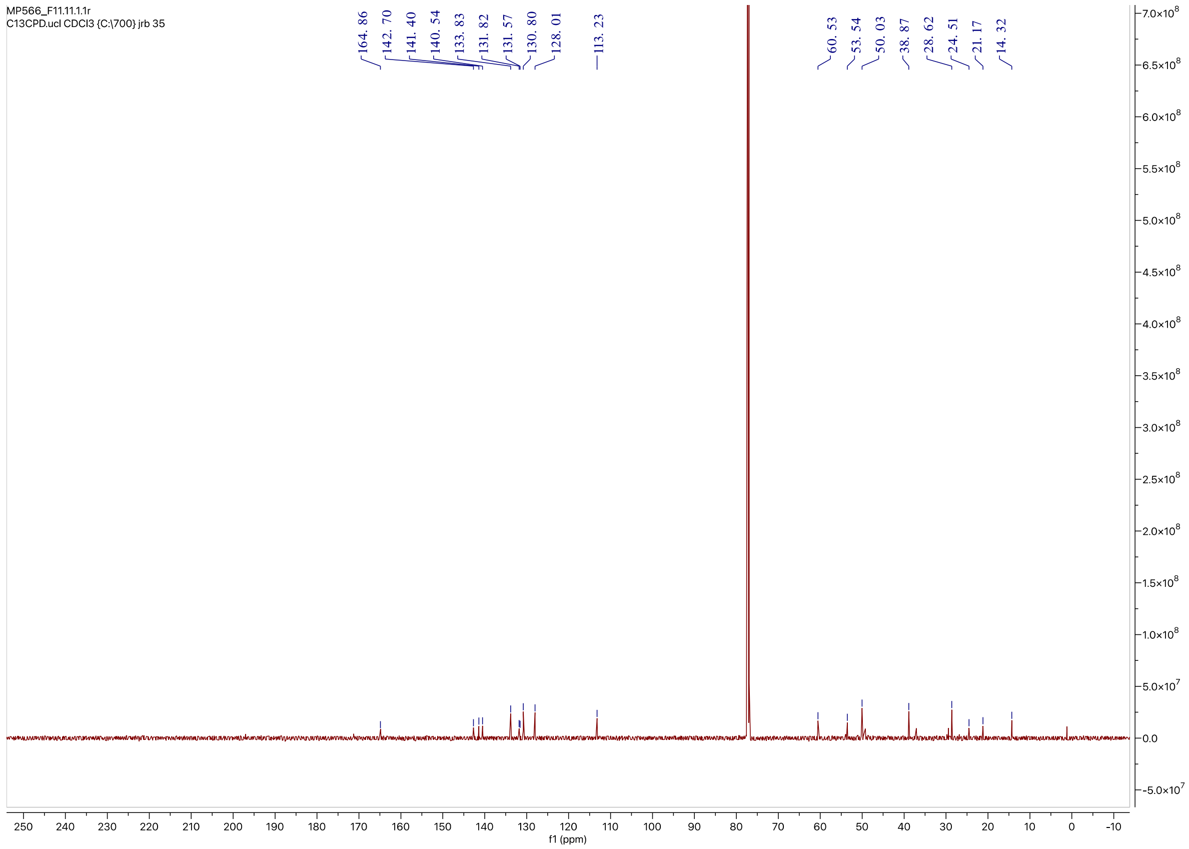

2,3-Dicyano-*N*-(prop-2-yn-1-yl)quinoxaline-6-carboxamide (**S4**)

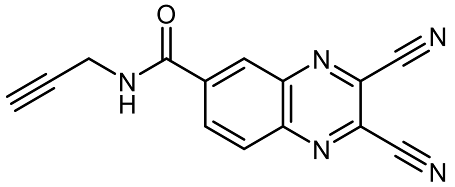

2,3-Dicyanoquinoxaline-6-carboxylic acid (110 mg, 0.49 mmol) was dissolved in a mixture of anhydrous THF (2.5 mL) and toluene (12.5 mL). To this solution, thionyl chloride (370 µL, 0.45 mmol) was added and mixture was refluxed for 3 h at 100 °C. After this time the excess thionyl chloride and solvents were removed under reduced pressure and the resulting residue was dissolved in anhydrous THF (2 mL), propargylamine (311 µL, 4.9 mmol) in anhydrous THF (0.5 mL) was then slowly dropped into the mixture which was then refluxed for a further 3 h at 60 °C. The crude mixture was concentrated *in vacuo* and extracted from water (5 mL) with EtOAc (3 X 5 mL). The organic layer was then washed with brine, dried over $MgSO_4$ and purified by flash column chromatography (Flashpure, 4 g, eluent 0-50% EtOAc:Cyclohexane) to yield **S4** as an off-white gum (57.5 mg, 0.22 mmol). NMR showed product contained minor CH_2Cl_2 and EtOAc solvent impurities, from which a final yield of 23% is calculated. ^{1}H NMR

(700 MHz, MeOD) δ 8.68 (s, 1H), 8.51 (d, 1H, *J* = 8.5 Hz), 8.35 (d, 1H, *J* = 8.8 Hz), 4.24 (d, 2H, *J* = 2.5 Hz), 2.66 (t, 1H, *J* = 2.5 Hz). CH_2Cl_2, EtOAc solvent peaks in ^1H NMR; ^{13}C NMR (176 MHz, MeOD) δ 166.9 (C=O), 143.7 (Ar-C), 142.4 (Ar-C), 140.7 (Ar-C), 134.0 (Ar-C), 133.6 (Ar-C), 133.3 (Ar-C), 131.3 (Ar-C), 129.9 (Ar-C), 114.81 (CN), 114.79 (CN), 80.1 (C), 72.4 (C), 30.2 (CH_2). IR vmax / cm^{-1} 3286, 3015, 2955, 2242, 2121, 1704. HRMS theoretical $[C_{14}H_7N_5O\text{-}H]^-$: 260.0572, measured : 260.0578.

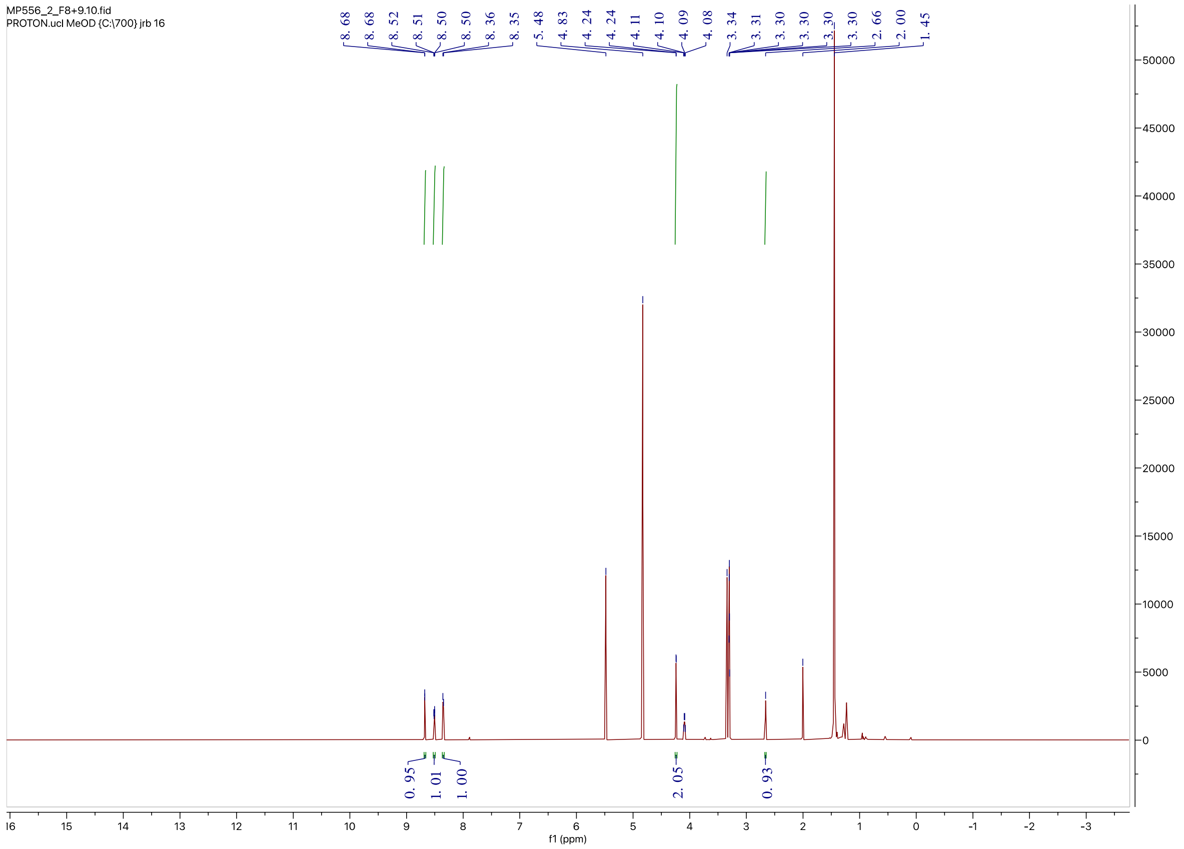

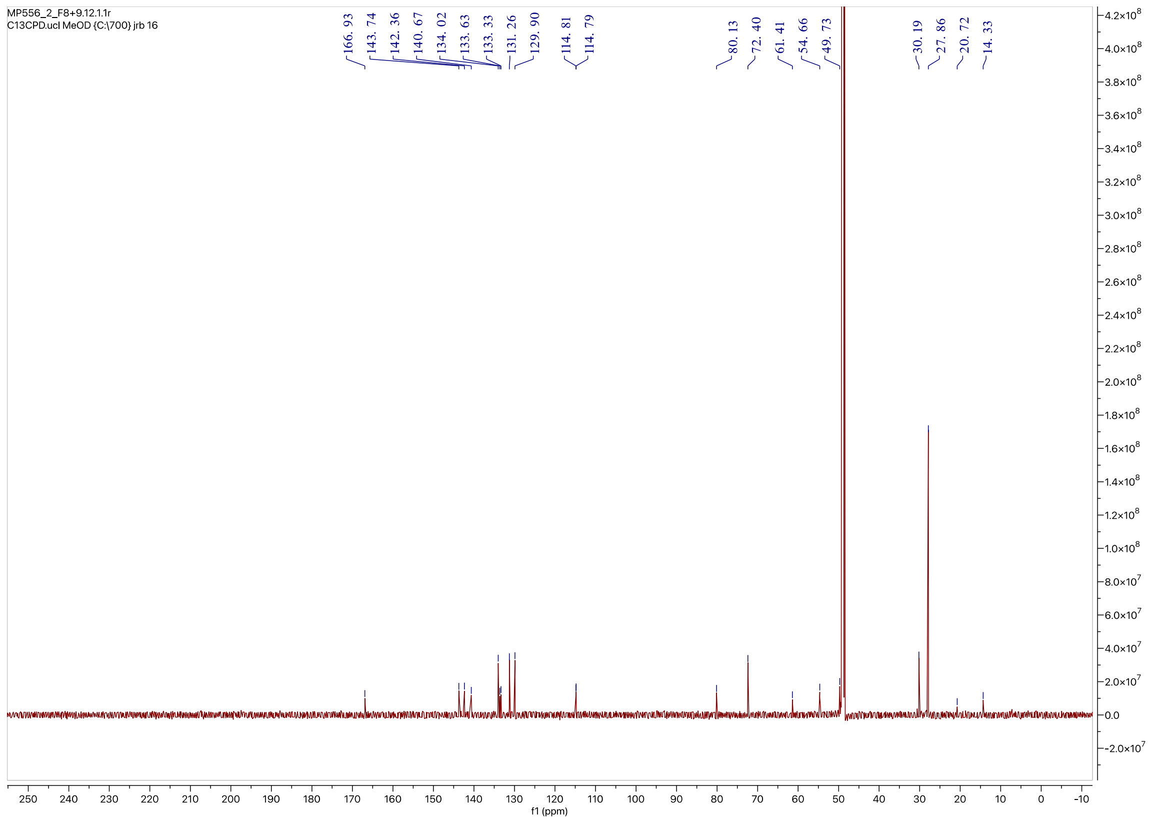

2-(Pyrimidin-2-yl)-1,3-dithiolan-2-amine - 5 membered ADTA (**2**)

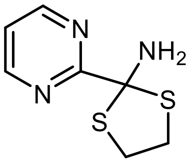

2-Cyano pyrimidine (105 mg, 1 mmol) was dissolved in MeCN (4 mL) followed by addition of 50 mM Phosphate Buffer pH 7.4 (16 mL). To this mixture was added ethanedithiol (109 µL, 1.2 mmol) was then added and stirred at room temperature for 60 min. The reaction was then extracted with EtOAc (3 X 10 mL). The organic layer was dried over $MgSO_4$ and purified by flash column chromatography (Flashpure, 4 g, eluent 0-10% MeOH: CH_2Cl_2) to afford the title product as a yellow-brown oil (181 mg, 0.91 mmol, 91%). ^{1}H NMR (700 MHz, $CDCl_3$) δ 8.71 (d, 2H, J = 4.8 Hz), 7.15 (t, 1H, J = 4.8 Hz), 3.61 (s, 4H). ^{13}C NMR (176 MHz, $CDCl_3$) δ 171.5 (Ar-C), 157.3 (Ar-C), 119.4 (Ar-C), 88.5 (C), 41.6 (CH_2). HRMS theoretical $[C_7H_9N_3S_2\text{-}NH_2]^+$: 183.0051, measured: 183.0046.

Title product presents an unpleasant smell.

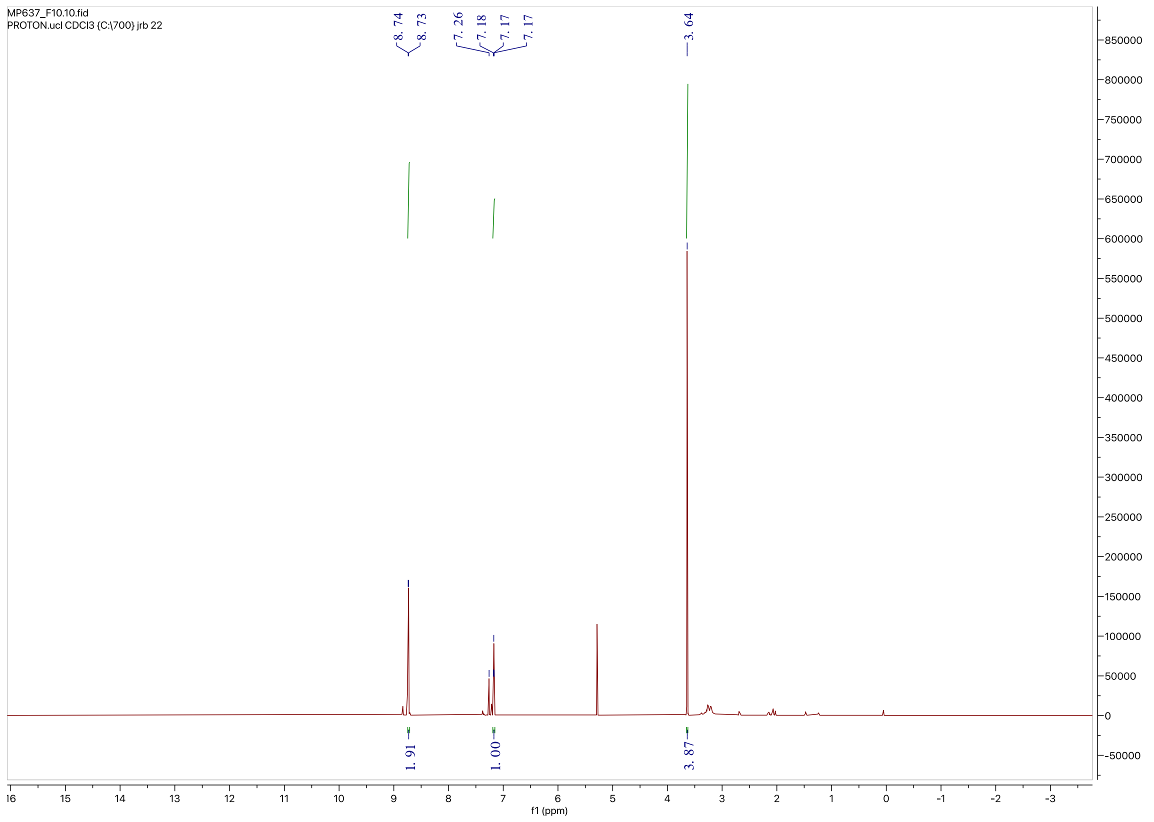
MP637_F10.10.fid
PROTON.ucl CDCl3 {C:\700} jrb 22
8.74
8.73
7.26
7.18
7.17
7.17
3.64
1.91
1.00
3.87
f1 (ppm)

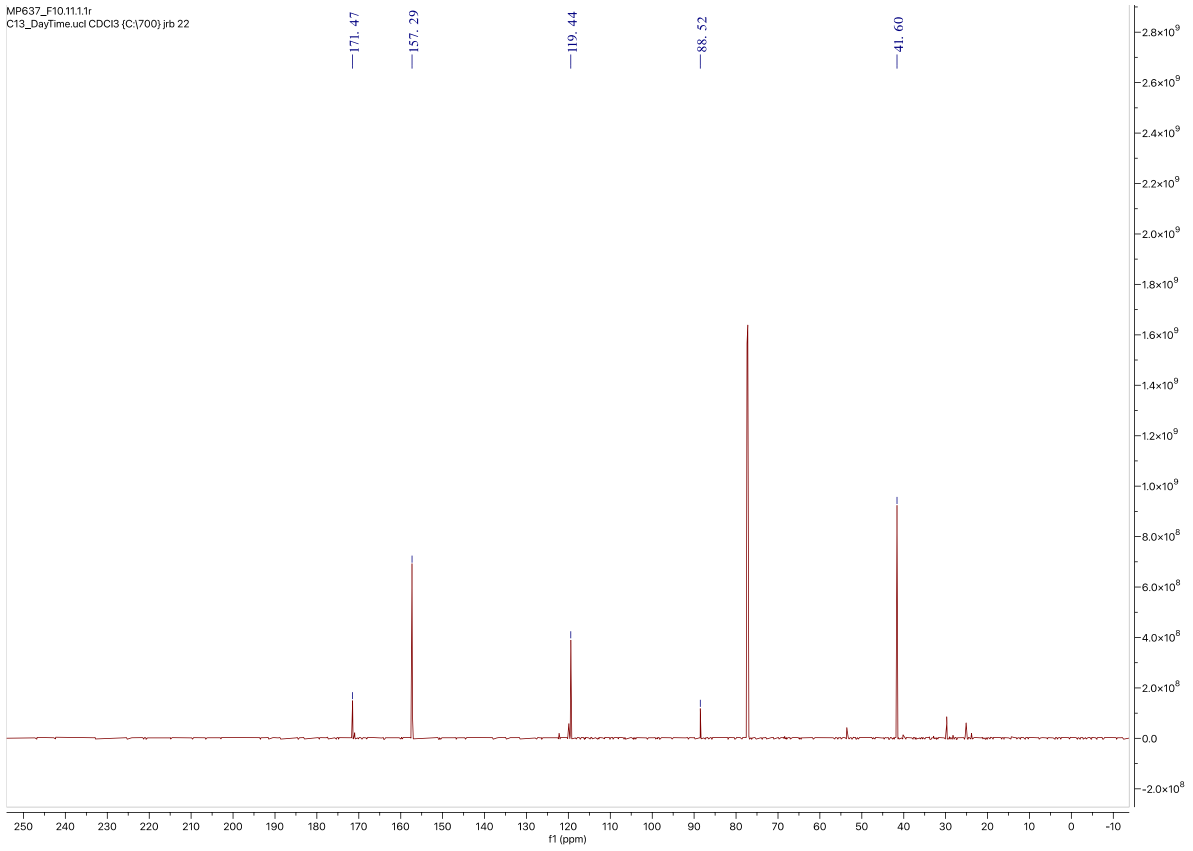
MP637_F10.11.1.1r
C13_DayTime.ucl CDCl3 {C:\700} jrb 22
171.47
157.29
119.44
88.52
41.60
f1 (ppm)

Spiro[pyrrolo[3,4-b]pyrazine-5,2'-[1,3]dithiolan]-7(6H)-imine (**4**)

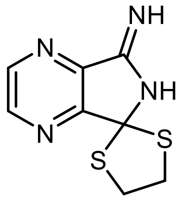

Pyrazine-2,3-dicarbonitrile (130 mg, 1 mmol) was dissolved in MeCN (4 mL) followed by addition of 50 mM Phosphate Buffer pH 7.4 (16 mL). To this mixture was added ethanedithiol (109 µL, 1.2 mmol) was then added and stirred at room temperature for 60 min. The reaction was then extracted with EtOAc (3 X 10 mL). The organic layer was dried over $MgSO_4$ and purified by flash column chromatography (Flashpure, 4 g, eluent 0-10% MeOH: CH_2Cl_2) to afford the title product as a red oil (206 mg, 0.92 mmol, 92%). ^1H NMR (500 MHz, $CDCl_3$) δ 8.61 (d, 1H, *J* = 2.7 Hz), 8.51 (d, 1H, *J* = 2.7 Hz), 3.79 (s, 4H). ^{13}C NMR (126 MHz, $CDCl_3$) δ 166.8 (C), 161.1 (Ar-C), 144.8 (Ar-C), 144.0 (Ar-C), 143.8 (Ar-C), 90.5 (C), 41.7 (CH_2). IR vmax / cm^{-1} 3345, 3082, 2930, 1657.HRMS (ES+) theoretical $[C_8H_8N_4S_2+H]^+$: 225.0258, measured: 225.0263.

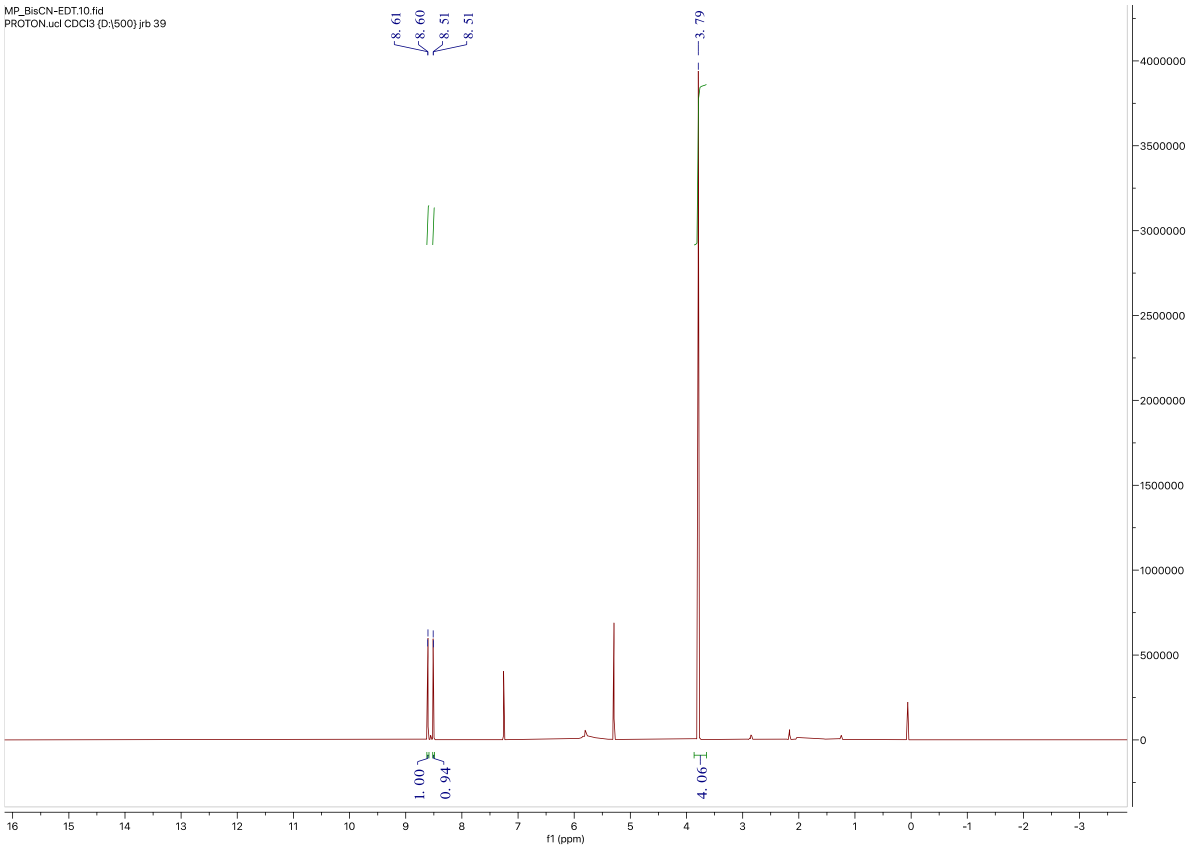

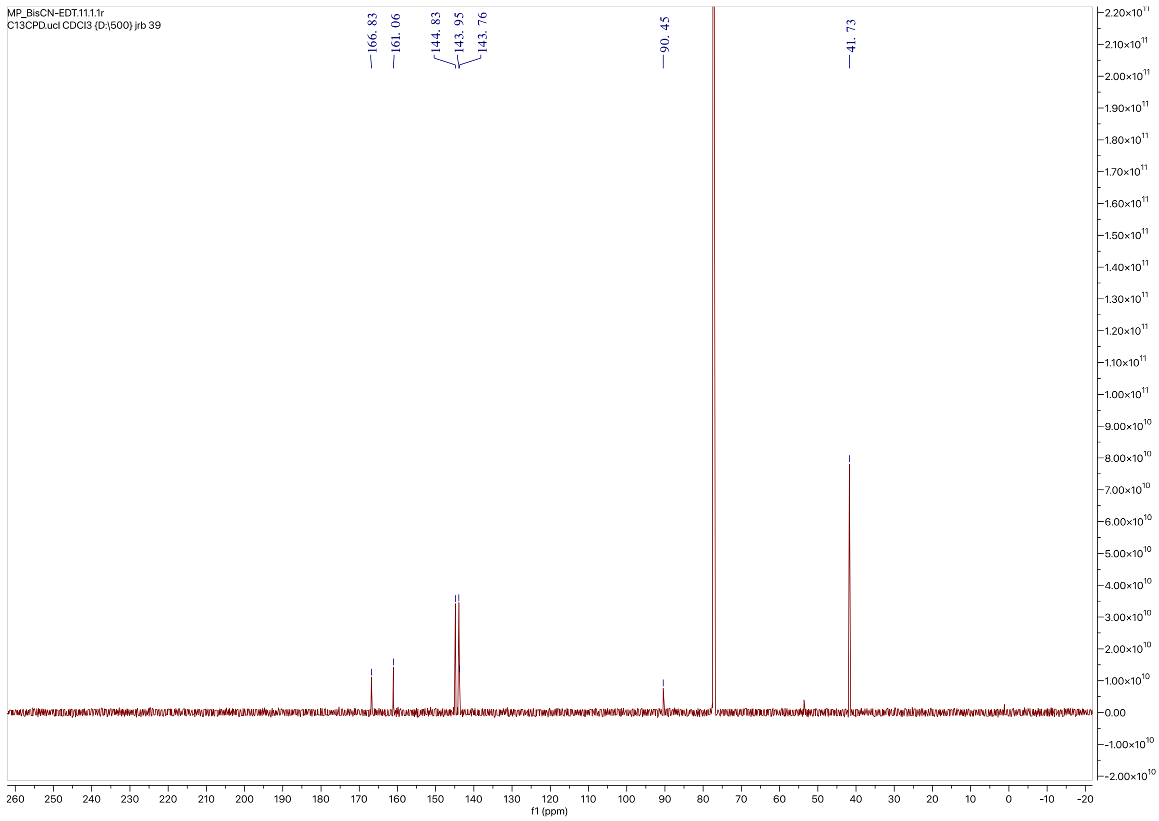

7,7-Bis(ethylthio)-6,7-dihydro-5H-pyrrolo[3,4-b]pyrazin-5-imine (**5**)

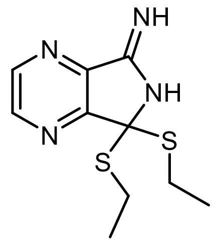

2-Cyano pyrimidine (105 mg, 1 mmol) was dissolved in MeCN (4 mL) followed by addition of 50 mM Phosphate Buffer pH 7.4 (16 mL). To this mixture was added ethanedithiol (109 µL, 1.2 mmol) was then added and stirred at room temperature for 60 min. The reaction was then extracted with EtOAc (3 X 10 mL). The organic layer was dried over $MgSO_4$ and purified by flash column chromatography (Flashpure, 4 g, eluent 0-10% MeOH: CH_2Cl_2) to afford the title product as a red oil (229 mg, 0.90 mmol, 90%).^{1}H NMR (600 MHz, $CDCl_3$) δ 8.60 (d, 1H, *J* = 2.2 Hz), 8.56 (d, 1H, *J* = 2.3 Hz), 2.88-2.73 (m, 4H), 1.19 (t, 6H, *J* = 7.6 Hz). ^{13}C NMR (151 MHz, $CDCl_3$) δ 165.9 (C), 161.2 (Ar-C), 144.4 (Ar-C), 144.1 (Ar-C), 143.4 (Ar-C), 81.8 (C), 25.4 (CH_2), 14.3 (CH_3). IR vmax / cm^{-1} 3344, 3080, 2931, 1657. HRMS (ES+) theoretical $[C_{10}H_{14}N_4S_2+H]^+$: 255.0729, measured: 255.0733.

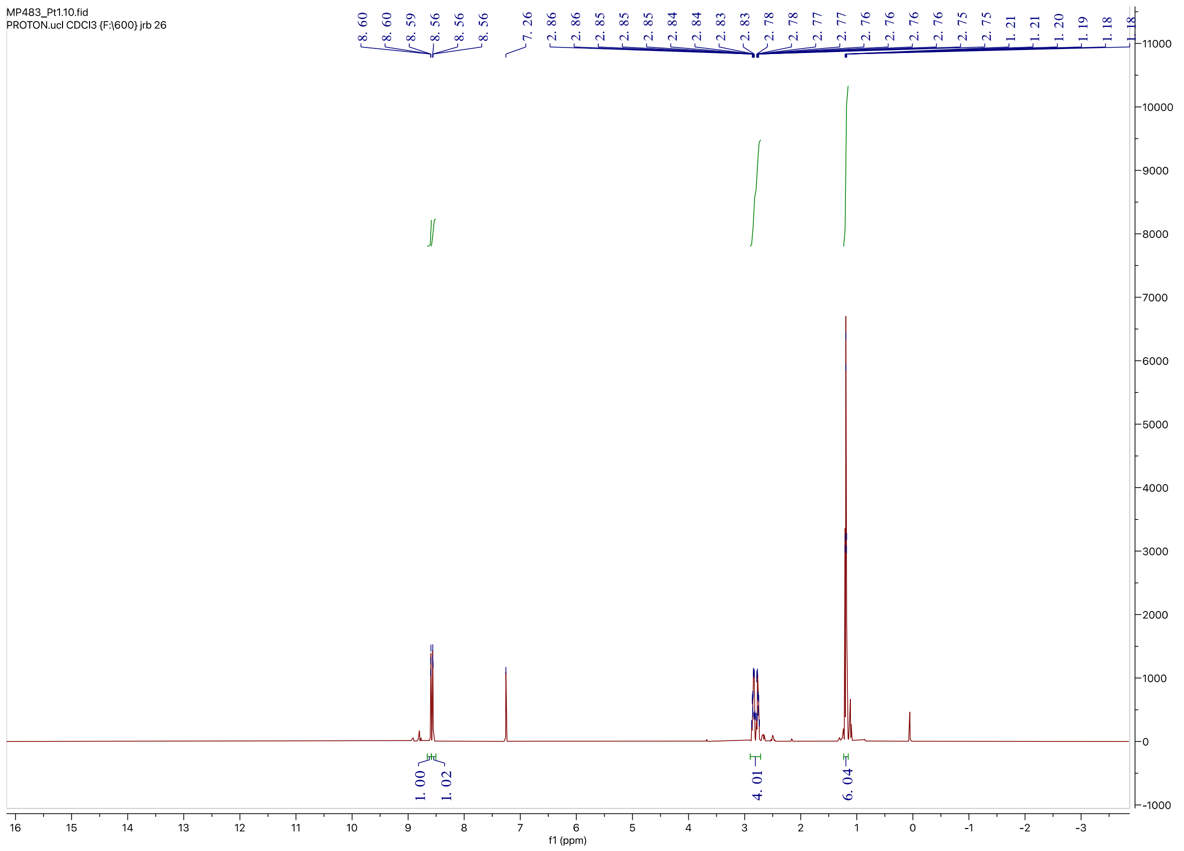
MP483_Pt1.10.fid
PROTON.ucl CDCl3 {F:\600} jrb 26
8.60
8.60
8.59
8.56
8.56
8.56
7.26
2.86
2.86
2.85
2.85
2.85
2.84
2.84
2.83
2.83
2.78
2.78
2.77
2.77
2.76
2.76
2.76
2.76
2.75
2.75
1.21
1.21
1.20
1.19
1.18
1.18
1.00
1.02
4.01
6.04
f1 (ppm)

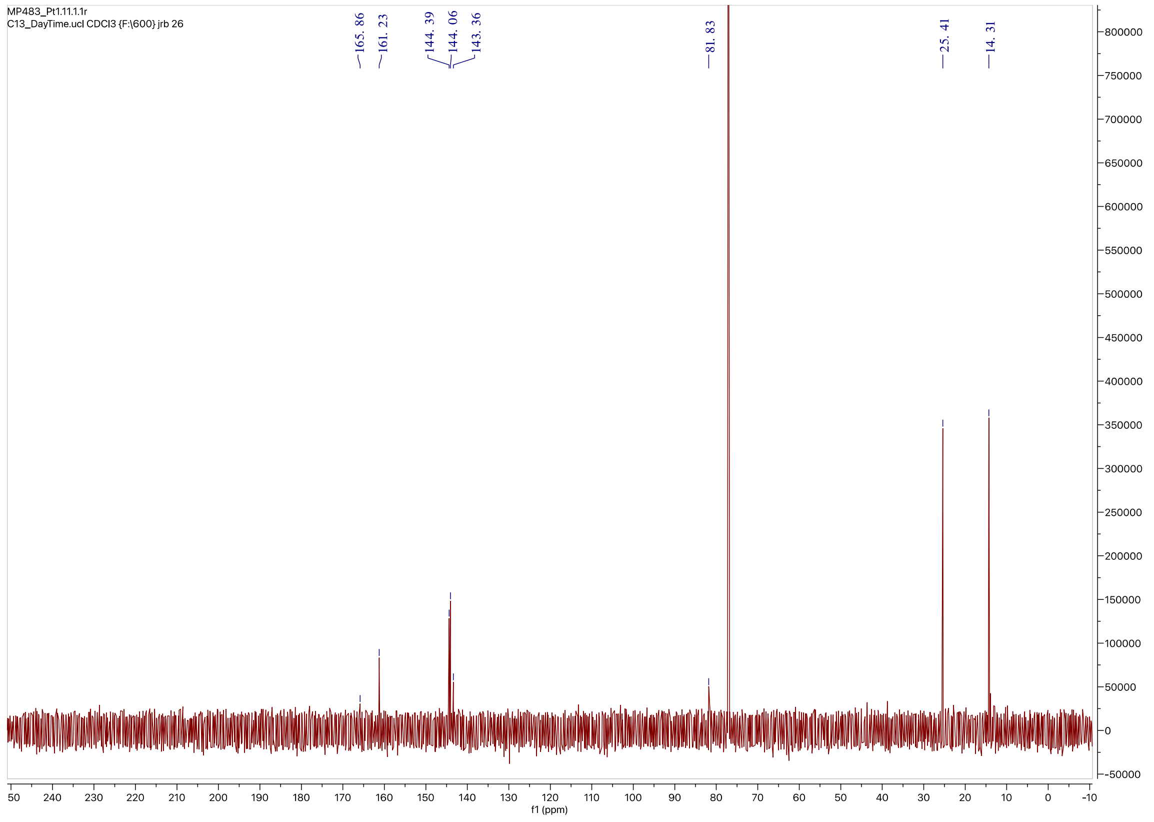
MP483_Pt1.11.1.1r
C13_DayTime.ucl CDCl3 {F:\600} jrb 26
165.86
161.23
144.39
144.06
143.36
81.83
25.41
14.31
f1 (ppm)

5-(Trifluoromethyl)pyrimidine-2-carbonitrile (**S5**)[6]

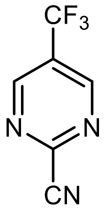

To a solution of DABCO (4.6 mg, 0.041 mmol) in DMSO:H_2O (0.6:0.25 mL) was added finely ground sodium cyanide (15 mg, 0.31 mmol). The flask was then sealed under an atmosphere of nitrogen and cooled to 0 °C. 2-chloro-5-(trifluoromethyl)pyrimidine (50.3 mg, 0.28 mmol) dissolved in DMSO (0.65 mL) was then added dropwise over 15 min, the reaction was then stirred at RT for 2 h. The reaction was then quenched with water (5 mL) and extracted with diethyl ether (3 x 5 mL). The combined organic layers were washed with brine (1 x 3 mL). The organic phase was dried over $MgSO_4$ and concentrated *in vacuo*. Crude 5-(trifluoromethyl)pyrimidine-2-carbonitrile was purified by flash column chromatography (Flashpure, 4g, eluent 0-75% CH_2Cl_2:hexane) to obtain the title product as a pale-yellow oil (33.4 mg, 0.19 mmol, 69%). ^{1}H NMR (600 MHz, $CDCl_3$) δ 9.13 (2H, s). ^{13}C NMR (151 MHz, $CDCl_3$) δ 155.6 (q, J_{CF} = 3.6 Hz), 147.6, 127.0 (q, J_{CF} = 35.2 Hz), 121.8 (q, J_{CF} = 273.2 Hz), 114.8 (**CN**). IR vmax / cm^{-1} 2983 (C-H sp^2), 2166 (C≡N), 1328 (C-F). HRMS (ES+) Theoretical [$C_6H_2N_2F_3$]: 173.0201, Measured : 173.0200.

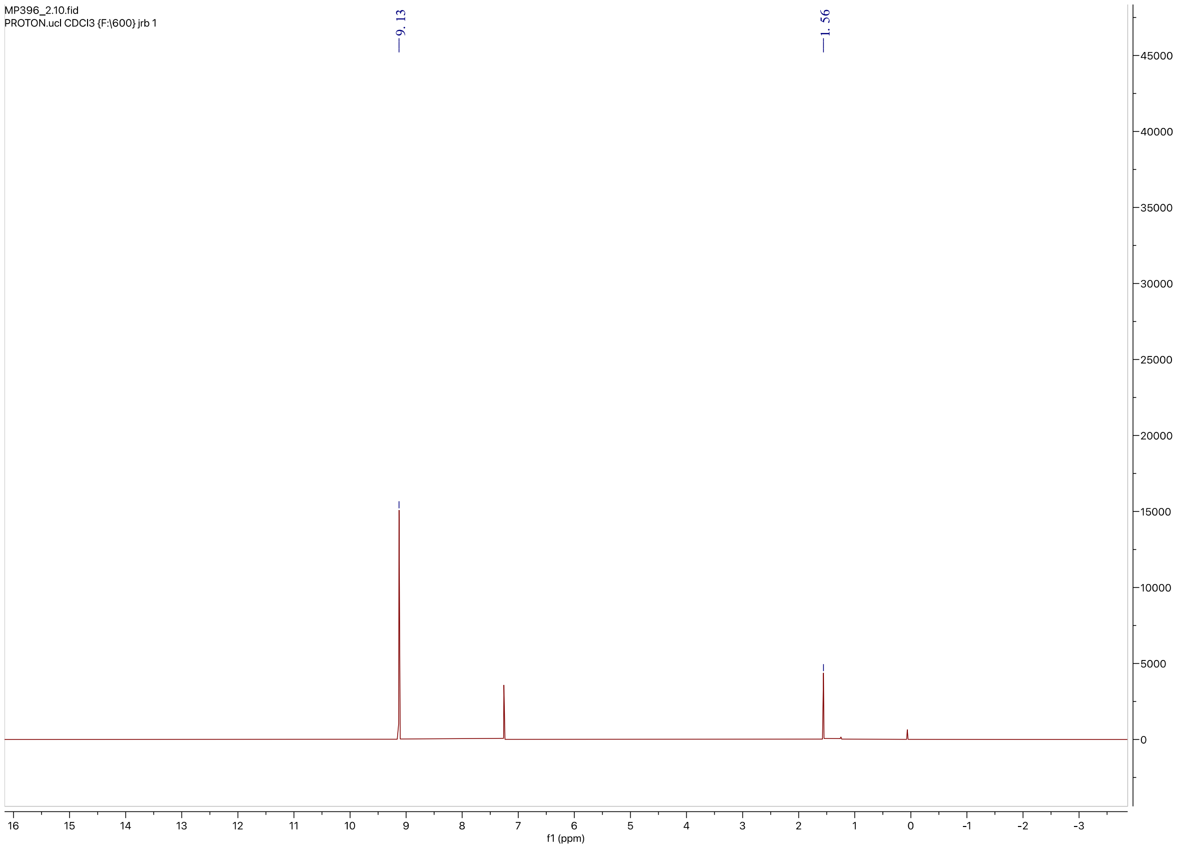

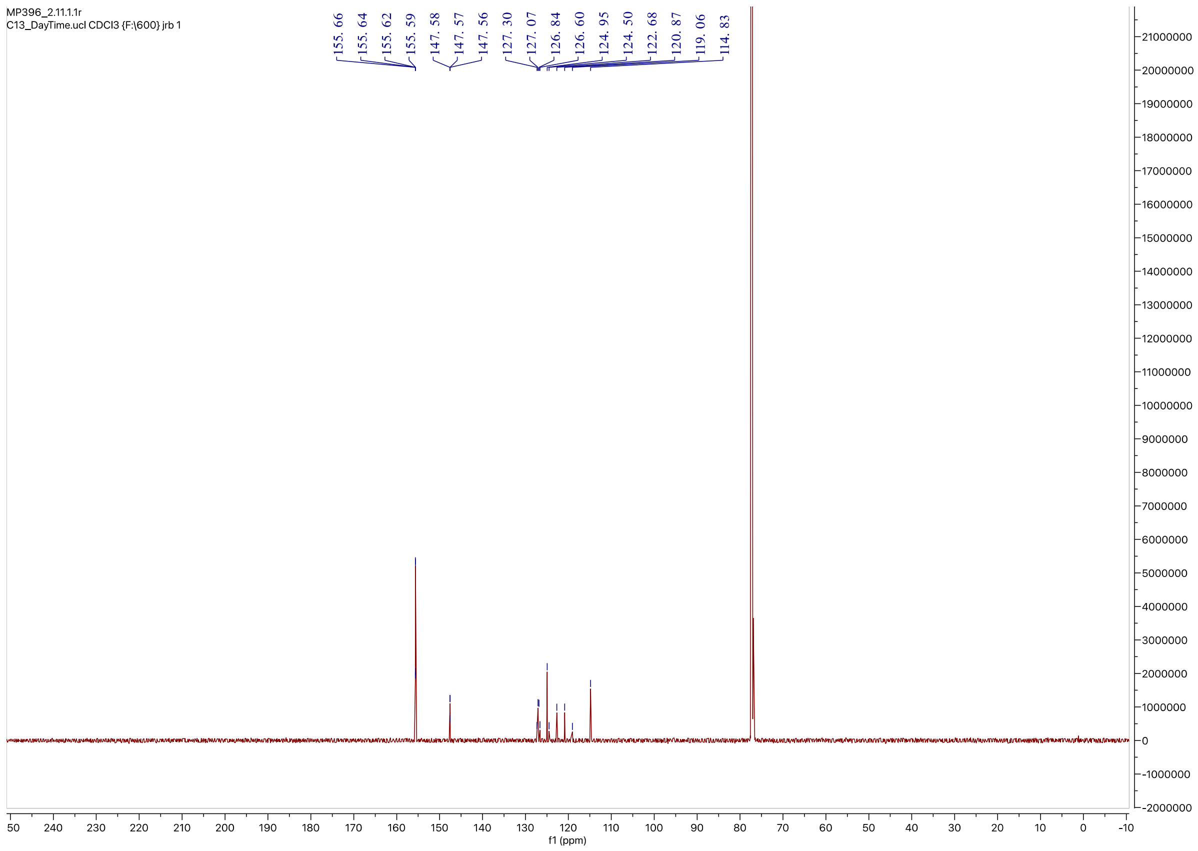

5,6-Dimethyl-3-(methylsulfonyl)-1,2,4-triazine[7]

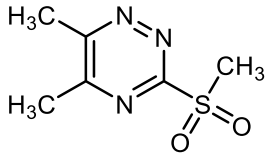

To a solution of freshly distilled 2,3-butanedione (0.226 mL, 2.57 mmol) and sodium bicarbonate (198 mg, 2.36 mmol) in water (4 mL) at RT was added a solution of *S*-methyl isothiosemicarbazide hydroiodide (500 mg, 2.15 mmol) in water (2.5 mL). The mixture was stirred for 16 h at RT. The aqueous layer was then extracted with dichloromethane (3 X 10 mL). The combined organic layers were dried over $MgSO_4$, filtered and concentrated *in vacuo*. Crude 5,6-dimethyl-3-(methylthio)-1,2,4-triazine was carried forward in the next step.

Under a nitrogen atmosphere, mCPBA (753 mg, 4.36 mmol) was added in small portions at 0 °C to a solution of 5,6-dimethyl-3-(methylthio)-1,2,4-triazine (278 mg, 1.79 mol) in dry dichloromethane (11 mL). After complete addition, the mixture was stirred for 2 h at RT and the suspension was filtered through a pad of Celite®. The filtrate was then washed with an aq. saturated solution of sodium bicarbonate (2 X 50 mL) and an aqueous saturated solution of sodium thiosulfate (2 X 50 mL). The organic layer was dried over $MgSO_4$, filtered and concentrated *in vacuo*. The crude product was purified by flash column chromatography

(Flashpure, 12g, eluent 10- 80% EtOAc:Cyclohexane) to obtain the title product as a white solid (175 mg, 0.93 mmol, 52%). m.p 54 130–131°C; ¹H NMR (600 MHz, $CDCl_3$) δ 3.46 (3H, s), 2.81 (3H, s), 2.71 (3H, s); ¹³C NMR (151 MHz, $CDCl_3$) δ 165.3, 162.3, 161.2, 39.8, 22.2, 20.1. IR vmax / cm^{-1} 3009 (C-H sp^2), 2927 (C-H sp^3), 1313 (S=O); Theoretical $[C_6H_9N_3O_2S+H]^+$: 188.0488, Measured : 188.0488.

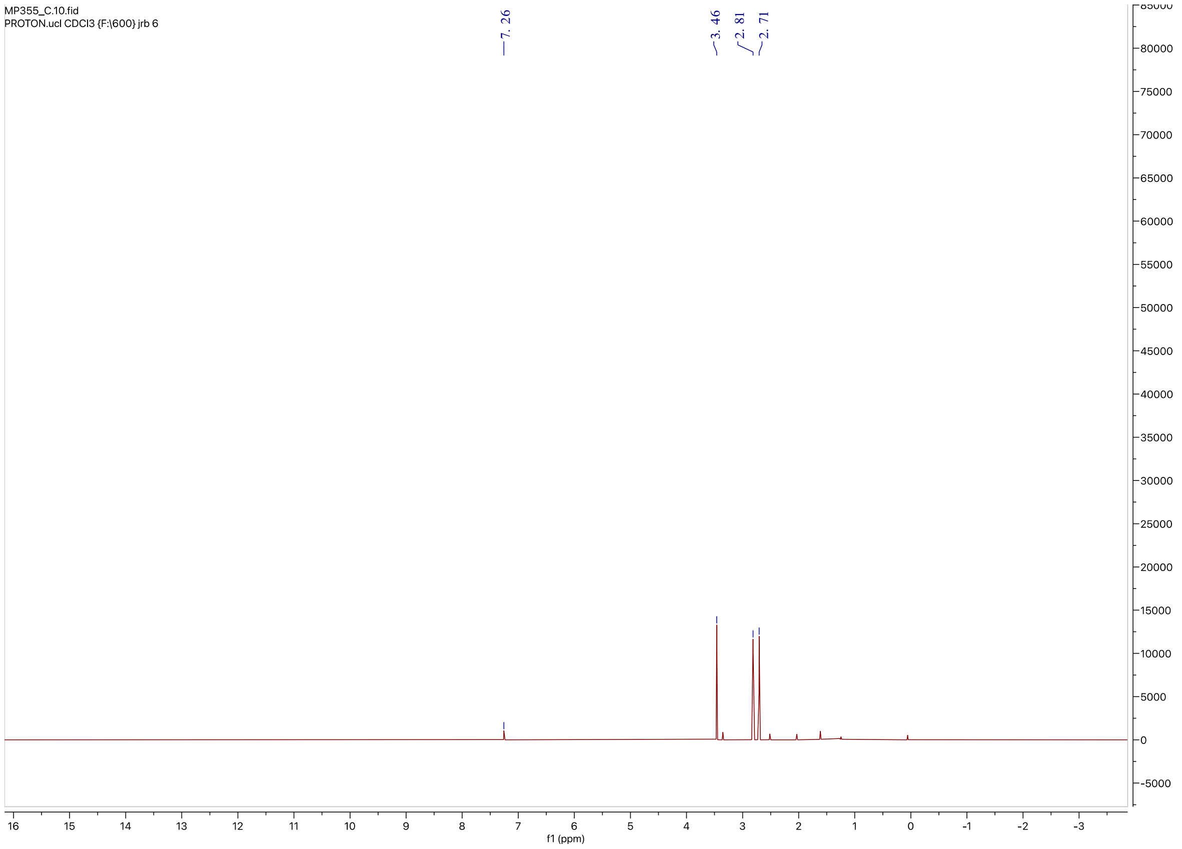

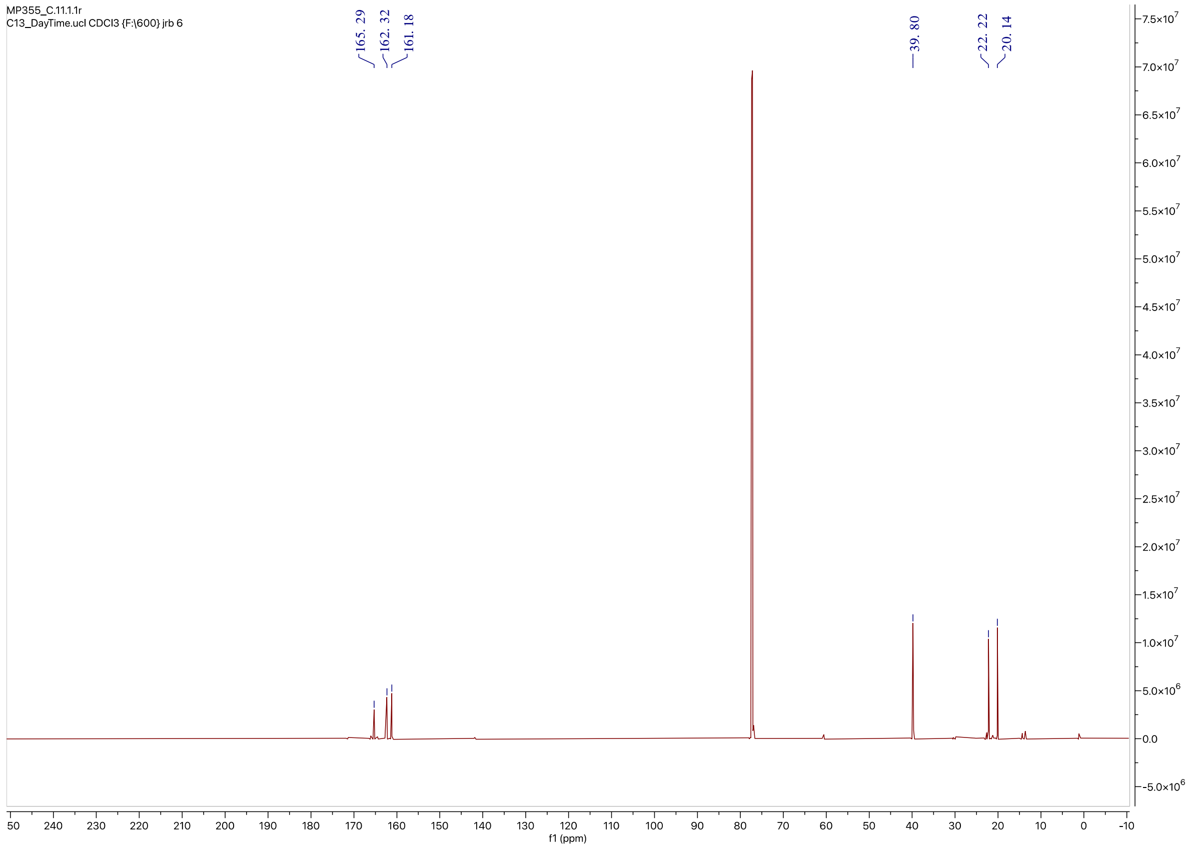

5,6-Dimethyl-1,2,4-triazine-3-carbonitrile (**S6**)

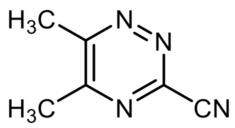

To a solution of 5,6-dimethyl-3-(methylsulfonyl)-1,2,4-triazine (93.6 mg, 0.5 mmol) in DMF (3 mL) was NaCN (27.5 mg, 0.56 mmol). The resulting solution was stirred at RT for 1.5 h. After this time water (5 mL) and EtOAc (3 mL) were added to the mixture. The aqueous layer was extracted with EtOAc (3 x 3 mL). The combined organic phases were then washed with water (3 x 3 mL) and brine (5 mL). Organic phase was dried over $MgSO_4$ and concentrated *in vacuo*. Crude 4,6-dimethoxy-1,3,5-triazine-2-carbonitrile was purified by flash column chromatography (Flashpure, 4g, eluent 10-60% EtOAc:Cyclohexane) to obtain the title product as a brown oil (40.9 mg, 0.305 mmol, 61%). ^{1}H NMR (600 MHz, $CDCl_3$) δ 2.79 (3H, s), 2.63 (3H, s); ^{13}C NMR (151 MHz, $CDCl_3$) δ 160.3, 160.1, 145.6, 114.7 (**CN**), 21.9, 20.3. IR vmax / cm^{-1} 2926 (C-H sp^3), 2333 (C≡N); HRMS (ES+) Theoretical $[C_6H_6N_4+H]^+$: 135.0665 Measured : 135.0662.

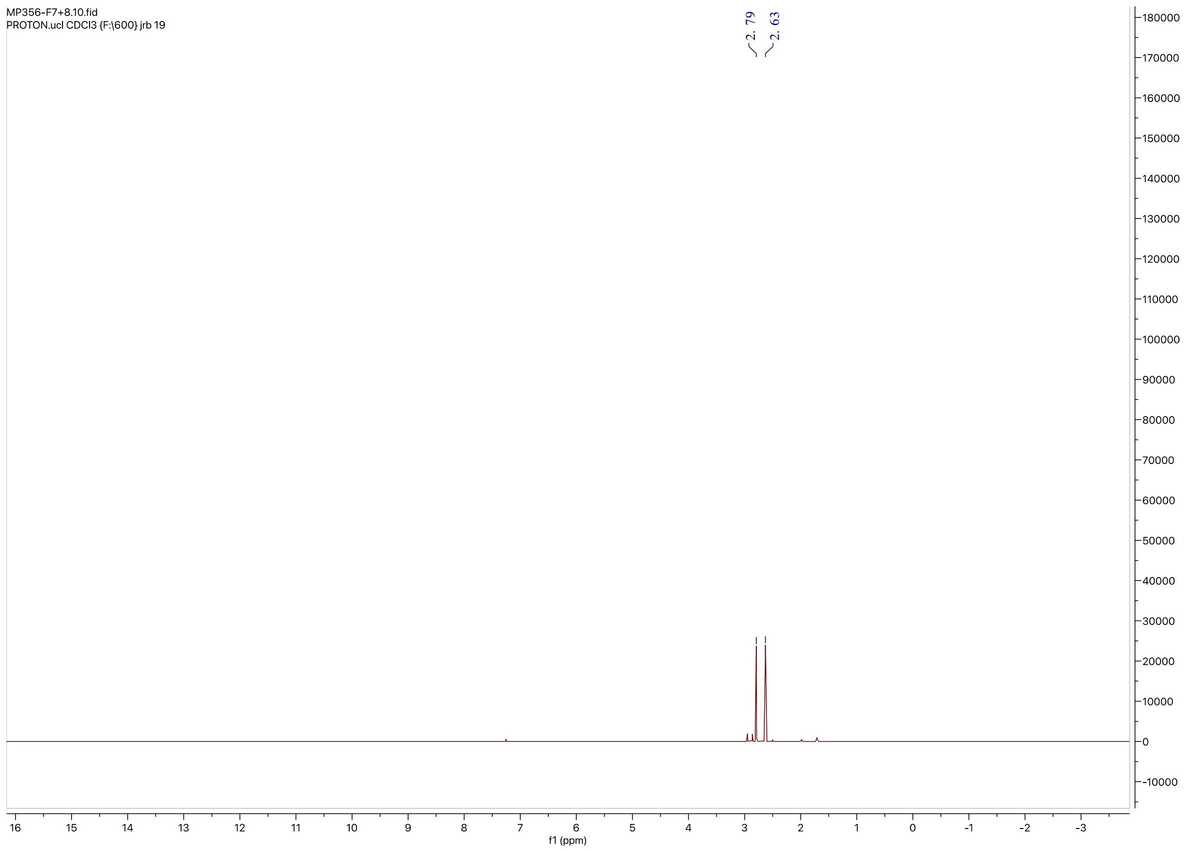
MP356-F7+8.10.fid
PROTON.ucl CDCl3 {F:\600} jrb 19
2.79
2.63
f1 (ppm)

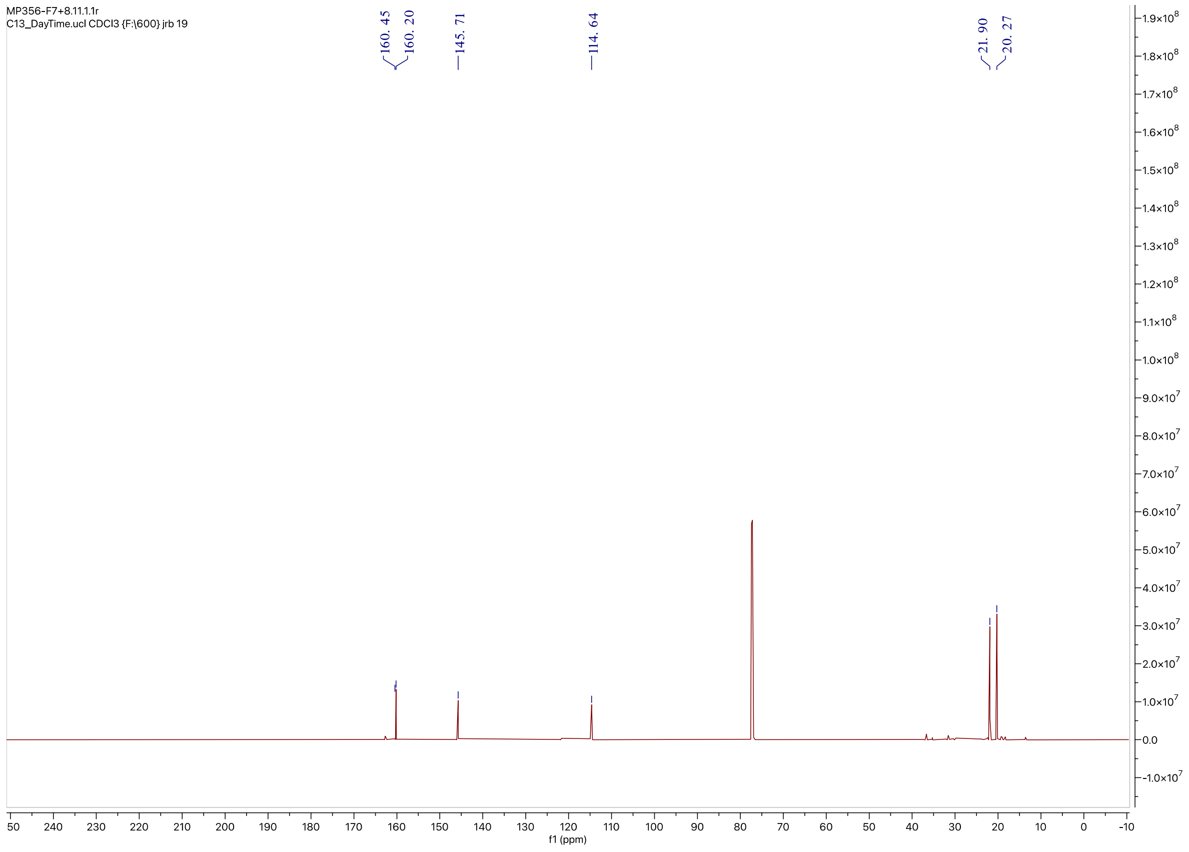
MP356-F7+8.11.1.1r
C13_DayTime.ucl CDCl3 {F:\600} jrb 19
160.45
160.20
145.71
114.64
21.90
20.27
f1 (ppm)

2-(Pyrimidin-2-yl)-1,3-dithian-2-amine - 6 membered ADTA (**S7**)

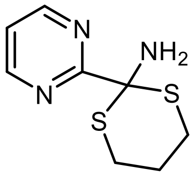

2-Cyano pyrimidine (62 mg, 0.59 mmol) was dissolved in MeCN (4 mL) followed by addition of 50 mM Phosphate Buffer pH 7.4 (16 mL). To this mixture was added 1,3 propanedithiol (71 μL, 0.71 mmol) was then added and stirred at room temperature for 2 h. The reaction was then extracted with EtOAc (3 X 10 mL). The organic layer was dried over $MgSO_4$ and purified by flash column chromatography (Flashpure, 4 g, eluent 0-10% MeOH: CH_2Cl_2) to afford a yellow oil (107.0 mg, 0.50 mmol, 85%). Analysis in D_2O confirmed title product formed, though notably in CD_3CN it was a mixture of this cyclic product and the acyclic thioimidate (see below).

Title product presents an unpleasant smell.

ADTA product (D_2O)

^1H NMR (700 MHz, D_2O) δ 8.79 (d, *J* = 5.0 Hz, 2H), 7.48 (t, *J* = 5.0 Hz, 1H), 3.25 (ddd, *J* = 14.5, 10.3, 2.8 Hz, 2H), 2.98 (ddd, *J* = 14.6, 6.6, 3.2 Hz, 2H), 2.11 (dtt, *J* = 13.7, 6.4, 2.9 Hz, 1H), 1.92 (tdd, *J* = 14.0, 6.8, 3.2 Hz, 1H).

^{13}C NMR (176 MHz, D_2O) δ 168.61, 158.47, 121.67, 69.46, 28.23, 24.52.

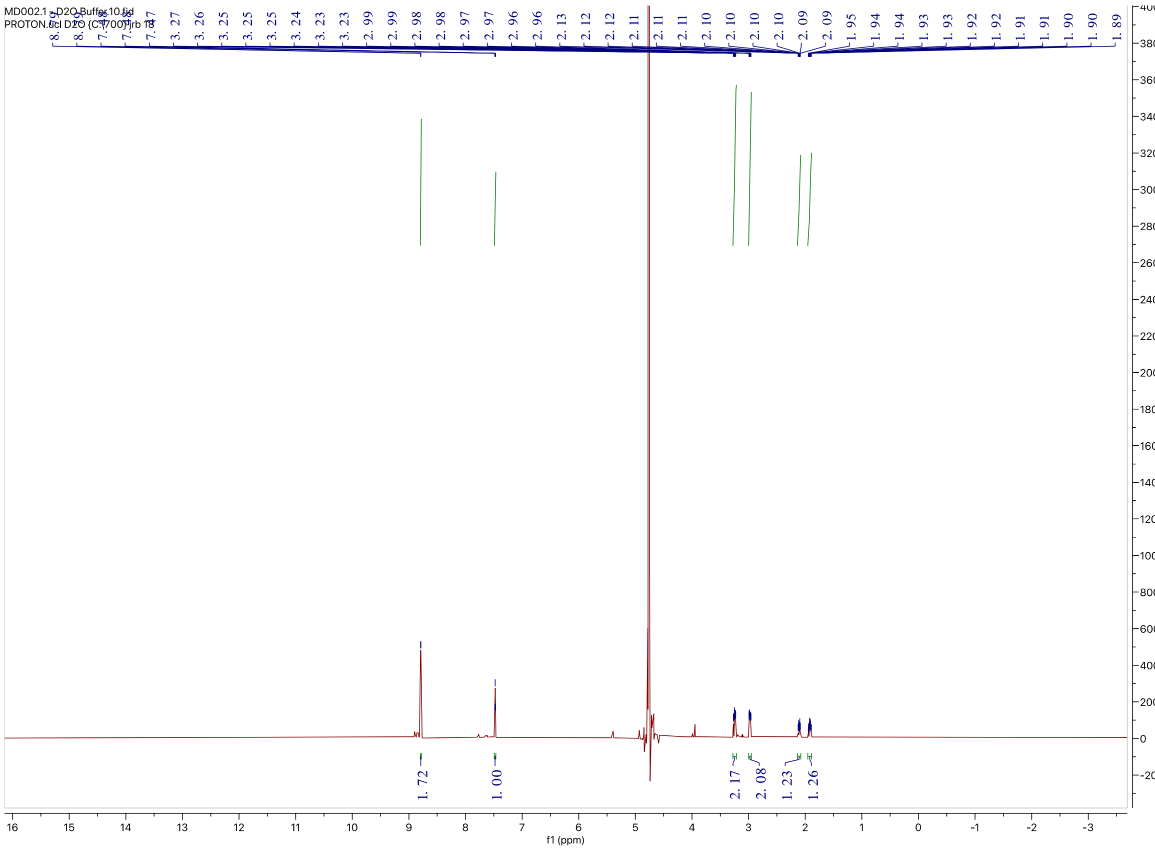

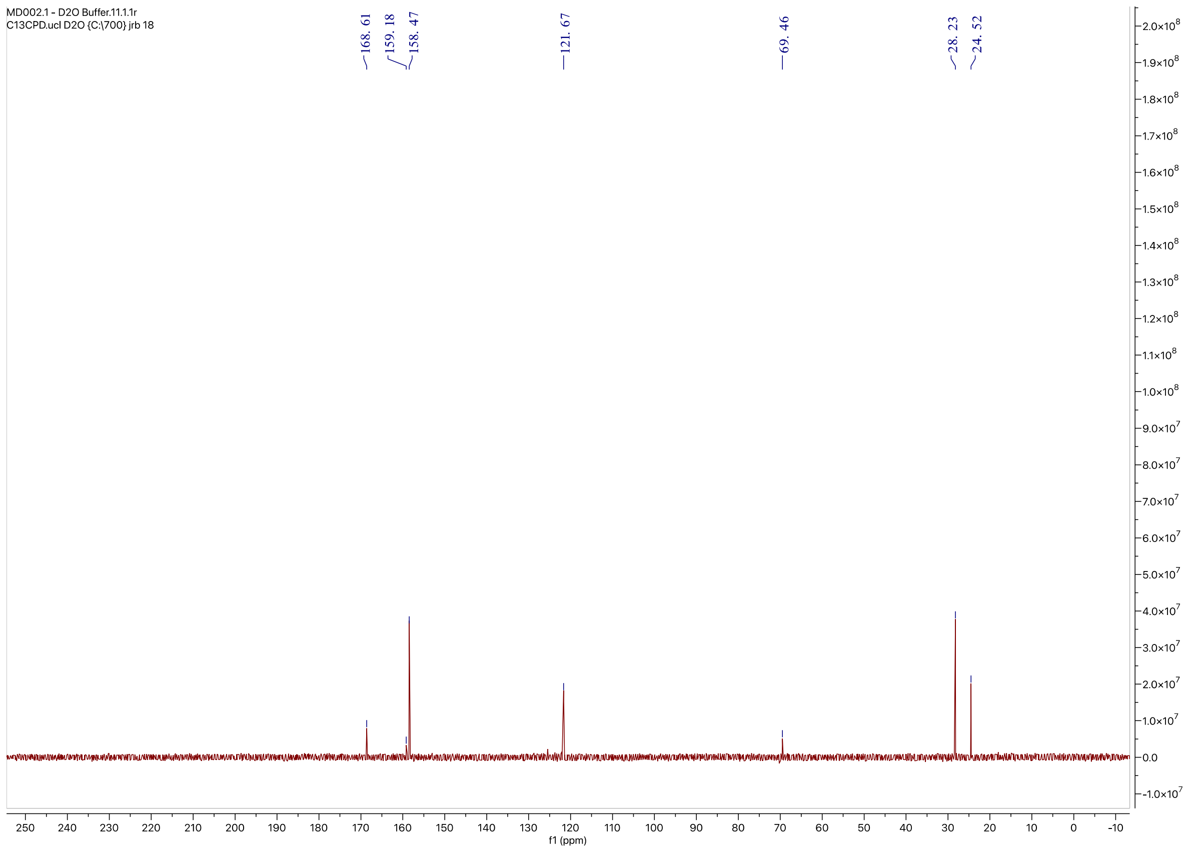

MeCN ratio, ADTA : Thioimidates = 1 : 2.6

ADTA

^{1}H NMR (700 MHz, CD_3CN) δ 8.76 (d, *J* = 4.8 Hz, 2H), 7.32 (t, *J* = 4.8 Hz, 1H), 3.36 (ddd, *J* = 13.2, 9.7, 2.9 Hz, 2H), 3.04 (ddd, *J* = 14.0, 7.3, 3.1 Hz, 2H), 2.09 (dq, *J* = 10.4, 3.6 Hz, 1H), 2.00 (p, *J* = 7.4 Hz, 1H).

Major Thioimidate Product

^{1}H NMR (700 MHz, CD_3CN) δ 8.86 (d, *J* = 4.8 Hz, 2H), 7.50 (t, *J* = 4.8 Hz, 1H), 3.24 (t, *J* = 7.2 Hz, 2H), 3.20 (t, *J* = 7.2 Hz, 2H), 1.94 (p, *J* = 2.5 Hz, 2H).

DCM peak is present in proton NMR. Unknown impurity at 3.94 ppm but does not affect the conclusions drawn.

^{13}C NMR

^{13}C NMR (176 MHz, CD_3CN) δ 174.31, 171.80, 158.64, 158.36, 158.03, 123.61, 121.01, 68.27, 55.35, 33.81, 30.77, 29.48, 28.95, 28.68, 25.90, 24.20.

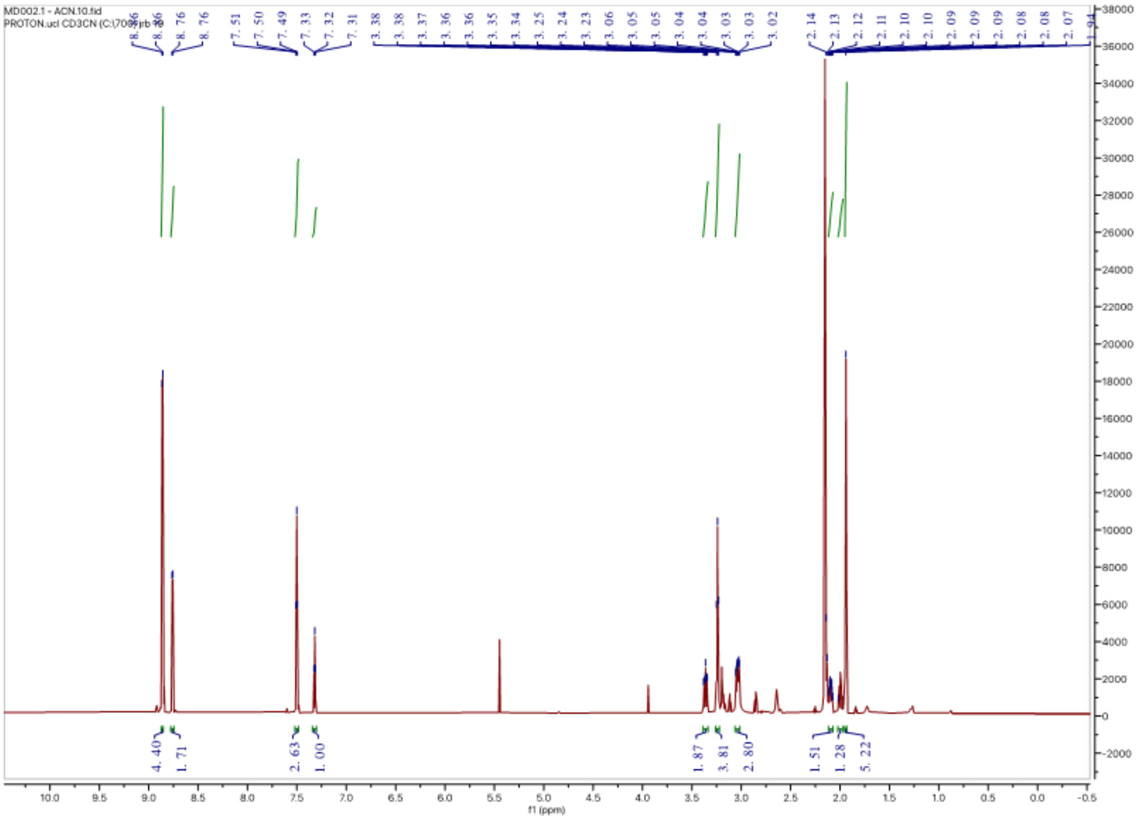
MD002.1 - ACN.10.fid
PROTON.ucl CD3CN {C:\700}jrb 19
8.86 8.86 8.76 8.76 7.51 7.50 7.49 7.33 7.32 7.31 3.38 3.38 3.37 3.36 3.36 3.35 3.34 3.25 3.24 3.23 3.06 3.05 3.05 3.04 3.04 3.03 3.03 3.02 2.14 2.13 2.12 2.11 2.10 2.10 2.09 2.09 2.09 2.08 2.08 2.07 1.94
4.40 1.71 2.63 1.00 1.87 3.81 2.80 1.51 1.28 5.22
f1 (ppm)

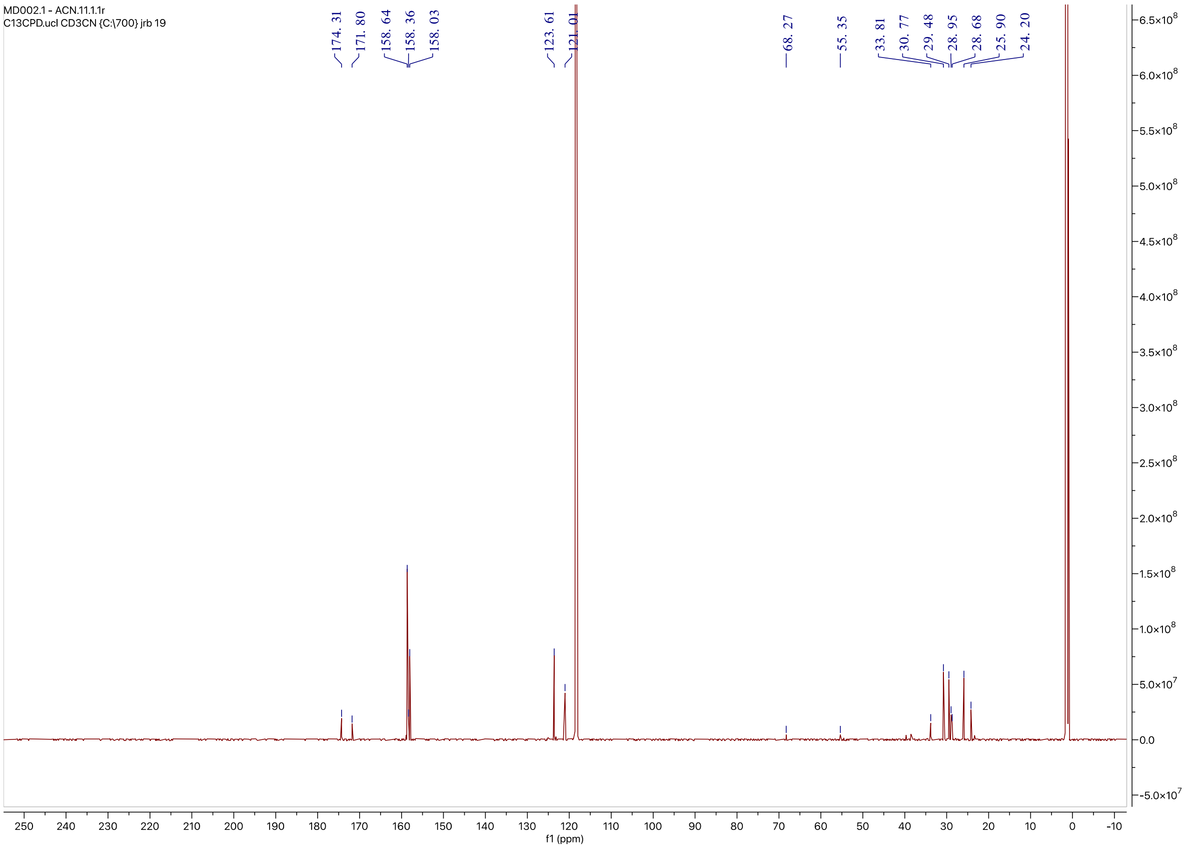
MD002.1 - ACN.11.1.1r
C13CPD.ucl CD3CN {C:\700} jrb 19
174.31 171.80 158.64 158.36 158.03 123.61 121.01 68.27 55.35 33.81 30.77 29.48 28.95 28.68 25.90 24.20
f1 (ppm)

ADTA (**2**) thiol stability studies

ADTA (**2**) incubation with ethanethiol

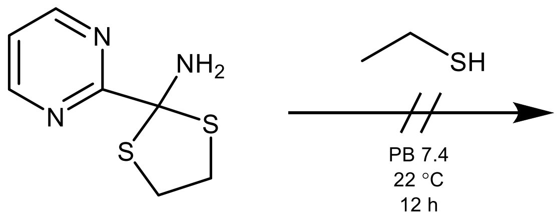

Figure S1; reaction scheme for the incubation of ADTA (**2**) with ethanethiol.

2-(pyrimidin-2-yl)-1,3-dithiolan-2-amine was dissolved in MeCN (10%), to this solution was added 250 mM Phosphate Buffer pH 7.4 (10 mM final concentration) followed by ethanethiol (9 µL, 0.12 mmol). The mixture was tracked *via* NMR. Over 12 h, no changes were seen to the starting material.

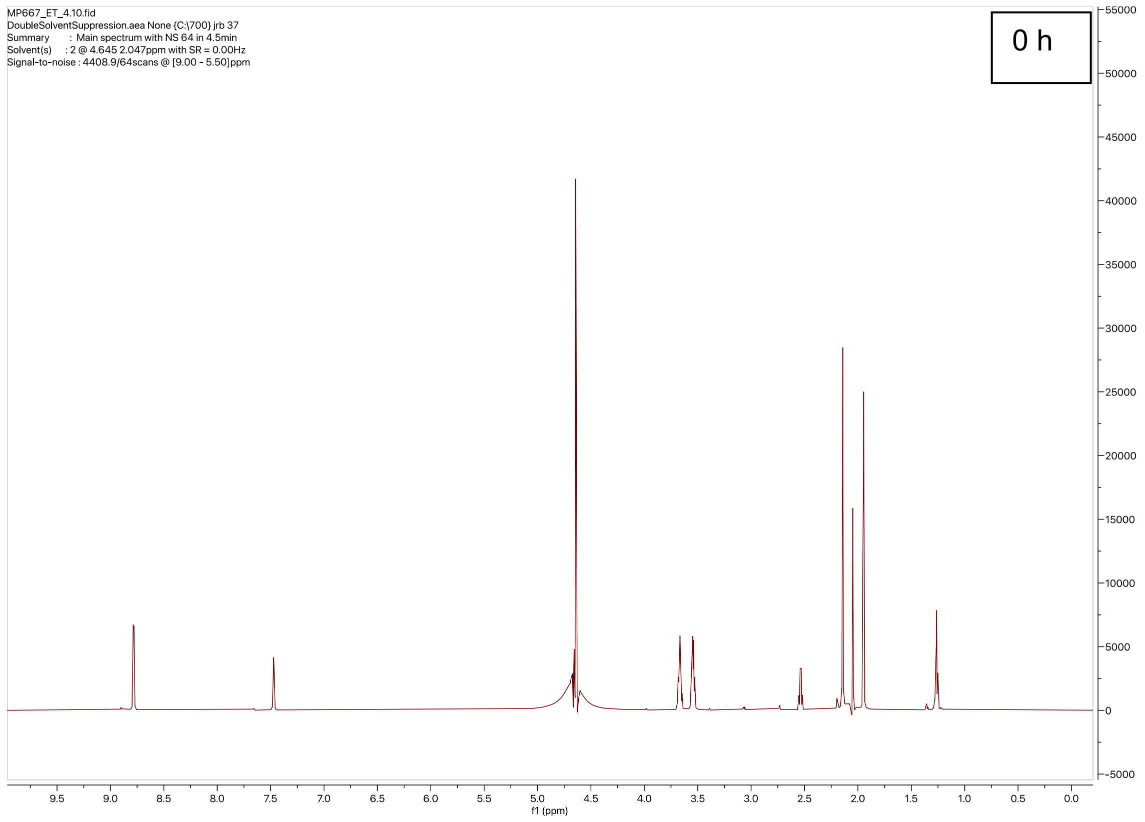
MP667_ET_4.10.fid
DoubleSolventSuppression.aea None {C:\700} jrb 37
Summary : Main spectrum with NS 64 in 4.5min
Solvent(s) : 2 @ 4.645 2.047ppm with SR = 0.00Hz
Signal-to-noise : 4408.9/64scans @ [9.00 - 5.50]ppm
0 h
f1 (ppm)

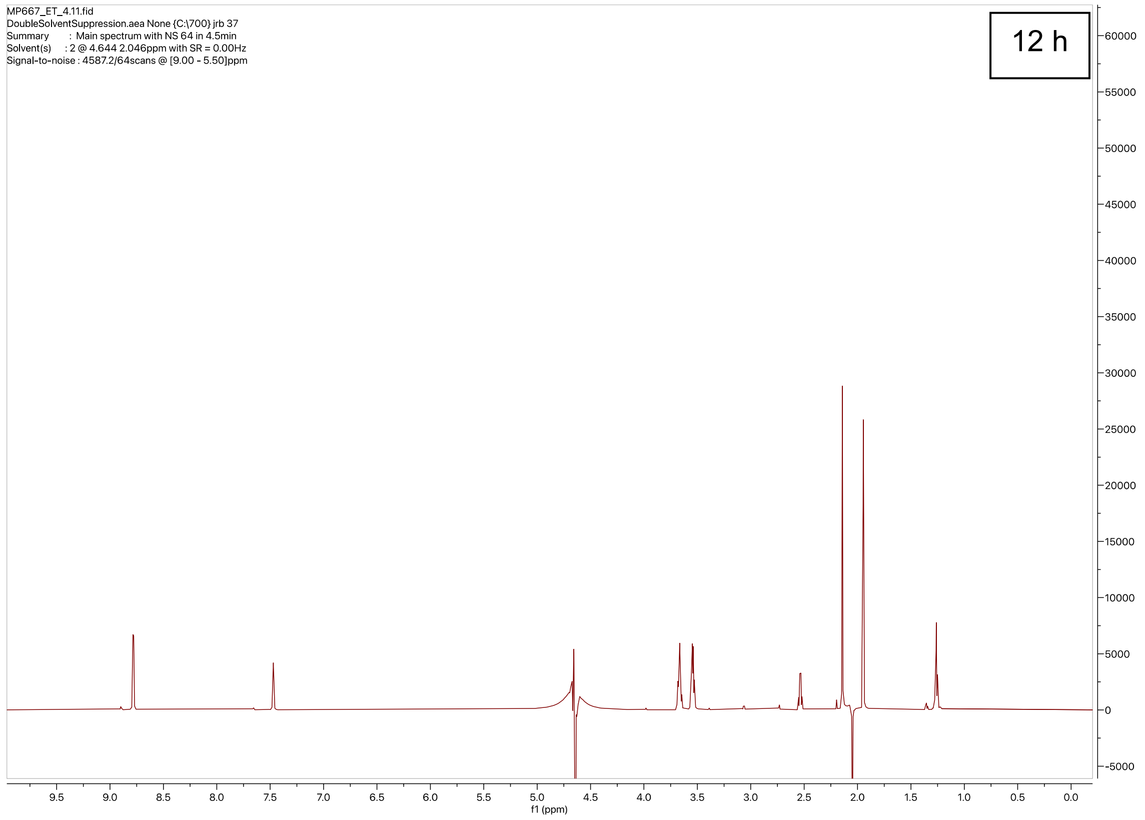
MP667_ET_4.11.fid
DoubleSolventSuppression.aea None {C:\700} jrb 37
Summary : Main spectrum with NS 64 in 4.5min
Solvent(s) : 2 @ 4.644 2.046ppm with SR = 0.00Hz
Signal-to-noise : 4587.2/64scans @ [9.00 - 5.50]ppm
12 h
f1 (ppm)

ADTA (**2**) aqueous stability

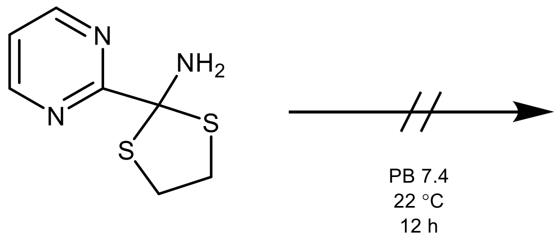

Figure S2; incubation of ADTA (**2**) with in phosphate buffer (7.4).

2-(Pyrimidin-2-yl)-1,3-dithiolan-2-amine was dissolved in 250 mM Phosphate Buffer pH 7.4 (10 mM), The mixture was tracked *via* NMR. Over 12 h, no changes were seen to the starting material.

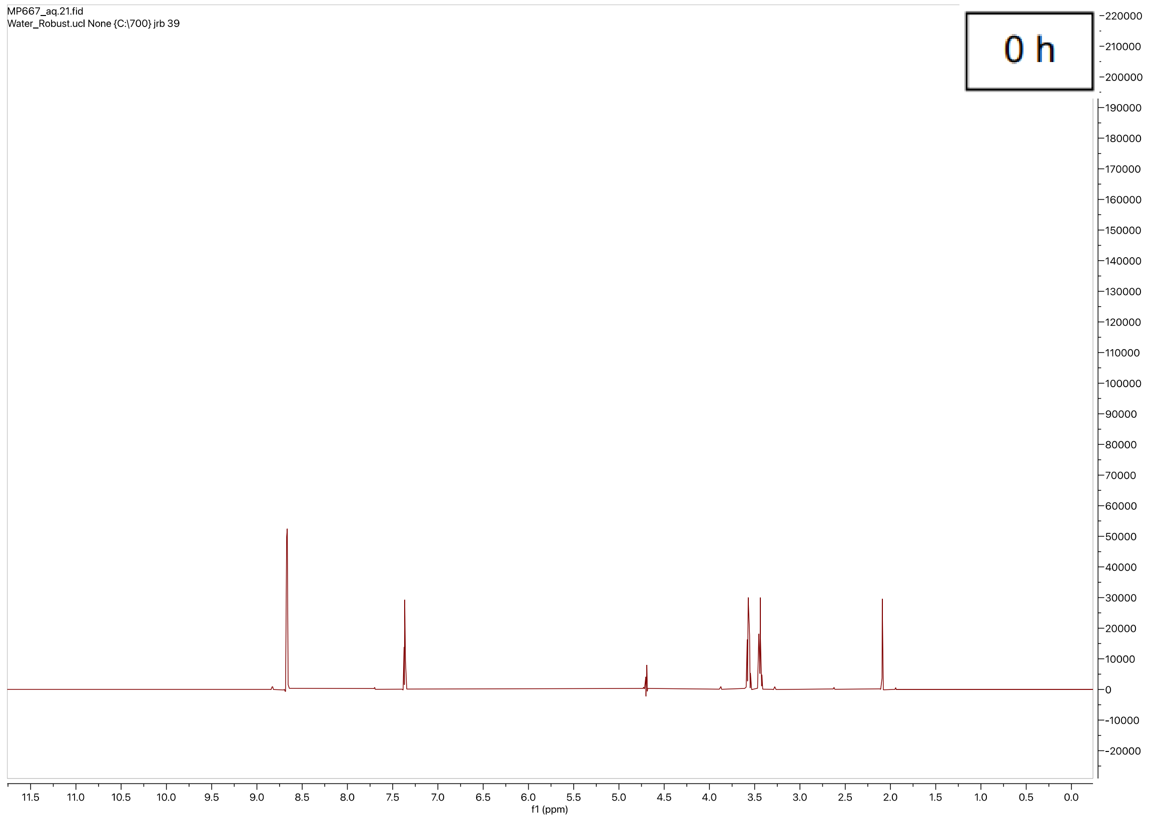

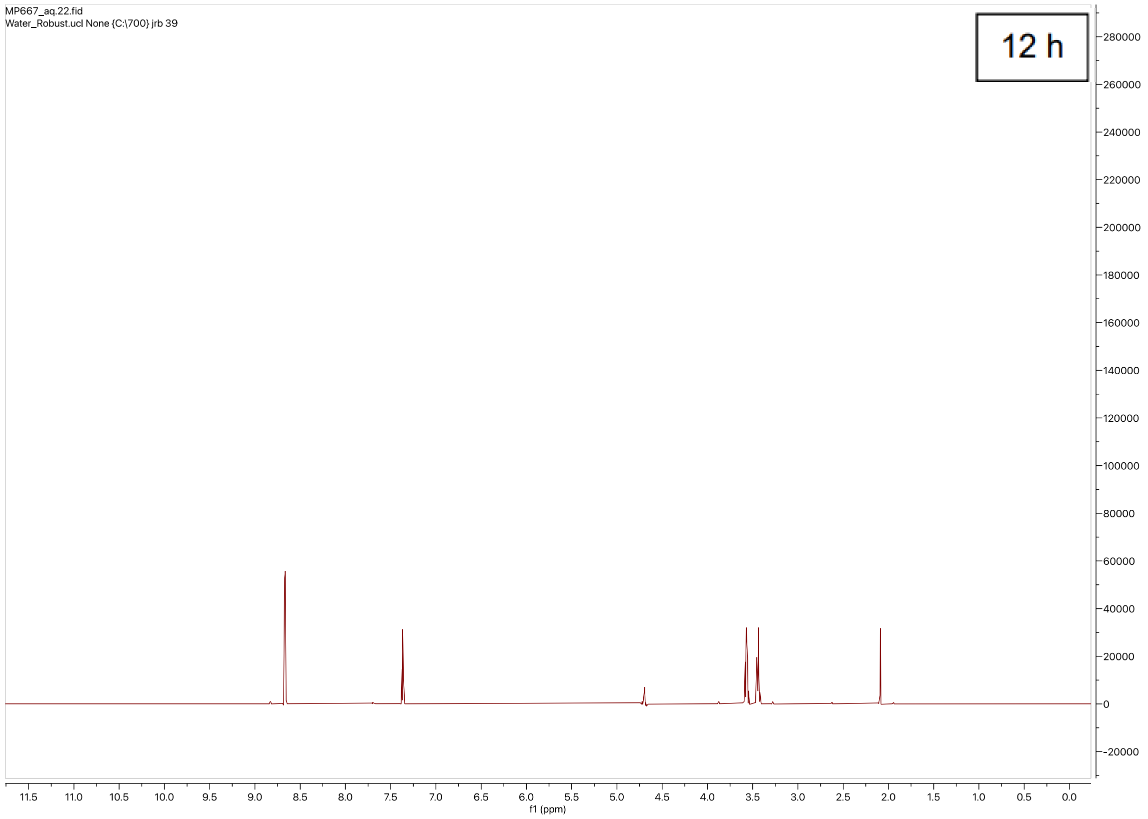

Ethanedithiol Vs. Propanedithiol NBT competition experiment

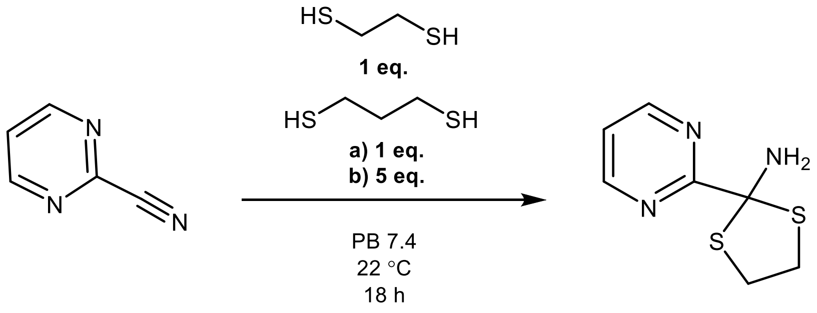

Figure S3; reaction scheme for the competition NBT formation of ADTA (**2**) with ethanedithiol and propanedithiol.

Ethanedithiol (49.6 µL, 0.59 mmol) and propanedithiol ((a) 59.1 µL, 0.59 mmol, 1 eq. / (b) 295 µL, 2.95 mmol, 5 eq.) were dissolved in MeCN (5 mL) followed by addition of 50 mM Phosphate Buffer pH 7.4 (13 mL). To this mixture was added 2-cyano pyrimidine (62 mg, 0.59 mmol) dissolved in MeCN (2 mL). The reaction was stirred for 18 h at RT before being extracted with EtOAc (3 X 10 mL). The organic layer was dried over $MgSO_4$ and purified by flash column chromatography (Flashpure, 4 g, eluent 0-10% MeOH: CH_2Cl_2) to afford 2-(pyrimidin-2-yl)-1,3-dithiolan-2-amine as a yellow-brown oil ((a) 88.0 mg ,0.44 mmol, 75% (b)

84.5 mg, 0.42 mmol, 72%). ^{1}H NMR (700 MHz, $CDCl_3$) δ 8.71 (d, 2H, J = 4.8 Hz), 7.15 (t, 1H, J = 4.8 Hz), 3.61 (s, 4H).

*NMR as before (**2**).*

*This insitu reaction highlights the 1,2-ethanedithiol selectivity over 1,3-propanedithiol. This is shown by the clear shift in aromatic protons in the line with the EDT-ADTA (**2**) upon addition of 1,2-ethanedithiol to **S7**.*

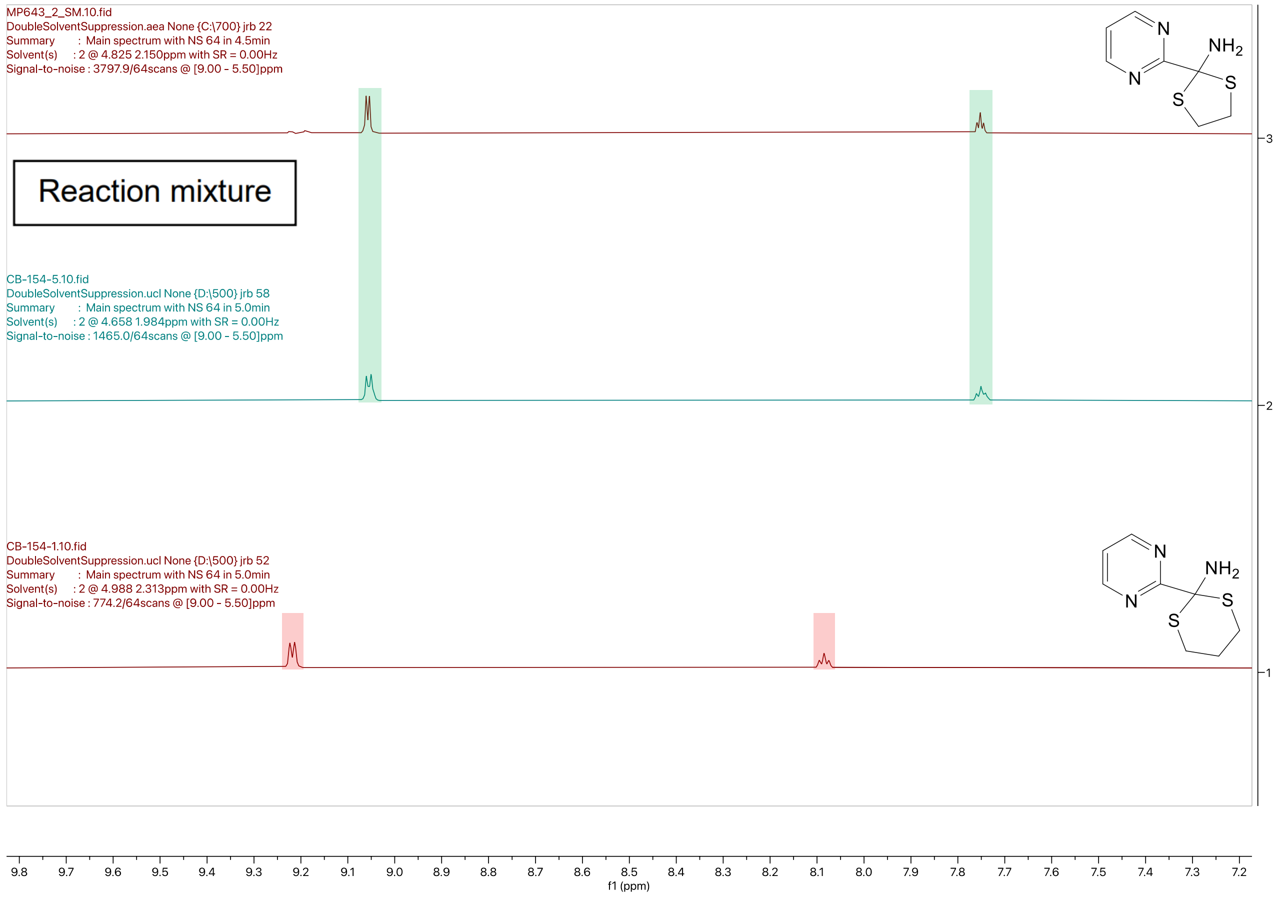

ADTA (2) reversibility- *N*-methyl maleimide

A 10 mM solution of 2-(pyrimidin-2-yl)-1,3-dithiolan-2-amine was prepared in PB (7.4)/ 20% MeCN (0.4 mL). To this solution was added *N*-methyl maleimide (3 eq.) in 0.1 mL MeCN. The reaction was analysed *via* a double solvent suppression ^{1}H NMR method and ^{13}C NMR.

(a)

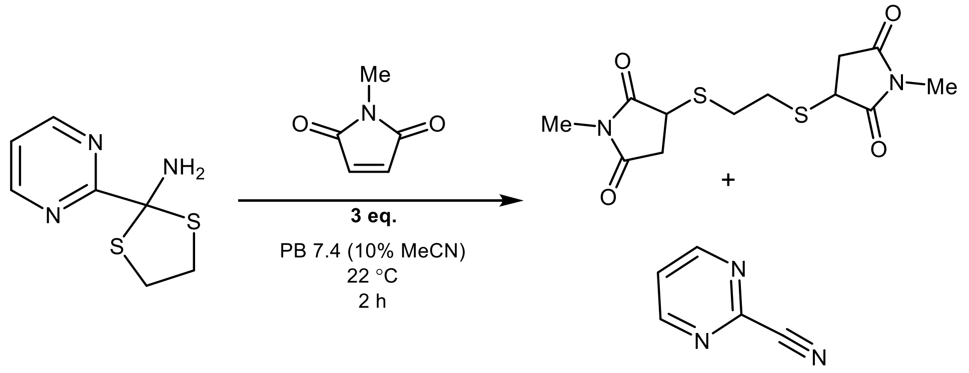

(b)

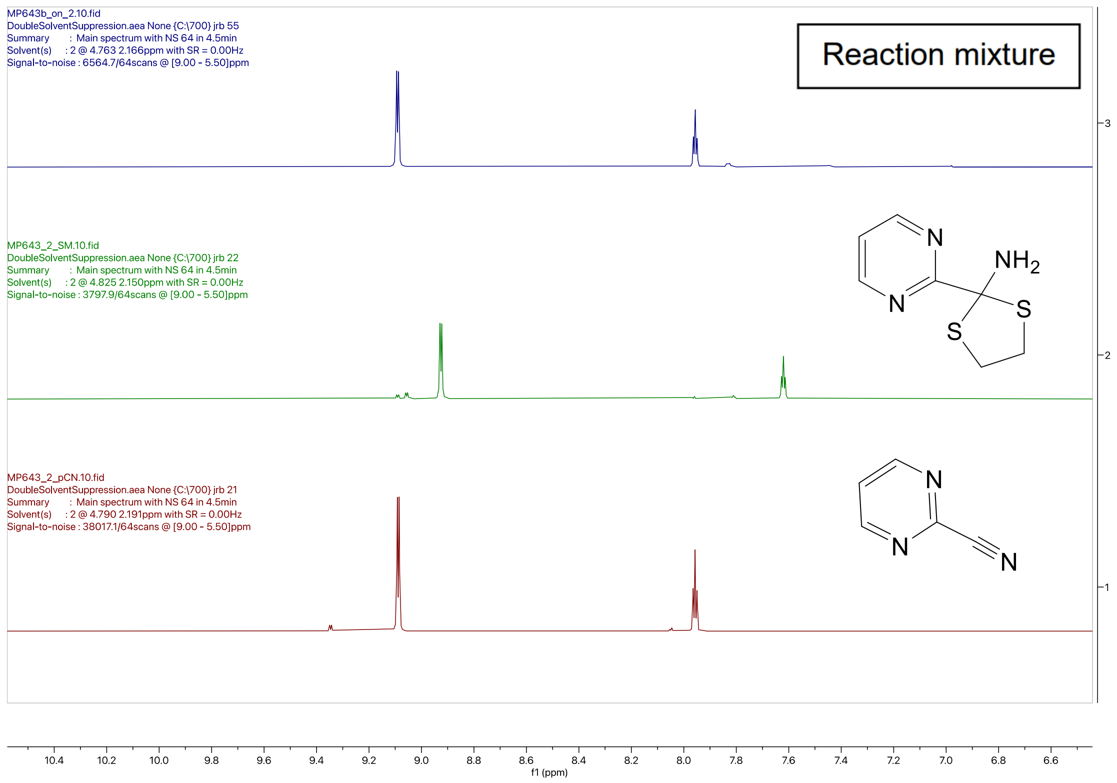
MP643b_on_2.10.fid
DoubleSolventSuppression.aea None {C:\700} jrb 55
Summary : Main spectrum with NS 64 in 4.5min
Solvent(s) : 2 @ 4.763 2.166ppm with SR = 0.00Hz
Signal-to-noise : 6564.7/64scans @ [9.00 - 5.50]ppm
Reaction mixture
MP643_2_SM.10.fid
DoubleSolventSuppression.aea None {C:\700} jrb 22
Summary : Main spectrum with NS 64 in 4.5min
Solvent(s) : 2 @ 4.825 2.150ppm with SR = 0.00Hz
Signal-to-noise : 3797.9/64scans @ [9.00 - 5.50]ppm
MP643_2_pCN.10.fid
DoubleSolventSuppression.aea None {C:\700} jrb 21
Summary : Main spectrum with NS 64 in 4.5min
Solvent(s) : 2 @ 4.790 2.191ppm with SR = 0.00Hz
Signal-to-noise : 38017.1/64scans @ [9.00 - 5.50]ppm
f1 (ppm)

(c)

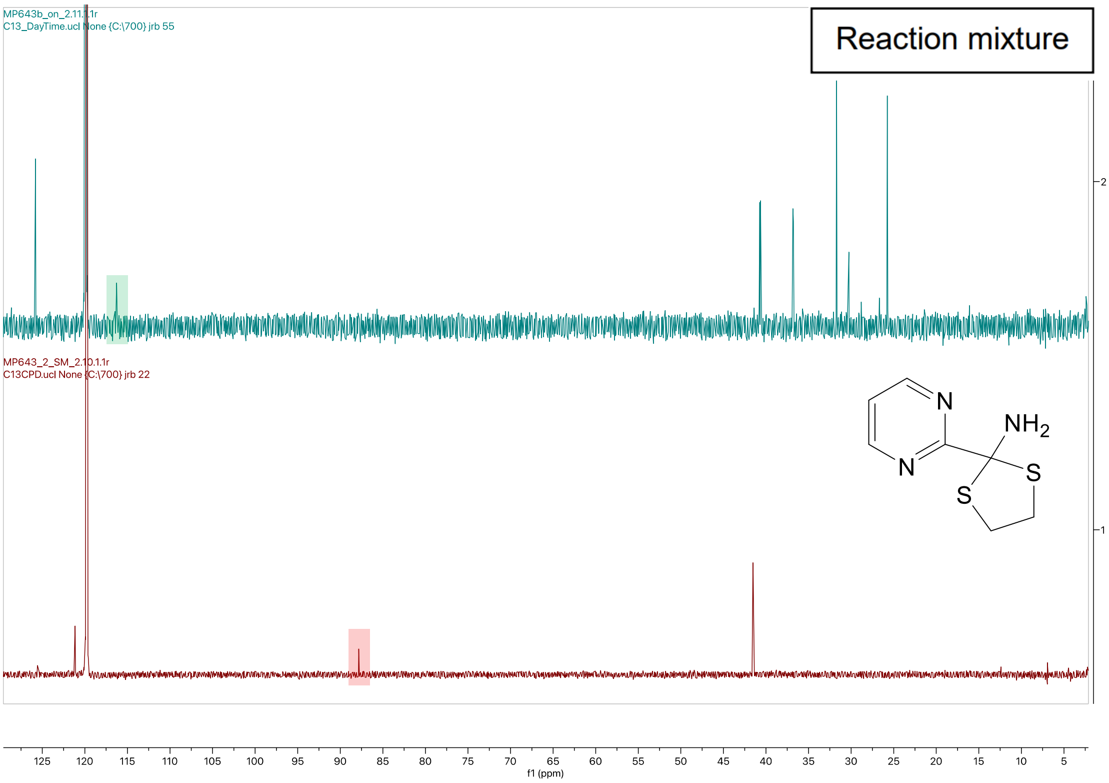
MP643b_on_2.11.1.1r
C13_DayTime.ucl None {C:\700} jrb 55
Reaction mixture
MP643_2_SM_2.10.1.1r
C13CPD.ucl None {C:\700} jrb 22
N
N
NH2
S
S
f1 (ppm)

(d)

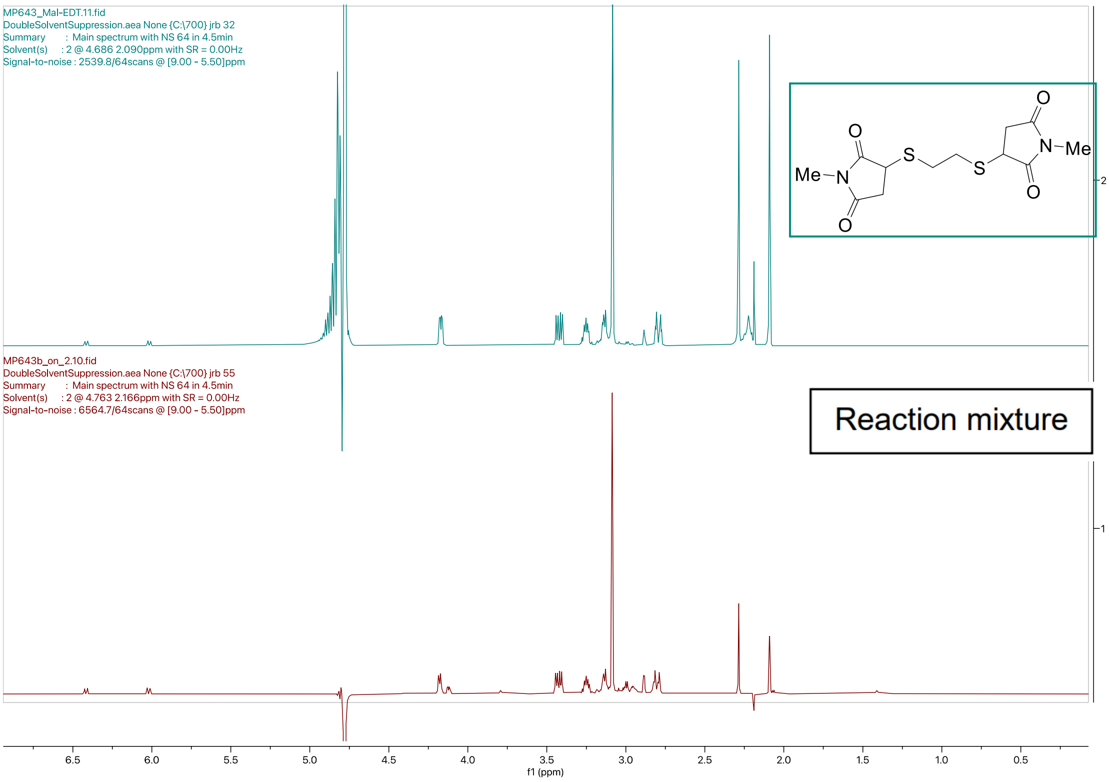
MP643_Mal-EDT.11.fid
DoubleSolventSuppression.aea None {C:\700} jrb 32
Summary : Main spectrum with NS 64 in 4.5min
Solvent(s) : 2 @ 4.686 2.090ppm with SR = 0.00Hz
Signal-to-noise : 2539.8/64scans @ [9.00 - 5.50]ppm
Me−N
N−Me
S
S
O
O
O
O
MP643b_on_2.10.fid
DoubleSolventSuppression.aea None {C:\700} jrb 55
Summary : Main spectrum with NS 64 in 4.5min
Solvent(s) : 2 @ 4.763 2.166ppm with SR = 0.00Hz
Signal-to-noise : 6564.7/64scans @ [9.00 - 5.50]ppm
Reaction mixture
f1 (ppm)

(e)

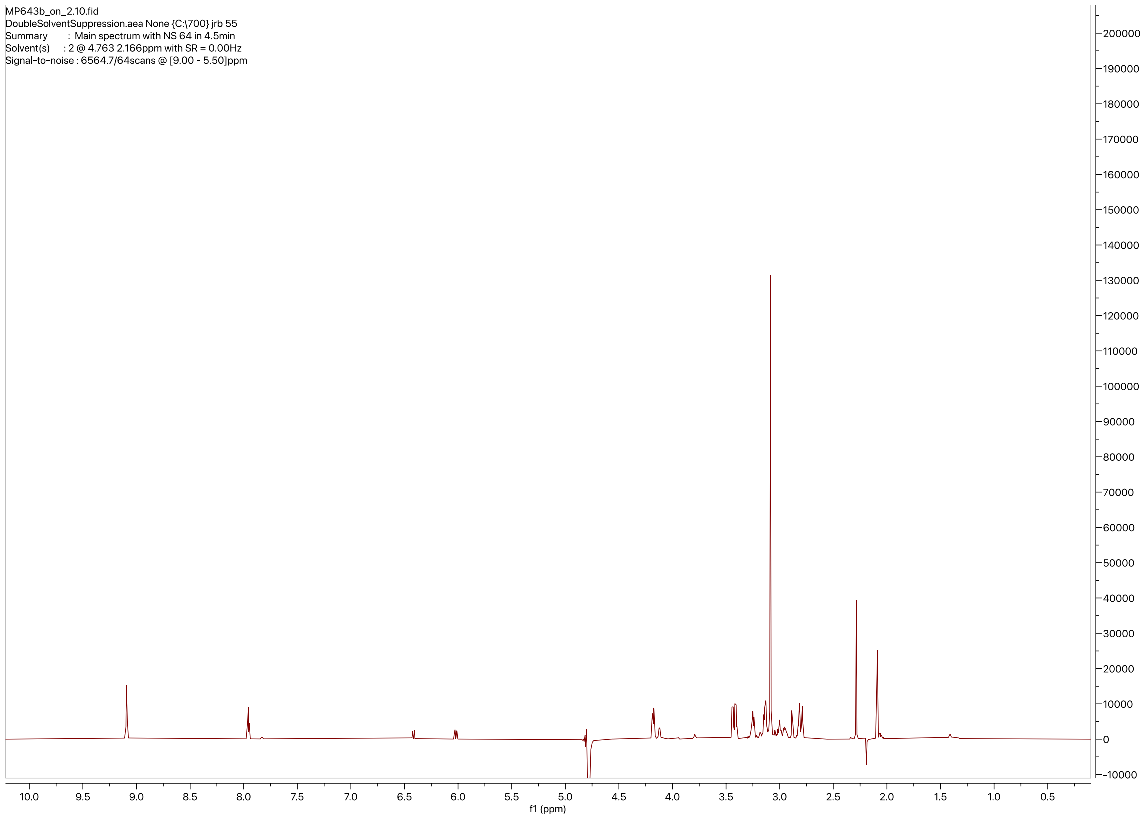

Figure S4; ADTA **2** removal with *N*-methyl maleimide; (a) reaction scheme for maleimide mediated removal of ADTA (b) Water suppression ^{1}H NMR zoomed into the aromatic region showing reformation of the 2-cyano pyrimidine nitrile aromatic proton peaks (9.09 ppm, d and 7.96 ppm, t) (c) ^{13}C NMR showing the loss of the quaternary carbon (87.83 ppm) and reformation of the characteristic nitrile carbon peak (116.27 ppm) (d) Stacked comparison between the EDT maleimide product (green), formed *via* the reaction of EDT (1 eq.) and *N*-methyl maleimide (3 eq.) under identical conditions, and the reaction mixture after 2 h. (e) Full reaction NMR spectra.

DFT general remarks and calculated co-ordinates (Figure S5)

Conformational searching was carried out using Conformer-Rotamer Ensemble Sampling Tool (CREST).[8,9] Geometry optimisations were performed using Gaussian 16[10] at the M06-2X/6-31+G(d,p) level using the SMD[11] solvation model with water as solvent. Geometry optimisation was carried out on all the structures identified by CREST for each compound, and the final structures confirmed as minima by the presence of zero imaginary vibrational frequencies. The coordinates given below are for the conformer of each compound with the lowest Gibbs free energy.

M06-2X/6-31+G(d,p) – 5-membered Acyclic optimised geometry
Sum of electronic and thermal Free Energies = -1232.448505 Hartree / -3235793.55 kJ mol^{-1}

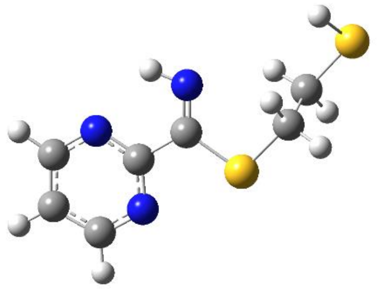

Symbol	X	Y	Z
C	1.771327	0.178937	-0.08098
N	2.28153	-1.03277	0.126555
C	3.601208	-1.09316	0.341227
C	4.398759	0.042692	0.347921
C	3.757785	1.255064	0.120776
N	2.441791	1.331469	-0.09499
H	4.022705	-2.07928	0.511206
H	5.466156	-0.01194	0.522686
H	4.309502	2.190149	0.110567
C	0.288687	0.27719	-0.33145
C	-2.26214	-0.70607	-0.59744
H	-2.24016	0.025523	-1.40839
H	-2.8082	-1.58993	-0.9364
C	-2.89765	-0.12825	0.658073
H	-3.00743	-0.90128	1.422185
H	-2.29312	0.681239	1.069856
H	0.374952	2.136443	-0.53732
N	-0.31271	1.377298	-0.55635
S	-0.56073	-1.27628	-0.3011
H	-4.23344	1.478076	-0.49315
S	-4.59008	0.487565	0.342571

M06-2X/6-31+G(d,p) – 5-membered cyclic optimised geometry

Sum of electronic and thermal Free Energies = -1232.457165 Hartree / -3235816.287 kJ mol^{-1}

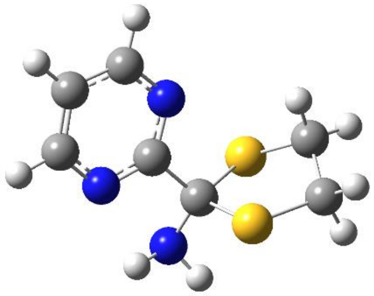

**Symbol**	**X**	**Y**	**Z**
C	-0.94853	0.154282	0.183062
N	-1.15342	-1.16095	0.093175
C	-2.40909	-1.55447	-0.1306
C	-3.45951	-0.65423	-0.2725
C	-3.13205	0.688836	-0.17538
N	-1.87835	1.101244	0.053315
H	-2.57714	-2.6256	-0.20051
H	-4.47481	-0.98464	-0.45346
H	-3.88601	1.463417	-0.28201
C	0.483017	0.651463	0.380146
C	2.270096	-1.38229	-0.01664
C	2.658122	-0.35407	-1.06601
H	3.151641	-1.90446	0.365271
H	1.560779	-2.11042	-0.41505
H	3.501384	0.251117	-0.72723
H	2.915568	-0.83531	-2.01293
N	0.467352	1.907342	1.075389
H	1.386039	2.339228	1.033206
H	-0.21515	2.530801	0.652163
S	1.532412	-0.47905	1.369836
S	1.20611	0.706877	-1.36516

M06-2X/6-31+G(d,p) – 6-membered Acyclic optimised geometry
Sum of electronic and thermal Free Energies = -1271.718972 Hartree / -3338898.161 kJ mol^{-1}

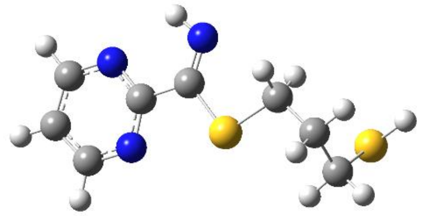

Symbol	X	Y	Z
C	2.121481	0.20796	0.041964
N	2.204696	-1.06882	-0.32541
C	3.414586	-1.63498	-0.23782
C	4.52507	-0.93351	0.210331
C	4.3205	0.39365	0.568632
N	3.1191	0.972574	0.487197
H	3.488615	-2.67633	-0.53583
H	5.501664	-1.39652	0.278351
H	5.137111	1.011021	0.930459
C	0.772891	0.874798	-0.05011
C	-1.9016	1.01983	-0.63867
H	-2.05007	1.345877	0.393343
H	-1.6318	1.889578	-1.24135
C	-3.15946	0.362206	-1.19555
H	-3.94901	1.122289	-1.19999
H	-2.99662	0.069321	-2.23983
C	-3.65266	-0.86776	-0.43807
H	-2.90724	-1.66811	-0.44205
H	-4.55207	-1.26111	-0.91503
H	-4.99293	0.323441	1.140924
S	-0.51213	-0.15723	-0.67811
S	-4.01096	-0.57435	1.332675
N	0.565782	2.08595	0.291314
H	1.449839	2.484642	0.62206

M06-2X/6-31+G(d,p) – 6-membered Cyclic optimised geometry

Sum of electronic and thermal Free Energies = -1271.725038 Hartree / -3338914.087 kJ mol^{-1}

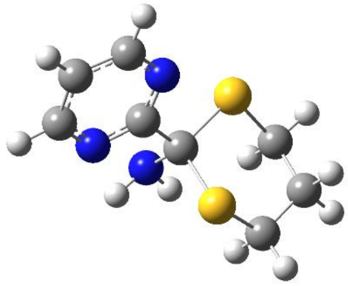

Symbol	X	Y	Z
C	1.260163	0.030843	0.164636
N	1.689469	1.036411	-0.59495
C	3.004879	1.079554	-0.84127
C	3.883825	0.128981	-0.34336
C	3.326383	-0.87889	0.432931
N	2.016661	-0.93502	0.691827
H	3.355917	1.903193	-1.4561
H	4.946422	0.170164	-0.54846
H	3.941047	-1.66517	0.861546
C	-0.23462	-0.08725	0.442091
C	-2.77366	1.081499	0.450458
C	-2.61469	-1.27989	-0.49404
C	-3.27976	0.087293	-0.58781
H	-3.3123	2.028706	0.362139
H	-2.94412	0.710121	1.466725
H	-3.04741	-1.9695	-1.22348
H	-2.76382	-1.72017	0.498075
H	-4.35497	-0.05161	-0.42326
H	-3.14691	0.499288	-1.59382
N	-0.4262	-0.56595	1.78964
H	-1.41152	-0.72291	1.984017
H	0.052938	-1.4586	1.891393
S	-0.83176	-1.27094	-0.87975
S	-1.02511	1.555665	0.231868

DFT Energy Difference Calculations

**5-Mem-Water**	**Hartree**	**kJ/mol**
Acyclic	-1232.448505	-3235794
Cyclic	-1232.457159	-3235816
Difference (Acyclic – Cyclic)	0.008654	23

**6-Mem-Water**	**Hartree**	**kJ/mol**
Acyclic	-1271.718972	-3338898
Cyclic	-1271.725038	-3338914
Difference (Acyclic – Cyclic)	0.006066	16

3. Bioconjugation general remarks

All conjugation experiments were performed in a standard polypropylene Eppendorf safe-lock tubes (1.5 or 2.0 mL) at atmospheric pressure with mixing at the temperature stated. All reagents and solvents were purchased from commercial sources; Sigma UK, Fisher UK or VWR UK and used as per manufacturer instructions. Buffers were prepared with double-deionised water and filter sterilised (0.20 µm). Borate Buffer Saline (BBS) contains 50 mM sodium borate, 50 mM sodium chloride, and 5 mM ethylenediaminetetraacetic acid (EDTA) at pH 8.5 or 7.4. Phosphate Buffer Saline (PBS) contains 10 mM phosphate, 138 mM NaCl, 2.7 mM KCl, pH 7.4. Conjugation Buffer contains 40 mM phosphate, 20 mM NaCl, 6 mM EDTA, pH 7.4. Ultrapure DMF was purchased from Sigma and stored under dry conditions. Ultrafiltration was carried out using Amicon Ultra-4 Centrifugal Filter Units with molecular weight cutoff (MWCO) of 10 kDa or in Vivaspin 500 centrifugal concentrators (Sartorius, UK) with molecular weight cut-off (MWCO) of 10 kDa. Centrifugation was performed using Eppendorf 5415R fixed angle bench rotor operating at 14000 rcf at 20 °C or in an Eppendorf 5810 swing-bucket rotor centrifuge operating at 3220 rcf at 20 °C. Trastuzumab (Ontruzant^{TM}) was purchased from UCLH in its clinical formulation (Samsung Bioepis, lyophilised). Ontruzant Fab was prepared by a sequential enzymatic digest of the full antibody with pepsin and papain, following a literature procedure.[12]

UV-Vis spectroscopy was used to determine protein concentration using NanoDrop One/One Microvolume UV-Vis Spectrophotometer (Thermo Fisher) operating at room temperature. Sample buffer was used as blank for baseline correction with extinction coefficients: ε280 = 68590 M^{-1} cm^{-1} for Fab, ε280 = 215380 $M^{-1}cm^{-1}$ for trastuzumab,[13] ε505 = 74000 M^{-1} cm^{-1} for Azide-fluor 488. A correction factor at 280 nm of 0.178 for Fluorescein, 0.11 for Azide-fluor 488

was employed. Antibody conjugate concentration was determined using the same extinction coefficient as for native trastuzumab Fab (Quinoxaline nitriles were found to have negligible absorbance at 280 nm). Protein conjugation reactions were monitored by 12% glycine-SDS-PAGE with 6% stacking gel under nonreducing conditions. Samples were mixed 1:1 with SDS non-reducing loading buffer (composition for 5X SDS: 10 g (8 mL) of glycerol, 4 mL dH2O, 1.6 mL of 10 % (w/v) SDS, 1 mL of 0.5 M Tris-HCl pH 6.8, 0.025 g Coomassie Brilliant Blue R-250) and heated at 75 °C for 5 min before loaded to the gel. Page Ruler Plus Pre-Stained Protein Ladder (Thermo Scientific) was used. Samples were run at 200 V, 60 min at 1 X running buffer. The 10 X running buffer contained 30 g of Tris base, 144 g of glycine and 10 g of SDS in 1 L of dH2O, with final pH of 8.3. Gels were stained in Coomassie Blue Stain (10% Ammonium Sulfate (100 g), 0.1% Coomassie Brilliant Blue R-250 (500 mg), 3% Phosphoric Acid (30 mL), ethanol (200 mL), and water (1 L). Gels were de-stained with water. The SDS-PAGE gels (Figure S12, 14, 23, 28 and 33), have had lanes cut out of the gel that included data not related to the specific experiments.

LCMS general remarks

For protein analysis, liquid chromatography mass spectrometry (LCMS) was performed at the Department of Chemistry, UCL, on an Agilent 1100/1200 LC system, with a 6510A QTOF mass spectrometer or a 6530A QTOF mass spectrometer. Samples for LCMS analysis were prepared in LCMS grade water using ZebaSpin (7 kDa) as per manufacturer's instructions. All Fab LCMS samples were prepared at 5 μM. For trastuzumab, LCMS samples were prepared at 6.5 μM, and the antibody was deglycosylated with 1 μL PNGase (NEB) for 16 h at 37 ºC prior to LCMS submission. Sodium adducts are commonly found as $[M+23]^+$ in some of the obtained LCMS data. 2 μL of sample was injected through a 100 μL loop into the Agilent PLRPS 1000 Å column (150 mm x 2.1 mm, 8 μM particle size) at 60 ºC. Proteins were separated by gradient elution of mobile phase A (water with 0.1% formic acid) and mobile phase B (acetonitrile with 0.1% formic acid) as per the gradient(s) specified in Table S.1 (Agilent 6530) and Table S.2 (Agilent 6510). The flow rate was 0.8 mL/min (Agilent 6530) or 0.3 mL/min (Agilent 6510), and the 6530/6510 QTOF was prepared in positive mode. Other LCMS parameters include: VCap = 3500 V, gas temperature = 350 ºC, dry gas flow rate = 10 L/min, nebuliser = 30 psi, fragmentor = 380 V, skimmer = 65 V, acquisition rate = 0.5 spectra/s. Data was acquired with a LCMS scan within the m/z range 700-7000. Deconvolution of raw LCMS data to zero charge mass spectra was carried out using the maximum entropy deconvolution algorithm within the Agilent MassHunter software (v B.07.00). The number of compound additions is shown on the LCMS (0, 1, 2, 3... etc). For labelling of protein conjugates: LC refers to light chain, HC refers to heavy chain, HHLL refers to full antibody, and HL refers to half antibody. Deconvolution artefacts include species with masses

corresponding to LC-LC (2 x LC) or HC-HC (2 x HC). Due to ionisation differences between smaller and larger proteins and subsequent ion detection, the HC cannot be observed in some of the obtained LCMS data.

Table S.1 LCMS gradient for protein LCMS analysis on Agilent 6530.

Time (min)	Solvent A (water + 0.1% formic acid) (%)	Solvent B (acetonitrile+ 0.1% formic acid) (%)
0.00	80	20
1.00	80	20
6.50	40	60
7.50	40	60
7.60	80	20
8.50	80	20

Table S.2 LCMS gradient for protein LCMS analysis on Agilent 6510.

Time (min)	Solvent A (water + 0.1% formic acid) (%)	Solvent B (acetonitrile+ 0.1% formic acid) (%)
0.0	85	15
2.0	85	15
3.0	68	32
4.0	68	32
14.0	65	35
18.0	5	95
20.0	5	95
22.0	85	15
25.0	85	15

Native Fab

Fab (20 μL, 150 μM, 4.76 mg/mL) in conjugation buffer was desalted (7 kDa MWCO, ZebaSpin) prior to LCMS analysis. Concentration was determined photometrically using ε_{280}= 68590 M^{-1} cm^{-1}.

(a)

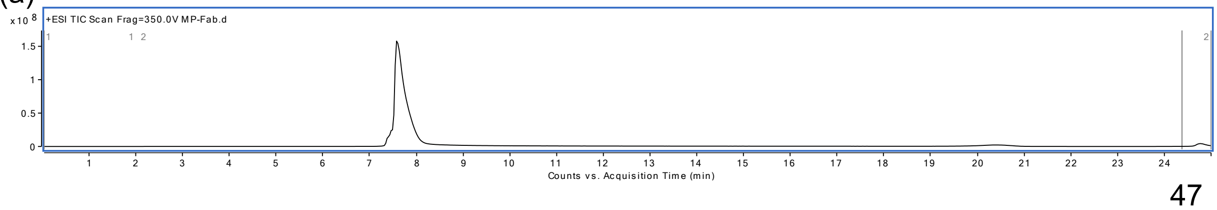

(b)

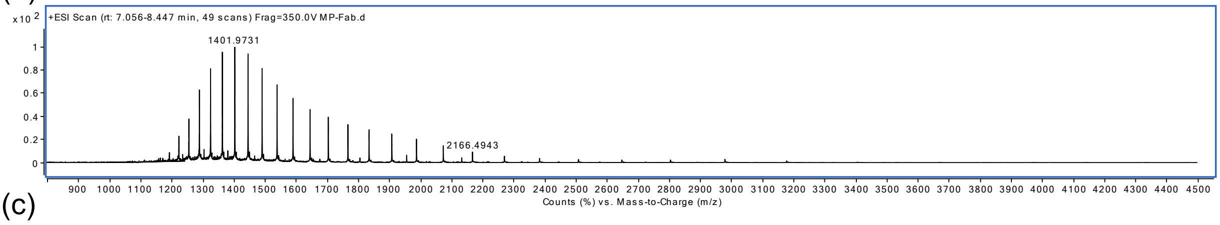

(c)

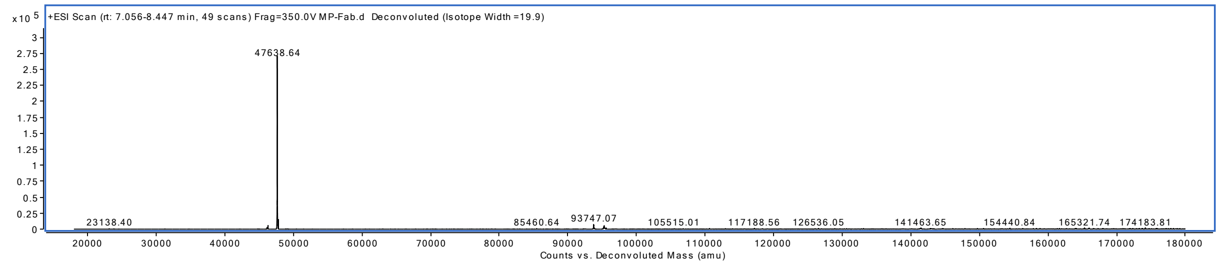

Figure S6: LCMS analysis of conjugate Native Fab; a) TIC, b) non-deconvoluted ion-series, c) full range deconvoluted ion series mass spectrum; native Fab expected 47638, observed 47638.

Reduced Fab

Fab (20 µL, 150 µM, 4.76 mg/mL) in conjugation buffer was reduced with tris(2-carboxyethyl)phosphine (TCEP) (2 µL, 15 mM in dH_2O, 10 eq.) The mixture was incubated at 37 °C for 1.5 h, 300 rpm. Lastly, sample was desalted (7 kDa MWCO, ZebaSpin) prior to LCMS analysis. Concentration was determined photometrically using ε_{280}= 68590 M^{-1} cm^{-1} .

(a)

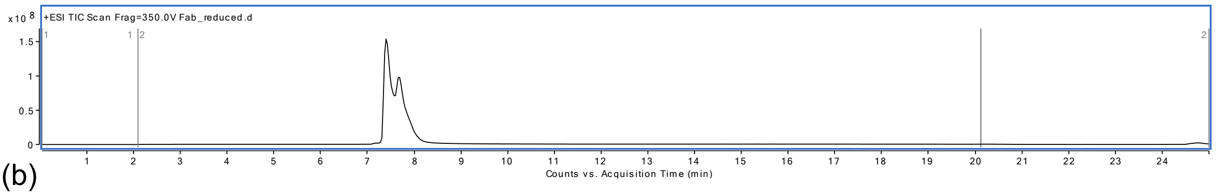

(b)

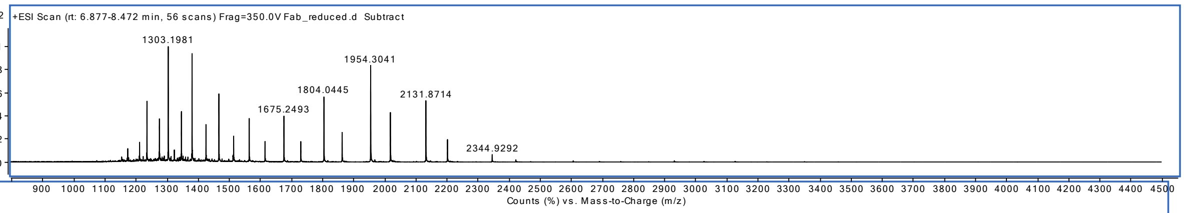

(c)

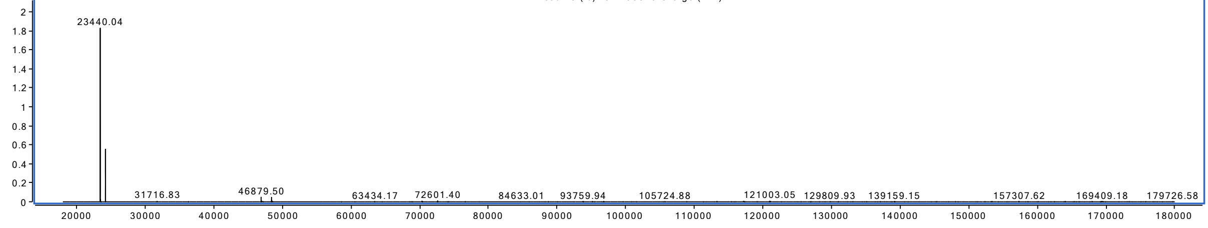

(d)

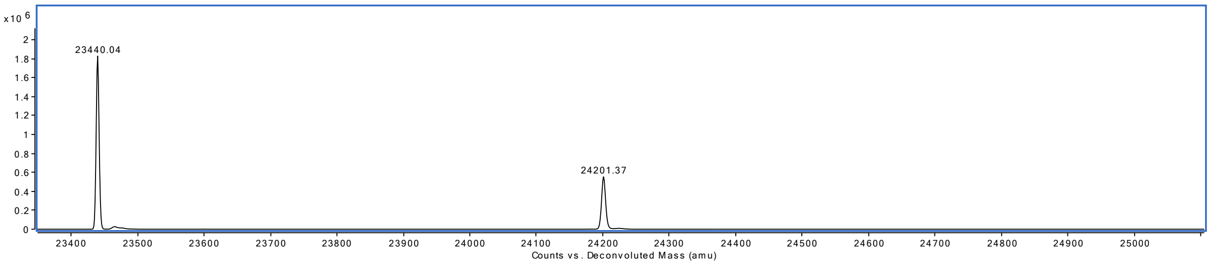

Figure S7: LCMS analysis of Reduced Fab; a) TIC, b) non-deconvoluted ion-series, c) full range deconvoluted ion series mass spectrum, d) zoomed in deconvoluted ion series mass spectrum; Expected: native light chain (LC) 23438, native heavy chain (HC) 24200, Observed: 23440, 24201.

Trastuzumab

Trastuzumab full antibody (80 µL, 25 µM, 5.8 mg/mL) in conjugation buffer was buffer swapped into 50 mM ammonium acetate pH 6.9 (7 kDa MWCO, ZebaSpin) and adjusted to 5 µM with ammonium acetate (pH 6.9). To this (40 µL) was added 0.67 µL of PNGase F and the reaction was left at 37 °C for 16 h. Concentration was determined photometrically using ε_{280}=215380 M^{-1} cm^{-1}.

(a)

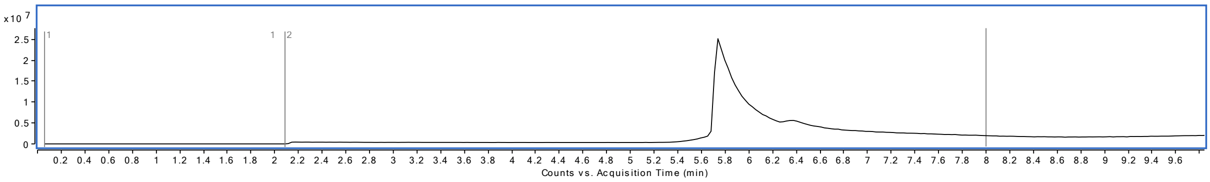

(b)

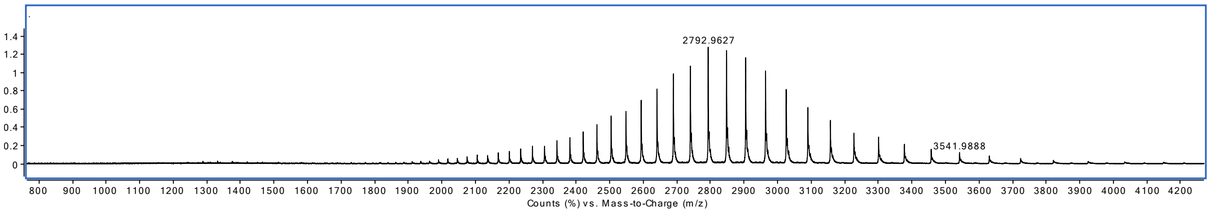

(c)

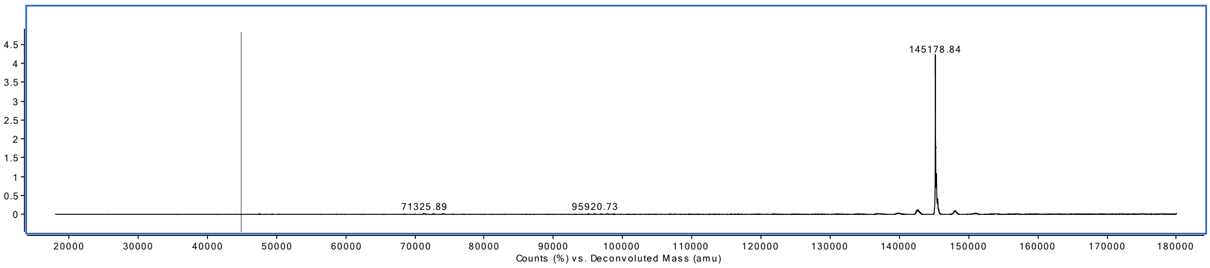

(d)

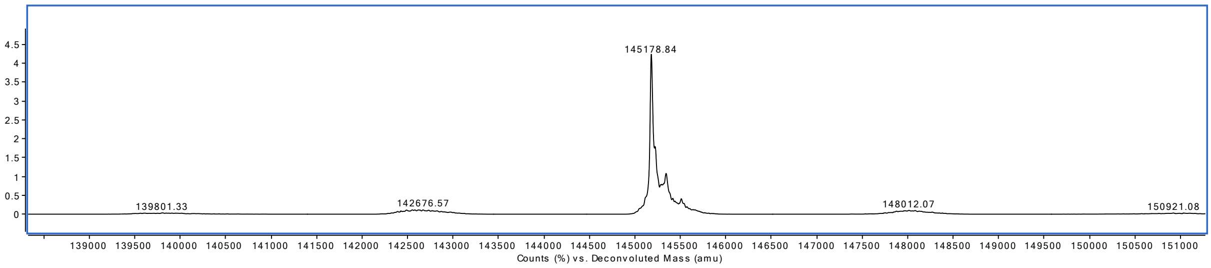

Figure S8: LCMS analysis of Trastuzumab; a) TIC, b) non-deconvoluted ion-series, c) full range deconvoluted ion series mass spectrum, d) zoomed in deconvoluted ion series mass spectrum; native full antibody expected 145177, observed 145178.

Reduced Trastuzumab

Trastuzumab full antibody (80 µL, 25 µM, 5.8 mg/mL) in conjugation buffer was reduced with tris(2-carboxyethyl)phosphine (TCEP) (1.3 µL, 15 mM in diH$_2$O, 10 eq.). The mixture was incubated at 37 °C for 2 h, 300 rpm. The excess reagent was removed *via* ultrafiltration (10 kDa MWCO) into 50 mM ammonium acetate pH 6.9 (7 kDa MWCO, ZebaSpin) and adjusted to 5 µM with ammonium acetate (pH 6.9). To this (40 µL) was added 0.67 µL of PNGase F and the reaction was left at 37 °C for 16 h. Concentration was determined photometrically using ε_{280}=215380 M^{-1} cm^{-1}.

(a)

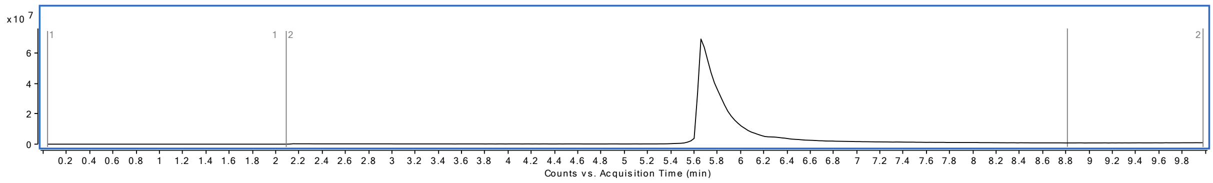

(b)

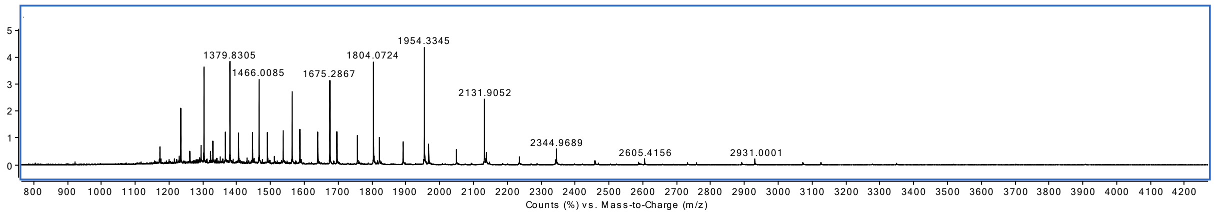

(c)

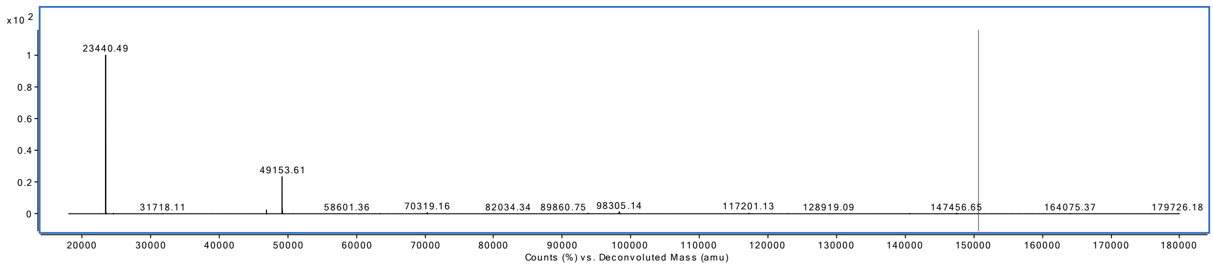

(d)

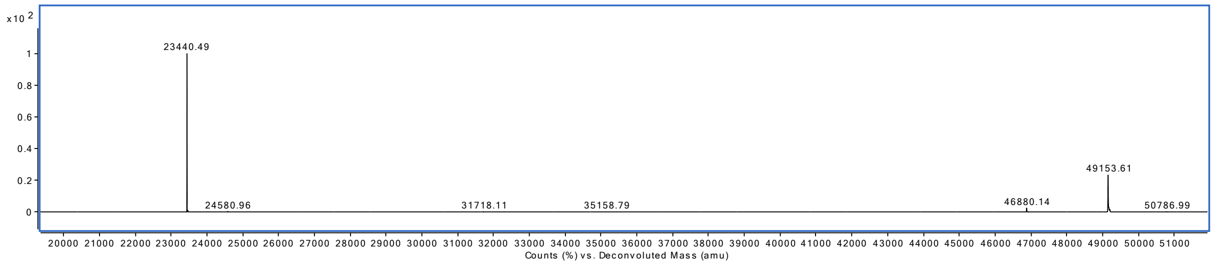

Figure S9: LCMS analysis of Reduced Trastuzumab; a) TIC, b) non-deconvoluted ion-series, c) full range deconvoluted ion series mass spectrum, d) zoomed in deconvoluted ion series mass spectrum; Expected mass of reduced trastuzumab: LC 23439, HL 49153. Observed: LC 23440, HL 49153.

CLT reaction of Fab with 2-cyanopyrimidine (Low equivalents)

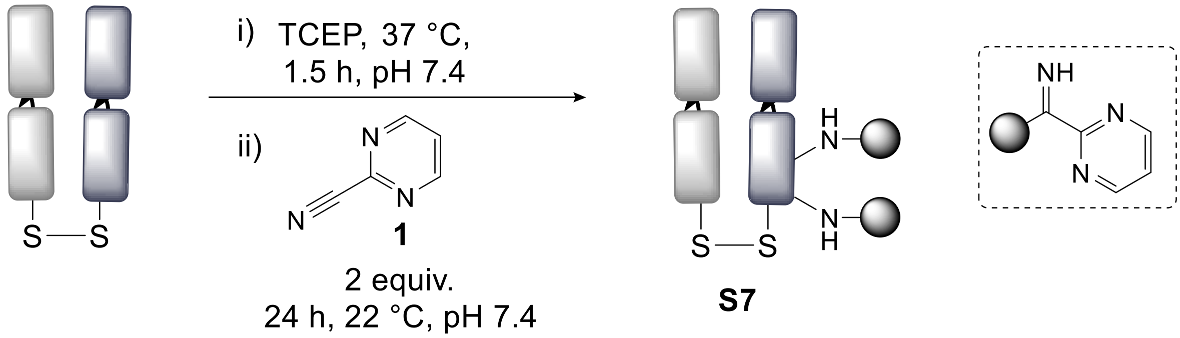

Fab (20 µL, 150 µM, 4.76 mg/mL) in conjugation buffer was reduced with tris(2-carboxyethyl)phosphine (TCEP) (2.0 µL, 15 mM in diH_2O, 10 eq.) The mixture was incubated at 37 °C for 1.5 h, 300 rpm. 2-Cyanopyrimidine 1 was then added (0.4 µL, 15 mM in DMF, 2 eq.) was added and incubated at 22 °C for 24 h. Lastly, sample was desalted into HPLC grade water (7 kDa MWCO, ZebaSpin) prior to LCMS analysis. Concentration was determined photometrically using ε_{280}= 68590 M^{-1} cm^{-1} .

(a)

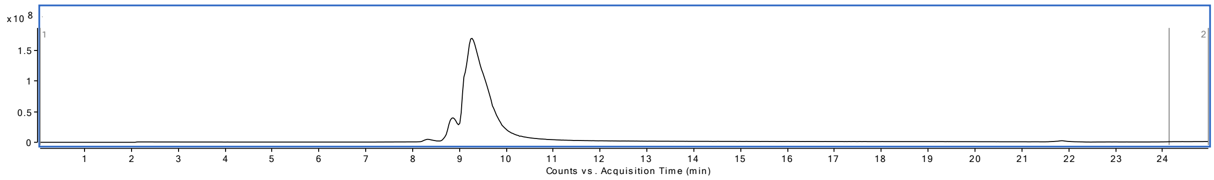

(b)

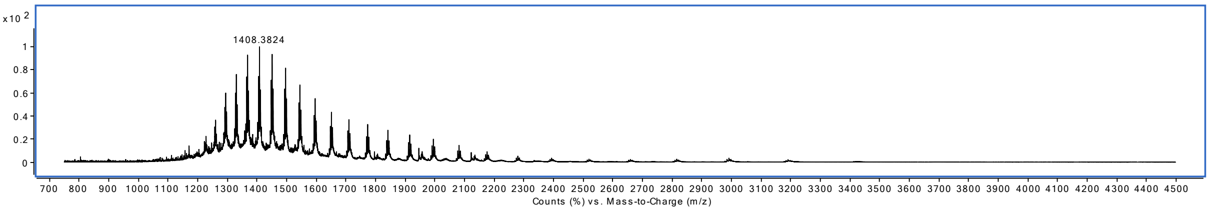

(c)

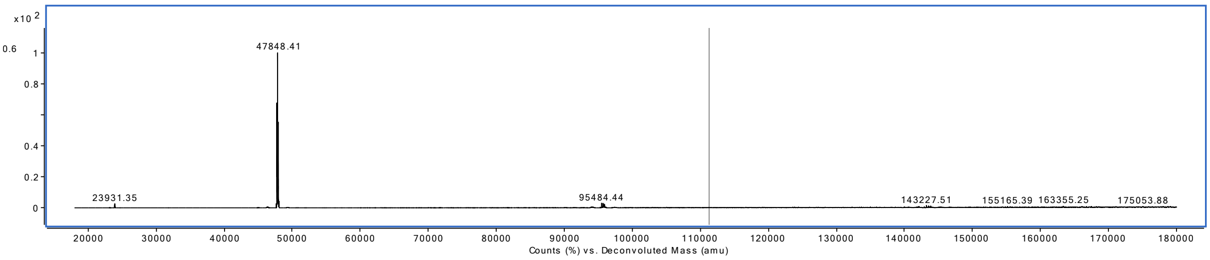

(d)

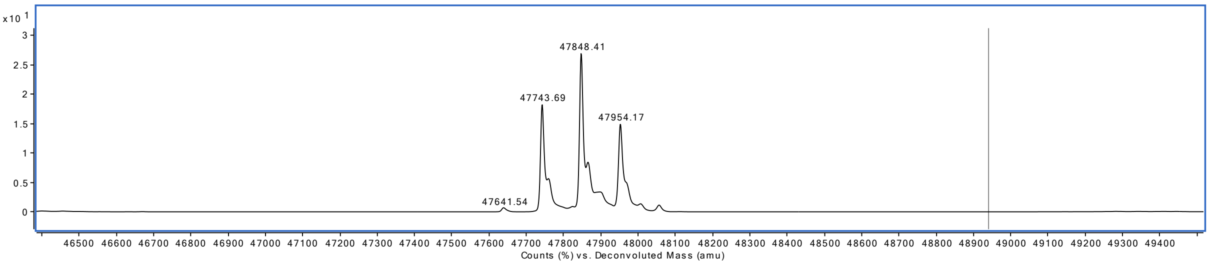

Figure S10: LCMS analysis of conjugate **S7**; a) TIC, b) non-deconvoluted ion-series, c) full range deconvoluted ion series mass spectrum, d) zoomed in deconvoluted ion series mass spectrum; Expected mass of pyrimidine CLT conjugate: 47638 (native, 0 additions), 47743 (1 addition), 47848 (2 additions), 47953 (3 additions). Observed: 47641, 47743, 47848, 47954.

CLT reaction of Fab with 2-cyanopyrimidine (High equivalents)

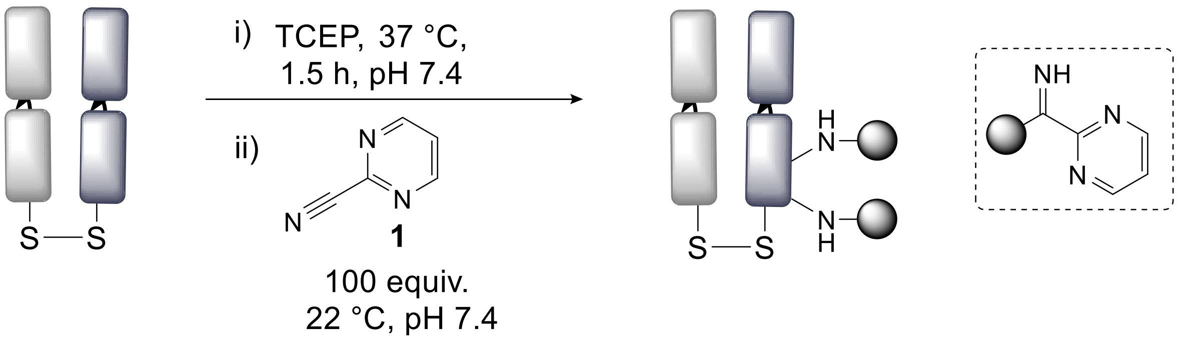

Fab (20 µL, 150 µM, 7.15 mg/mL) in conjugation buffer was reduced with tris(2-carboxyethyl)phosphine (TCEP) (2.0 µL, 15 mM in dH_2O, 10 equiv.) The mixture was incubated at 37 °C for 1.5 h, 300 rpm. 2-Cyanopyrimidine was then added (2 µL, 150 mM in DMF, 100 equiv.) and the reaction was incubated at 22 °C. Following this DTNB (1.0 µL, 150

mM in EtOH, 50 equiv.) was added and the reaction was left at 22 °C for 10 min. Lastly, sample was desalted into HPLC grade water (7 kDa MWCO, ZebaSpin) prior to LCMS analysis. Concentration was determined photometrically using ε_{280}= 68590 M^{-1} cm^{-1}.

15 min

(a)

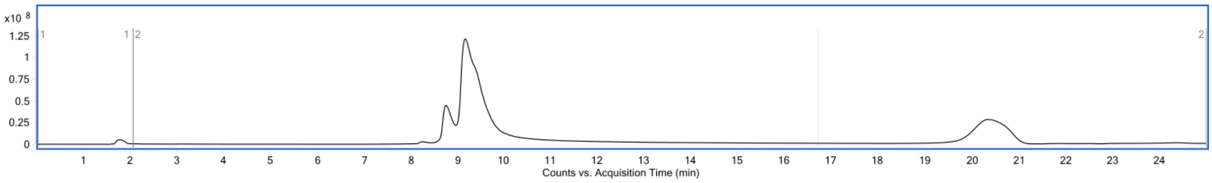

(b)

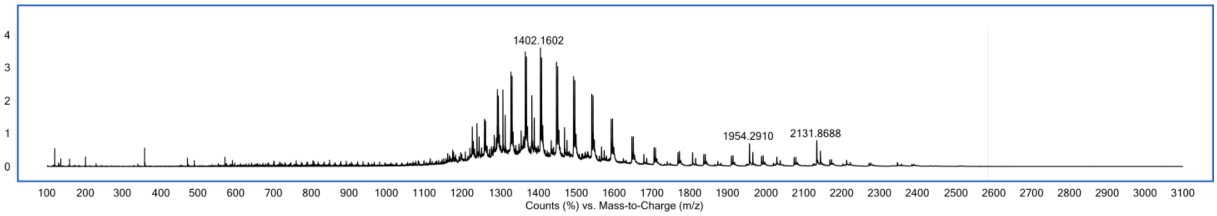

(c)

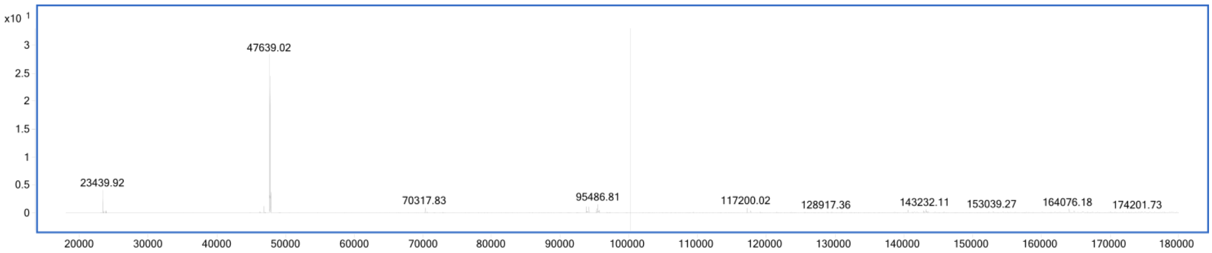

(d)

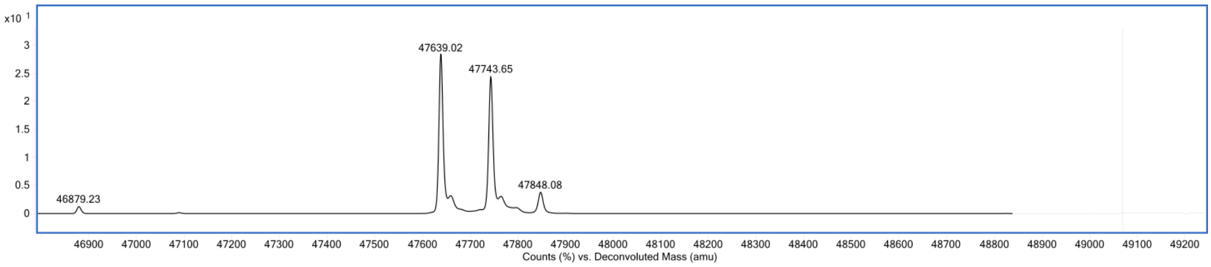

Figure S11a: LCMS analysis of Fab conjugate; a) TIC, b) non-deconvoluted ion-series, c) full range deconvoluted ion series mass spectrum, d) zoomed in deconvoluted ion series mass spectrum; Expected mass of pyrimidine CLT conjugate: 47639 Da (native, 0 additions), 47743 Da (1 addition), 47848 Da (2 additions). Observed: 47639 Da, 47744 Da, 47848 Da.

30 min

(a)

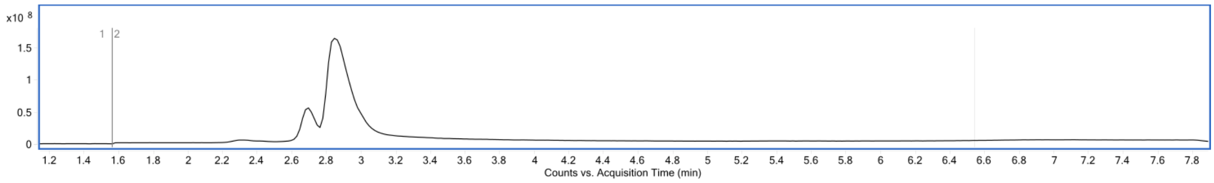

(b)

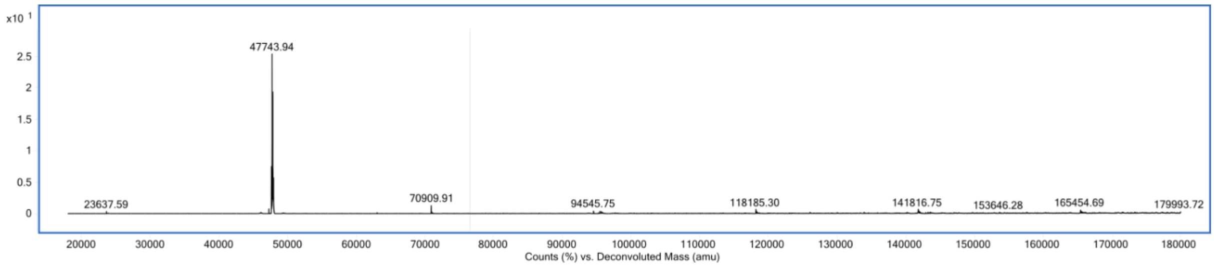

(c)

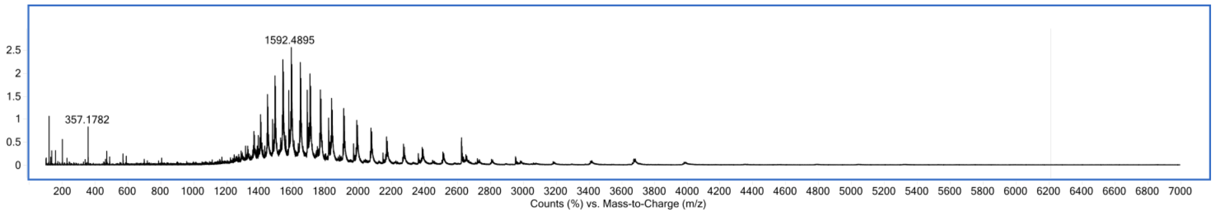

(d)

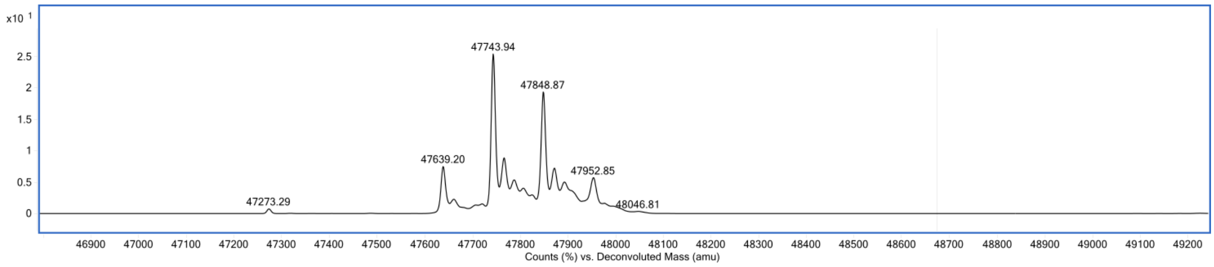

Figure S11b: LCMS analysis of Fab conjugate; a) TIC, b) non-deconvoluted ion-series, c) full range deconvoluted ion series mass spectrum, d) zoomed in deconvoluted ion series mass spectrum; Expected mass of pyrimidine CLT conjugate: 47639 Da (native, 0 additions), 47743 Da (1 addition), 47848 Da (2 additions), 47953 Da (3 additions). Observed: 47639 Da, 47744 Da, 47849 Da, 47953 Da.

60 min

(a)

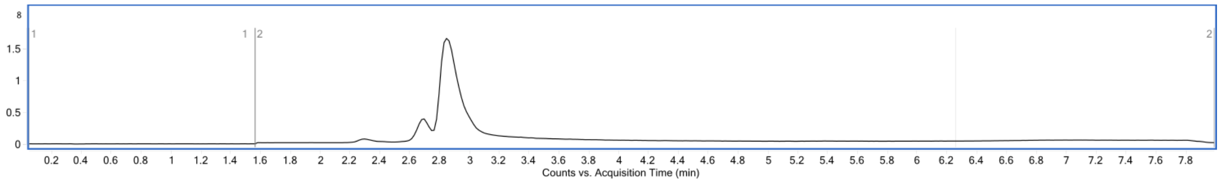

(b)

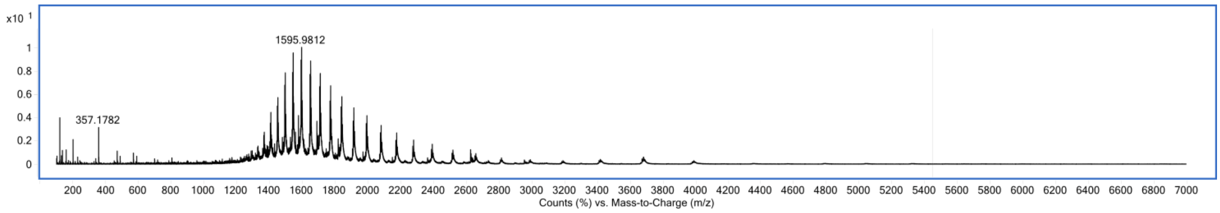

(c)

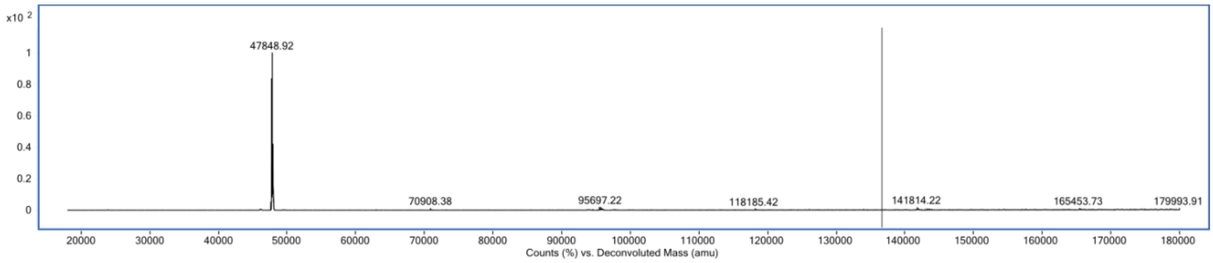

(d)

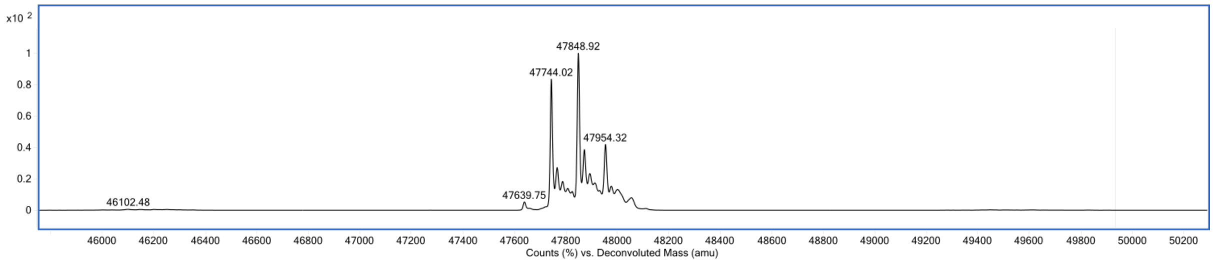

Figure S11c: LCMS analysis of Fab conjugate a) TIC, b) non-deconvoluted ion-series, c) full range deconvoluted ion series mass spectrum, d) zoomed in deconvoluted ion series mass spectrum; Expected mass of pyrimidine CLT conjugate: 47639 Da (native, 0 additions), 47743 Da (1 addition), 47848 Da (2 additions), 47953 Da (3 additions). Observed: 47639 Da, 47744 Da, 47849 Da, 47954 Da.

Average DAR = 1.8

CLT reaction of Fab with 2-cyanopyrimidine (Reduce and cap)

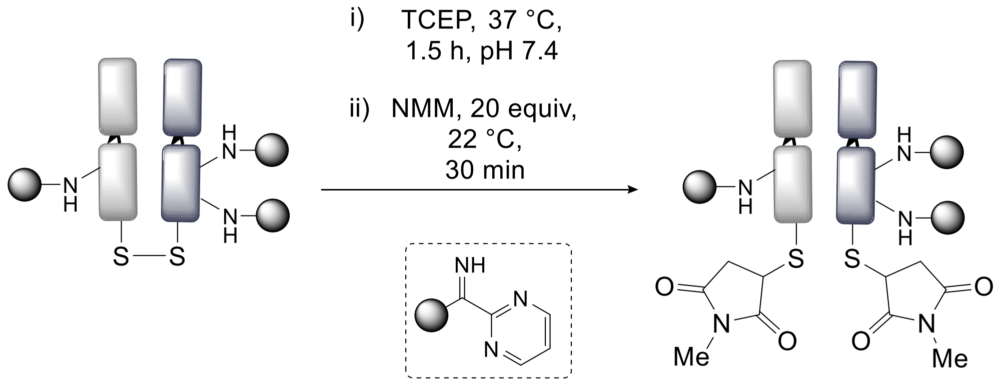

Fab conjugate **S7** was buffer exchanged to conjugation buffer via ultrafiltration (10 kDa MWCO). To this solution was added tris(2-carboxyethyl)phosphine (TCEP) (15 mM in diH_2O, 10 eq.) The mixture was incubated at 37 °C for 1.5 h, 300 rpm. Following this NMM (20 mM in DMF, 20 eq.) was added, and the reaction was left at RT for 30 min. Excess reagent was then removed *via* ultrafiltration (10 kDa MWCO, VivaSpin 500) into HPLC grade water prior to LCMS analysis. Concentration was determined photometrically using ε_{280}= 68590 M^{-1} cm^{-1}.

(a)

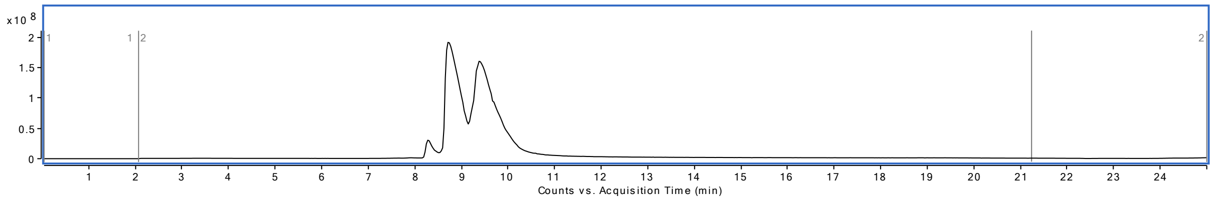

(b)

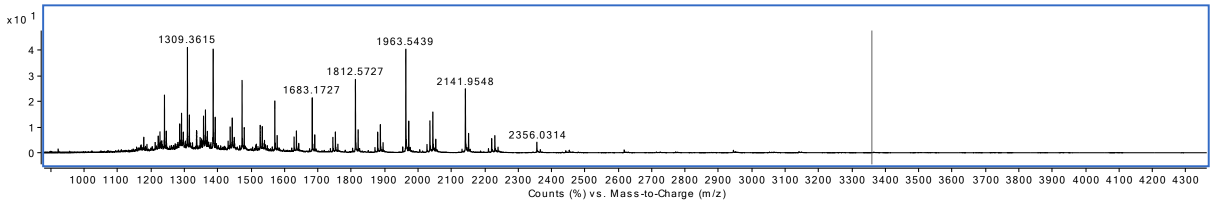

(c)

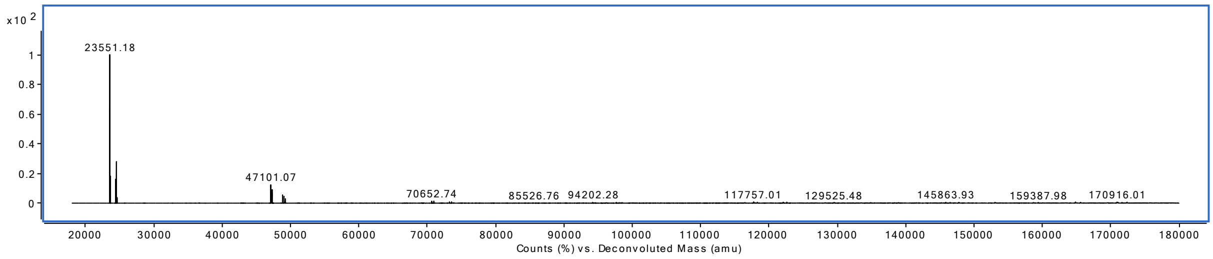

(d)

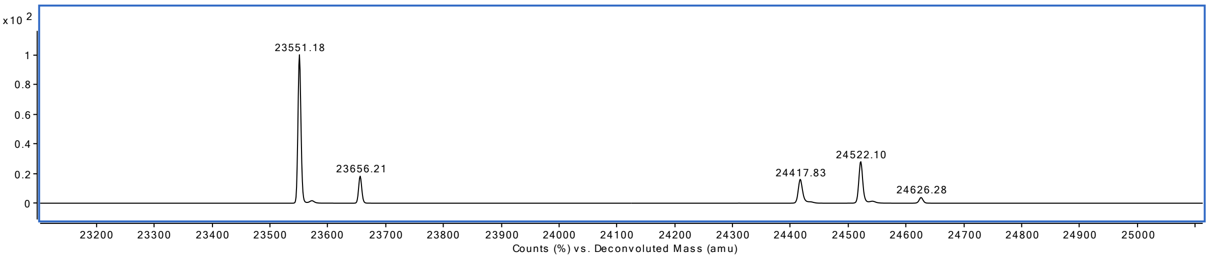

Figure S12: LCMS analysis of reduced and capped conjugate **S7b**; a) TIC, b) non-deconvoluted ion-series, c) full range deconvoluted ion series mass spectrum, d) zoomed in deconvoluted ion series mass spectrum; Expected mass of capped pyrimidine CLT conjugate: 23550 (NMM capped LC with 0 additions), 23665 (NMM capped LC with 1 addition); 24416 (NMM capped HC with 1 addition), 24521 (NMM capped HC with 2 additions), 24626 (NMM capped HC with 3 additions). Observed: LC 23551, 23656; HC 24418, 24522, 24626.

CLT reaction of Fab with *N*-(2-(2-(2-(2-azidoethoxy)ethoxy)ethoxy)ethyl)-2-cyanopyrimidine-5-carboxamide

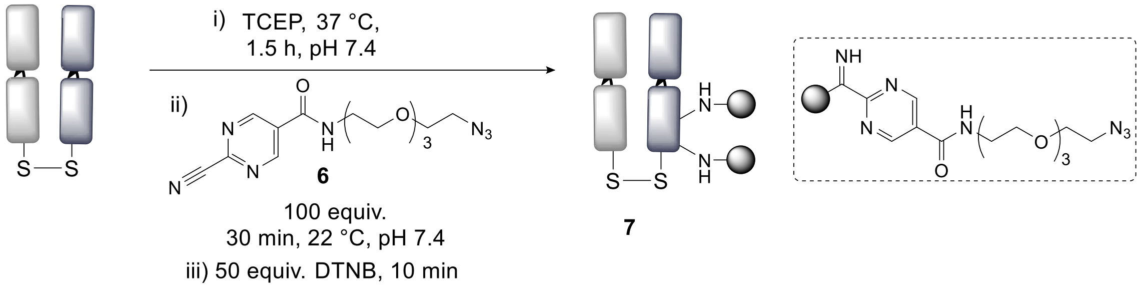

Fab (20 µL, 150 µM, 4.76 mg/mL) in conjugation buffer was reduced with tris(2-carboxyethyl)phosphine (TCEP) (2.0 µL, 15 mM in diH_2O, 10 eq.) The mixture was incubated at 37 °C for 1.5 h, 300 rpm. *N*-(2-(2-(2-(2-azidoethoxy)ethoxy)ethoxy)ethyl)-2-cyanopyrimidine-5-carboxamide **6** (2.0 µL, 150 mM in DMF, 100 eq.) was added and incubated at 22 °C for 30 min. Following this DTNB (1.0 µL, 150 mM in EtOH, 50 eq.) was added and the reaction was left at 22 °C for 10 min. Excess reagent was then removed *via* ultrafiltration (10 kDa MWCO, VivaSpin 500) into HPLC grade water prior to LCMS analysis. Concentration was determined photometrically using ε_{280}= 68590 M^{-1} cm^{-1}.

(a)

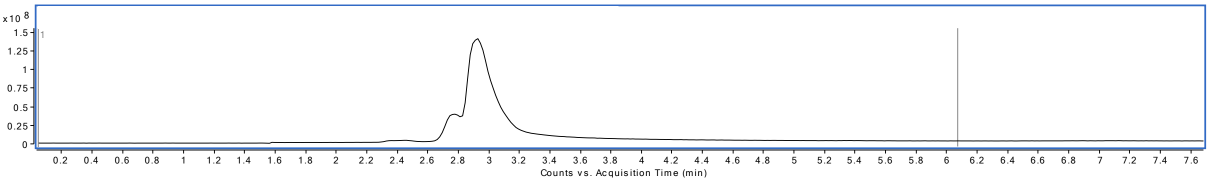

(b)

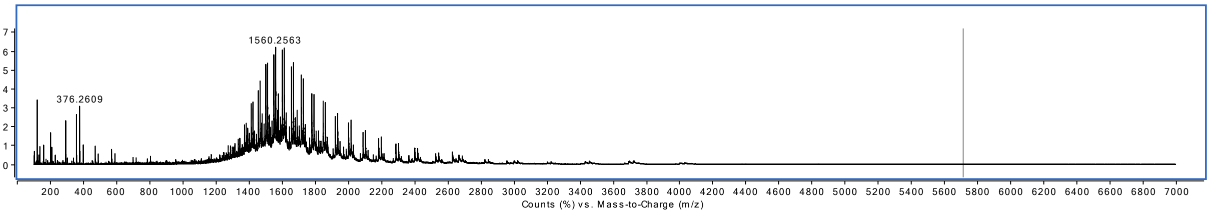

(c)

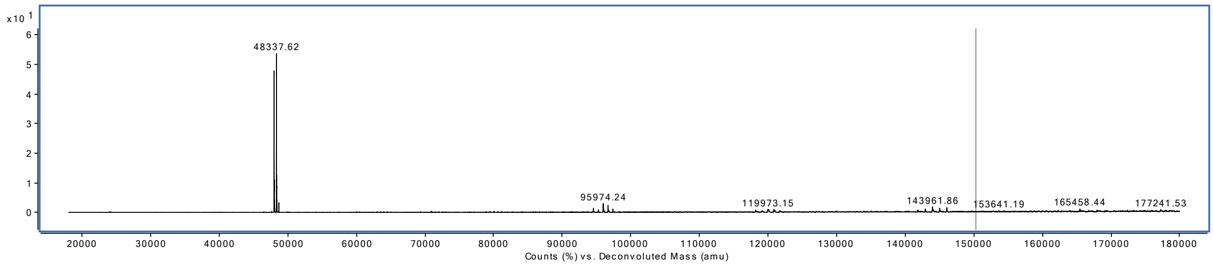

(d)

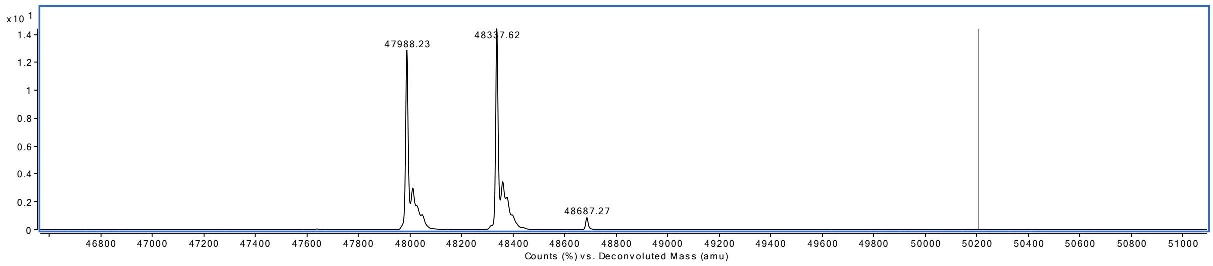

Figure S13: LCMS analysis of conjugate **7**; a) TIC, b) non-deconvoluted ion-series, c) full range deconvoluted ion series mass spectrum, d) zoomed in deconvoluted ion series mass spectrum; Expected mass of CLT conjugate: 47638 (native, 0 additions), 47987 (1 addition), 48336 (2 additions), 48686 (3 additions). Observed:47988, 48337, 48687.

Average DAR = 1.6

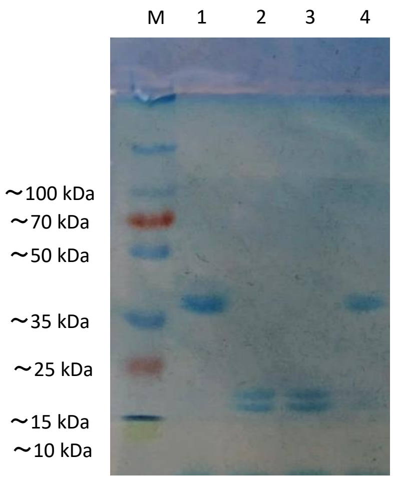

Figure S14: SDS-PAGE of cysteine to lysine transfer (CLT) on trastuzumab Fab; M – molecular marker, 1 – Native Fab, 2 – Reduced Fab, 3 – CLT step 1, transfer, 4 – CLT step 2, reoxidation.

SPAAC functionalisation of Fab conjugate **7**

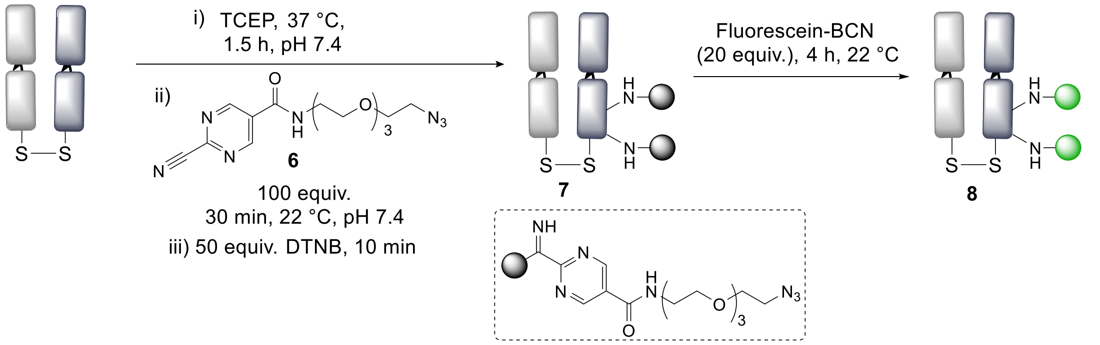

Fab (20 µL, 150 µM, 4.76 mg/mL) in conjugation buffer was reduced with tris(2-carboxyethyl)phosphine (TCEP) (2.0 µL, 15 mM in diH_2O, 10 eq.) The mixture was incubated at 37 °C for 1.5 h, 300 rpm. *N*-(2-(2-(2-(2-azidoethoxy)ethoxy)ethoxy)ethyl)-2-cyanopyrimidine-5-carboxamide **6** (2.0 µL, 150 mM in DMF, 100 eq.) was added and incubated at 22 °C for 30 min. Following this DTNB (1.0 µL, 150 mM in EtOH, 50 eq.) was added and the reaction was left at RT for 10 min. Excess reagent was then removed *via* ultrafiltration (10 kDa MWCO, VivaSpin 500) into PBS (pH = 7.4). To conjugate 7 was added fluorescein-PEG$_3$-BCN (20 mM in DMF, 20 eq.), this was then left in the dark at 22 °C for 4 h, 300 rpm. Excess reagent was then removed by ultrafiltration (10 kDa MWCO) into HPLC grade water prior to LCMS analysis. Concentration was determined photometrically using ε_{280}= 68590 M^{-1} cm^{-1}.

(a)

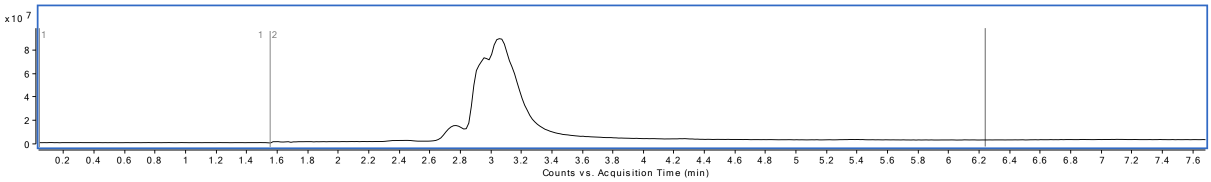

(b)

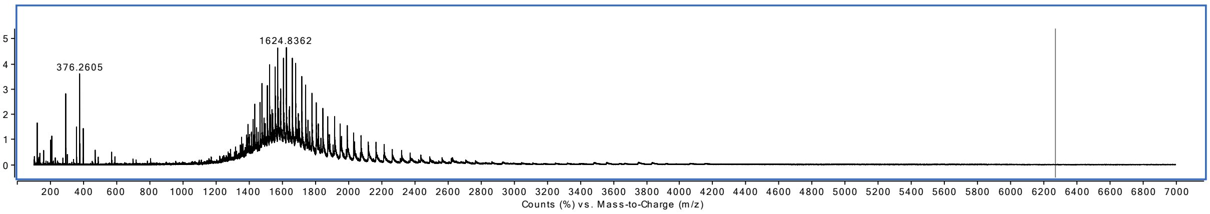

(c)

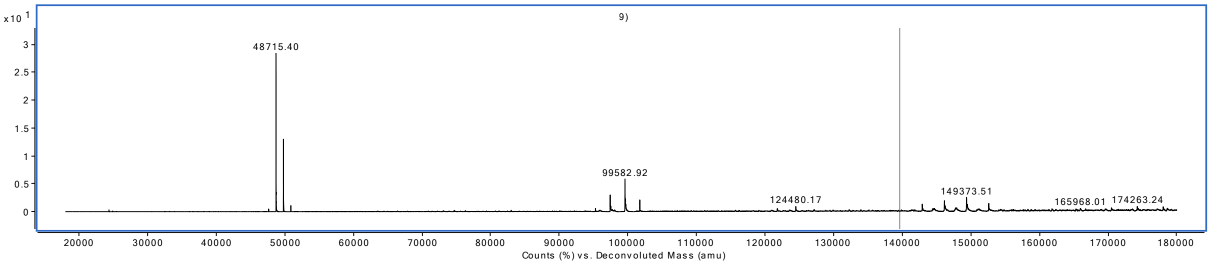

(d)

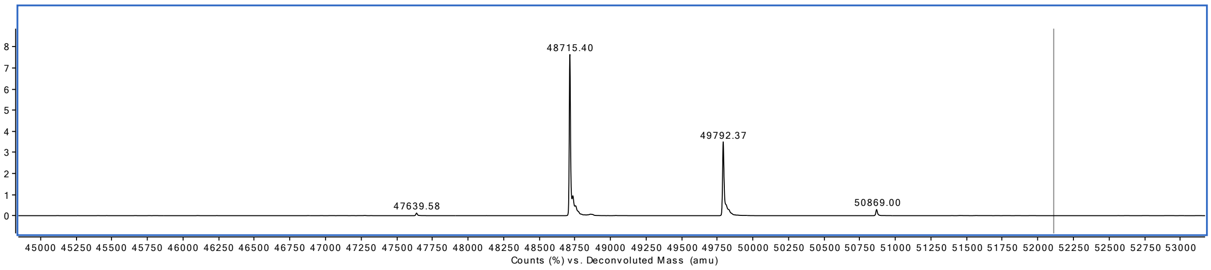

Figure S15: LCMS analysis of conjugate **8**; a) TIC, b) non-deconvoluted ion-series, c) full range deconvoluted ion series mass spectrum, d) zoomed in deconvoluted ion series mass spectrum; Expected mass of CLT-SPAAC conjugate: 47638 (native, 0 additions), 48714 (1 addition), 49790 (2 additions), 50866 (3 additions). Observed: 47639, 48715, 49792, 50869. The fluorophore-to-antibody ratio (FAR) was determined photometrically by obtaining the

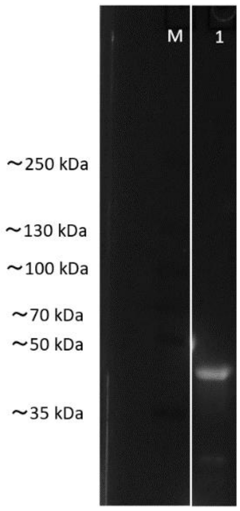

UV/Vis absorption reading of the conjugate **8** and using the following formula, where *Cf* is the correction factor for the absorbance of the fluorescein-PEG$_3$-BCN at 280 nm:

$$FAR = \frac{\frac{Abs_{505}}{\varepsilon_{505}}}{\frac{Abs_{280} - (Cf\ x\ Abs_{505})}{\varepsilon_{280}}} = \frac{\frac{0.434}{83000}}{\frac{0.320 - (0.178\ x\ 0.434)}{68590}} = 1.5$$

Figure S16: SDS-PAGE analysis of conjugate **8** (CLT); M – molecular marker, 1 – Conjugate **8,** The fluorescent bands were visualised using an AZURE 200® (Epi Blue).

Reaction of Fab with pyrazine-2,3-dicarbonitrile

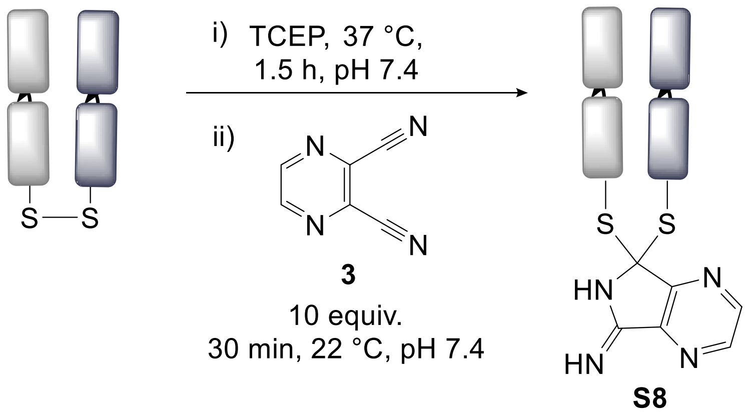

Fab (20 µL, 150 µM, 4.76 mg/mL) in conjugation buffer was reduced with tris(2-carboxyethyl)phosphine (TCEP) (2.0 µL, 15 mM in diH$_2$O, 10 eq.) The mixture was incubated at 37 °C for 1.5 h, 300 rpm. Pyrazine-2,3-dicarbonitrile **3** (2.0 µL, 15 mM in DMF, 10 eq.) was added and incubated at 22 °C for 30 min. Lastly, excess reagent was removed, and the sample was desalted (7 kDa MWCO, ZebaSpin) prior to LCMS analysis. Concentration was determined photometrically using ε280= 68590 M^{-1}cm^{-1}.

(a)

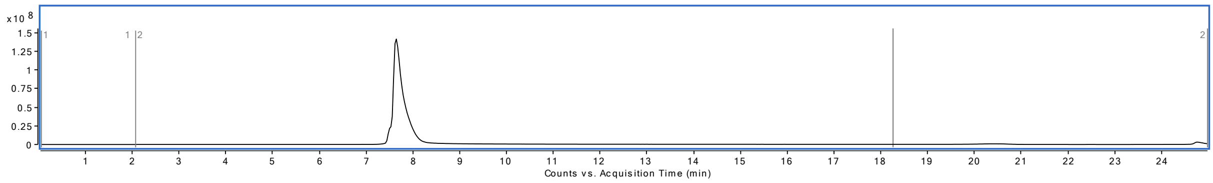

(b)

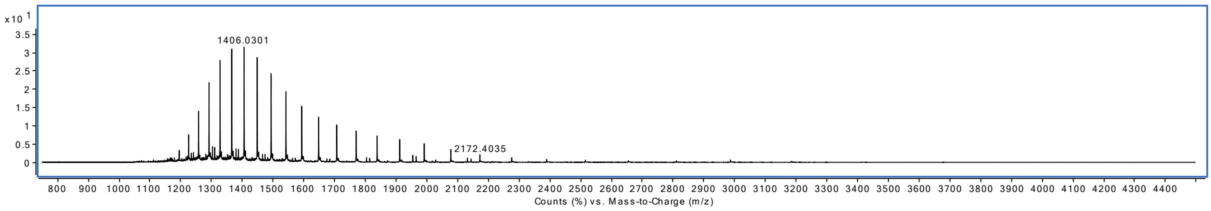

(c)

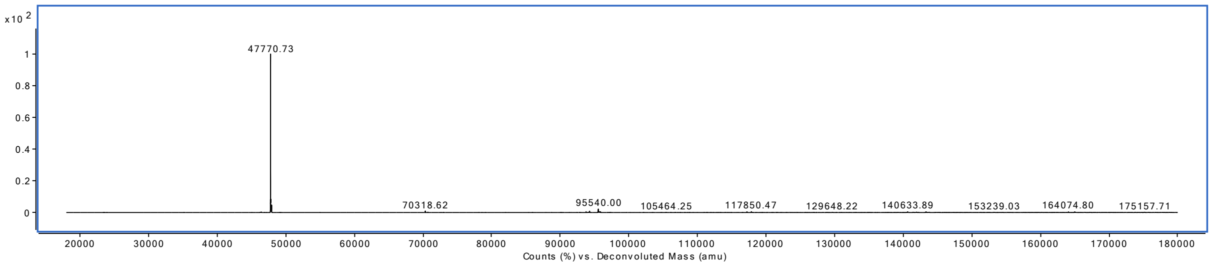

Figure S17: LCMS analysis of conjugate **S8**; a) TIC, b) non-deconvoluted ion-series, c) full range deconvoluted ion series mass spectrum; Expected mass of re-bridged Fab with pyrazine-2,3-dicarbonitrile: 47768, Observed: 47770.

Reaction of Fab with 5,6-bis((methyl(prop-2-yn-1-yl)amino)methyl)pyrazine-2,3-dicarbonitrile

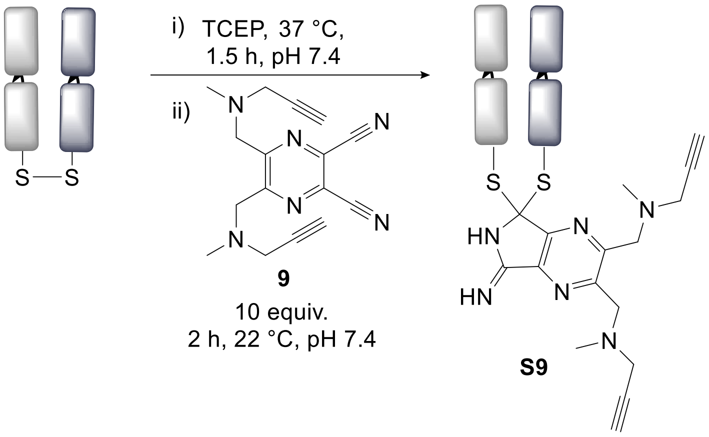

Fab (20 µL, 150 µM, 4.76 mg/mL) in conjugation buffer was reduced with tris(2-carboxyethyl)phosphine (TCEP) (2.0 µL, 15 mM in diH_2O, 10 eq.) The mixture was incubated at 37 °C for 1.5 h, 300 rpm. 5,6-Bis((methyl(prop-2-yn-1-yl)amino)methyl)pyrazine-2,3-dicarbonitrile **9** (2.0 µL, 15 mM in DMF, 10 eq.) was added and incubated at 22 °C for 2 h. Lastly, excess reagent was removed, and the sample was desalted (7 kDa MWCO, ZebaSpin) prior to LCMS analysis. Concentration was determined photometrically using ε280= 68590 $M^{-1}cm^{-1}$.

(a)

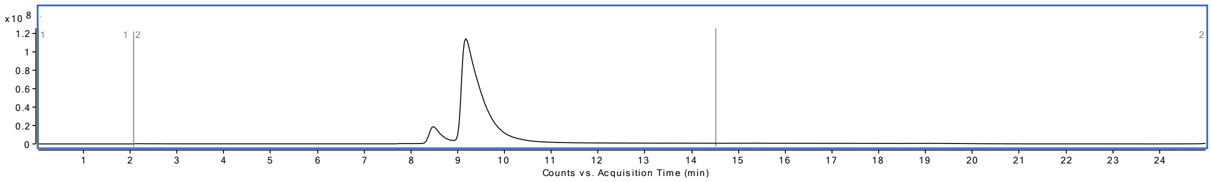

(b)

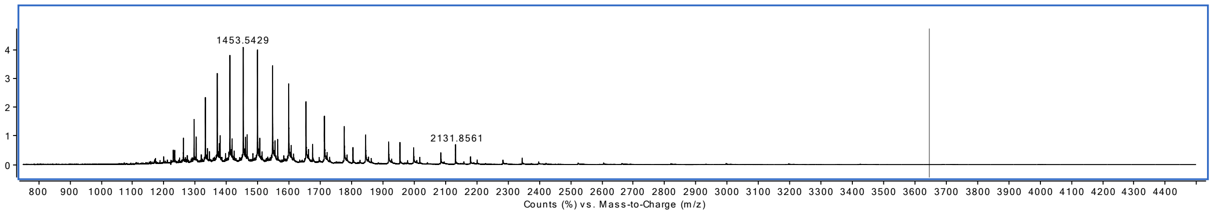

(c)

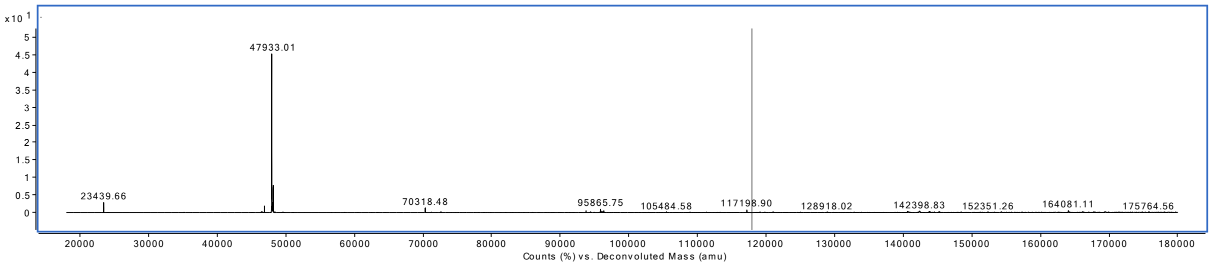

Figure S18: LCMS analysis of conjugate **S9**; a) TIC, b) non-deconvoluted ion-series, c) full range deconvoluted ion series mass spectrum; Expected mass of re-bridged Fab with 5,6-bis((methyl(prop-2-yn-1-yl)amino)methyl)pyrazine-2,3-dicarbonitrile:47930, Observed:47933.

Reaction of Fab with 5,6-bis((methyl(prop-2-yn-1-yl)amino)methyl)pyrazine-2,3-dicarbonitrile with CuAAC attempt

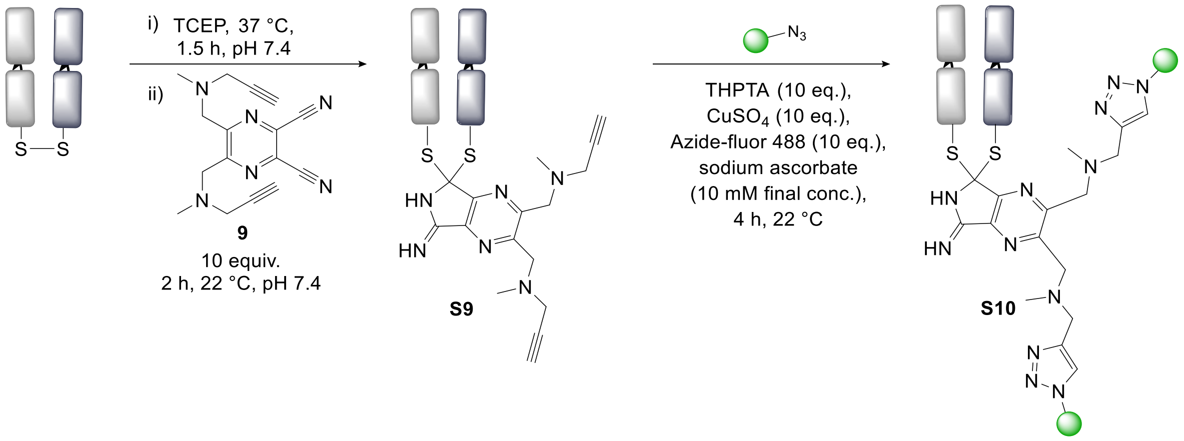

Fab (20 µL, 150 µM, 4.76 mg/mL) in conjugation buffer was reduced with tris(2-carboxyethyl)phosphine (TCEP) (2.0 µL, 15 mM in diH_2O, 10 eq.) The mixture was incubated at 37 °C for 1.5 h, 300 rpm. 5,6-Bis((methyl(prop-2-yn-1-yl)amino)methyl)pyrazine-2,3-

dicarbonitrile **9** (2.0 µL, 15 mM in DMF, 10 eq.) was added and incubated at 22 °C for 2 h. Excess reagent was removed *via* ultrafiltration (7 kDa MWCO, ZebaSpin) into PB (pH 7.0). To conjugate S9 was added THPTA (100 mM in diH_2O, 10 eq.), $CuSO_4$ (20 mM in diH_2O, 10 eq.), Azide-fluor 488 (10 mM in DMF, 10 eq.), and sodium ascorbate (100 mM in diH_2O, final conc. 10 mM), the reaction was left at 22 °C, 300 rpm for 4 h. The excess reagent was then removed using a desalting column (PD Minitrap G-25, GE Healthcare) followed by ultrafiltration (10 kDa MWCO) into conjugate buffer to concentrate the sample. Lastly, the sample was desalted into HPLC grade water (7 kDa MWCO, ZebaSpin) prior to LCMS analysis. Concentration was determined photometrically using ε280= 68590 $M^{-1}cm^{-1}$.

(a)

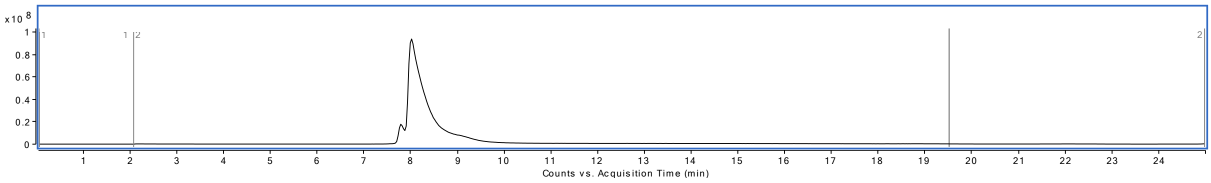

(b)

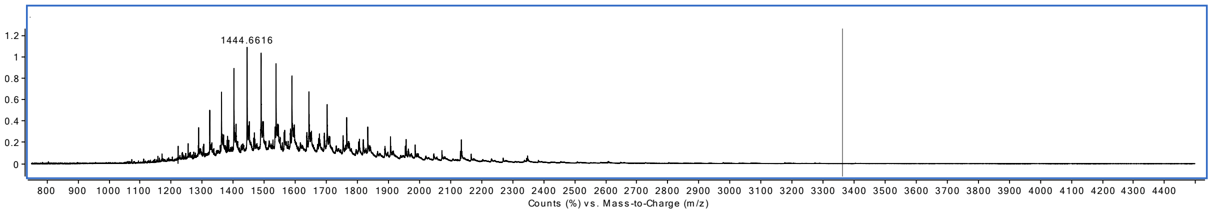

(c)

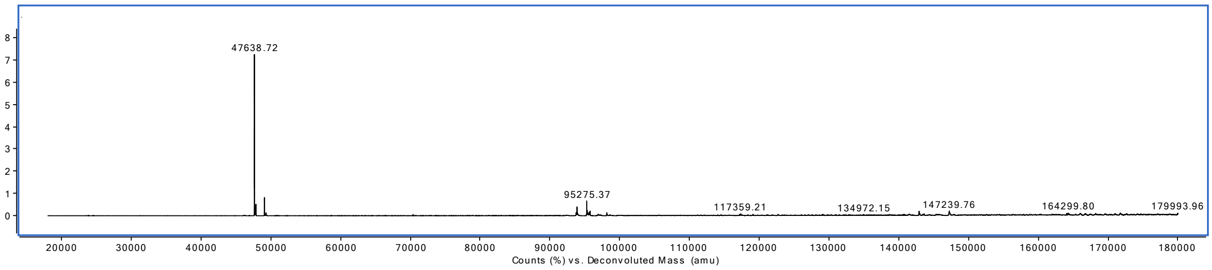

Figure S19: LCMS analysis of conjugate **S10**; a) TIC, b) non-deconvoluted ion-series, c) full range deconvoluted ion series mass spectrum, d) zoomed in deconvoluted ion series mass spectrum; Expected mass of re-bridged Fab with 5,6-bis((methyl(prop-2-yn-1-yl)amino)methyl)pyrazine-2,3-dicarbonitrile after CuAAC: 49079, Observed: 47638, 49083.

Reaction of Fab with methyl 2-((3-cyanopyrazine-2-carbonyl)thio)acetate

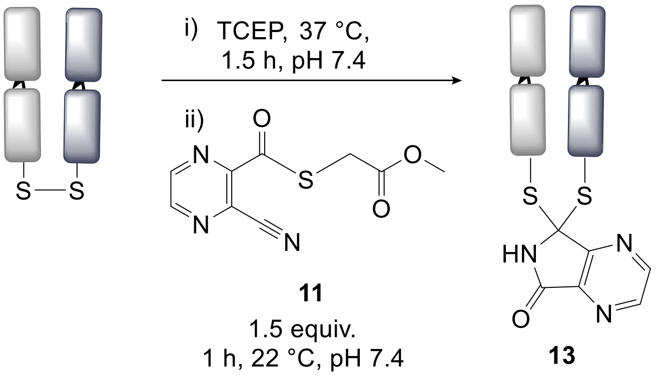

Fab (20 µL, 150 µM, 4.76 mg/mL) in conjugation buffer was reduced with tris(2-carboxyethyl)phosphine (TCEP) (2.0 µL, 15 mM in diH_2O, 10 eq.) The mixture was incubated at 37 °C for 1.5 h, 300 rpm. Methyl 2-((3-cyanopyrazine-2-carbonyl)thio)acetate **11** (0.9 µL, 5 mM in DMF, 1.5 eq.) was added and incubated at 22 °C for 1 h. Lastly, excess reagent was removed, and the sample was desalted (7 kDa MWCO, ZebaSpin) prior to LCMS analysis. Concentration was determined photometrically using ε280= 68590 $M^{-1}cm^{-1}$.

(a)

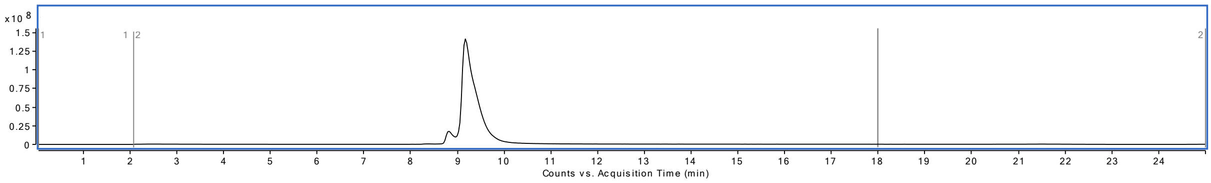

(b)

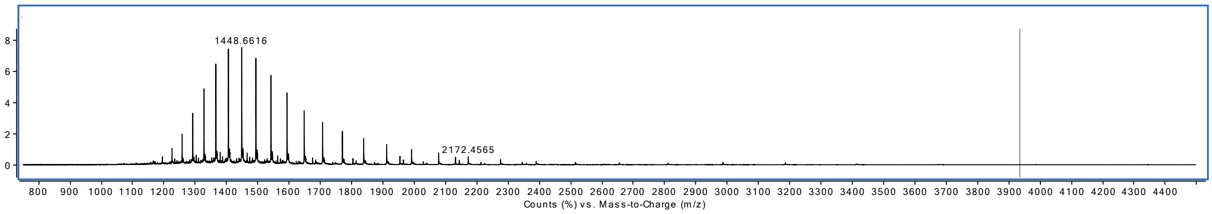

(c)

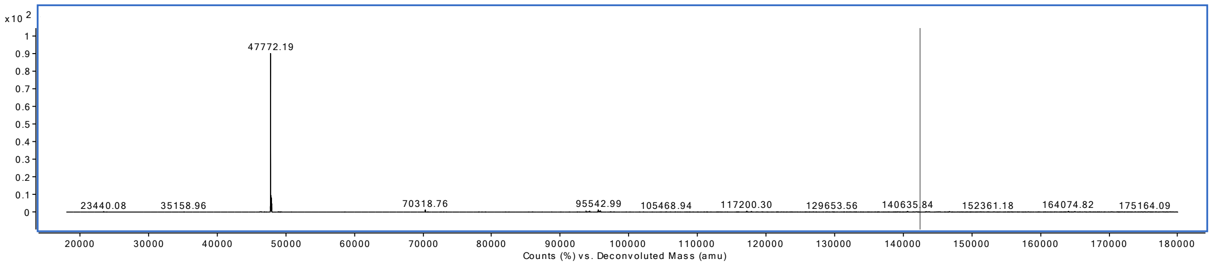

(d)

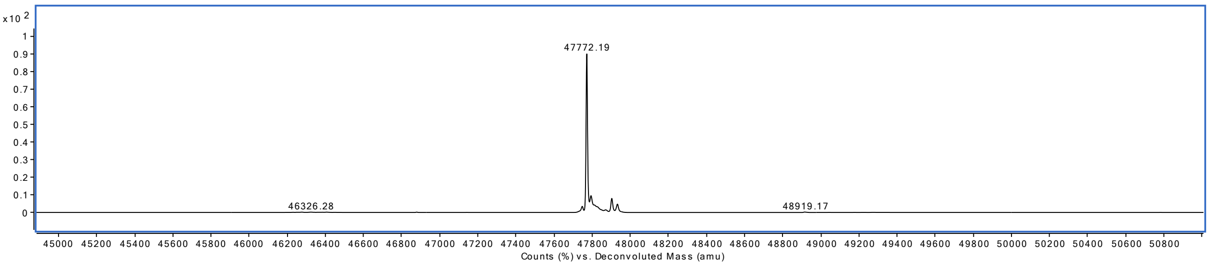

Figure S20: LCMS analysis of conjugate **13**; a) TIC, b) non-deconvoluted ion-series, c) full range deconvoluted ion series mass spectrum, d) zoomed in deconvoluted ion series mass spectrum; Expected mass of re-bridged Fab with methyl 2-((3-cyanopyrazine-2-carbonyl)thio)acetate: 47771, Observed: 47772.

Reaction of Fab with diethyl 5,6-dicyanopyrazine-2,3-dicarboxylate

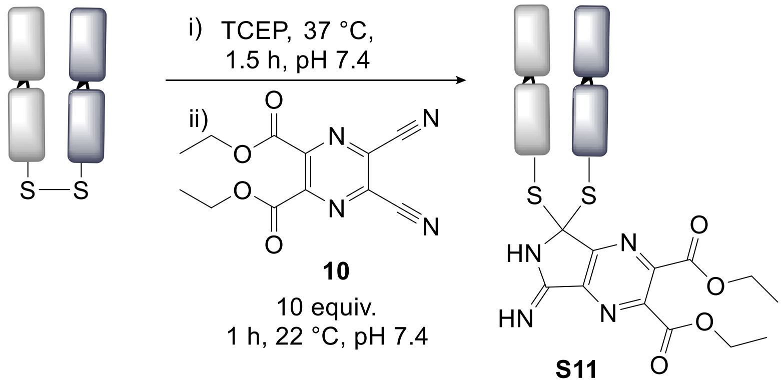

Fab (20 µL, 150 µM, 4.76 mg/mL) in conjugation buffer was reduced with tris(2-carboxyethyl)phosphine (TCEP) (2.0 µL, 15 mM in diH_2O, 10 eq.) The mixture was incubated at 37 °C for 1.5 h, 300 rpm. Diethyl 5,6-dicyanopyrazine-2,3-dicarboxylate **10** (2.0 µL, 15 mM in DMF, 10 eq.) was added and incubated at 22 °C for 1 h. Lastly, excess reagent was removed, and the sample was desalted (7 kDa MWCO, ZebaSpin) prior to LCMS analysis. Concentration was determined photometrically using ε280= 68590 $M^{-1}cm^{-1}$.

(a)

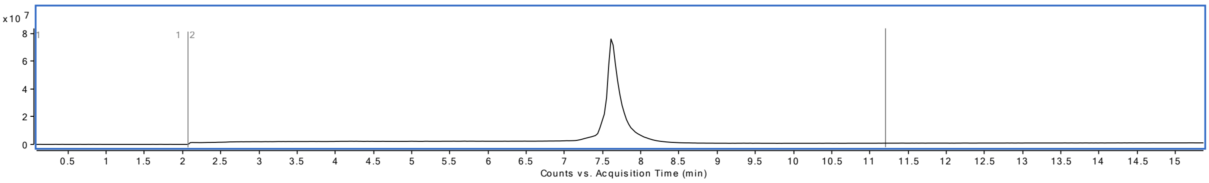

(b)

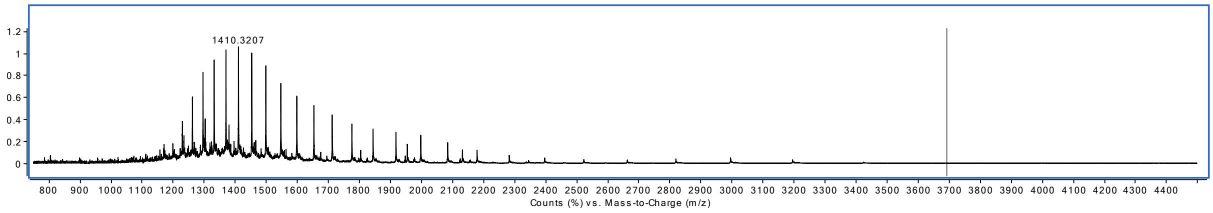

(c)

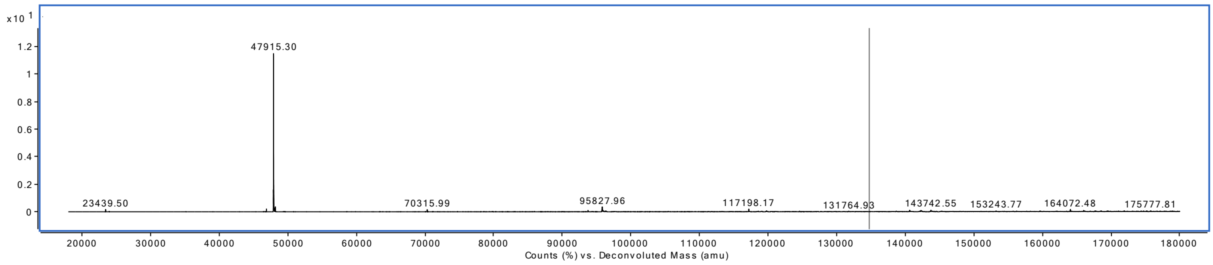

Figure S21: LCMS analysis of conjugate **S11**; a) TIC, b) non-deconvoluted ion-series, c) full range deconvoluted ion series mass spectrum; Expected mass of re-bridged Fab with diethyl 5,6-dicyanopyrazine-2,3-dicarboxylate:47912, Observed: 47915.

Removal of diethyl 5,6-dicyanopyrazine-2,3-dicarboxylate bridge

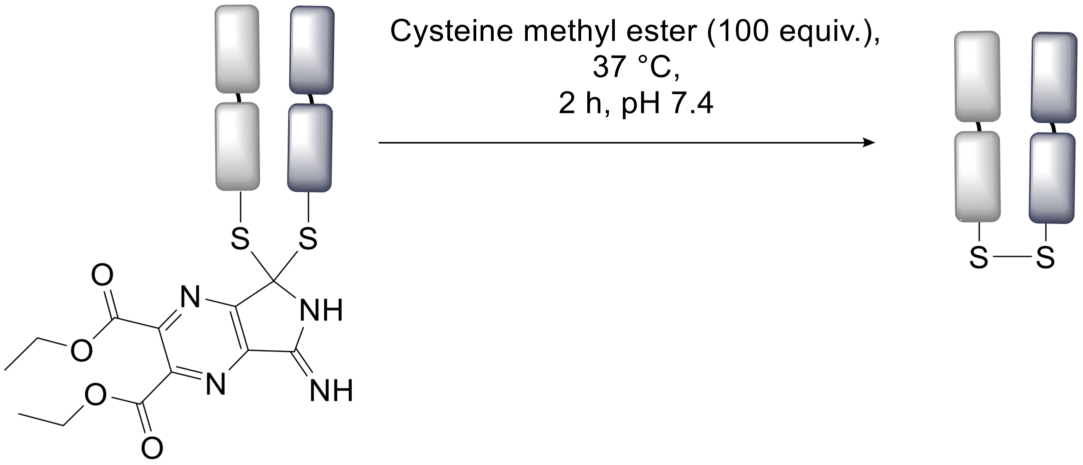

To conjugate S11 was added cysteine methyl ester (150mM in diH_2O, 100 eq.), the reaction was left at 37 °C, 300 rpm for 2 h. After this time the excess reagent was then removed *via* ultrafiltration (10 kDa MWCO) into HPLC grade water prior to LCMS analysis. Concentration was determined photometrically using ε280= 68590 $M^{-1}cm^{-1}$.

(a)

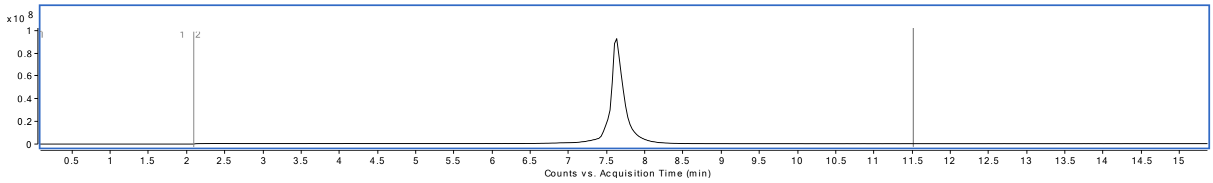

(b)

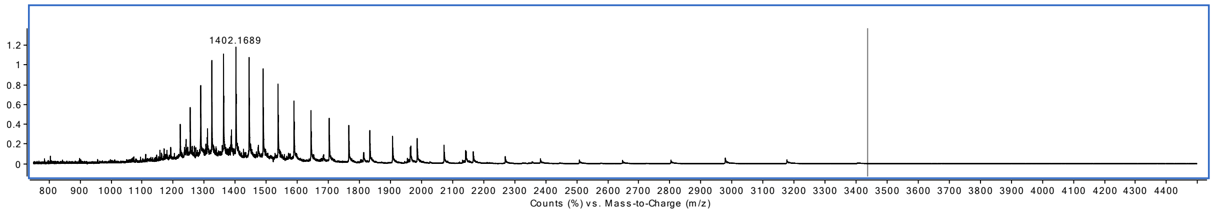

(c)

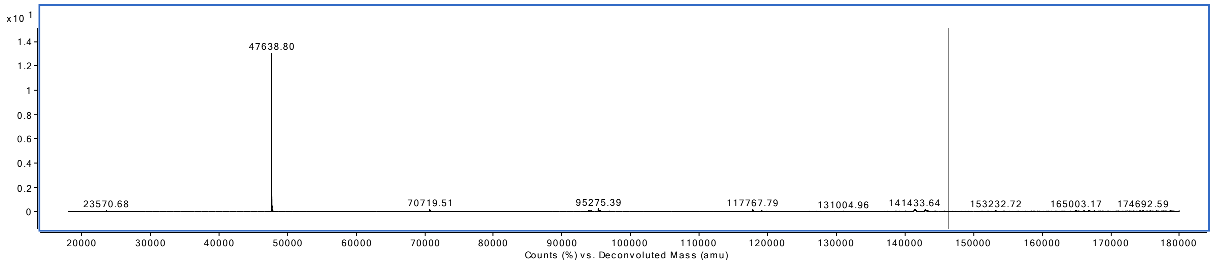

(d)

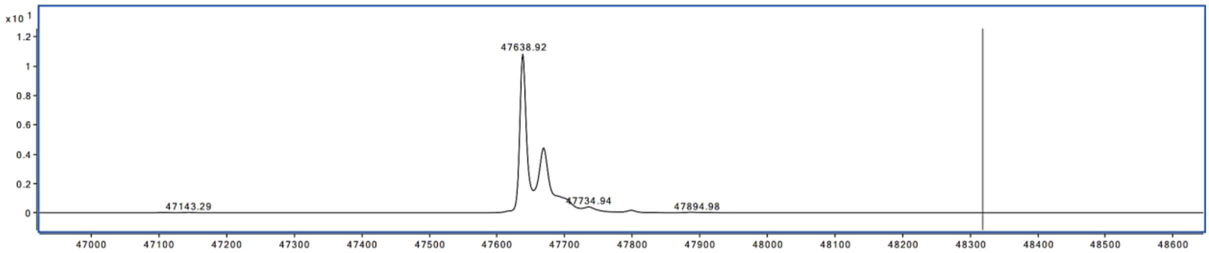

Figure S22: LCMS analysis of conjugate **S11** removal; a) TIC, b) non-deconvoluted ion-series, c) full range deconvoluted ion series mass spectrum; Expected mass of bridge removal Fab with cysteine methyl ester: 47638, Observed: 47638. d) zoomed in deconvoluted ion series mass spectrum; Expected mass of bridge removal Fab with cysteine methyl ester: 47638, Observed: 47638, 47668 (-OMe adduct due to excess cysteine methyl ester).

Reaction of Fab with *N*-(3-azidopropyl)-2,3-dicyanoquinoxaline-6-carboxamide

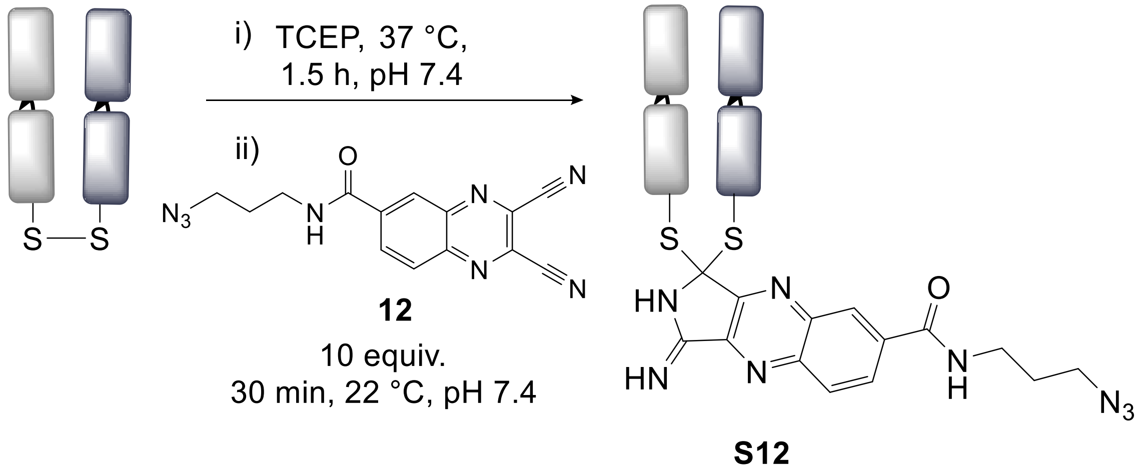

Fab (20 µL, 150 µM, 4.76 mg/mL) in conjugation buffer was reduced with tris(2-carboxyethyl)phosphine (TCEP) (2.0 µL, 15 mM in diH_2O, 10 eq.) The mixture was incubated at 37 °C for 1.5 h, 300 rpm. *N*-(3-azidopropyl)-2,3-dicyanoquinoxaline-6-carboxamide **6** (2.0 µL, 15 mM in DMF, 10 eq.) was added and incubated at 22 °C for 30 min. Lastly, excess reagent was removed, and the sample was desalted *via* ultrafiltration (7 kDa MWCO, ZebaSpin) prior to LCMS analysis. Concentration was determined photometrically using ε280= 68590 $M^{-1}cm^{-1}$.

(a)

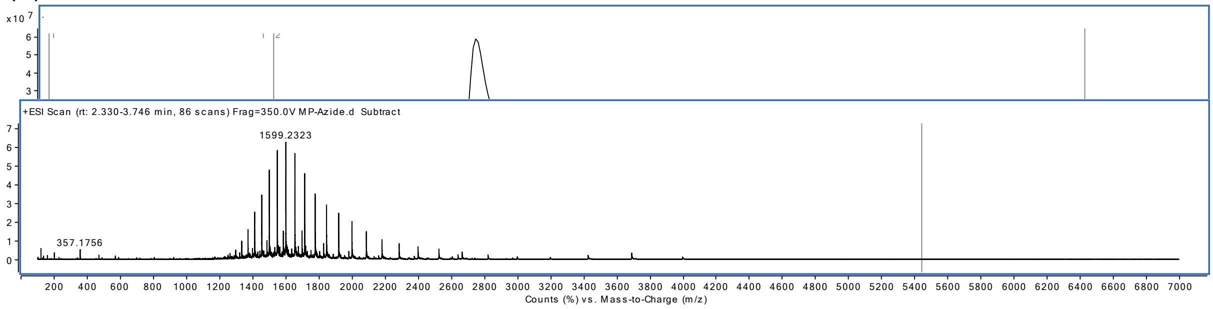

(b)

(c)

(d)

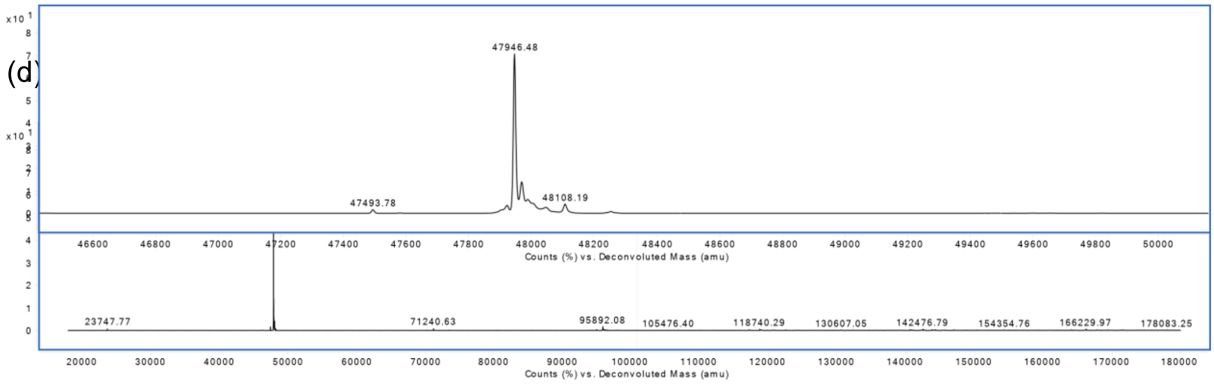

Figure S23: LCMS analysis of conjugate **S12**; a) TIC, b) non-deconvoluted ion-series, c) full range deconvoluted ion series mass spectrum; Expected mass of re-bridged Fab with *N*-(3-azidopropyl)-2,3-dicyanoquinoxaline-6-carboxamide:47944, Observed: 47946.

Reaction of Fab with *N*-(3-azidopropyl)-2,3-dicyanoquinoxaline-6-carboxamide and SPAAC

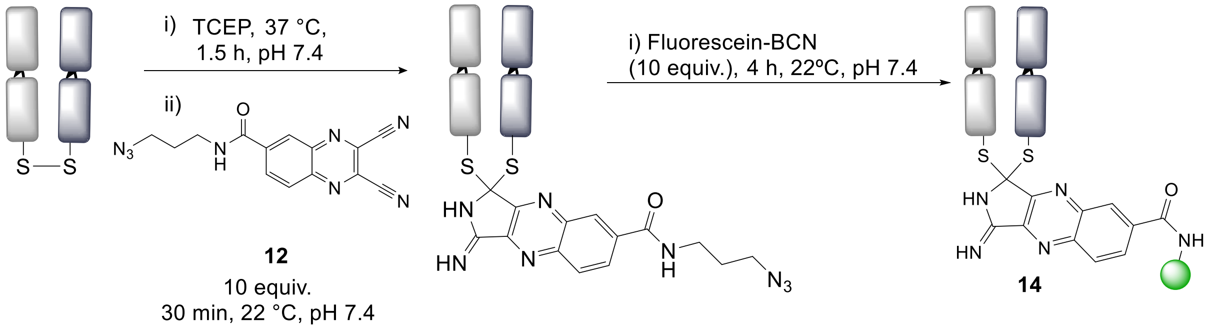

Fab (20 µL, 150 µM, 4.76 mg/mL) in conjugation buffer was reduced with tris(2-carboxyethyl)phosphine (TCEP) (2.0 µL, 15 mM in diH_2O, 10 eq.) The mixture was incubated at 37 °C for 1.5 h, 300 rpm. *N*-(3-azidopropyl)-2,3-dicyanoquinoxaline-6-carboxamide **12** was then added (2.0 µL, 150 mM in DMF, 10 eq.) was added and incubated at 22 °C for 30 min. Excess reagent was then removed *via* ultrafiltration (7 kDa MWCO, ZebaSpin) into PBS (pH = 7.4). To conjugate S12 was added fluorescein-PEG_3-BCN (20 mM in DMF, 10 eq.), this was then left in the dark at 22 °C for 4 h, 300 rpm. Excess reagent was then removed by ultrafiltration (10 kDa MWCO) into HPLC grade water prior to LCMS analysis. Concentration was determined photometrically using ε_{280}= 68590 M^{-1} cm^{-1}.

(a)

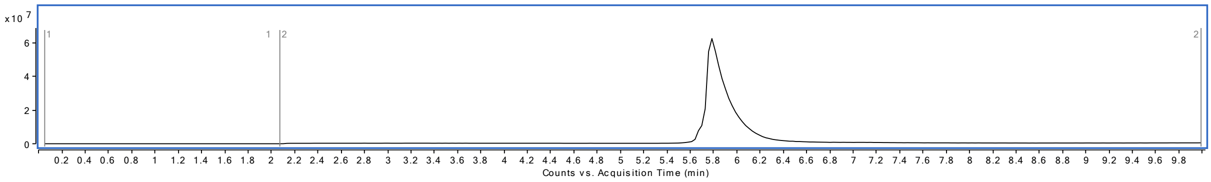

(b)

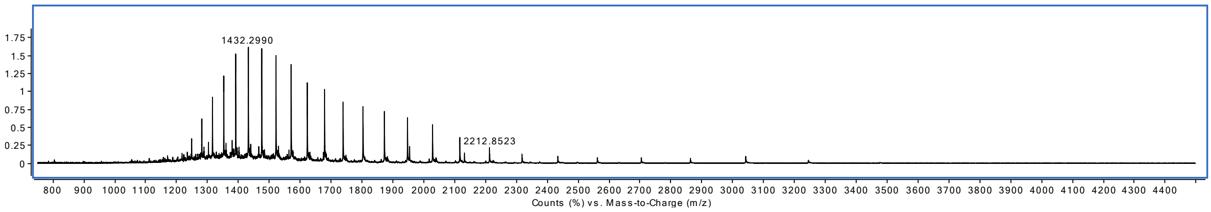

(c)

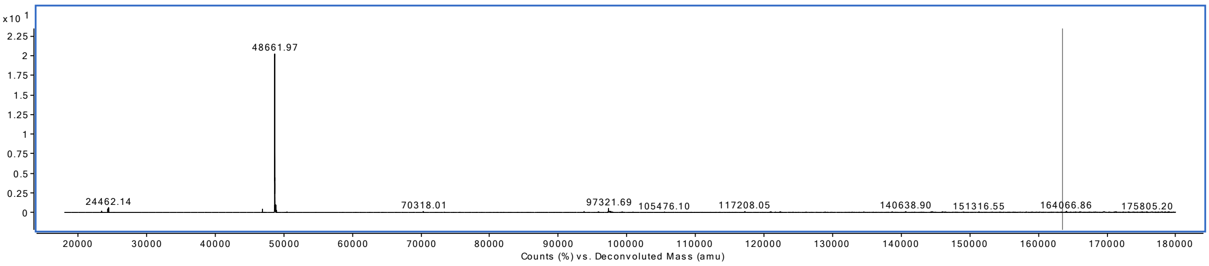

(d)

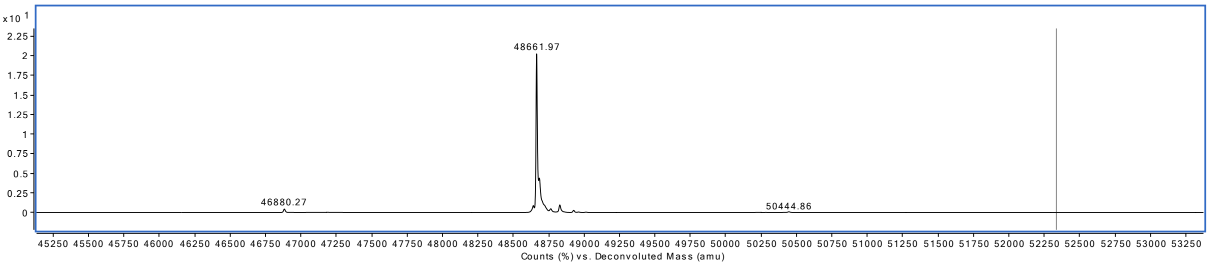

Figure S24: LCMS analysis of conjugate **14**; a) TIC, b) non-deconvoluted ion-series, c) full range deconvoluted ion series mass spectrum, d) zoomed in deconvoluted ion series mass spectrum; Expected mass of re-bridged Fab with *N*-(3-azidopropyl)-2,3-dicyanoquinoxaline-6-carboxamide after SPAAC:48670, Observed: 48662.

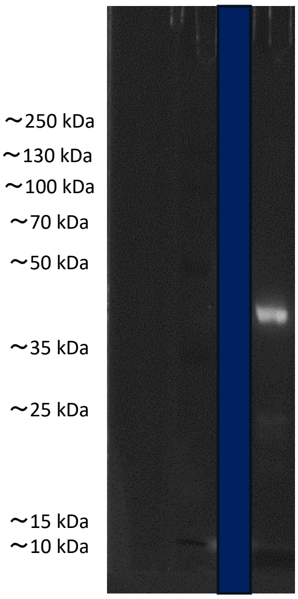

Figure S25: SDS-PAGE analysis of conjugate 14 (Bridge); M – molecular marker, 1 – Conjugate **14**, The fluorescent bands were visualised using an AZURE 200® (Epi Blue).

Reaction of Fab with 2,3-dicyano-N-(prop-2-yn-1-yl)quinoxaline-6-carboxamide.

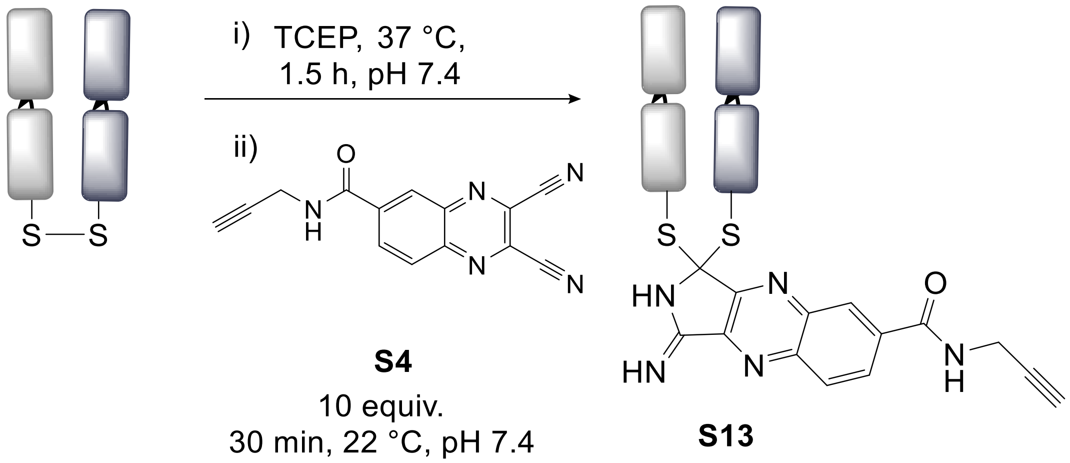

Fab (20 µL, 150 µM, 4.76 mg/mL) in conjugation buffer was reduced with tris(2-carboxyethyl)phosphine (TCEP) (2.0 µL, 15 mM in diH_2O, 10 eq.) The mixture was incubated at 37 °C for 1.5 h, 300 rpm. 2,3-dicyano-N-(prop-2-yn-1-yl)quinoxaline-6-carboxamide **S4** (2.0 µL, 15 mM in DMF, 10 eq.) was added and incubated at 22 °C for 30 min. Lastly, excess reagent was removed, and the sample was desalted *via* ultrafiltration (7 kDa MWCO, ZebaSpin) prior to LCMS analysis. Concentration was determined photometrically using ε280= 68590 $M^{-1}cm^{-1}$.

(a)

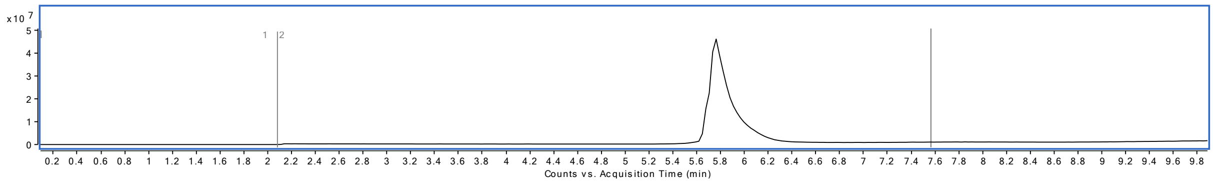

(b)

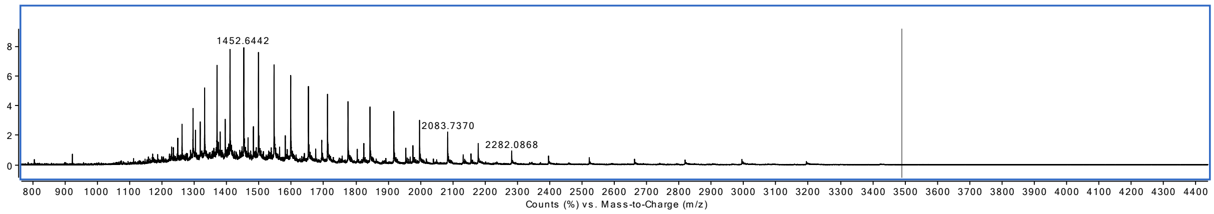

(c)

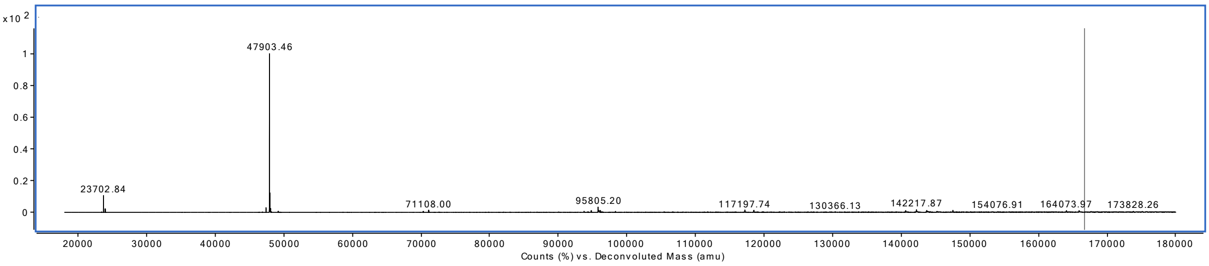

(d)

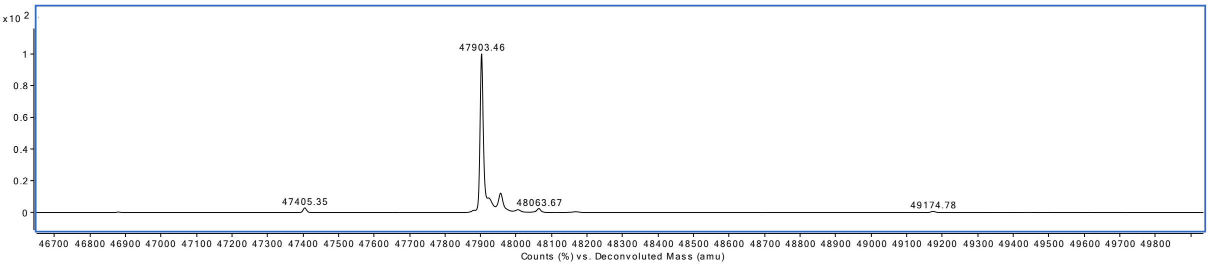

Figure S26: LCMS analysis of conjugate **S13**; a) TIC, b) non-deconvoluted ion-series, c) full range deconvoluted ion series mass spectrum, d) zoomed in deconvoluted ion series mass spectrum; Expected mass of re-bridged Fab with 2,3-dicyano-N-(prop-2-yn-1-yl)quinoxaline-6-carboxamide:47899, Observed: 47903.

Reaction of Fab with 2,3-dicyano-N-(prop-2-yn-1-yl)quinoxaline-6-carboxamide with CuAAC.

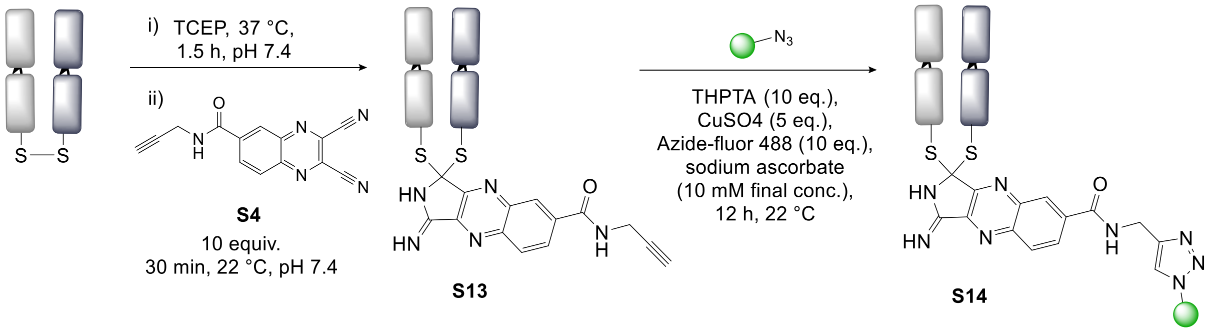

Fab (20 μL, 150 μM, 4.76 mg/mL) in conjugation buffer was reduced with TCEP (2.0 μL, 15 mM in diH_2O, 10 eq.) The mixture was incubated at 37 °C for 1.5 h, 300 rpm. 2,3-Dicyano-*N*-(prop-2-yn-1-yl)quinoxaline-6-carboxamide **S4** (2.0 μL, 15 mM in DMF, 10 eq.) was added and incubated at 22 °C for 30 min. Excess reagent was removed *via* ultrafiltration (7 kDa MWCO, ZebaSpin) into PB (pH 7.0). To conjugate **S13** was added THPTA (100 mM in diH_2O, 10 eq.), $CuSO_4$ (20 mM in diH_2O, 5 eq.), Azide-fluor 488 (10 mM in DMF, 10 eq.), and sodium ascorbate (100 mM in diH_2O, final conc. 10 mM), the reaction was left at 22 °C, 300 rpm for 4 h. The excess reagent was then removed using a desalting column (PD Minitrap G-25, GE

Healthcare) followed by ultrafiltration (10 kDa MWCO) into conjugate buffer to concentrate the sample. Lastly, the sample was desalted into HPLC grade water (7 kDa MWCO, ZebaSpin) prior to LCMS analysis. Concentration was determined photometrically using ε280= 68590 $M^{-1}cm^{-1}$.

(a)

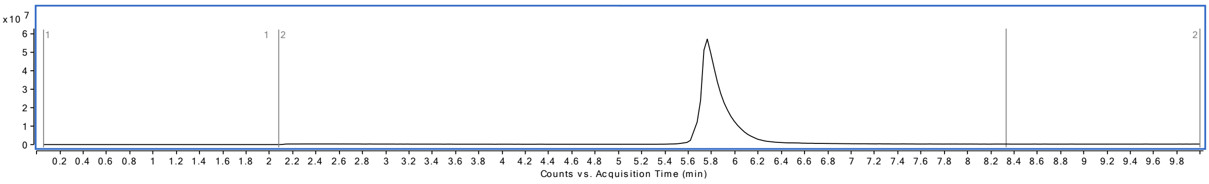

(b)

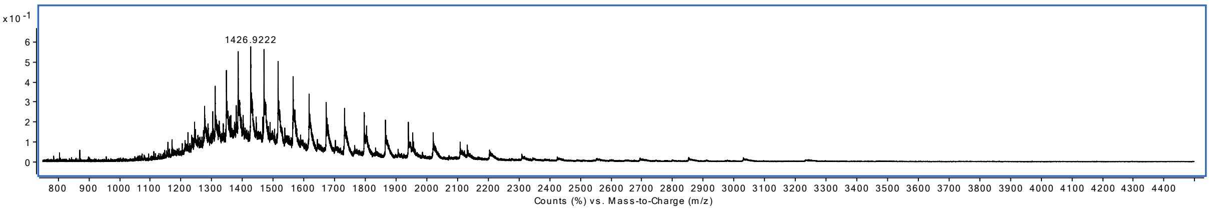

(c)

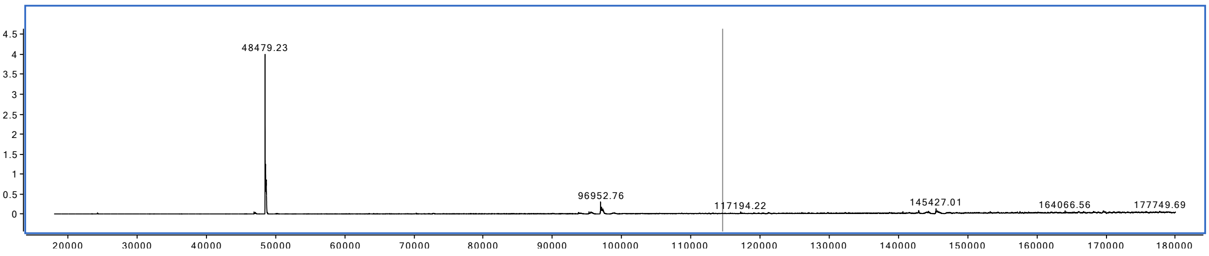

(d)

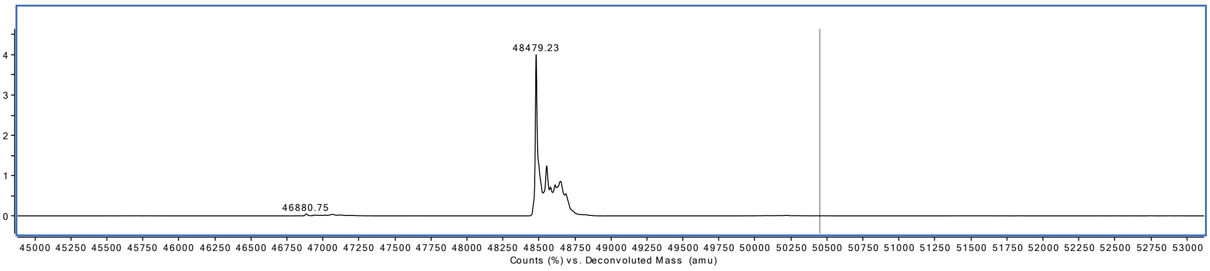

Figure S27: LCMS analysis of conjugate **S14**; a) TIC, b) non-deconvoluted ion-series, c) full range deconvoluted ion series mass spectrum, d) zoomed in deconvoluted ion series mass spectrum; Expected mass of re-bridged Fab with 2,3-dicyano-N-(prop-2-yn-1-yl)quinoxaline-6-carboxamide after CuAAC: 48473. Observed: 48479.

Re-bridging of full antibody with *N*-(3-azidopropyl)-2,3-dicyanoquinoxaline-6-carboxamide

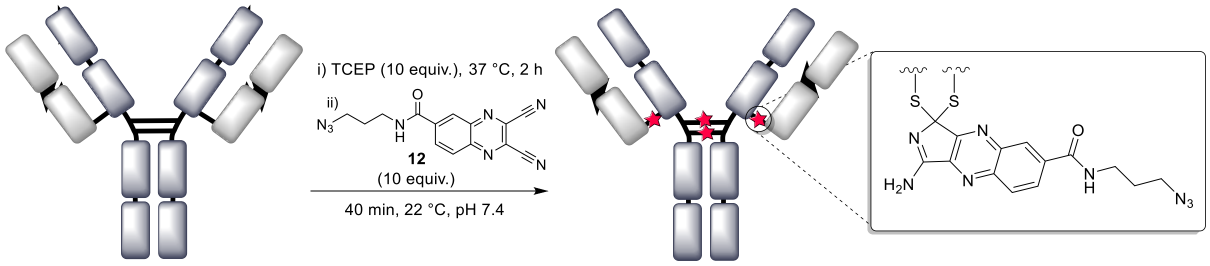

S15

Trastuzumab full antibody (80 µL, 25 µM, 5.8 mg/mL) in conjugation buffer was reduced with TCEP (1.3 µL, 15 mM in diH_2O, 10 eq.). The mixture was incubated at 37 °C for 2 h, 300 rpm. Following this *N*-(3-azidopropyl)-2,3-dicyanoquinoxaline-6-carboxamide (0.92 µL, 21.8 mM in DMF, 10 eq.) was added and incubated at 22 °C for 40 min. The excess reagent was removed *via* ultrafiltration (10 kDa MWCO) into 50 mM ammonium acetate pH 6.9 (7 kDa MWCO, ZebaSpin) and adjusted to 5 µM with ammonium acetate (pH 6.9). To this (40 µL) was added 0.67 µL of PNGase F and the reaction was left at 37 °C for 16 h. Concentration was determined photometrically using ε_{280}=215380 M^{-1} cm^{-1}.

(a)

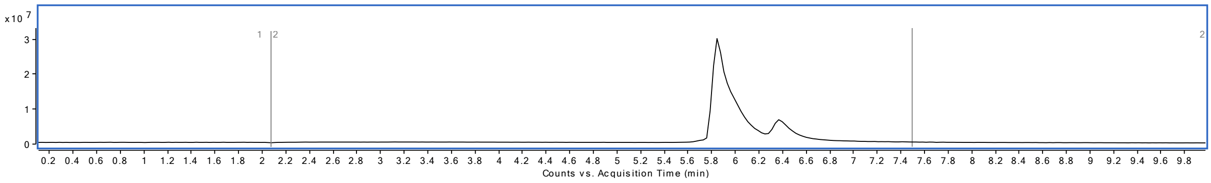

(b)

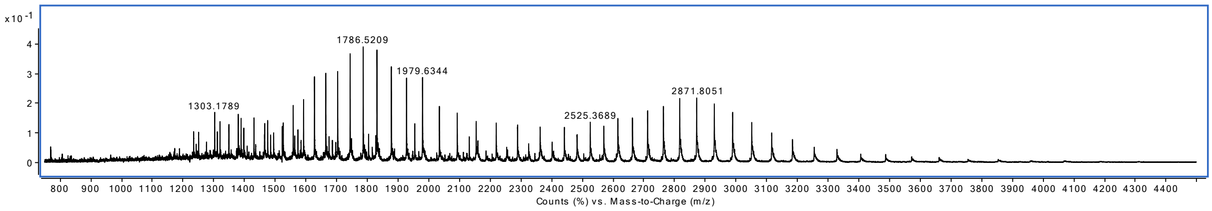

(c)

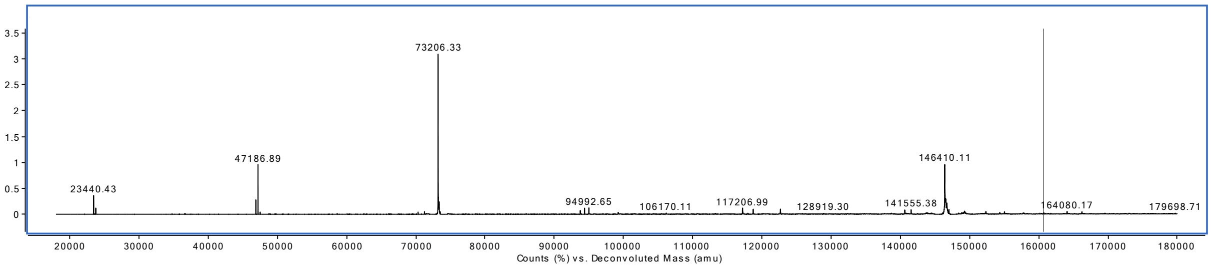

(d)

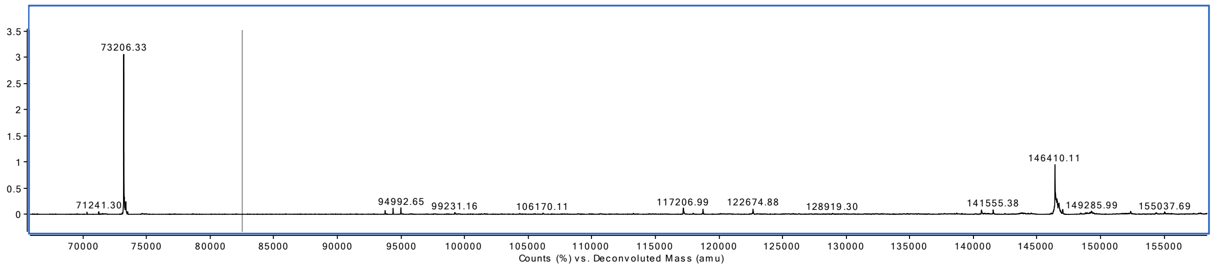

Figure S28: LCMS analysis of conjugate **S15**; a) TIC, b) non-deconvoluted ion-series, c) full range deconvoluted ion series mass spectrum, d) zoomed in deconvoluted ion series mass spectrum; Expected mass of re-bridged trastuzumab with *N*-(3-azidopropyl)-2,3-dicyanoquinoxaline-6-carboxamide: HHLL 146403, HL 73206, Observed: HHLL 146410, HL 73206, LC 23440.

Re-bridging of full antibody with *N*-(3-azidopropyl)-2,3-dicyanoquinoxaline-6-carboxamide and SPAAC

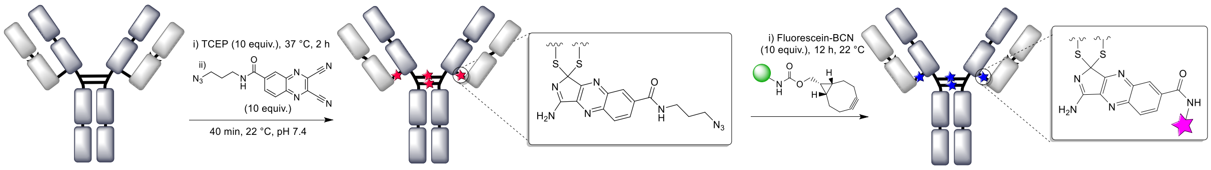

Trastuzumab full antibody (80 µL, 25 µM, 5.8 mg/mL) in conjugation buffer was reduced with TCEP (1.3 µL, 15 mM in diH_2O, 10 eq.). The mixture was incubated at 37 °C for 2 h, 300 rpm. Following this *N*-(3-azidopropyl)-2,3-dicyanoquinoxaline-6-carboxamide (0.92 µL, 21.8 mM in DMF, 10 eq.) was added and incubated at 22 °C for 40 min. After this time, excess reagent was removed *via* ultrafiltration (7 kDa MWCO, ZebaSpin) into PBS (pH = 7.4). To conjugate X was added fluorescein-PEG$_3$-BCN (20 mM in DMF, 20 eq.), this was then left in the dark at 22 °C for 12 h, 300 rpm. The excess reagent was removed *via* ultrafiltration (10 kDa MWCO) into 50 mM ammonium acetate pH 6.9 (7 kDa MWCO, ZebaSpin) and adjusted to 5 µM with ammonium acetate (pH 6.9). To this (40 µL) was added 0.67 µL of PNGase F and the reaction was left at 37 °C for 16 h. Concentration was determined photometrically using ε_{280}=215380 M^{-1} cm^{-1}.

(a)

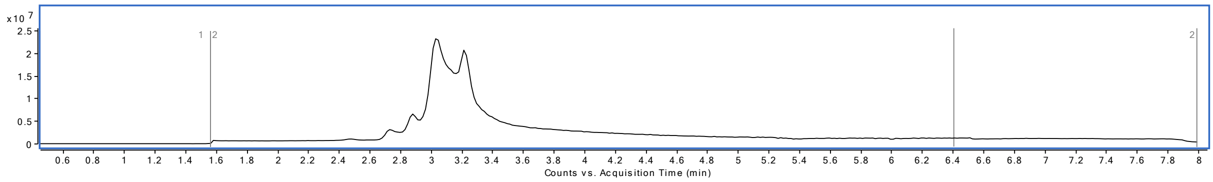

(b)

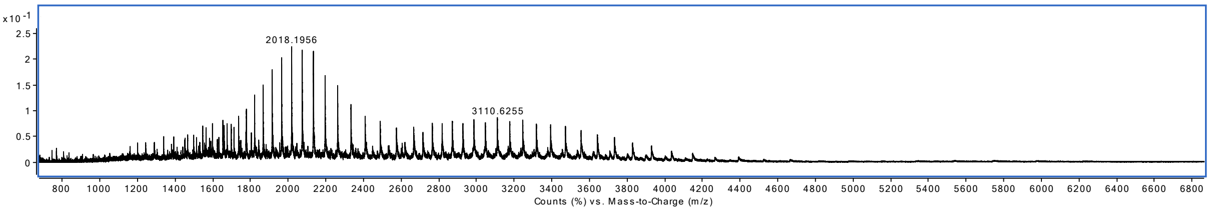

(c)

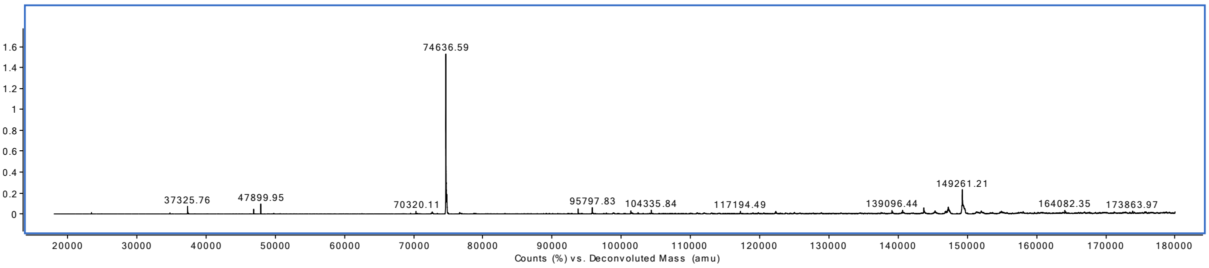

(d)

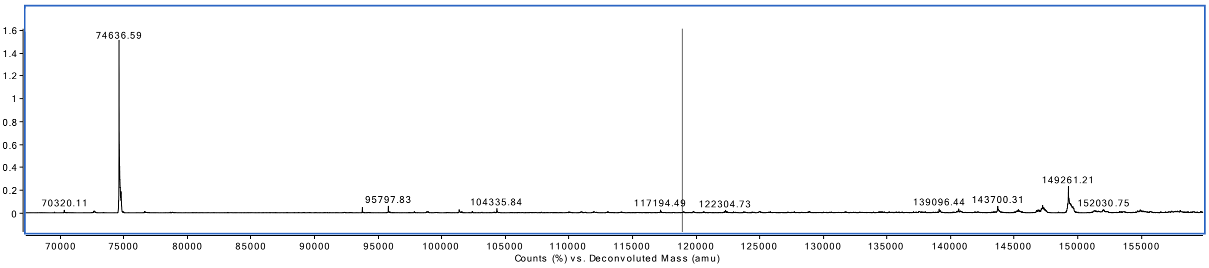

Figure S29: LCMS analysis of conjugate **15**; a) TIC, b) non-deconvoluted ion-series, c) full range deconvoluted ion series mass spectrum, d) zoomed in deconvoluted ion series mass spectrum; Expected mass of re-bridged trastuzumab with *N*-(3-azidopropyl)-2,3-dicyanoquinoxaline-6-carboxamide after SPAAC: HHLL 149306, HL 74658, Observed: HHLL 149261, HL 74636.

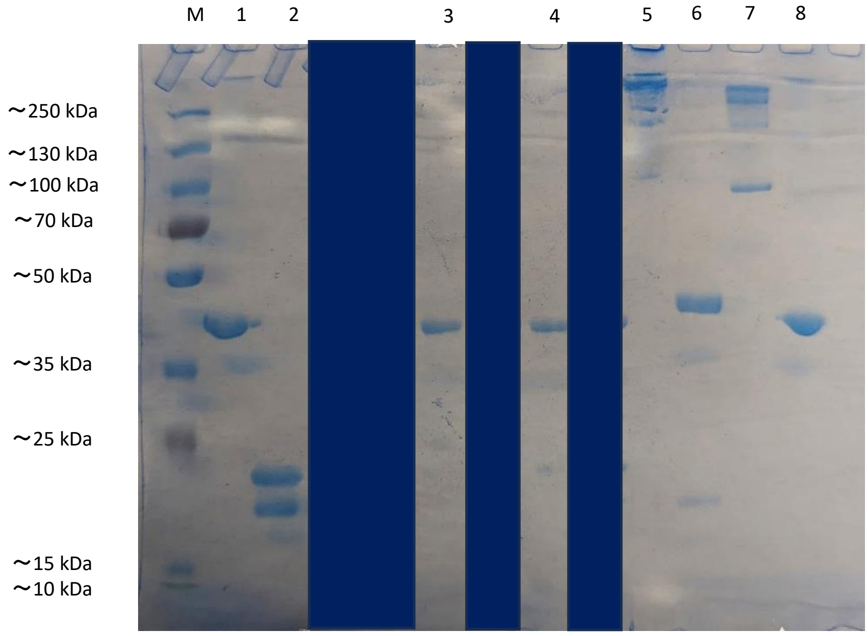

Figure S30: SDS-PAGE analysis; M – molecular marker, 1 – Native Fab, 2 – Reduced Fab, 3 – Re-bridged Fab with **3**, 4 – Re-bridged Fab with **12**, 5 – Full antibody, 6 – Reduced full antibody, 7 – Full antibody re-bridged with **12**, 8 – re-bridged Fab with **11**.

Disulfide competition experiment (GSH Vs Fab)- NMM

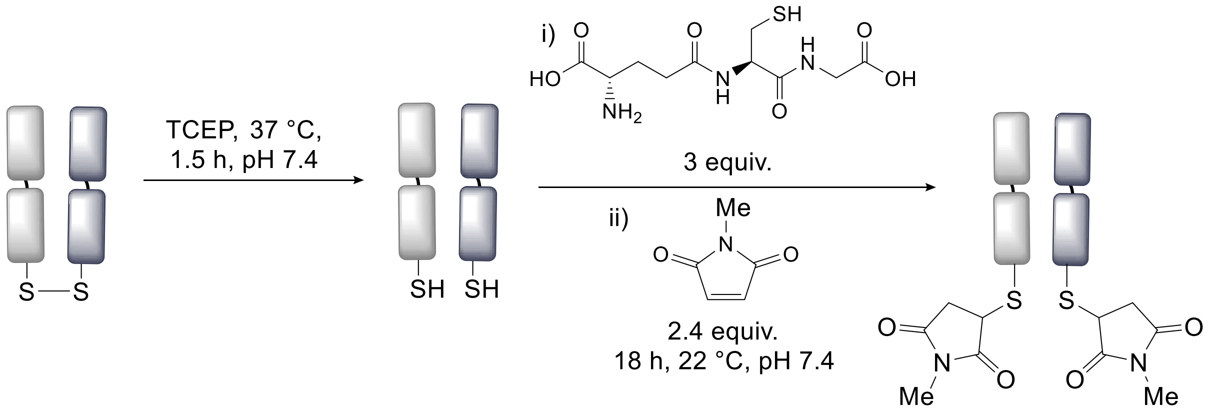

Fab (20 µL, 150 µM, 4.76 mg/mL) in conjugation buffer was reduced with TCEP (2.0 µL, 15 mM in diH_2O, 10 eq.) The mixture was incubated at 37 °C for 1.5 h, 300 rpm. To this was added glutathione (0.9 µL, 10 mM in diH_2O, 3 eq.) followed closely by *N*-methyl maleimide (0.72 µL, 10 mM in DMF, 2.4 eq.), the reaction was then incubated at 22 °C for 18 h. Finally, excess reagent was removed, and the sample was desalted (7 kDa MWCO, ZebaSpin) prior to LCMS analysis. Concentration was determined photometrically using ε280= 68590 $M^{-1}cm^{-1}$.

(a)

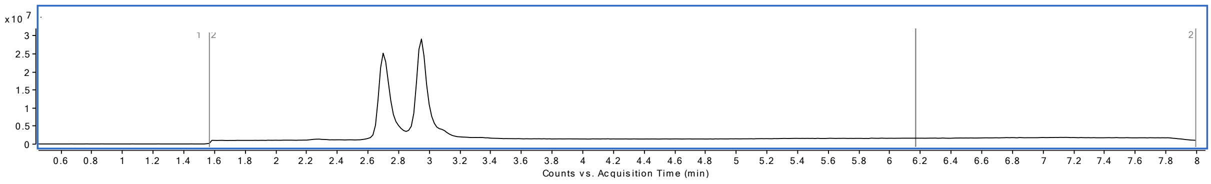

(b)

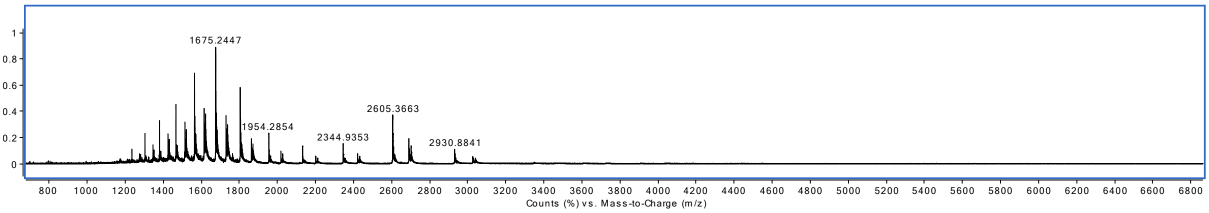

(c)

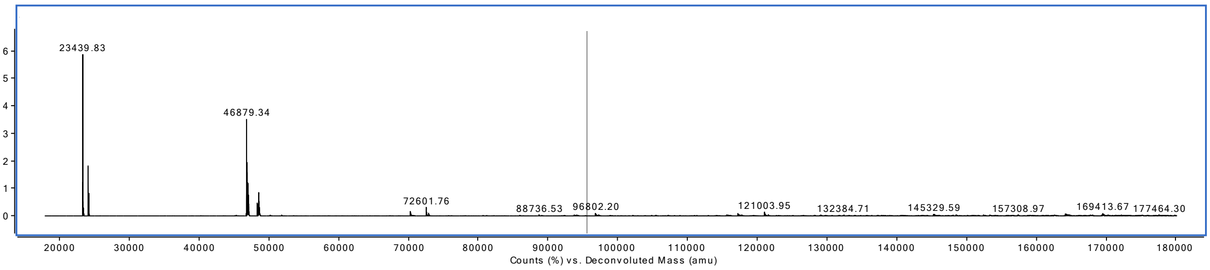

(d)

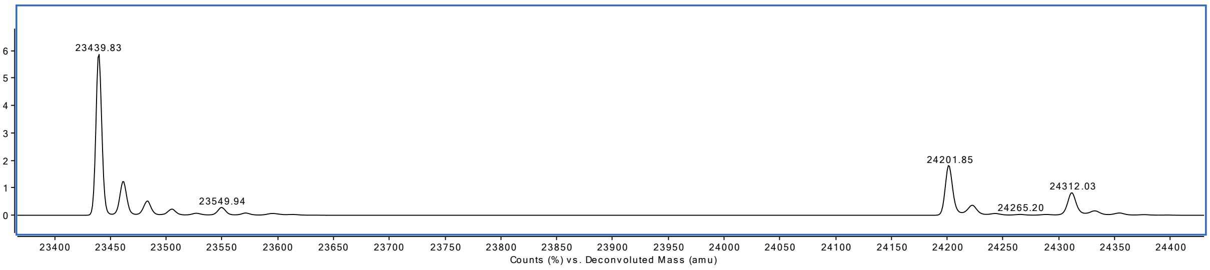

Figure S31: LCMS analysis of N-methyl maleimide competition experiment; a) TIC, b) non-deconvoluted ion-series, c) full range deconvoluted ion series mass spectrum, d) zoomed in deconvoluted ion series mass spectrum; Expected mass of capped Fab with *N*-methyl maleimide: LC 23549 HC 24311, Observed: Native LC 23439, Modified LC 23549, Native HC 24201, Modified HC 24312.

Disulfide competition experiment (GSH Vs Fab)- 2,3-dicyanoquinoxaline-6-carboxylic acid

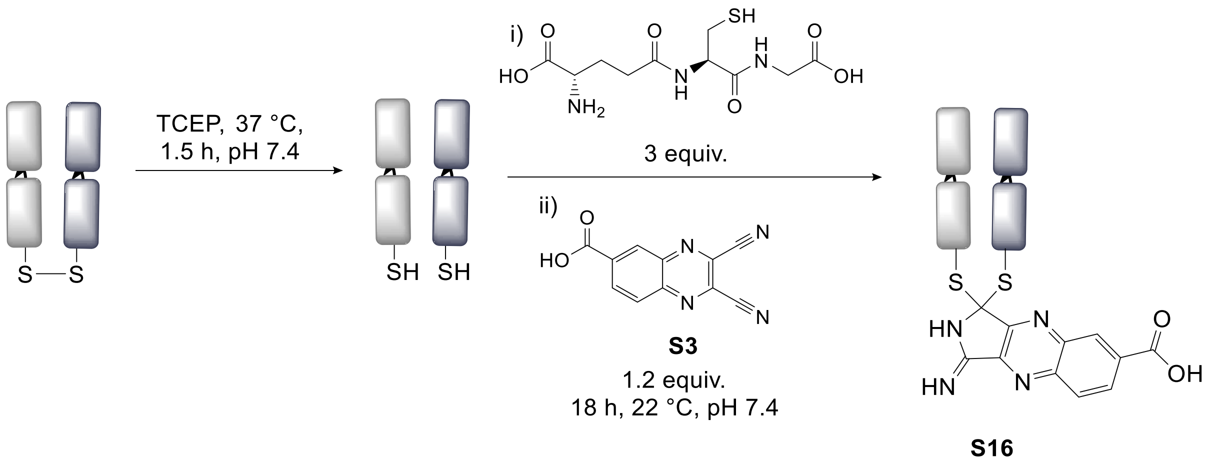

Fab (20 µL, 150 µM, 4.76 mg/mL) in conjugation buffer was reduced with TCEP (2.0 µL, 15 mM in diH_2O, 10 eq.) The mixture was incubated at 37 °C for 1.5 h, 300 rpm. To this was added glutathione (0.9 µL, 10 mM in diH_2O, 3 eq.) followed closely by 2,3-dicyanoquinoxaline-6-carboxylic acid **S3** (0.72 µL, 10 mM in DMF, 1.2 eq.), the reaction was then incubated at 22 °C for 18 h. Finally, excess reagent was removed, and the sample was desalted (7 kDa

MWCO, ZebaSpin) prior to LCMS analysis. Concentration was determined photometrically using ε280= 68590 $M^{-1}cm^{-1}$.

(a)

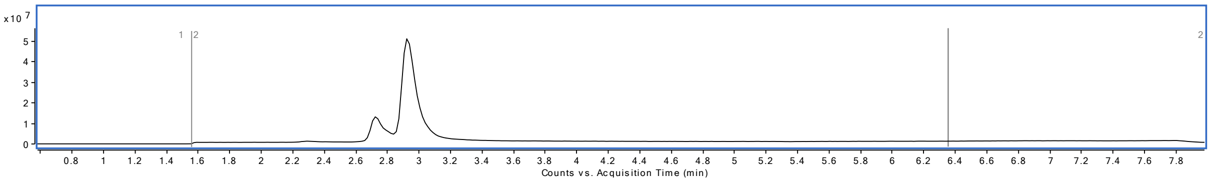

(b)

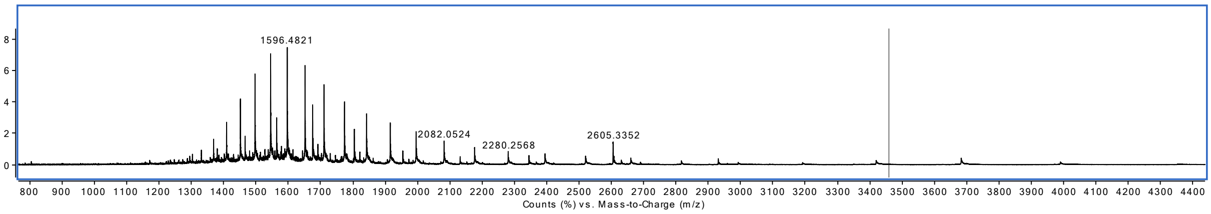

(c)

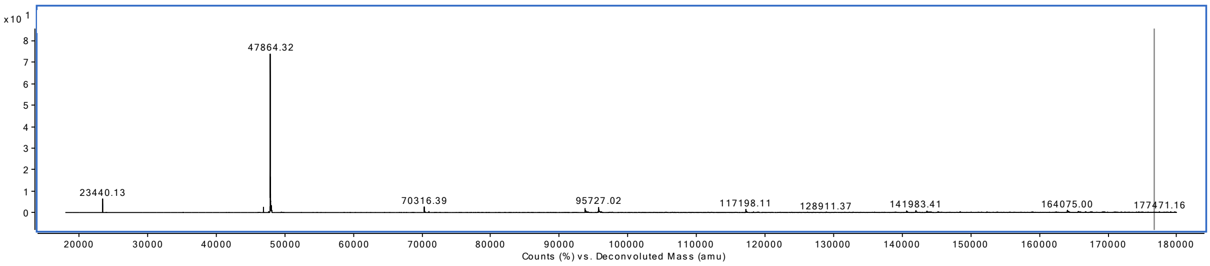

(d)

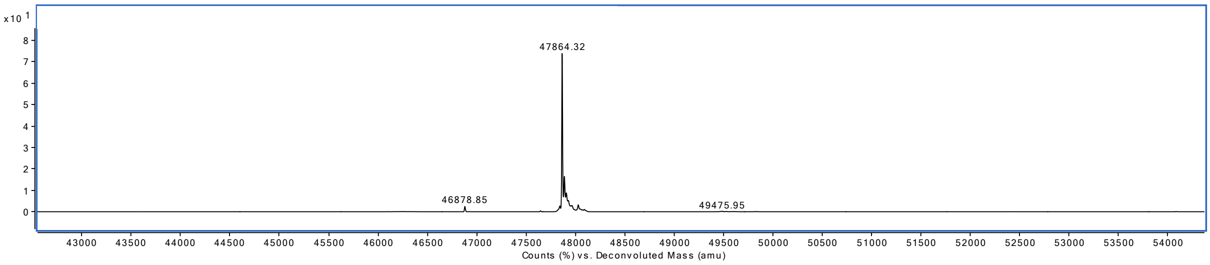

Figure S32: LCMS analysis of conjugate **S16**; a) TIC, b) non-deconvoluted ion-series, c) full range deconvoluted ion series mass spectrum, d) zoomed in deconvoluted ion series mass spectrum; Expected mass of re-bridged Fab with 2,3-dicyanoquinoxaline-6-carboxylic acid: 47862. Observed: 47864, LC 23440.

GSH stability studies

Blood mimicking Glutathione (GSH) concentration

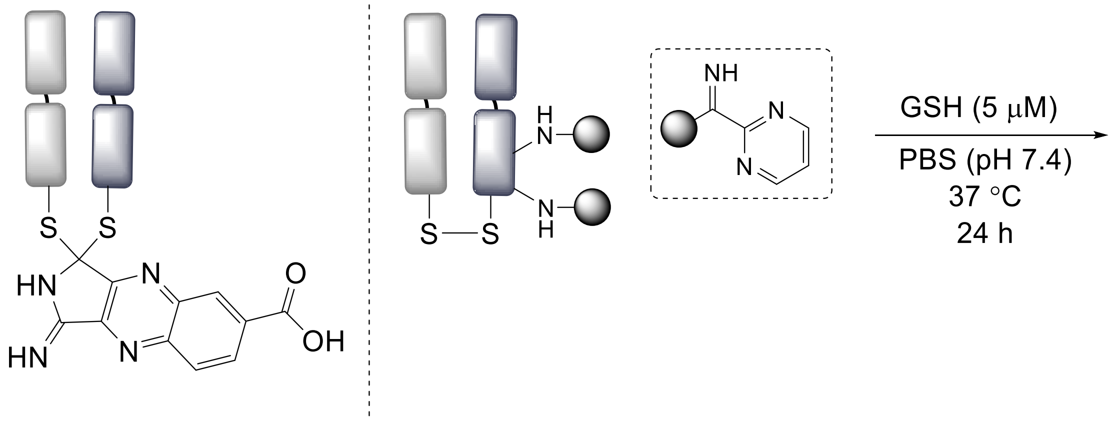

Fab conjugates S16 and S7 (50 µL, 7 µM) were buffer exchanged using desalting columns (7000 MWCO, ZebaSpin, Thermo Scientific) into GSH containing buffer (5 µM GSH, in PBS pH 7.4) and maintained at 37 °C for 24 h. Excess reagents were removed using desalting columns (7000 MWCO, ZebaSpin, Thermo Scientific) prior to LCMS analysis.

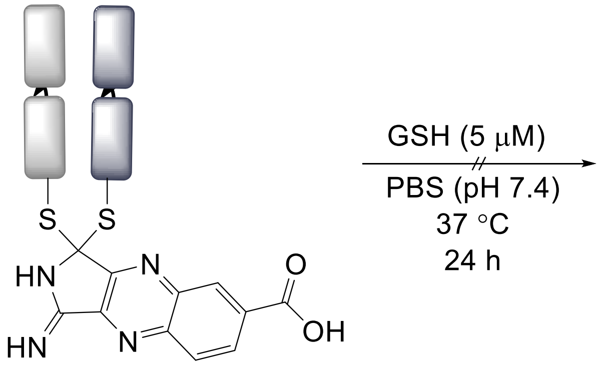

0 hours

(a)

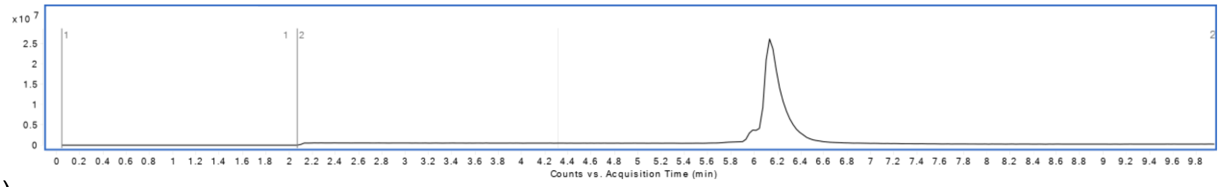

(b)

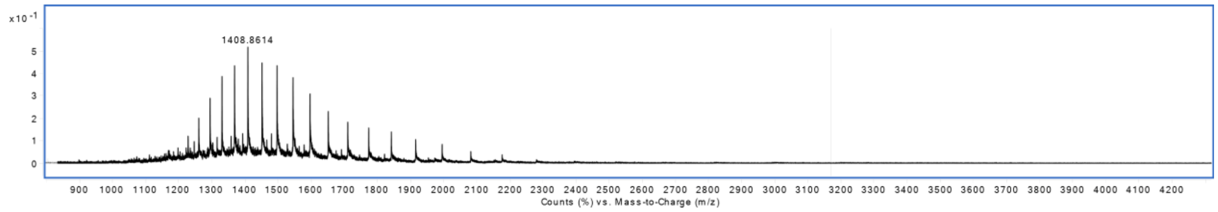

(c)

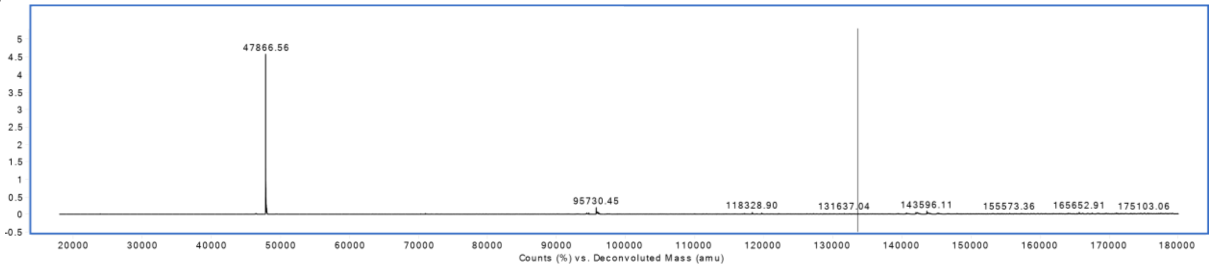

(d)

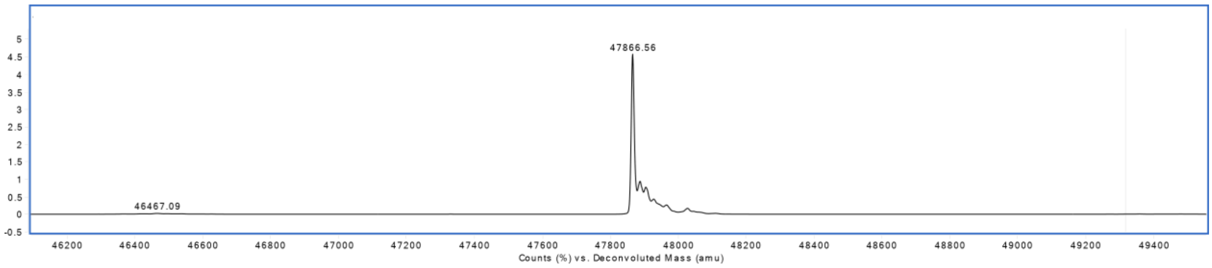

Figure S33: LCMS analysis of conjugate **S16**; a) TIC, b) non-deconvoluted ion-series, c) full range deconvoluted ion series mass spectrum; Expected mass of re-bridged Fab with 2,3-dicyanoquinoxaline-6-carboxylic acid: 47862. Observed: 47866.

24 hours

(a)

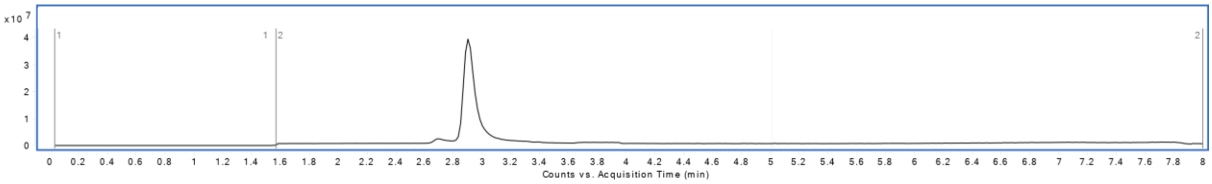

(b)

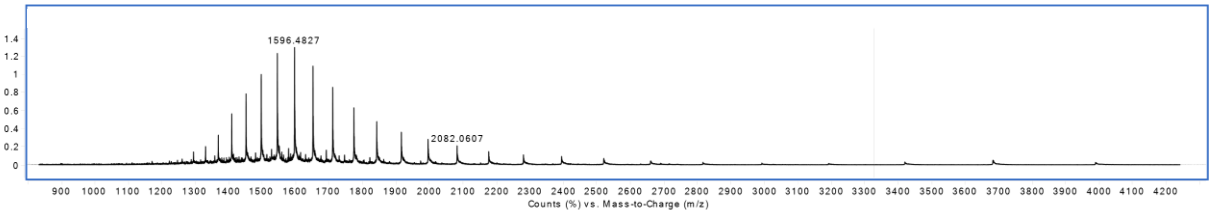

(c)

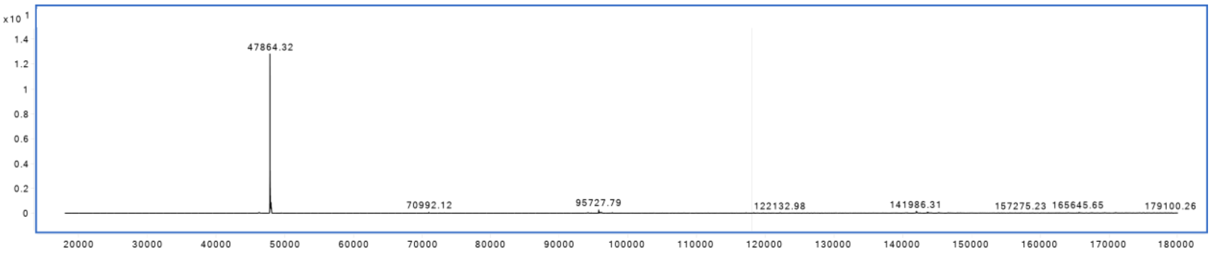

(d)

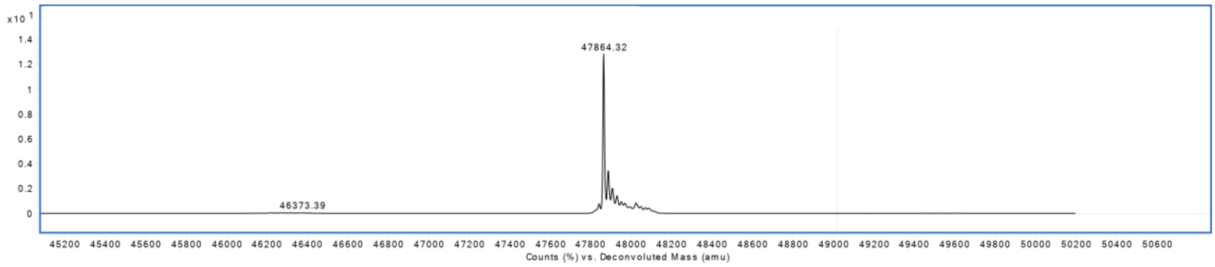

Figure S34: LCMS analysis of conjugate **S16**; a) TIC, b) non-deconvoluted ion-series, c) full range deconvoluted ion series mass spectrum; Expected mass of re-bridged Fab with 2,3-dicyanoquinoxaline-6-carboxylic acid: 47862. Observed: 47864.

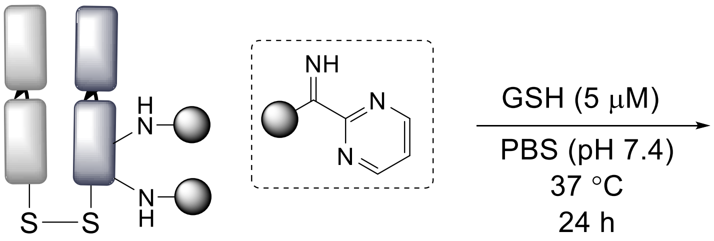

0 hours

(a)

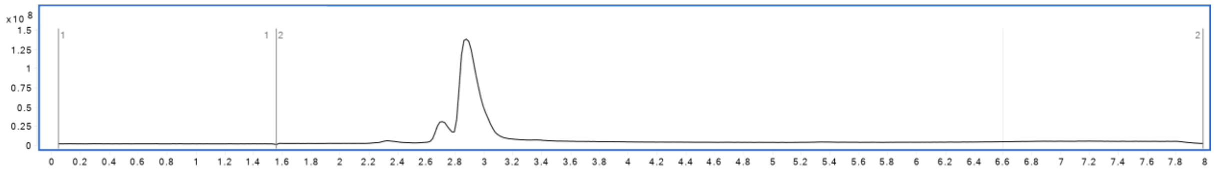

(b)

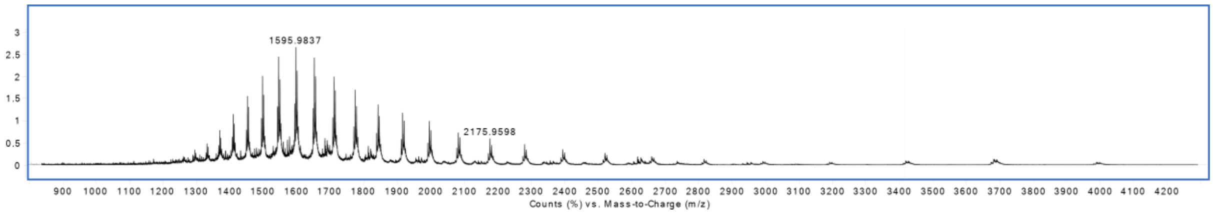

(c)

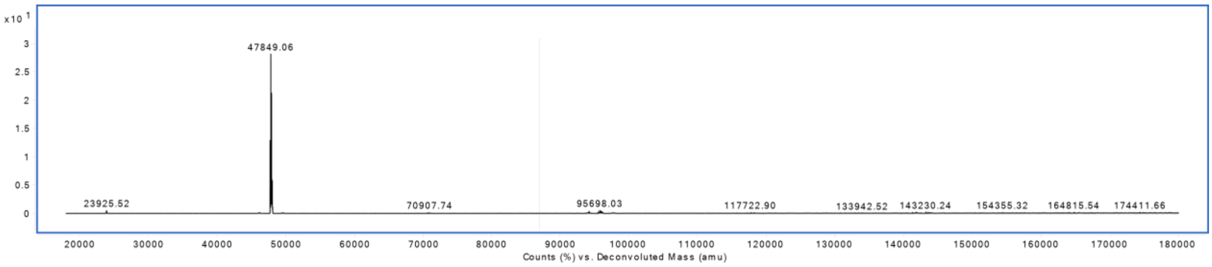

(d)

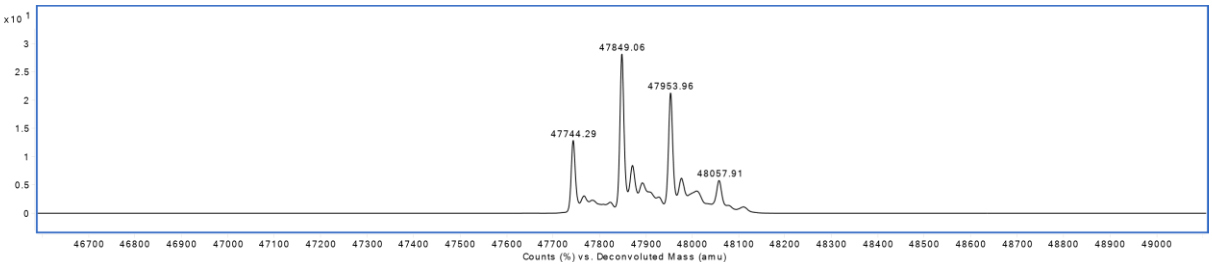

Figure S35: LCMS analysis of conjugate **S7**; a) TIC, b) non-deconvoluted ion-series, c) full range deconvoluted ion series mass spectrum, d) zoomed in deconvoluted ion series mass spectrum; Expected mass of pyrimidine CLT conjugate: 47743 (1 addition), 47848 (2 additions), 47953 (3 additions), 48058 (4 additions). Observed: 47744, 47849, 47954, 48058.

24 hours

(a)

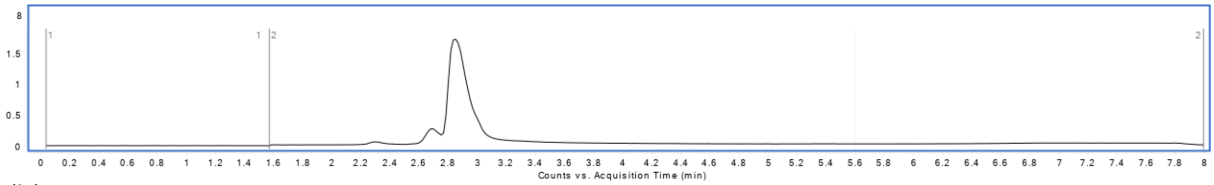

(b)

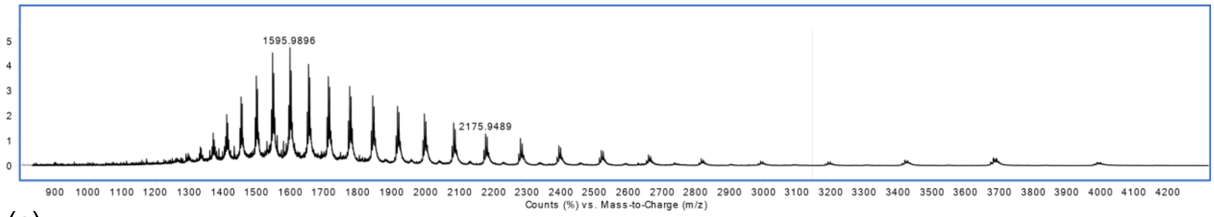

(c)

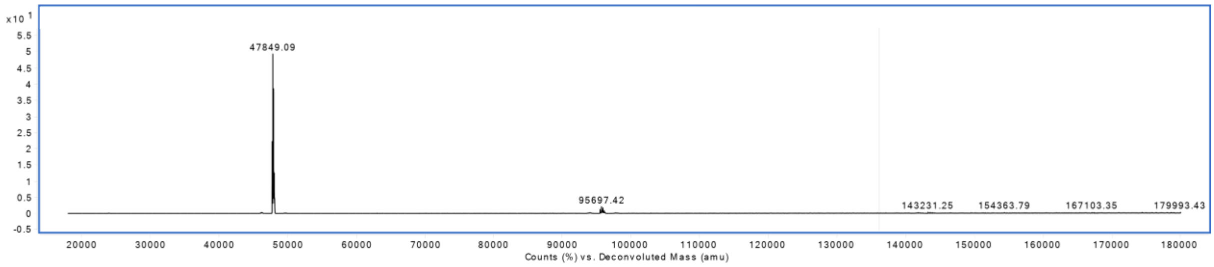

(d)

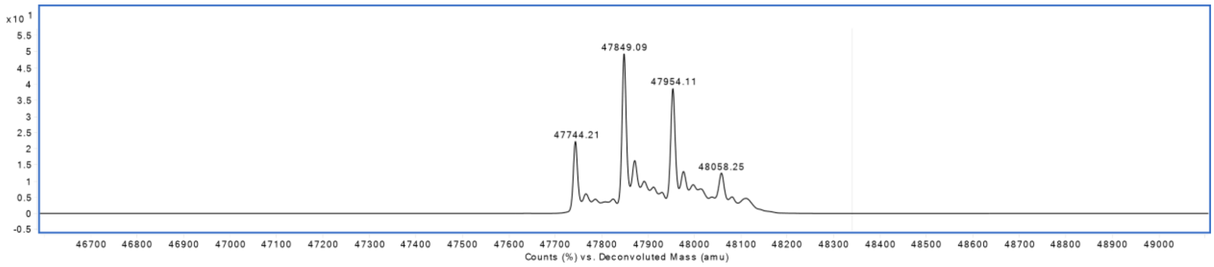

Figure S36: LCMS analysis of conjugate **S7**; a) TIC, b) non-deconvoluted ion-series, c) full range deconvoluted ion series mass spectrum, d) zoomed in deconvoluted ion series mass spectrum; Expected mass of pyrimidine CLT conjugate: 47743 (1 addition), 47848 (2 additions), 47953 (3 additions), 48058 (4 additions). Observed: 47744, 47849, 47954, 48058.

Early endosomal mimicking Glutathione (GSH) concentration

Fab conjugates S16 and S7 (50 µL, 7 µM) were buffer exchanged using desalting columns (7000 MWCO, ZebaSpin, Thermo Scientific) into GSH containing buffer (5 mM GSH, in PBS pH 6.5) and maintained at 37 °C for 24 h. Excess reagents were removed using desalting columns (7000 MWCO, ZebaSpin, Thermo Scientific) prior to LCMS analysis.

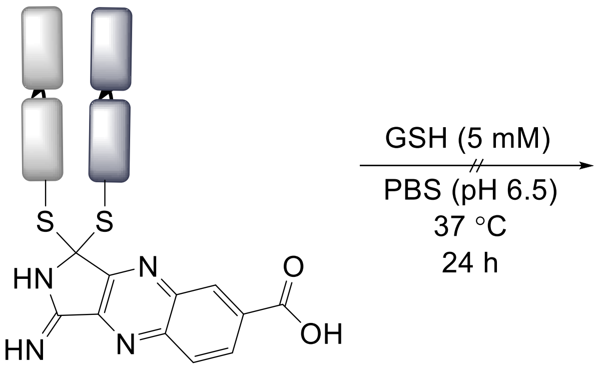

0 hours

(a)

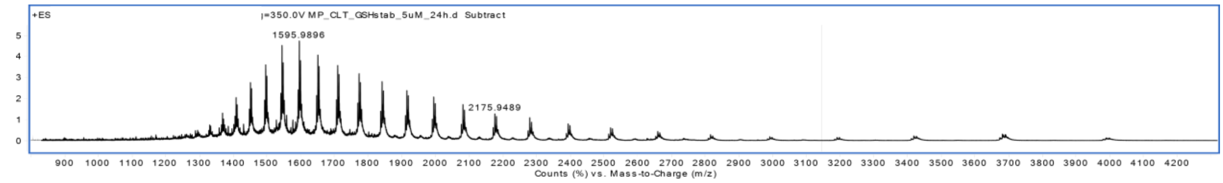

(b)

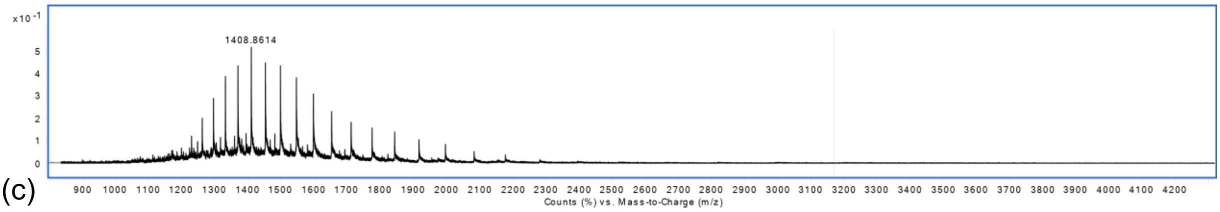

(c)

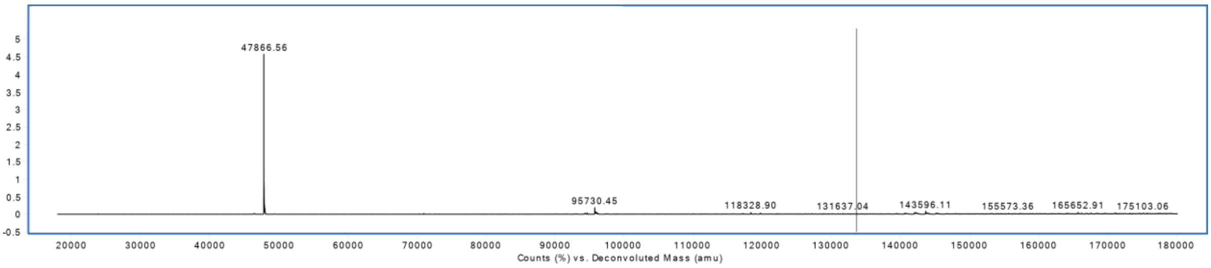

(d)

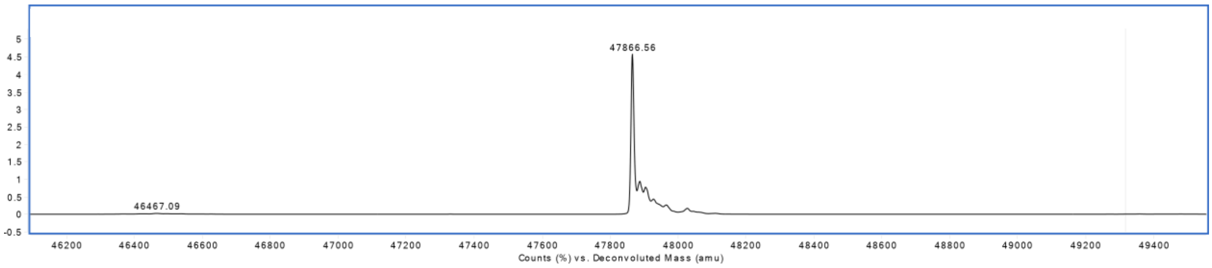

Figure S37: LCMS analysis of conjugate **S16**; a) TIC, b) non-deconvoluted ion-series, c) full range deconvoluted ion series mass spectrum; Expected mass of re-bridged Fab with 2,3-dicyanoquinoxaline-6-carboxylic acid: 47862. Observed: 47866.

24 hours

(a)

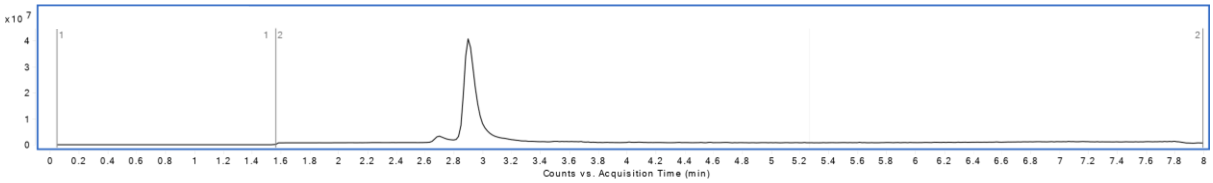

(b)

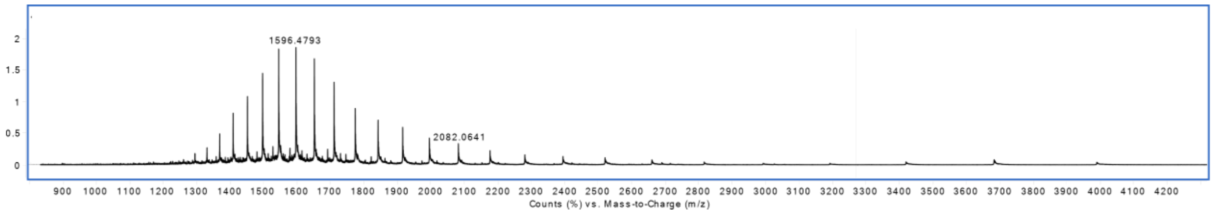

(c)

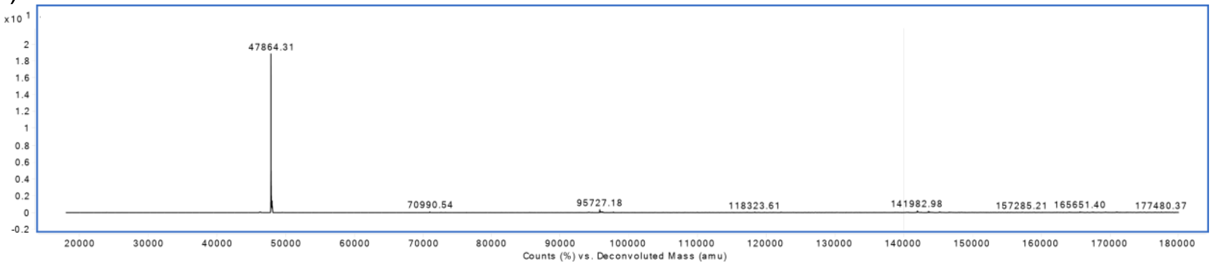

(d)

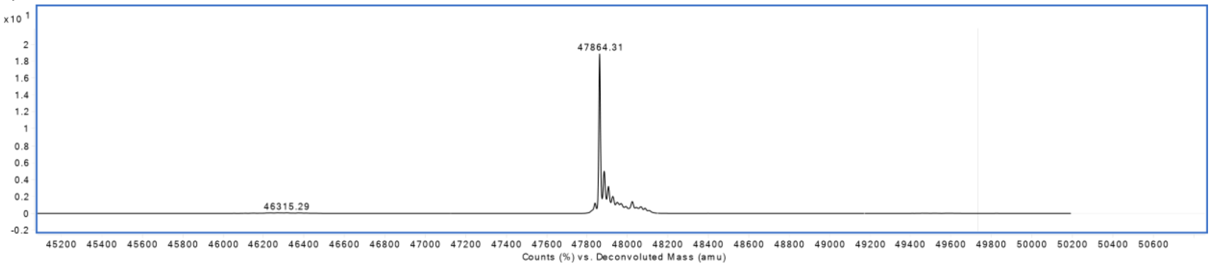

Figure S38: LCMS analysis of conjugate **S16**; a) TIC, b) non-deconvoluted ion-series, c) full range deconvoluted ion series mass spectrum; Expected mass of re-bridged Fab with 2,3-dicyanoquinoxaline-6-carboxylic acid: 47862. Observed: 47864.

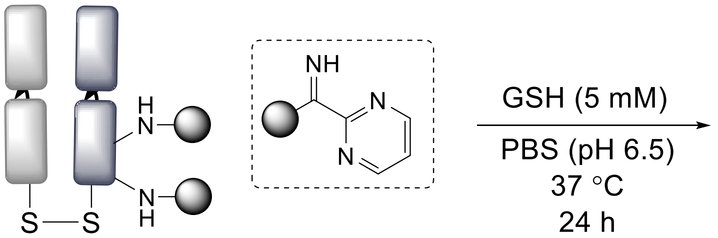

0 hours

(a)

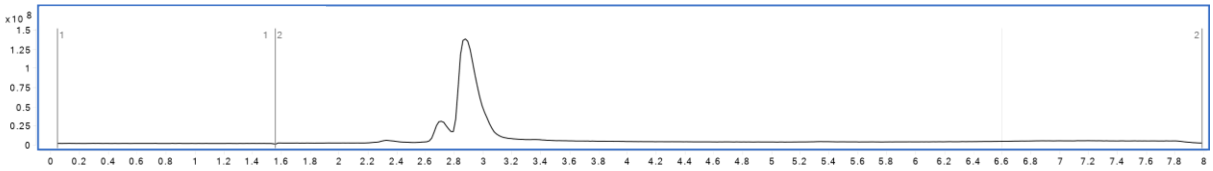

(b)

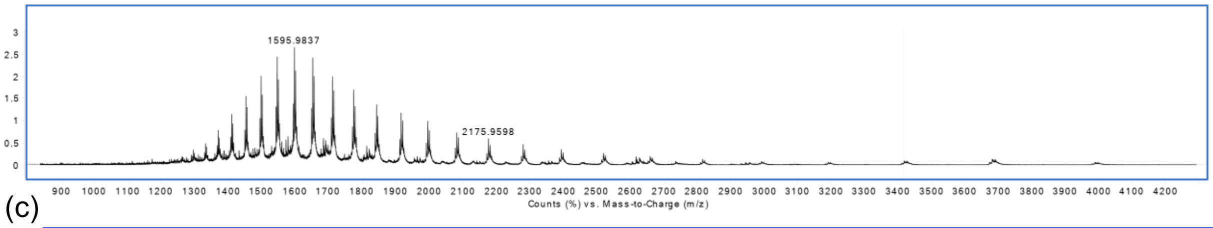

(c)

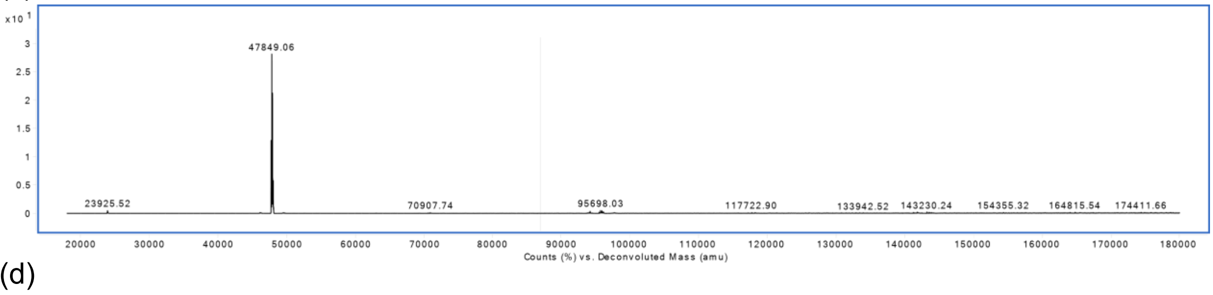

(d)

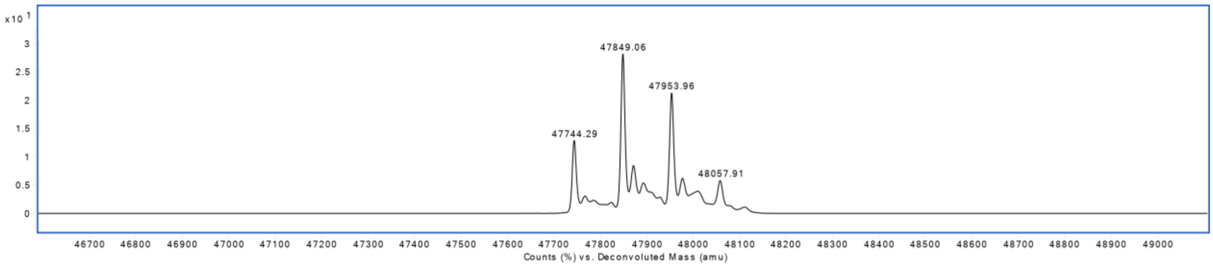

Figure S39: LCMS analysis of conjugate **S7**; a) TIC, b) non-deconvoluted ion-series, c) full range deconvoluted ion series mass spectrum, d) zoomed in deconvoluted ion series mass

spectrum; Expected mass of pyrimidine CLT conjugate: 47743 (1 addition), 47848 (2 additions), 47953 (3 additions), 48058 (4 additions). Observed: 47744, 47849, 47954, 48058.

24 hours

(a)

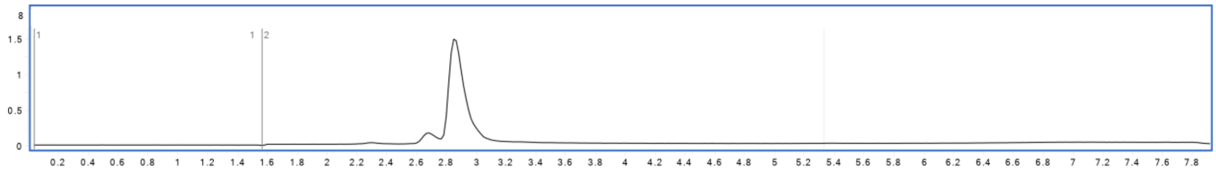

(b)

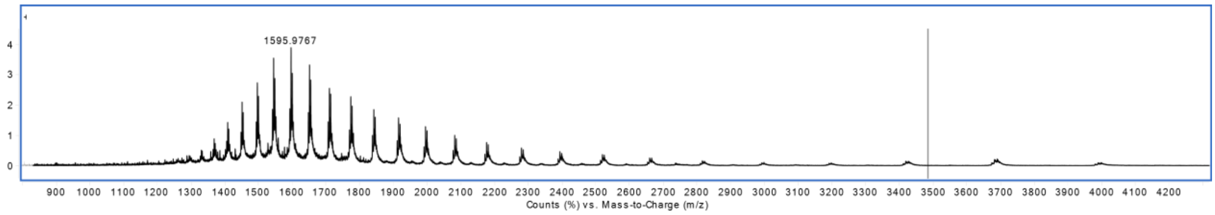

(c)

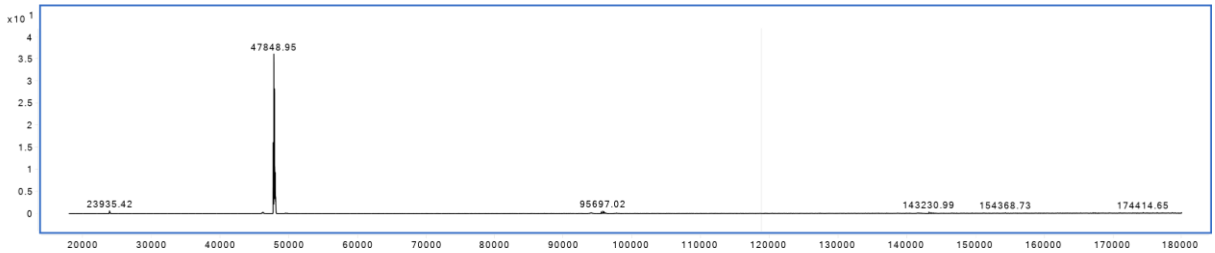

(d)

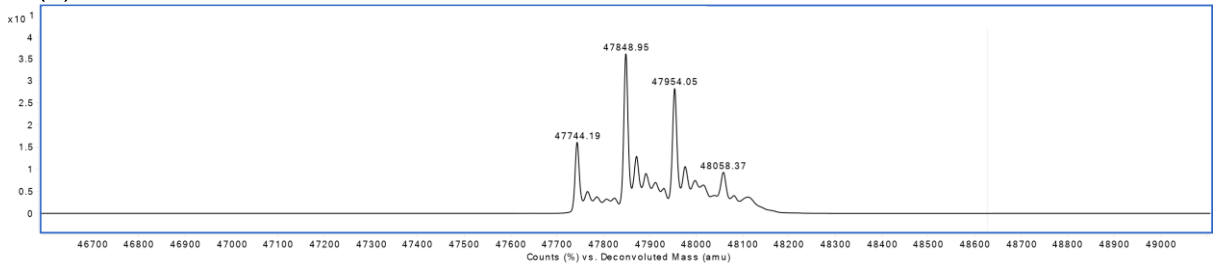

Figure S40: LCMS analysis of conjugate **S7**; a) TIC, b) non-deconvoluted ion-series, c) full range deconvoluted ion series mass spectrum, d) zoomed in deconvoluted ion series mass spectrum; Expected mass of pyrimidine CLT conjugate: 47743 (1 addition), 47848 (2 additions), 47953 (3 additions), 48058 (4 additions). Observed: 47744, 47849, 47954, 48058.

CLT reaction of Fab with 5-(trifluoromethyl)pyrimidine-2-carbonitrile

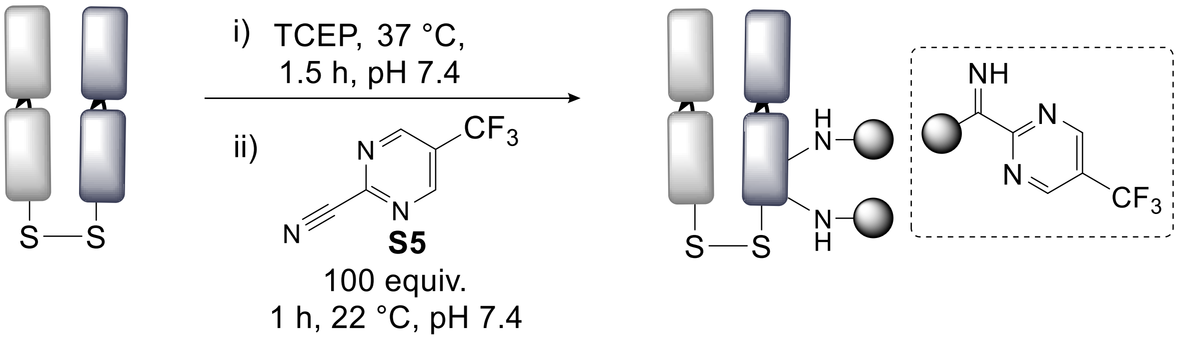

Fab (20 µL, 150 µM, 7.15 mg/mL) in conjugation buffer was reduced with tris(2-carboxyethyl)phosphine (TCEP) (2.0 µL, 15 mM in dH_2O, 10 equiv.) The mixture was incubated at 37 °C for 1.5 h, 300 rpm. 5-(trifluoromethyl)pyrimidine-2-carbonitrile was then added (2.0 µL, 150 mM in DMF, 100 equiv.) was added and incubated at 22 °C for 1 h. Following this DTNB (1.0 µL, 150 mM in EtOH, 50 equiv.) was added and the reaction was left at 22 °C for 10 min. Lastly, sample was desalted into HPLC grade water (7 kDa MWCO, ZebaSpin) prior to LCMS analysis. Concentration was determined photometrically using ε_{280}= 68590 M^{-1} cm^{-1}.

(a)

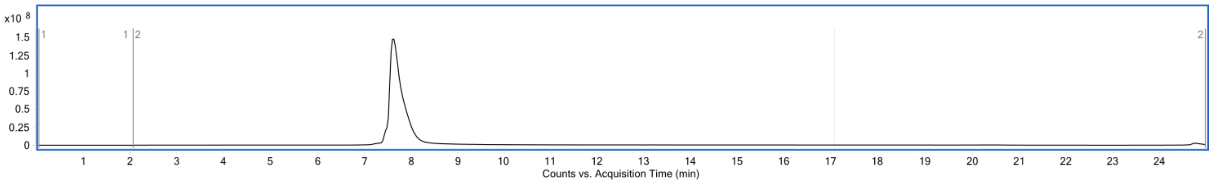

(b)

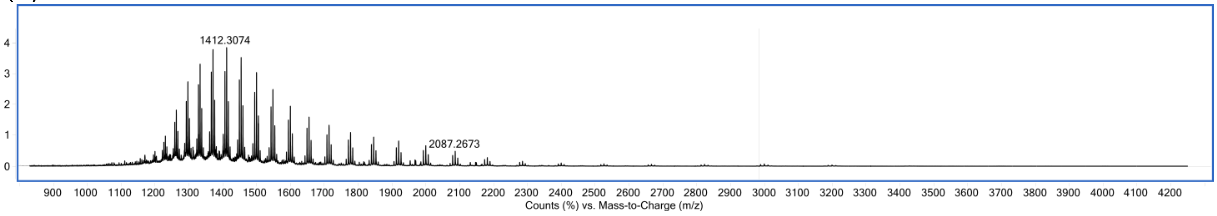

(c)

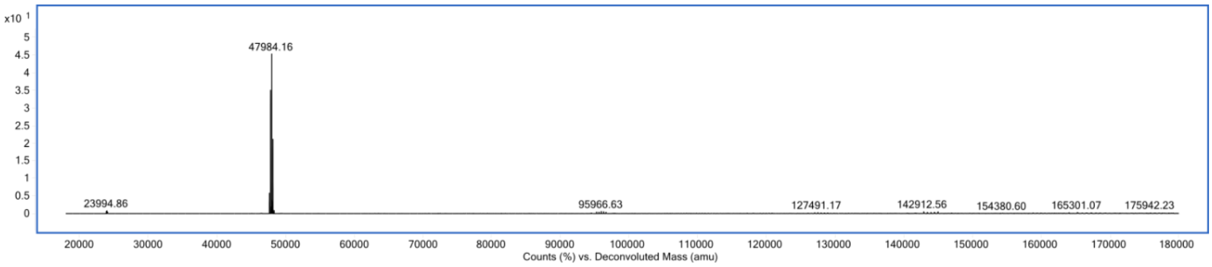

(d)

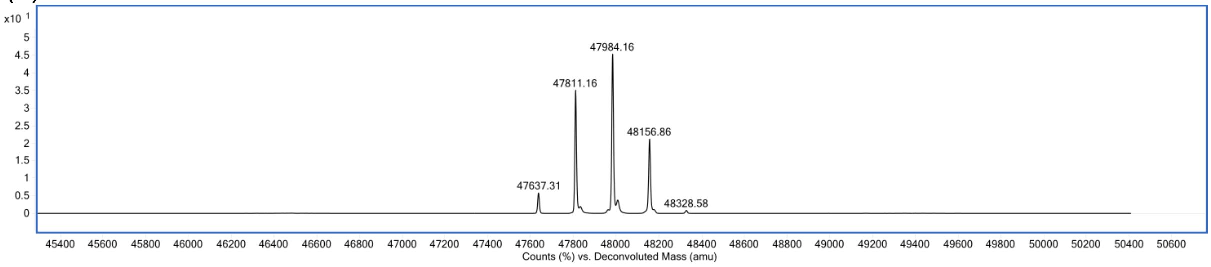

Figure S41: LCMS analysis of Fab CLT conjugate; a) TIC, b) non-deconvoluted ion-series, c) full range deconvoluted ion series mass spectrum, d) zoomed in deconvoluted ion series mass spectrum; Expected mass of CLT conjugate: 47637 Da (native, 0 additions), 47810 Da (1 addition), 47983 Da (2 additions), 48156 Da (3 additions), 48329 Da (4 additions). Observed: 47637 Da, 47811 Da, 47984 Da, 48157 Da, 48329 Da.

Average DAR = 1.8

CLT reaction of Fab with 5,6-Dimethyl-1,2,4-triazine-3-carbonitrile

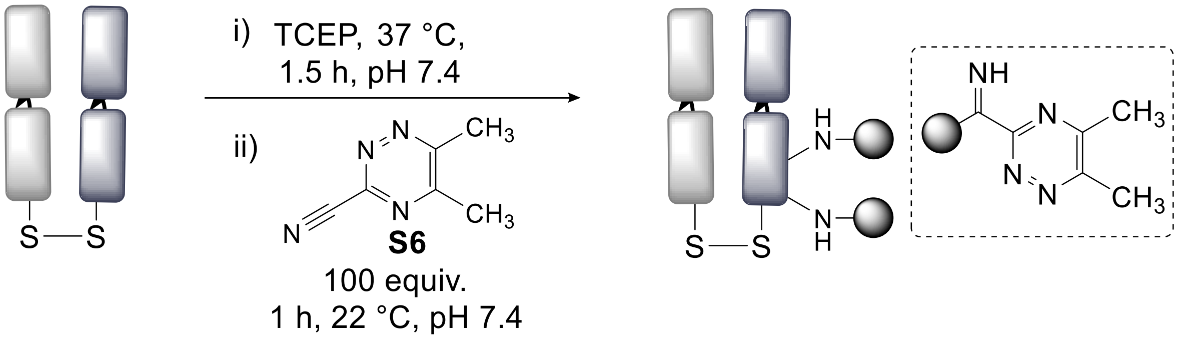

Fab (20 µL, 150 µM, 7.15 mg/mL) in conjugation buffer was reduced with tris(2-carboxyethyl)phosphine (TCEP) (2.0 µL, 15 mM in dH_2O, 10 equiv.) The mixture was incubated at 37 °C for 1.5 h, 300 rpm. 5,6-dimethyl-1,2,4-triazine-3-carbonitrile was then added (2.0 µL, 150 mM in DMF, 100 equiv.) and incubated at 22 °C for 1 h. Following this DTNB (1.0 µL, 150 mM in EtOH, 50 equiv.) was added and the reaction was left at 22 °C for 10 min. Lastly, sample was desalted into HPLC grade water (7 kDa MWCO, ZebaSpin) prior to LCMS analysis. Concentration was determined photometrically using ε_{280}= 68590 M^{-1} cm^{-1}.

(a)

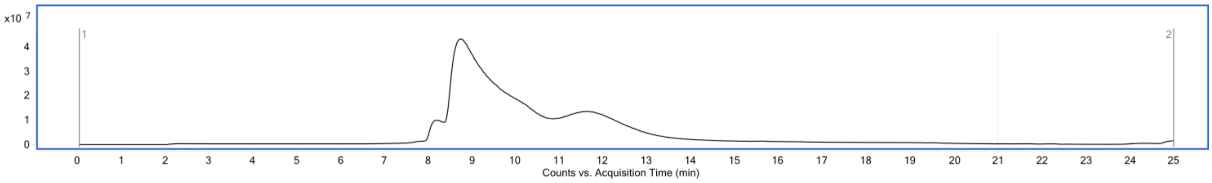

(b)

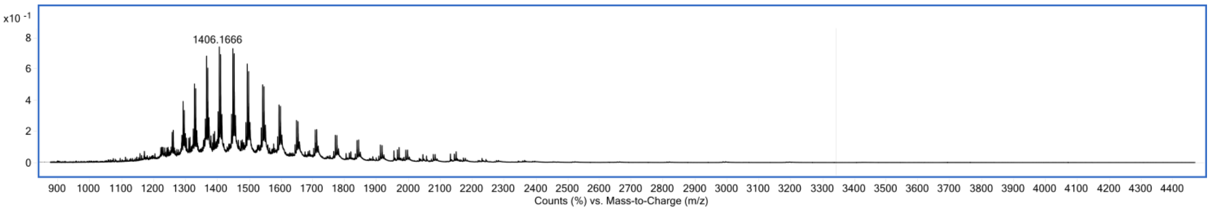

(c)

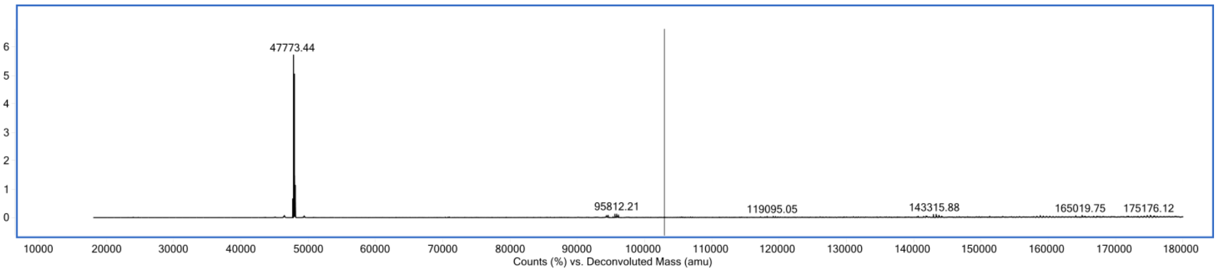

(d)

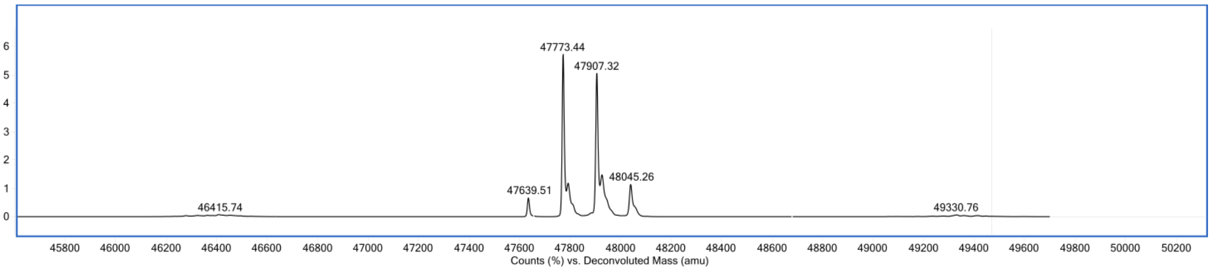

Figure S42: LCMS analysis of Fab CLT conjugate; a) TIC, b) non-deconvoluted ion-series, c) full range deconvoluted ion series mass spectrum, d) zoomed in deconvoluted ion series mass spectrum; Expected mass of CLT conjugate: 47639 Da (native, 0 additions), 47773 Da (1 addition), 47907 Da (2 additions), 48041 Da (3 additions). Observed: 47639 Da, 47773 Da, 47907 Da, 48045 Da.

Average DAR = 1.5

Thioredoxin (Trx)

(a)

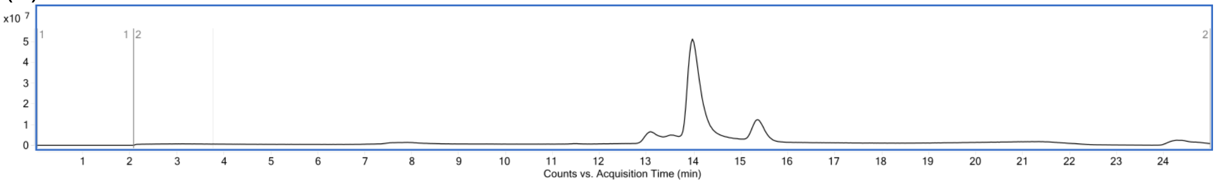

(b)

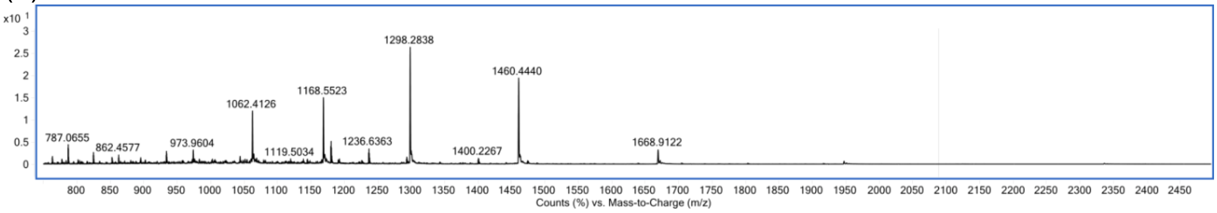

(c)

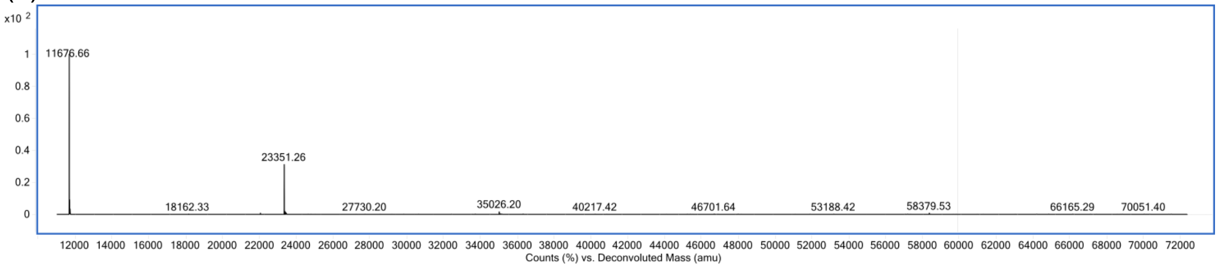

(d)

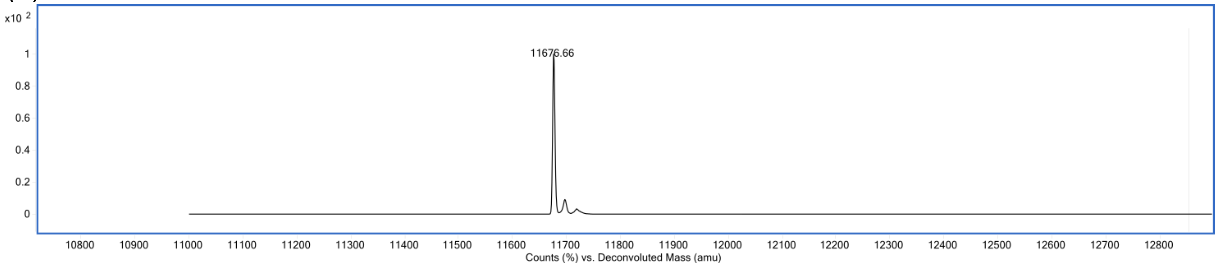

Figure S43: LCMS analysis of conjugate Native Trx; a) TIC, b) non-deconvoluted ion-series, c) full range deconvoluted ion series mass spectrum; native Trx expected 11675 Da. Observed : 11677 Da, Peak at 23351 Da (2 x 11675.5 Da) are proposed to be artifacts formed during ionisation.

Reaction of Trx with pyrazine-2,3-dicarbonitrile

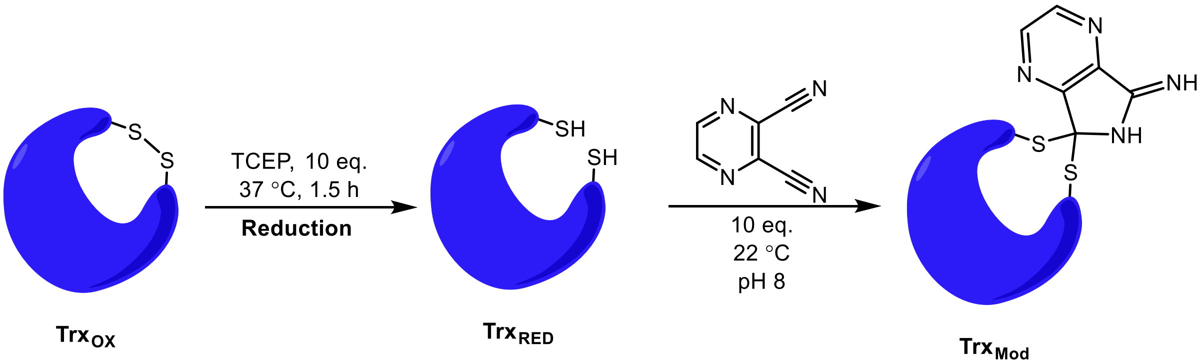

Thioredoxin (30 µL, 100 µM) in BBS (pH 8) was reduced with tris(2-carboxyethyl)phosphine (TCEP) (2.0 µL, 15 mM in dH_2O, 10 equiv.) The mixture was incubated at 37 °C for 1.5 h, 300 rpm. Pyrazine-2,3-dicarbonitrile (2.0 µL, 15 mM in DMF, 10 equiv.) was added and incubated at 22 °C for 2.5 h. Lastly, excess reagent was removed, and the sample was desalted (7 kDa MWCO, ZebaSpin) prior to LCMS analysis. Concentration was determined photometrically using ε280= 14180 $M^{-1}cm^{-1}$.

(a)

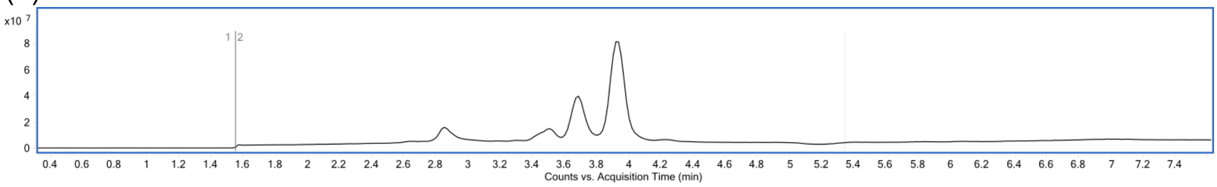

(b)

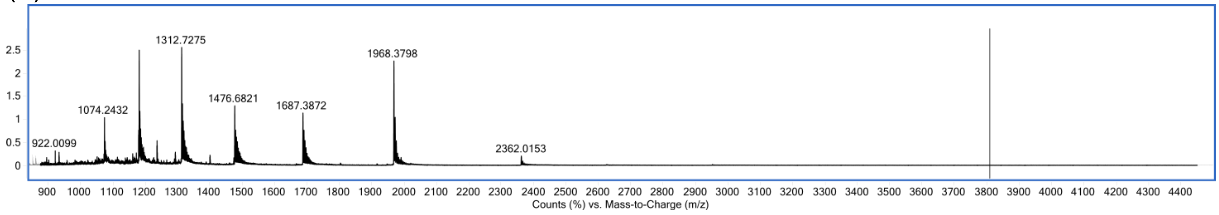

(c)

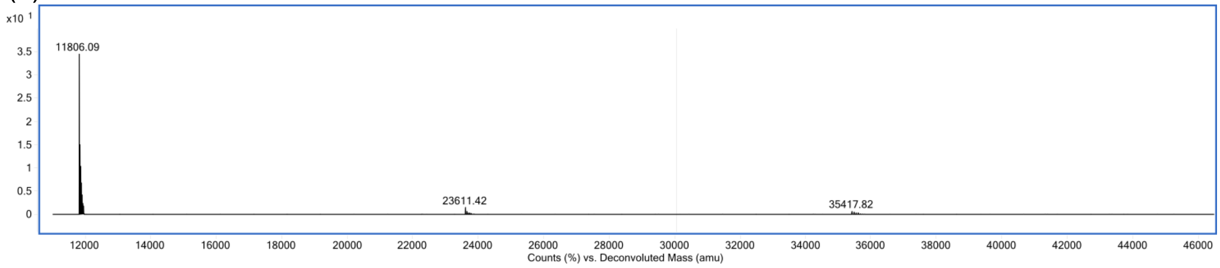

(d)

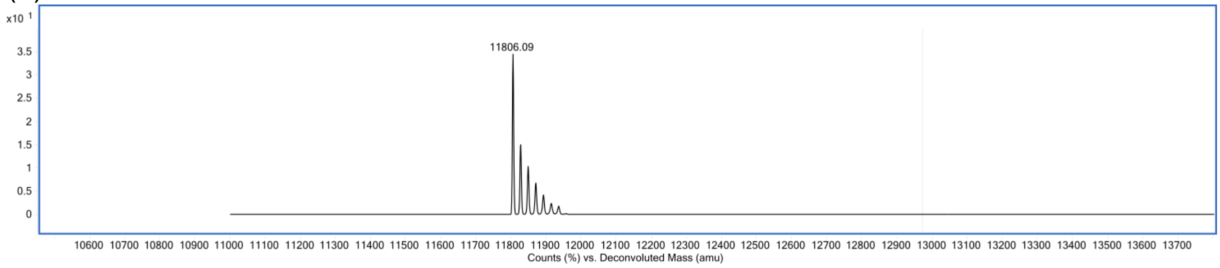

Figure S44: LCMS analysis of modified Trx; a) TIC, b) non-deconvoluted ion-series, c) full range deconvoluted ion series mass spectrum; Pyrazine-2,3-dicarbonitrile modified Trx expected 11805 Da. Observed : 11806 Da.

Serum Stability Study[14]

Fab conjugates 8 and 14 were prepared as 0.2 mg/mL solutions in PBS 140 mM sodium chloride 12 mM sodium phosphates and 2 mM sodium azide at pH 7.4. The conjugates were diluted with 50 % of human blood serum to give a final a concentration of 0.1 mg/mL of 5 or 12 and 1 mM of sodium azide. One aliquot (50 µL) for each conjugate was taken, flash frozen and stored at -20 °C. The remaining solution was incubated at 37 °C under mild shaking (300 rpm) and covered from light. Aliquots (5 µL) were taken at 1, 3, and 5, flash frozen and stored at -20 °C. Samples were diluted with Coomassie Brilliant Blue R-250 (3 µL) and analysed *via* SDS-page (samples were run at 150 V for 80 min). The fluorescent bands were visualised using an AZURE 200® (Epi Blue).

(A) (B)

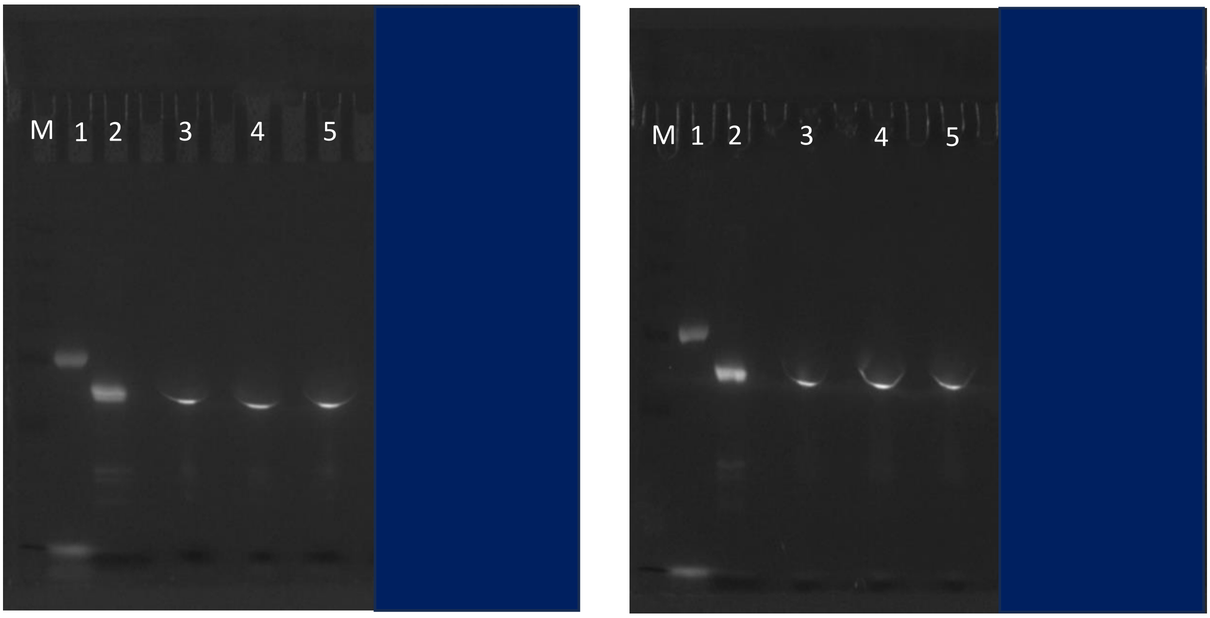

Figure S45: SDS-PAGE of serum stability study; (**A**) M – molecular marker, 1 – fluorescent HSA reference, 2 – fluorescent CLT Fab reference (conjugate 8), 3 – Day one, 4 – Day three, 5 – Day five. (**B**) M – molecular marker, 1 – fluorescent HSA reference, 2 – fluorescent bridged Fab reference (conjugate 14), 3 – Day one, 4 – Day three, 5 – Day five

Enzyme-linked immunosorbent assay (ELISA) – Trastuzumab against HER2

A 96-well plate was coated for 16 h at 4 °C with HER2 (Sino Biological, 100 µL/well, 0.25 µg/mL solution in PBS), all wells except row D and H. After washing (3 × 0.1% Tween® 20 in PBS, followed by 3 × PBS), wells were blocked for 1 h at room temperature with 5% Marvel milk powder (Premier foods) in PBS (200 µL/well). The wells were then washed (3 × 0.1% Tween® 20 in PBS, followed by 3 × PBS), and the following dilutions of Fab conjugate were applied: 810 nM, 270 nM, 90 nM, 30 nM, 10 nM, 3.33 nM, 1.11 nM, 0.37 nM, 0.123 nM, 0.0412 nM, 0.0137 nM, prepared in 1% Marvel solution in PBS (100 µL/well), these were added to rows A to C – 1-11 and E to G – 1-11. The assay was then incubated at room temperature for 1 h. After 1 h, the plate was washed (3 × 0.1% Tween® 20 in PBS, followed by 3 × PBS), and the detection antibody (Anti-Human IgG, Fab specific-horseradish peroxidase (HRP) antibody, Sigma Aldrich, 1:5000 in 1% Marvel solution in 0.1% Tween® 20 in PBS) was added to the whole plate (100 µL/well), and incubated for 1 h at room temperature. After that, the plate was washed (3 × 0.1% Tween® 20 in PBS, followed by 3 × PBS), and o-phenylenediamine hydrochloride (Sigma-Aldrich, 100 µL/well, 0.5 mg/mL in a phosphate-citrate buffer with sodium perborate) was added to the whole plate, left for 15-30 min in dark (monitor the colour development), room temperature. Once a yellow-orange colour was observed, the reaction was stopped by addition of HCl to the whole plate (4 M, 50 µL/well). Absorbance was

immediately measured at 450 nm and was corrected by subtracting the average of negative controls (i.e. PBS had been added to some of the wells instead of HER2 or instead of the 66 samples). Each sample was tested in triplicate and errors are shown as the standard deviation of the average. ELISA data was analysed with Graphpad Prism 7.03 (using equation Sigmoidal, 4PL, X is log(concentration)) and the values have been normalised.

MS/MS analysis (S46)

Fab pymol structure analysis: To analyse the Fab structure, to compare with the MS/MS results, two PDB files were used. 6bae is from a crystal structure of recombinantly produced trastuzumab.[15] It allows clear identification of proximal lysines LC K190 and HC K136. However, it lacks K225 (part of the hinge region, not included in Fab expressions) and K221 is part of a flexible region which is present but lacks resolution. As such PDB file 1HZH, which is a full human 1gG1, against HIV-1, is shown alongside; as this structure has K225 present, (and clearly shows K221). These two PDB structures have been shown to overlay extremely closely in this constant region of the Fab, as expected.[16] Distances are measured from the alpha-carbon of the lysine residues to the alpha-carbon of the proximal cysteine, to get a sense of distances between these residues (noting significant structural flexibility and side-chain mobility prevents accurate measurements). This analysis confirms that HC lysines K136, 221 and 225 and LC 190, are proximal to the interchain disulfide bond.

Figure S47: **A** From PDB file 6bae, of trastuzumab Fab, with proximal lysines HC K136 and LC K190 labelled.

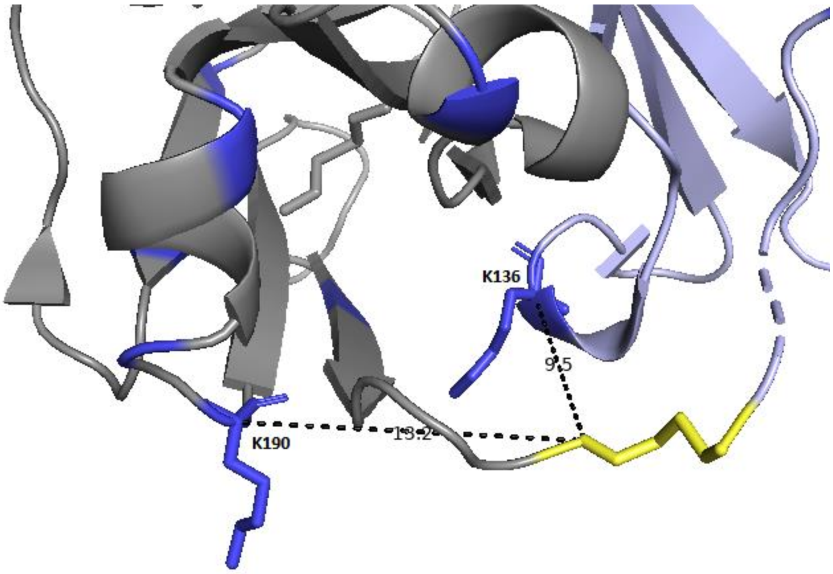

B From PDB file 1HZH, of a full IgG1 against HIV-1, with proximal lysines HC K221, K225 and LC K190 labelled (Note – number of amino-acid residues in this case taken from the trastuzumab sequence, to allow direct comparison).

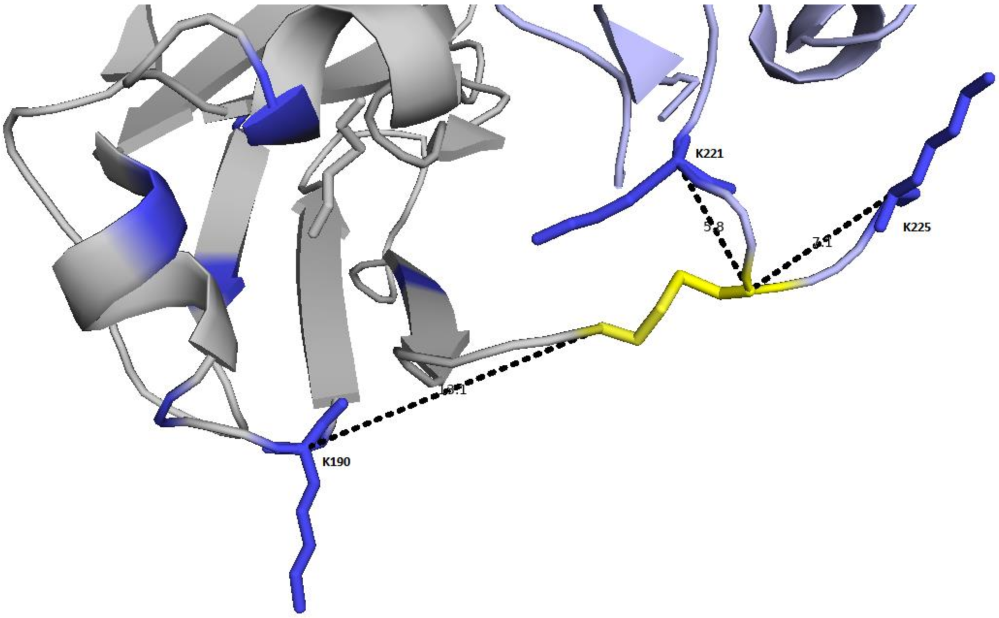

C From PDB file 6bae showing zoom out of full structure:

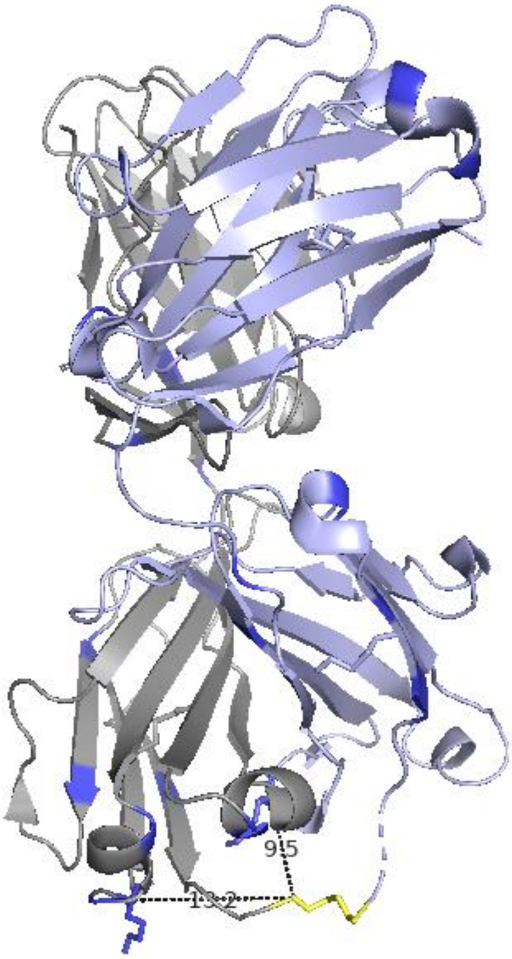

In-solution digestion of FAB and CLT conjugate

For peptide analysis, CLT conjugate was buffer exchanged into H_2O (10 kDa MWCO). The protein concentration was determined by UV/Vis absorbance and adjusted to 450 µM (21.4 mg/mL). 14 µL of the resulting solution were diluted to 70 µL with a solution of 6M Guanidine.HCl and 2 mM EDTA. DTT (3 µL, 100 mM solution in 100 mM Tris buffer, pH 8.0) was then added. After 75 min at 37 ^{o}C, iodoacetamide (6 µL, 100 mM solution in 100 mM Tris buffer, pH 8.0) was added and the mixture was incubated for further 75 min at 37 ^{o}C, in the dark. The reaction was diluted with 280 µL H_2O and 70 µL Tris buffer (50 mM, pH 8.0), before trypsin (Pierce^{TM} Trypsin Protease, MS Grade, 3 µL, 1 mg/mL solution in 10 mM HCl) was added. The resultant mixture was incubated for 16 h at 37 ^{o}C with shaking (300 rpm). After this period, the reaction was centrifuged and stopped by the addition of TFA (2.2 µL). The peptide mixture was then purified using a C18 cartridge (Sep-Pak C18, 360 mg sorbent, Waters, UK). The cartridge was washed with 1 mL of acetonitrile, 0.1% formic acid solution followed by 1.5 mL of water, 0.1% formic acid solution The sample was then loaded on the cartridge and peptides were eluted with 3.0 mL of 30% H_2O, 70% MeCN, 0.1% formic acid solution. The solvent was then evaporated using a Speedvac concentrator. The peptides were then re-dissolved in 150 µL of 5% acetonitrile 95% water, 0.2% formic acid and analysed using a capillary LC-MS/MS instrument.

Capillary LC-MS/MS analysis

The LC-MS/MS system consisted of a Q Exactive mass spectrometer equipped with a HESI probe coupled to a Vanquisher UHPLC system (Thermo Fisher Scientific, UK). Chromatographic separation of peptides was achieved on a Hypersil Gold C18 (100 mm x 2.1 mm, 1.9 µm particles, P/N 25002-101130) column from Thermo Fisher Scientific, UK. Mobile phase A consisted of water in 0.1% formic acid and Mobile phase B consisted of acetonitrile in 0.1% formic acid. The gradient was as follows: - at 0 min B was at 5% for 2 min then the proportion of B% was increased linearly to 40% over 35 min and then to 95% B in 6 sec and held at 95% B for a further 4.9 min to allow elution of more hydrophobic peptides from the C18 column. This followed by sudden change in 6 sec to 5% B so the column was finally re-equilibrated for 4.9 min at 95% A and 5% B prior to the next injection. The LC-MS/MS analysis time was 50 min. The flow rate was 0.2 mL/min. Ten µL of peptide mixture was injected on the C18 column giving the total amount of peptides injected around 19.9 µg per analysis. The effluent for the C18 column was directed to the HESI ion source. The HESI source was operated as follows:- source voltage 3.5 kV, capillary temperature 320˚C, sheath gas 25, auxiliary gas 5 and probe heater 180˚C and operated in positive ionisation mode. The Full MS-

ddTop8 HCD method was set for the identification, profile data was collected in the full MS scan and data dependent MS/MS modes. One micro-scan for full MS and MS/MS were performed with the maximum injection time of 100 ms and 200 ms, respectively. Automatic gain control (AGC) was in operation. Over the 50 min run time 9 events were repeated: first a full MS scan was recorded over the *m/z* range 380-1500, followed by MS/MS spectra of the eight most intense ions in the full spectrum. The collision energy was 30%. The minimum precursor-ion signal required for MS/MS data collection the intensity threshold was set to $1e^5$ cps, the isolation width was 1.4 Th, the dynamic exclusion 20 sec. The resolution for full MS scan was 70,000 and for MS/MS 17,500, and multiple charges states was all, with peptide match preferred.

Sequence coverage of CLT conjugate S7 by trypsin digestion

>sp Heavy Chain

1 EVQLVESGGG LVQPGGSLRL SCAASGFNIK DTYIHWVRQA PGKGLEWVAR IYPTNGYTRY ADSVKGRFTI SADTSKNTAY **80**

81 LQMNSLRAED TAVYYCSRWG GDGFYAMDYW GQGTLVTVSS ASTKGPSVFP LAPSSKSTSG GTAALGCLVK DYFPEPVTVS **160**

161 WNSGALTSGV HTFPAVLQSS GLYSLSSVVT VPSSSLGTQT YICNVNHKPS NTKVDKKVEP KSCDKTH **227**

>sp Light Chain

1 DIQMTQSPSS LSASVGDRVT ITCRASQDVN TAVAWYQQKP GKAPKLLIYS ASFLYSGVPS RFSGSRSGTD FTLTISSLQP **80**

81 EDFATYYCQQ HYTTPPTFGQ GTKVEIKRTV AAPSVFIFPP SDEQLKSGTA SVVCLLNNFY PREAKVQWKV DNALQSGNSQ **160**

161 ESVTEQDSKD STYSLSSTLT LSKADYEKHK VYACEVTHQG LSSPVTKSFN RGEC **214**

Sequence Coverage Map

Created on 11/14/23 by charl
Data Folder = C:\Users\charl\OneDrive - University College London\Documents\PhD\4. Mass Spec\MSMS\
Minimum MS Signal = 128000
Data File = CLT.raw
Protease = Trypsin

Proteins	Number of MS Peaks	MS Peak Area	Sequence Coverage	Abundance (mol)
1:sp Heavy Chain	411	30.1%	100.0%	51.39%
2:sp Light Chain	344	36.6%	100.0%	48.61%
Unidentified	3553	33.3%		

Minimum Recovery = 1%
Minimum Recovery of Overlapping Peptides = 0%
Minimum Confidence = 80
Maximum Mass = 7000

Color code for peptide recovery

>50.0%	>20.0%	>10.0%	>5.0%	>2.0%	>1.0%	>0.5%	>0.2%	>0.1%	>0.0%
good			fair			poor			

sp Heavy Chain

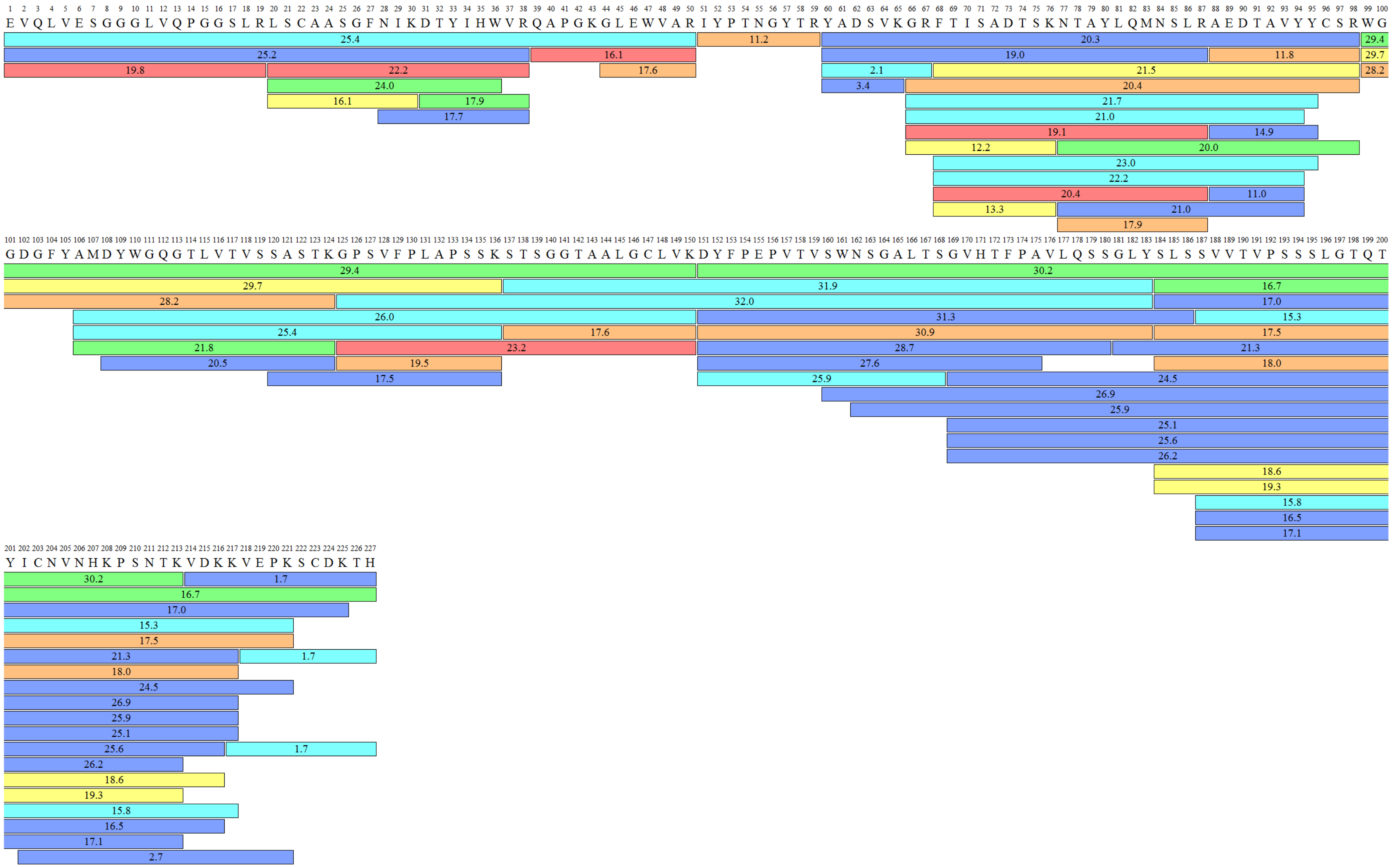

sp Light Chain

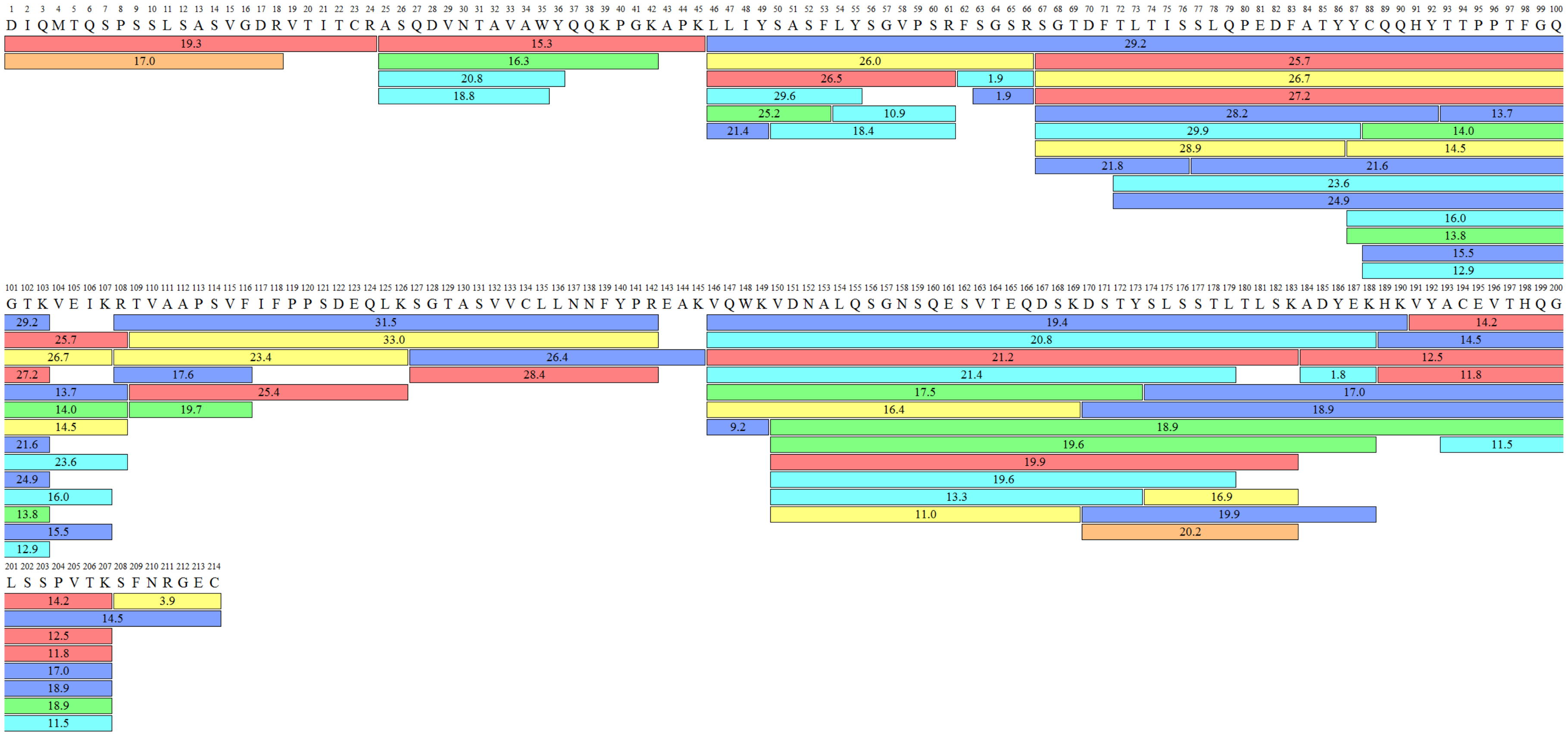

Modification summary table

Protease	Peptide Minimum Intensity (%)	Charge State Minimum Intensity (%)	Minimum Modification Level (%)	Relative Load	Peptide Map Quality
Trypsin	17	33	0.0005	100%	0.496672

Protein	Residue	Modification	Confidence	% Recovery	% Abundance CLT
sp Heavy Chain	136	+105	100	126.6	53.63660431
sp Heavy Chain	221	+105	100	40.2	48.44272232
sp Heavy Chain	225	+105	100	0.3	— [a]
sp Light Chain	190	+105	100	132.2	17.13671303

[a] % Abundance is uncertain due to low recovery of both modified and modified peptides.

100% sequence coverage of LC and HC was obtained, with 100% coverage of lysine residues. Abundance of modifications is shown above on K136, K221 (HC) and K190 (LC). K225 (HC) was also found to be modified, although the abundance of modification was unable to be obtained due to its small size and terminal position, leading to poor recovery of the peptide (< 0.5%)

K136 Modification

GPSVFPLAPSSKSTSGGTAALGULVK(K12+K105) (2+)

Average Structural Resolution = 1.3 residues

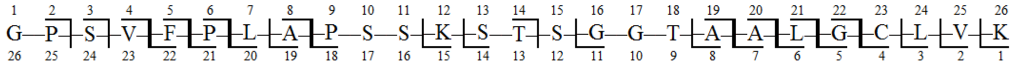

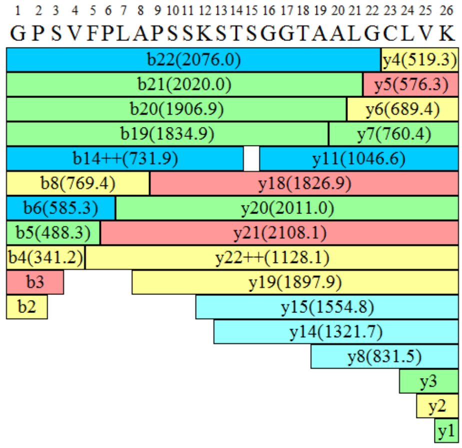

Color Code for Ion Intensity

>4.7e+04	>2.2e+04	>1.0e+04	>4.9e+03	>2.3e+03

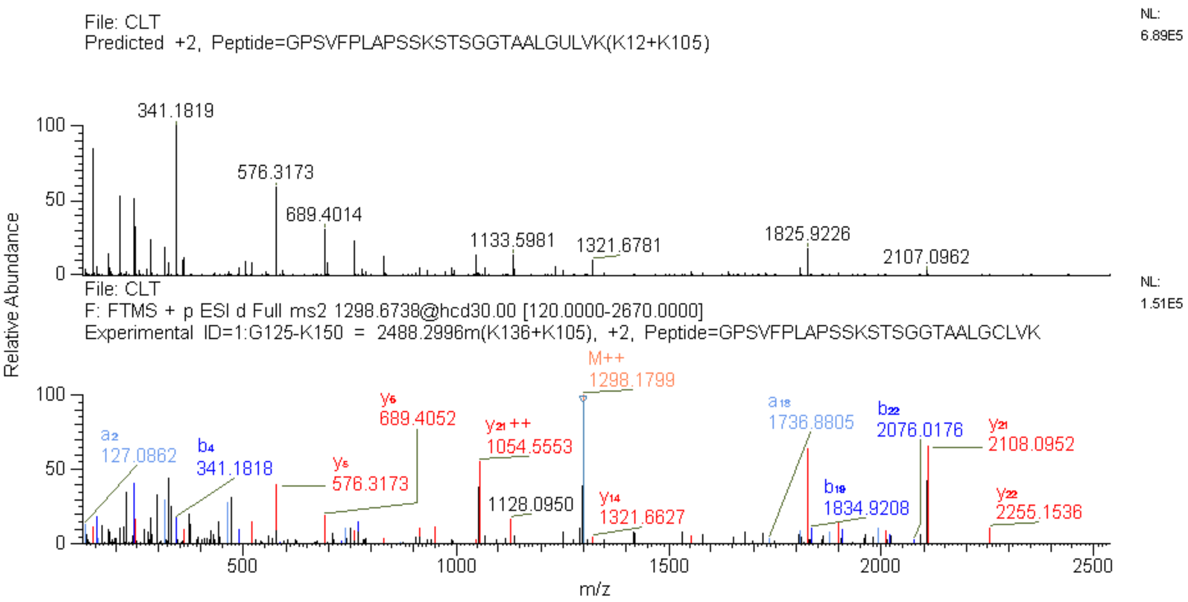

K221 Modification

VEPKSUDK(K4+K105) (2+)

Average Structural Resolution = 1.1 residues

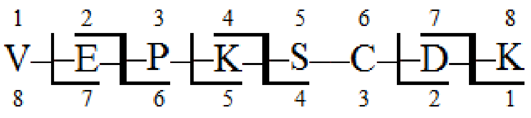

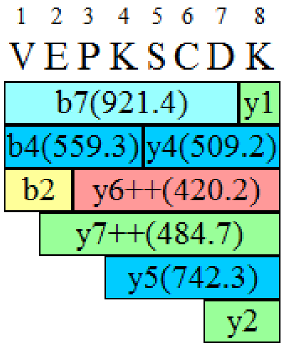

Color Code for Ion Intensity

>4.8e+04	>2.4e+04	>1.2e+04	>6.2e+03	>3.2e+03

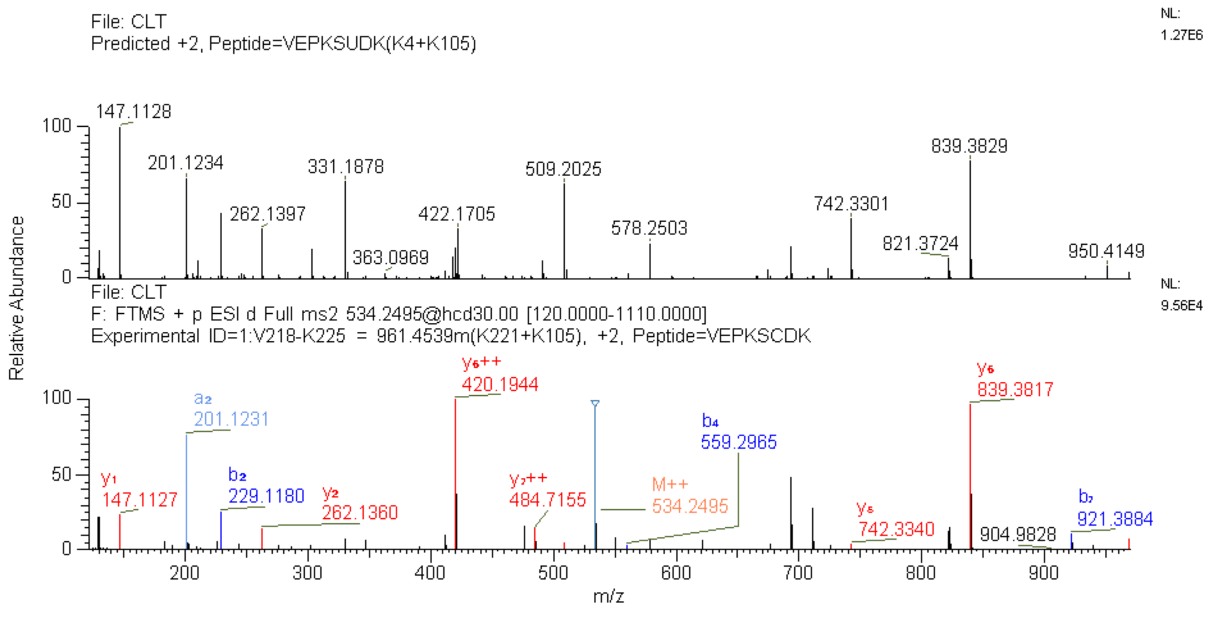

K190 Modification

HKVYAUEVTHQGLSSPVTK(K2+K105) (3+)

Average Structural Resolution = 1.1 residues

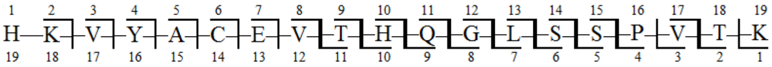

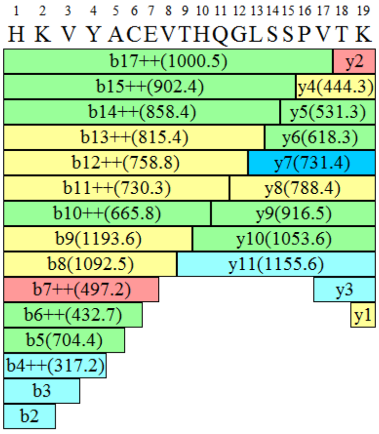

Color Code for Ion Intensity

>6.7e+04	>2.9e+04	>1.3e+04	>5.6e+03	>2.4e+03

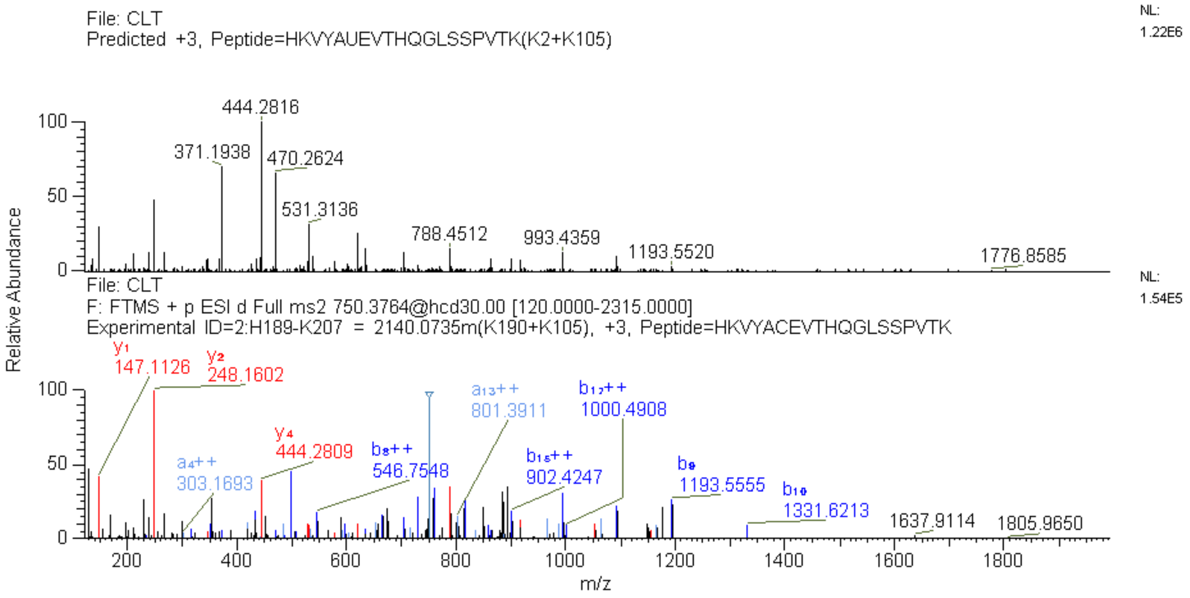

K225

Fragment Coverage Map

SUDKTH(K4+K105) (2+)

Average Structural Resolution = 1.0 residues

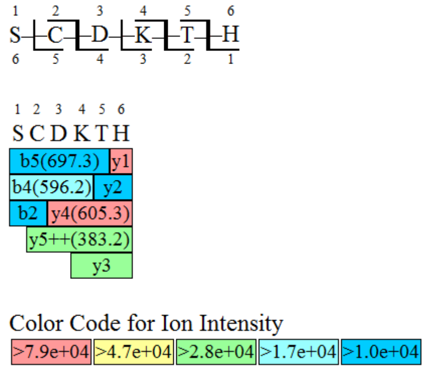

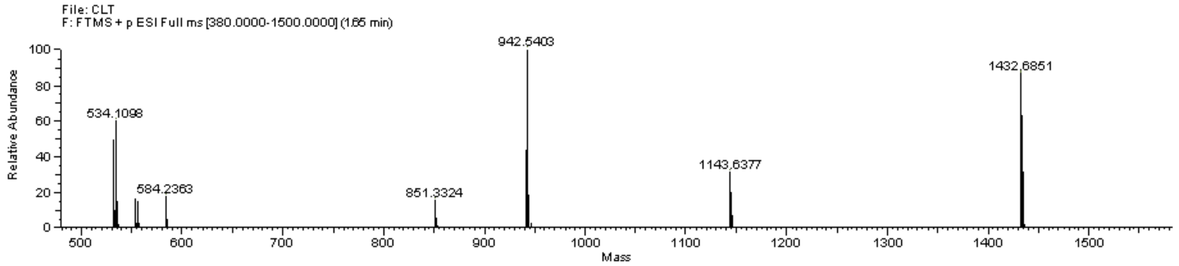

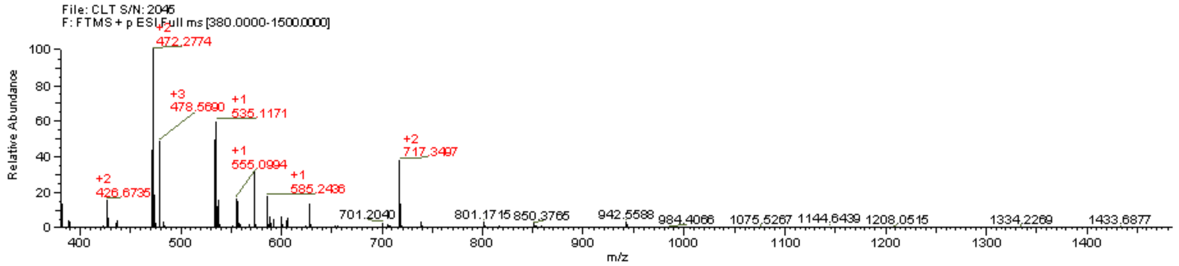

Fab conjugate **8** SEC trace[14]

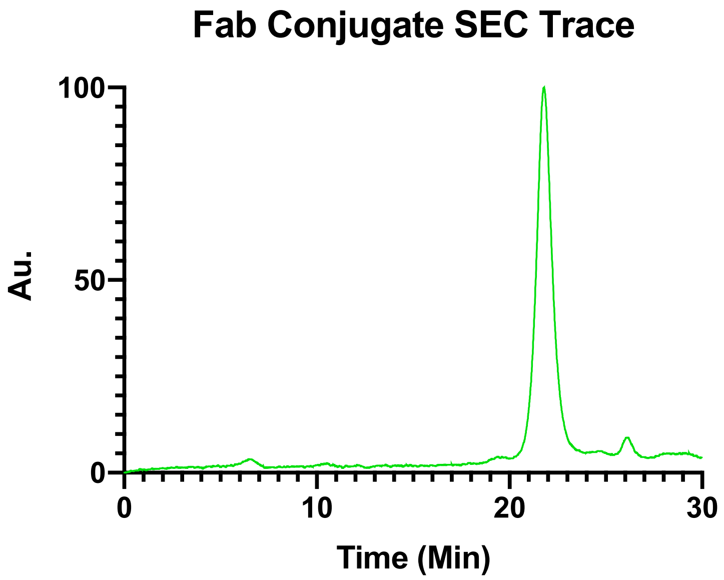

Figure S48: Fluorescent SEC trace of CLT conjugate **8**. Samples (20 μL) of diluted aliquots were analysed by SEC-HPLC on a TSK gel G3000SWXL (7.8 mm x 30 cm) column connected to an Agilent 1200 HPLC system equipped with a 1200 series diode array detector and a fluorescence detector. Samples were eluted using PBS 140 mM NaCl, 100 mM sodium phosphates and 0.02 % sodium azide at pH 7.0 as mobile phase at a flow rate of 0.5 mL/min. over 30 min.

NBT rate studies (S49)

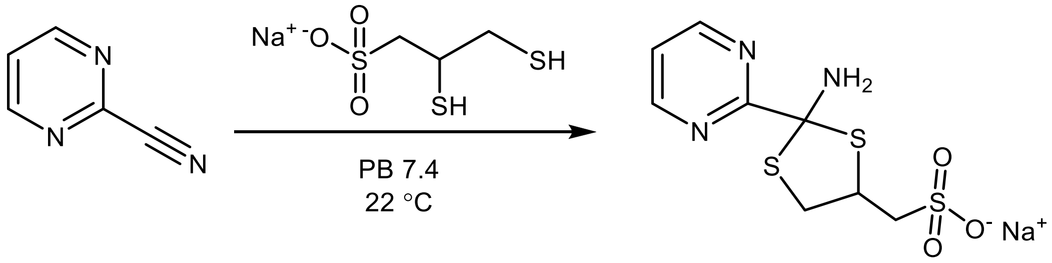

2-pyrimidine carbonitrile (**1**) (10.5 mg) was dissolved in acetonitrile (20 mL) to create a 5 mM stock solution. A stock solution of varying equivalence of sodium 2,3-dimercaptopropane sulfonate (DMPS) in phosphate buffer PB (8 mL, pH 7.4, 0.25 M) was made. The stock of **1** was added (2 mL) to the DMPS stock to give **1** at 1 mM in 10 mL with either 7, 10 or 15 equiv. DMPS. An aliquot of this was taken and submitted for NMR analysis at different time points.

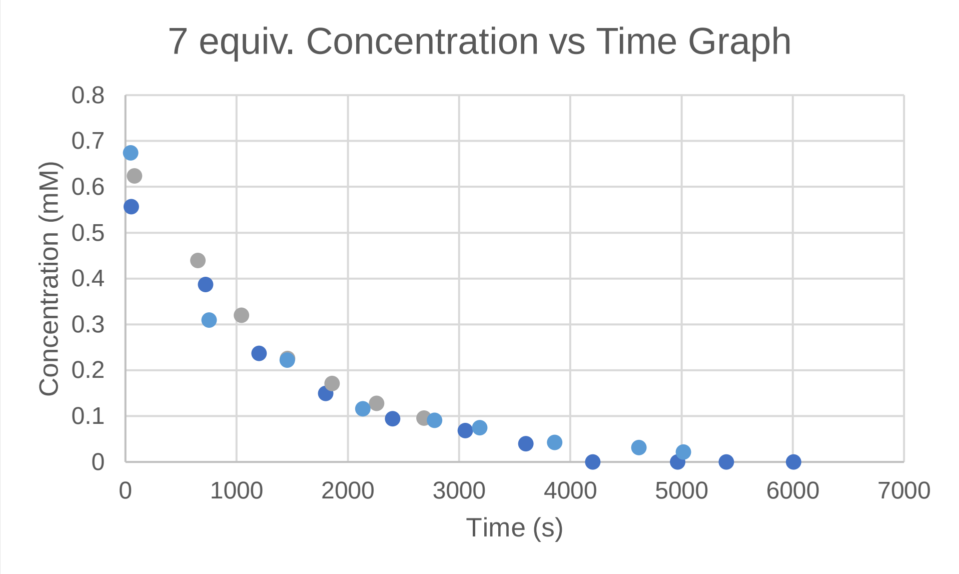

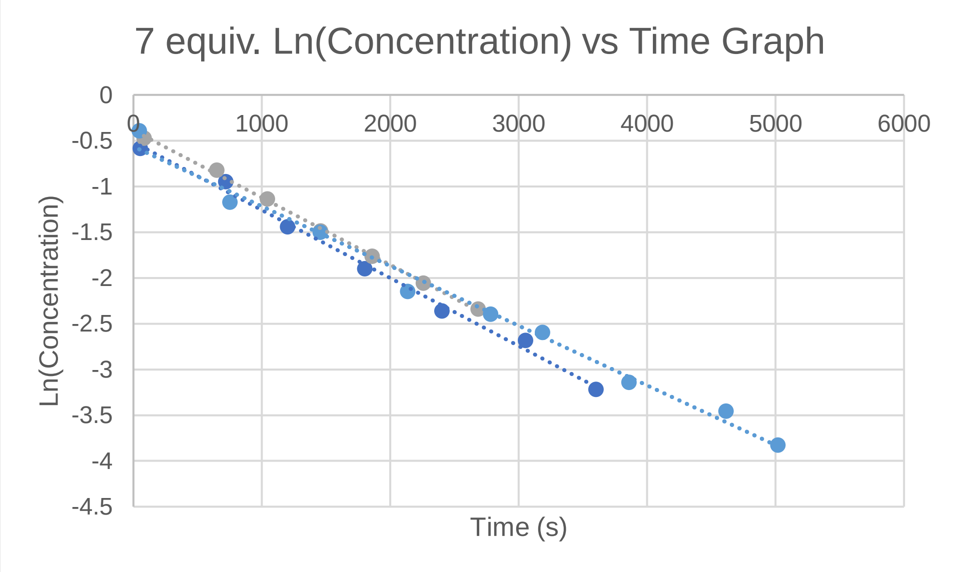

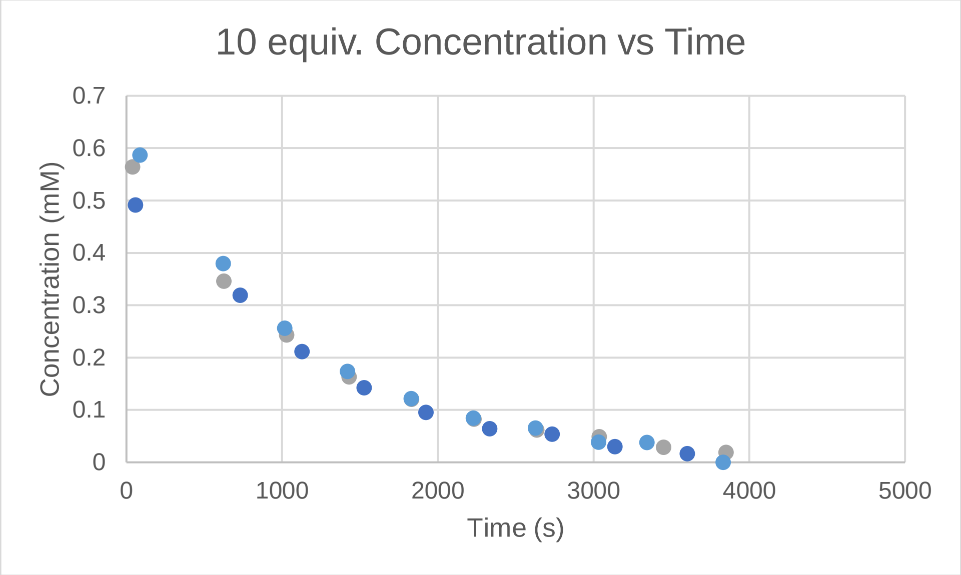
10 equiv. Concentration vs Time
Concentration (mM)
0.7
0.6
0.5
0.4
0.3
0.2
0.1
0
0
1000
2000
3000
4000
5000
Time (s)

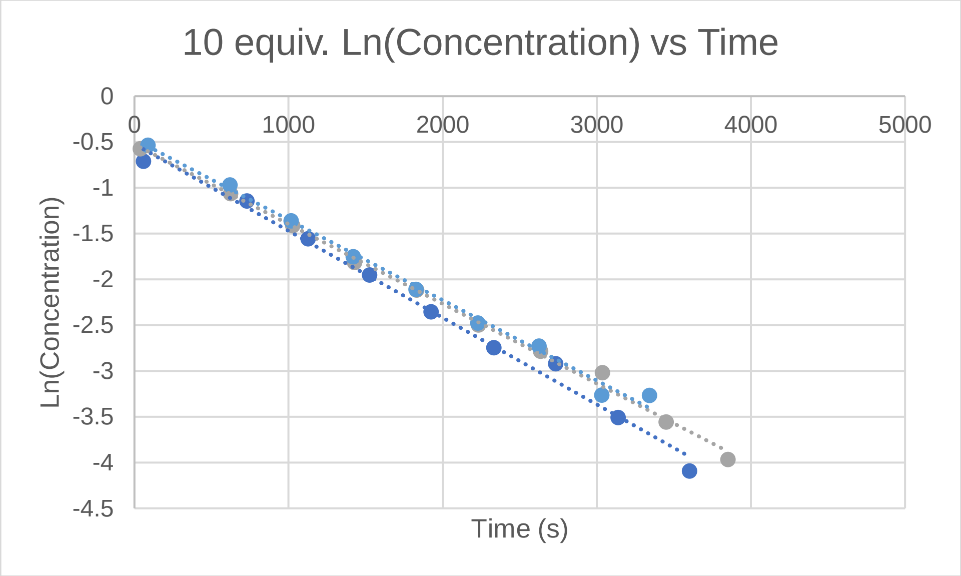
10 equiv. Ln(Concentration) vs Time
Ln(Concentration)
0
-0.5
-1
-1.5
-2
-2.5
-3
-3.5
-4
-4.5
0
1000
2000
3000
4000
5000
Time (s)

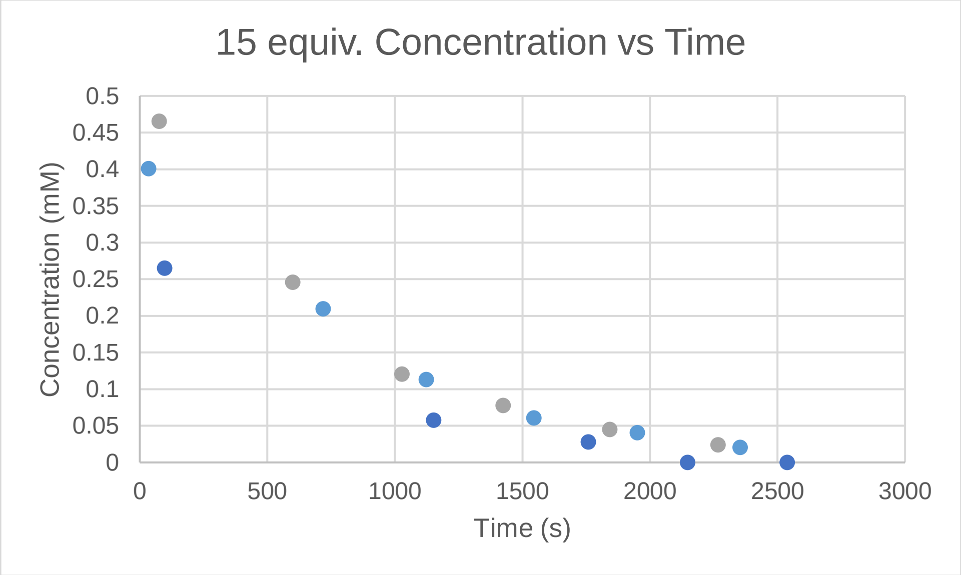
15 equiv. Concentration vs Time
Concentration (mM)
0.5
0.45
0.4
0.35
0.3
0.25
0.2
0.15
0.1
0.05
0
0
500
1000
1500
2000
2500
3000
Time (s)

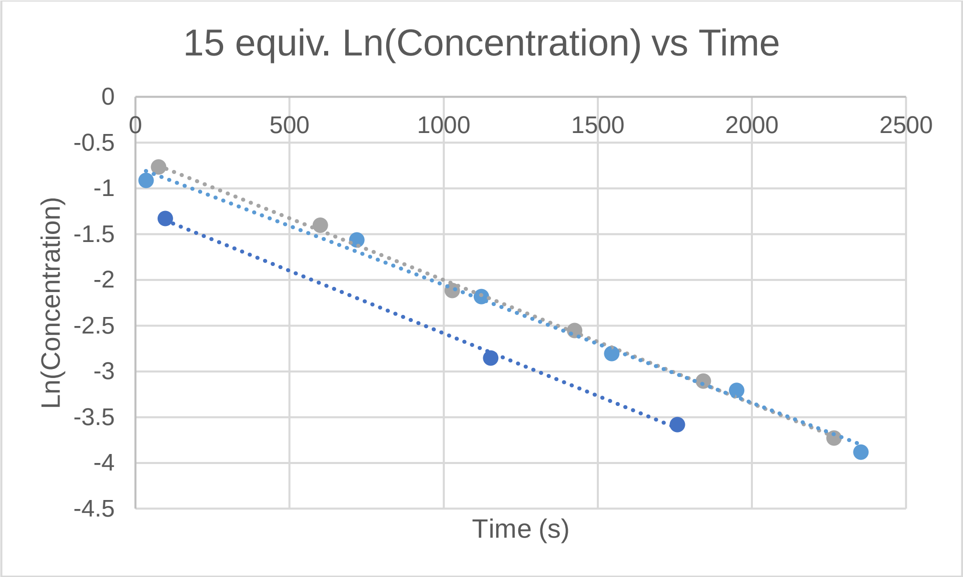
15 equiv. Ln(Concentration) vs Time
0
-0.5
-1
-1.5
-2
-2.5
-3
-3.5
-4
-4.5
0
500
1000
1500
2000
2500
Ln(Concentration)
Time (s)

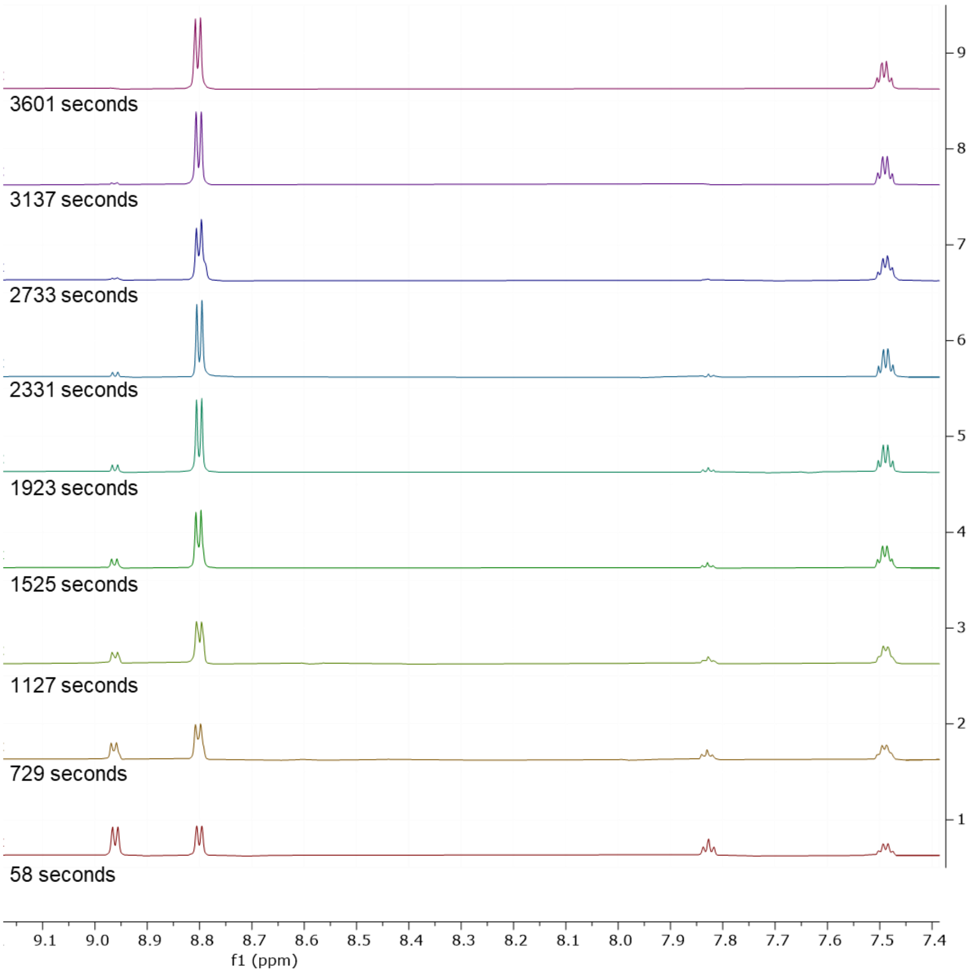

10 equiv. reaction example timepoints (seconds)

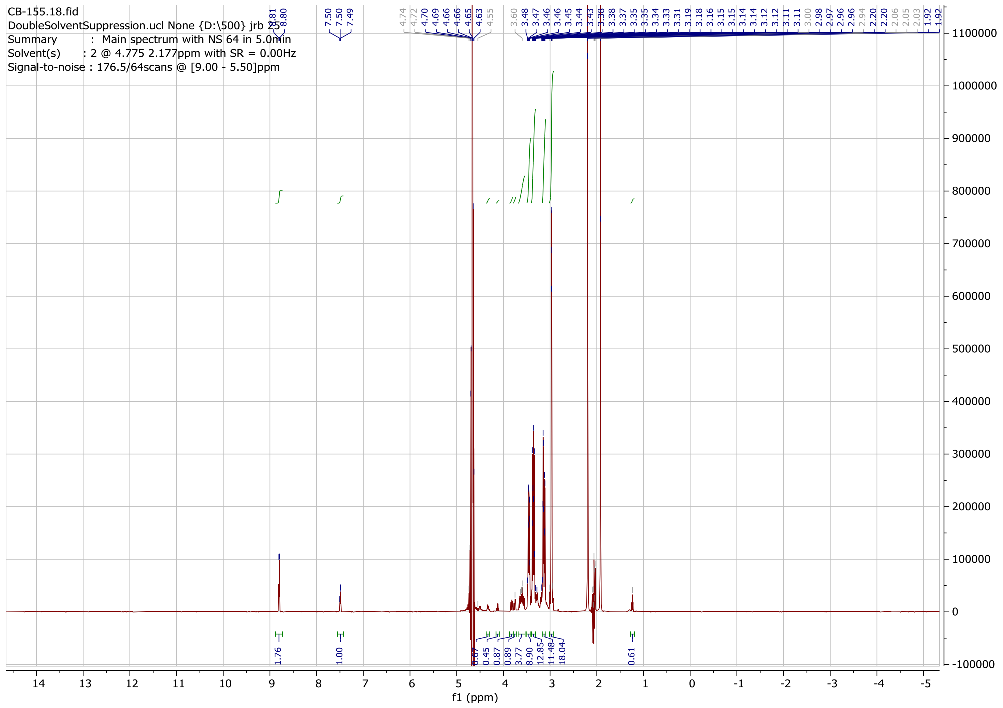

Full NMR of the completed NBT rate reaction (10 equiv.).

Pseudo-First Order Rate Constant

Experiment	Equivalence (eq.)	Rate (k_{obs}) (s^{-1})	Error
1	7	0.743	0.025
2	7	0.734	0.013
3	7	0.653	0.026
4	10	0.947	0.034
5	10	0.870	0.018
6	10	0.878	0.026
7	15	1.367	0.064
8	15	1.350	0.030
9	15	1.289	0.055

Average Pseudo-First Order Rate Constant

Equivalence	Average Rate (k_{obs}) (s^{-1})	Error
7	0.710	0.041
10	0.898	0.035
15	1.335	0.034

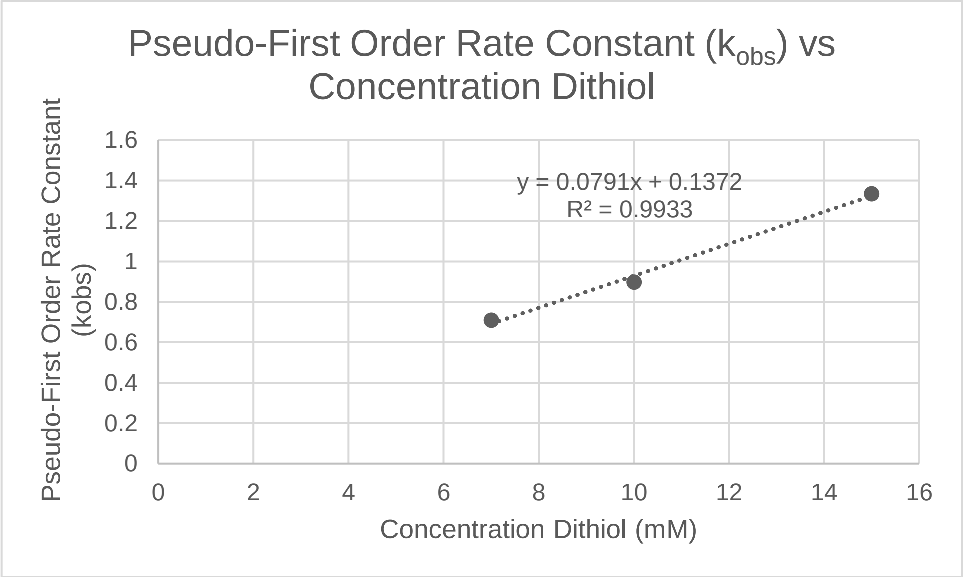

Second Order Rate Constant: 0.08 ± 0.01 M^{-1} s^{-1}

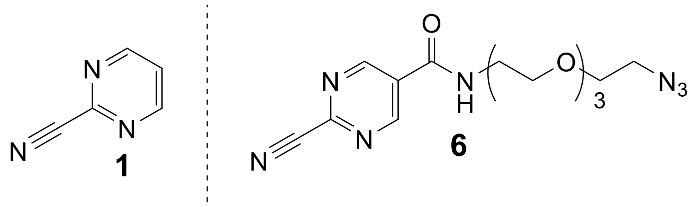

1

6

2-pyrimidine carbonitrile (**1**) (10.5 mg) or (**6**) was dissolved in acetonitrile (20 mL) to create a 5 mM stock solution. A stock solution of varying equivalence of sodium 2,3-dimercaptopropane sulfonate (DMPS) in phosphate buffer PB (8 mL, pH 7.4, 0.25 M) was made. The stock of **1** was added (2 mL) to the DMPS stock to give **1** at 1 mM in 10 mL with either 7, 10 or 15 equiv. DMPS. An aliquot of this was taken and submitted for NMR analysis at different time points.

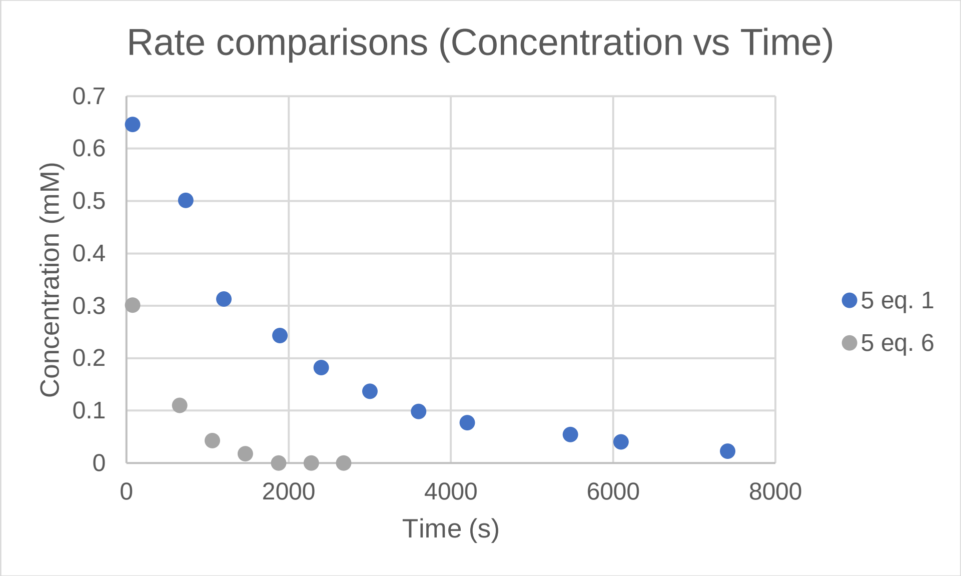

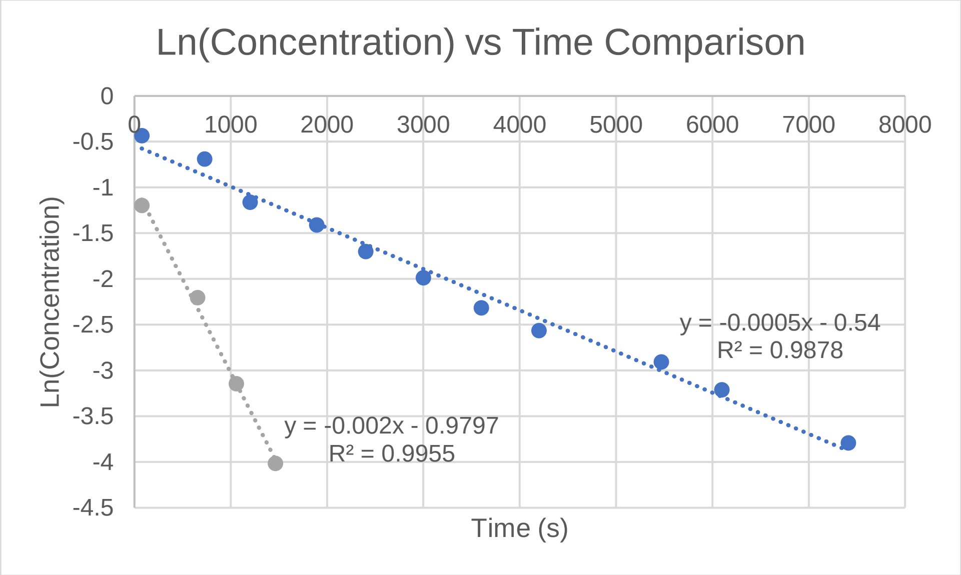

Pseudo-First Order Rate Constant Comparison

Compound	Rate Constant (s-1)	Error
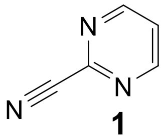 **1**	0.450	0.017
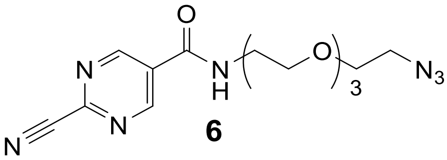 **6**	2.046	0.097

References

1 E. H. Mørkved and C. Wang, *J. für Prakt. Chemie/Chemiker-Zeitung*, 1997, **339**, 473–476.

2 O. Ovdiichuk, O. Hordiyenko, E. Fotou, C. Gaucher, A. Arrault and M. C. Averlant-Petit, *Struct. Chem.*, 2017, **28**, 813–822.

3 S. V. G. Kudrevich Maria G; van Lier, Johan E, *Synthesis (Stuttg).*, 1994, **1994**, 779–781.

4 P. Zimcik, M. Miletin, V. Novakova, K. Kopecky, M. Nejedla, V. Stara and K. Sedlackova, *Aust. J. Chem.*, 2009, **62**, 425–433.

5 V. Novakova, P. Zimcik, M. Miletin, K. Kopecky and J. Ivincová, *Tetrahedron Lett.*, 2010, **51**, 1016–1018.

6 J. Tu, D. Svatunek, S. Parvez, A. C. Liu, B. J. Levandowski, H. J. Eckvahl, R. T. Peterson, K. N. Houk and R. M. Franzini, *Angew. Chemie Int. Ed.*, 2019, **58**, 9043–9048.

7 J. Jouha, F. Buttard, M. Lorion, C. Berthonneau, M. Khouili, M.-A. Hiebel, G. Guillaumet, J.-F. Brière and F. Suzenet, *Org. Lett.*, 2017, **19**, 4770–4773.

8 P. Pracht, F. Bohle and S. Grimme, *Phys. Chem. Chem. Phys.*, 2020, **22**, 7169–7192.

9 S. Grimme, *J. Chem. Theory Comput.*, 2019, **15**, 2847–2862.

10 M. J. Frisch, G. W. Trucks, H. B. Schlegel, G. E. Scuseria, M. A. Robb, J. R. Cheeseman, G. Scalmani, V. Barone, G. A. Petersson, H. Nakatsuji, X. Li, M. Caricato, A. V. Marenich, J. Bloino, B. G. Janesko, R. Gomperts, B. Mennucci, H. P. Hratchian, J. V. Ortiz, A. F. Izmaylov, J. L. Sonnenberg, Williams, F. Ding, F. Lipparini, F. Egidi, J. Goings, B. Peng, A. Petrone, T. Henderson, D. Ranasinghe, V. G. Zakrzewski, J. Gao, N. Rega, G. Zheng, W. Liang, M. Hada, M. Ehara, K. Toyota, R. Fukuda, J. Hasegawa, M. Ishida, T. Nakajima, Y. Honda, O. Kitao, H. Nakai, T. Vreven, K. Throssell, J. A. Montgomery Jr., J. E. Peralta, F. Ogliaro, M. J. Bearpark, J. J. Heyd, E. N. Brothers, K. N. Kudin, V. N. Staroverov, T. A. Keith, R. Kobayashi, J. Normand, K. Raghavachari, a. P. Rendell, J. C. Burant, S. S. Iyengar, J. Tomasi, M. Cossi, J. M. Millam, M. Klene, C. Adamo, R. Cammi, J. W. Ochterski, R. L. Martin, K. Morokuma, O. Farkas, J. B. Foresman and D. J. Fox, 2019, Gaussian 16, Revision C.01, Gaussian, Inc., Wallingford, CT.

11 A. V Marenich, C. J. Cramer and D. G. Truhlar, *J. Phys. Chem. B*, 2009, **113**, 6378–6396.

12 M. T. W. Lee, A. Maruani, J. R. Baker, S. Caddick and V. Chudasama, *Chem. Sci.*, 2016, **7**, 799–802.

13 F. Bryden, A. Maruani, H. Savoie, V. Chudasama, M. E. B. Smith, S. Caddick and R. W. Boyle, *Bioconjugate Chem.*, 2014, **25**, 611–617.

14 C. Bahou, P. A. Szijj, R. J. Spears, A. Wall, F. Javaid, A. Sattikar, E. A. Love, J. R. Baker and V. Chudasama, *Bioconjugate Chem.*, 2021, **32**, 672–679.

15 J. D. King, Y. Ma, Y.-C. Kuo, K. P. Bzymek, L. H. Goodstein, K. Meyer, R. E. Moore, D. Crow, D. M. Colcher, G. Singh, D. A. Horne and J. C. Williams, *Bioconjugate Chem.*, 2018, **29**, 2074–2081.

16 N. Forte, I. Benni, K. Karu, V. Chudasama and J. R. Baker, *Chem. Sci.*, 2019, **10**, 10919–10924.
